# Relationship between Fungal Colonisation of the Respiratory Tract in Lung Transplant Recipients and Fungal Contamination of the Hospital Environment

**DOI:** 10.1371/journal.pone.0144044

**Published:** 2015-12-02

**Authors:** Christine Bonnal, Christopher Leleu, Olivier Brugière, Christian Chochillon, Raphael Porcher, Pierre-Yves Boelle, Jean Menotti, Sandrine Houze, Jean-Christophe Lucet, Francis Derouin

**Affiliations:** 1 AP-HP, Bichat-Claude Bernard Hospital, Infection Control Unit, F-75018, Paris, France; 2 University Paris Diderot, Sorbonne Paris Cité, Paris, France; 3 University Pierre et Marie Curie-Paris 6, Paris, France; 4 AP-HP, Service de Pneumologie B, Unité de Transplantation Pulmonaire, Centre Hospitalier Universitaire Bichat-Claude Bernard, Paris, France; 5 AP-HP, Laboratory of Parasitology and Mycology, Bichat-Claude Bernard University Hospital, Paris, France; 6 Centre de Recherche Epidémiologie et Statistique Sorbonne Paris Cité, UMR 1153, Inserm, Université Paris Descartes, Paris, France; 7 INSERM, Institut Pierre Louis d’Epidémiologie et de Santé Publique–U1136, Paris, France; 8 AP-HP, Laboratory of Parasitology and Mycology, Saint-Louis University Hospital, Paris, France; 9 UMR 216, Mère et enfants face aux infections tropicales, Faculté des Sciences Pharmaceutiques et Biologiques, Université Paris Descartes, Paris, France; 10 Paris Diderot University, IAME, UMR 1137, F-75018 Paris, France; Postgraduate Institute of Medical Education and Research, INDIA

## Abstract

**Background:**

Aspergillus colonisation is frequently reported after lung transplantation. The question of whether aspergillus colonisation is related to the hospital environment is crucial to prevention.

**Method:**

To elucidate this question, a prospective study of aspergillus colonisation after lung transplantation, along with a mycological survey of the patient environment, was performed.

**Results:**

Forty-four consecutive patients were included from the day of lung transplantation and then examined weekly for aspergillus colonisation until hospital discharge. Environmental fungal contamination of each patient was followed weekly via air and surface sampling. Twelve patients (27%) had transient aspergillus colonisation, occurring 1–13 weeks after lung transplantation, without associated manifestation of aspergillosis. Responsible *Aspergillus* species were *A*. *fumigatus* (6), *A*. *niger* (3), *A*. *sydowii* (1), *A*. *calidoustus* (1) and *Aspergillus sp*. (1). In the environment, contamination by *Penicillium* and *Aspergillus* was predominant. Multivariate analysis showed a significant association between occurrence of aspergillus colonisation and fungal contamination of the patient’s room, either by *Aspergillus* spp. in the air or by *A*.*fumigatus* on the floor. Related clinical and environmental isolates were genotyped in 9 cases of aspergillus colonisation. For *A*. *fumigatus* (4 cases), two identical microsatellite profiles were found between clinical and environmental isolates collected on distant dates or locations. For other *Aspergillus* species, isolates were different in 2 cases; in 3 cases of aspergillus colonisation by *A*. *sydowii*, *A*. *niger* and *A*. *calidoustus*, similarity between clinical and environmental internal transcribed spacer and tubulin sequences was >99%.

**Conclusion:**

Taken together, these results support the hypothesis of environmental risk of hospital acquisition of aspergillus colonisation in lung transplant recipients.

## Introduction

Solid-organ transplant recipients are at high risk of acquiring *Aspergillus* infection, with an average incidence of 6% in lung transplants (LT), higher than after other solid organ transplantations [1–3].

In LT recipients, aspergillosis possesses particularities linked to the type of transplanted organ [4]. The frequency of respiratory tract colonisation is high, between 22 and 85%, and most often occurs within the first 6-months post-transplant [5]. Whether or not it is associated with infection, colonisation results in increased mortality at 5 and 10 years [6].

The key to the prevention of aspergillosis lies in whether the infection is hospital- or community-acquired. If a relationship between fungal contamination of the hospital environment and the incidence of *Aspergillus* colonisation (AC) or other clinical manifestations of aspergillosis under our normal conditions of LT could be proven, specific measures should be taken to prevent aspergillosis contamination and colonisation during the hospitalization in post-transplantation period.

In order to evaluate this potential relationship, we initiated a prospective study of fungal colonisation by *Aspergillus* spp. after LT together with patient environmental surveillance.

## Patients, Materials and Methods

### Ethics Statement

The Institutional Review Board (Comité d’Evaluation de l’Ethique des projets de Recherche Biomédicale, Hôpital Bichat Claude Bernard, 46, rue Henri Huchard, 75018 PARIS, France, 21 mai 2010) approved the study protocol and did not require written informed consent from the participants. However, patients were informed of the study's purpose.

### Hospital Setting

The study was carried out in the surgical intensive care unit (SICU) and the lung transplant unit (LTU) of Bichat-Claude Bernard Hospital (Paris, France). LT recipients were admitted to the SICU the day of transplantation. This unit is provided with HEPA-filtered air (99% efficiency) and maintained under positive pressure. Patients were admitted to the LTU after discharge from the SICU and maintained in this unit until discharge from the hospital. This unit is supplied with filtered air at 85% efficiency, similar to other conventional ward of the hospital. During the study period, no construction or renovation has been performed in the study wards or adjacent wards.

#### Patient inclusion and follow-up

All consecutive LT patients were included prospectively, between April 2010 and September During the SICU stay, the policy of our LT center is to strictly restrict removal of LT patients from HEPA filtered system in place in each room of patient. In this way, only transfer for CT-scan in the radiology department are performed for respiratory events in this early postoperative period. When patients left their room for any purpose, they were required to wear a FFP2 protective mask to limit aspergillosis (or fungal) contamination.

After LT, clinical, radiological and endoscopic signs, biological follow-up and antifungal treatments in relation to possible manifestation of aspergillosis were collected weekly until hospital discharge. Reports of endoscopy were recorded in patient’s medical file. A mycological examination was performed on all bronchial and alveolar sample (direct examination, culture on Sabouraud-chloramphenicol medium and identification). Detection of galactomannan (GM) antigen was performed on the serum using the Platelia *Aspergillus* technique (BioRad, Marnes-La Coquette, France).

Patients with ischemic bronchitis and *Candida* spp. colonisation were all treated by fluconazole and when an invasive candidiasis was suspected or proven, an echinocandine was administered. If an aspergillosis infection was suspected or if the patient had a special risk of developing an aspergillosis infection (for example, administration of antilymphocyte serum), voriconazole was administered.

#### Definition of colonisation and infection

Endoscopic signs of bronchial or anastomotic aspergillosis infection were assessed by senior pneumologists of LT department. Bronchial or anastomotic aspergillosis infections were defined as isolation of *Aspergillus* in culture with histopathologic evidence of tissue invasion or necrosis, ulceration or pseudomembranes on bronchoscopy, as previously reported in lung transplant recipients [7].AC was defined as identification of *Aspergillus* spp. via culture of a bronchopulmonary sample in patients with no radiologic or endoscopic signs of pulmonary or tracheobronchial invasive aspergillosis. Colonisation was considered certain if two consecutive cultures were positive in expectorations or aspirations, or if a single BAL was positive in culture [8]. Colonisation was considered probable when a culture was positive in a single expectoration or aspiration.

Invasive aspergillosis was defined using EORTC/MSG 2008 criteria [9]. Possible, probable or proven aspergillosis is distinguished using these clinical, radiological and biological criteria.

#### Monitoring of the environment

During the post-transplant hospitalization, air and surface samples were collected each week. All samples were performed by the same person using a standardized protocol over the study period. One air sample was collected in the patient's room, one in the corridor immediately adjacent to the room and one in the unit's care station. Each sample (500 L) was collected on an Air Test Omega impactor (LCB, LaSalle, France) loaded with a 90-mm diameter Petri dish containing maltose-agar chloramphenicol medium. Surface samples were taken from the patient's room via wet swabbing early in the morning prior to daily room cleaning. Five surfaces were sampled: the edge of the bed, the mobile table, the medical fluid supply and light ramp, the floor, television and windowsill. Air and surface samples were incubated for 3 to 5 days at 27°C. Colonies were counted and molds were identified by their macroscopic and microscopic appearance after lactophenol cotton blue staining. Results were expressed in cfu/m^3^ and % of positive surface samples (i.e. at least one cfu).

#### Molecular identification and typing of *Aspergillus* isolates

This study was performed on *Aspergillus* isolates belonging to the same species or section based on mycological examination, and that were found in both clinical and environmental samples taken at the same period. DNA was extracted for isolates to be genotyped and *A*. *fumigatus* strains were typed with a set of 4 microsatellites as previously described [10]. Signals were read with an automatic sequencer (ABI Prism 310, Applied Biosystems) and data were stored and analyzed with Genescan software (version 2.1, Applied Biosystems).

Multilocus sequence typing was used for *Aspergillus* isolates for which microsatellite typing was not available. Internal transcribed spacer (ITS) 1 and 2 loci, the β-tubulin gene and the actin gene were amplified and sequenced via the fluorescent dideoxynucleoside triphosphate method [11–13]. Sequences were aligned using Clustalw software (http://www.ebi.ac.uk/Tools/msa/clustalw2/), and detection of homologous sequences in GenBank's nucleotide database was carried out using Nucleotide Basic Local Alignment Search Tool software (BLASTn) (http://blast.ncbi.nlm.nih.gov/).

### Statistical Analysis

Statistical analysis of environmental data included a comparison of frequencies of positive samples (i.e. presence of at least one colony) in air and surface samples collected in the SICU and LTU.

Descriptive and comparative analyses of patients’ characteristics according to their colonisation status were carried out using Fisher's exact test for categorical variables and the Mann-Whitney U test for continuous variables.

The Pearson test was used to analyze the correlation between average weekly contamination of air (cfu/m^3^) and % positive cultures on surfaces in the SICU and LTU.

A discrete-time Cox’s proportional hazards model was used to determine the role of individual characteristics and environmental data in risk of AC. In this analysis, we focused on time to first certain or probable AC sample in patients after transplant, ignoring repeat episodes in the same patient. Patients who remained negative were censored at their date of discharge or death. Environmental variables were entered in the model as time dependent, changing at each date of sampling. All variables were first tested in a univariable model. Covariates significant at the P<0.25 level were included in a multivariable model. P-values ≤ 0.05 were considered as indicating statistical significance. All analyses were carried out using R software v.2.12 [14].

## Results

### Clinical and Biological Follow-Up of Patients

Forty-four LT recipients were included. Their main characteristics at inclusion, and potential risk factors for aspergillosis, are presented in Table 1. No patient had cystic fibrosis. Average duration of hospitalization after LT was 10 weeks (range, 3–30 weeks), with an average duration of 4.5 ± 4.5 weeks in the SICU and 5.25 ± 4.2 weeks in the LTU.

**Table 1:** Patient characteristics.

		Colonised	Non-colonised	All patients	P-value
Number of patients, (n)		12	32	44	
Age (y), mean ± SD (range)		56 ± 8 (36–65)	54 ± 10 (31–69)	54 ± 9 (31–69)	>0,25
Male gender		9 (75)	24 (75)	33 (75)	>0,25
Underlying disease					
	Pulmonary emphysema	7 (58)	10 (31)	17 (39)	>0,25
	Idiop. pulm. fibrosis	3 (25)	1 (3)	4 (9)	0,19
	COPD	4 (34)	16 (50)	20 (45)	>0,25
	Other	0	2 (6,5)	2 (5)	>0,25
Smoking		11 (92)	24 (75)	35 (80)	>0,25
Diabetes		3 (25)	4 (13)	7 (16)	>0,25
Positive CMV status		8 (67)	20 (63)	28 (64)	>0,25
Before transplantation status					
	Anti- *Aspergillus* Ab	0	2 (6)	2 (5)	>0,25
	Aspergillus colonisation	1 (8)	4 (13)	5 (11)	>0,25
	Antifungal treatment	2 (17)	5 (16)	7 (16)	>0,25
Type of transplantation					
	Single-lung	7 (58)	19 (59)	26 (59)	>0,25
	Double-lung	5 (42)	13 (41)	18 (41)	>0,25
Two-year follow-up					
	Mortality	1 (8)	13 (41)	14 (32)	0.06
	Invasive aspergillosis	0	2 (6)	2 (4.5)	>0.25

All values are n (%), unless indicated

Abbreviations: SD, standard deviation; Idiop. pulm. Fibrosis, idiopathic pulmonary fibrosis; COPD, chronic obstructive pulmonary disease, CMV, cytomegalovirus; Ab, antibody

Eight patients (18%) died during their post-operative hospital stay, but none from fungal infection. Non-colonised patients had a higher mortality than colonised patients (p = 0,06).

No patient developed invasive aspergillosis during the study period. Twelve patients (27%) had transient AC (certain: 2, probable: 10), without associated manifestation of aspergillosis. All colonised patients had single species colonisation. Their clinical and biological characteristics are presented in Table 2. AC occurred 1 to 13 weeks after transplant (median, 5 weeks); it lasted from 1 to 2 weeks. AC occurred in the SICU and LTU in 4 and 8 cases, respectively.

**Table 2:** Characteristics of 12 patients with *Aspergillus* spp. colonisation after transplantation.

Patient	Type of transplantation	Underlying disease	First day of AC (Ward)	Duration of AC*	*Aspergillus* species
2	Double-lung	Emphysema	33 (SICU)	1	*A*. *fumigatus*
3	Double-lung	Emphysema	35 (LTU)	2	*A*. *section Nidulantes*
6	Single-lung	Idiopathic fibrosis	91 (LTU)	1	*A*. *fumigatus*
11	Single-lung	Emphysema	21 (LTU)	1	*A*. *fumigatus*
14	Single-lung	Idiopathic fibrosis	32 (LTU)	1	*A*. *niger*
22	Single-lung	Idiopathic fibrosis	69 (LTU)	1	*A*. *fumigatus*
23	Single-lung	Idiopathic fibrosis	8 (SICU)	2	*A*. *fumigatus*
25	Double-lung	Emphysema/COPD	6 (SICU)	2	*A*. *niger*
26	Single-lung	Idiopathic fibrosis	49 (LTU)	1	*A*. *section Usti*
32	Double-lung	Emphysema	69 (LTU)	1	*A*. *sp*.
34	Single-lung	Emphysema	14 (SICU)	1	*A*. *fumigatus*
36	Double-lung	Emphysema	38 (LTU)	1	*A*. *niger*

*Duration expressed in weeks

Abbreviations: COPD, chronic obstructive pulmonary disease; LTU, lung transplant unit; SICU, surgical intensive care unit

The following fungal species were identified by culture: *A*. *fumigatus* (6), *A*. *niger* (3), *A*. section *Nidulantes* (1), *A*. section *Usti* (1) and an unidentified *Aspergillus* sp. (1). GM serum dosages carried out the week of AC were negative in 11 patients and positive in one. An antifungal treatment active against *Aspergillus* spp., either voriconazole (n = 8), posaconazole (n = 1) and/or caspofungin (n = 3), was used for more than one week in 9 patients, without AC (n = 4), before AC (n = 2) or after AC (n = 3) respectively.

No significant association was found between age, sex, type of transplant, underlying pathology, potential risk factors for aspergillosis or colonisation before transplantation and occurrence of AC (Table 1). There was no association between duration of hospitalization or duration of stay in the SICU and occurrence of AC (P = 0.09 and 0.17, respectively).

All patients were followed for 2 years after transplantation and 14 died during the surveillance period; 1 had AC during hospitalization, while 13 did not (p = 0.06). Among survivors, one developed sinusal aspergilloma 13 months after LT and one pulmonary aspergillosis 17 months after LT; these two patients were not colonised during initial hospitalization.

#### Environmental contamination

Mycological surveillance of the environment included 198 series of samples in the SICU (1,592 samples) and 228 (1,848 samples) in the LTU.

Average fungal air contamination was significantly higher in the LTU (15.8 cfu/m^3^ ± 24.2) than in the SICU (4.6 cfu/m^3^ ± 6.8) (p<0.001). Similarly, the mean percentage of fungal-contaminated surfaces was significantly higher in the LTU (797/1368, 58.2%) than in the SICU (646/1188, 45.4%) (p<0.001).

S1 Fig shows the percentage of positive samples in the air and surfaces for total fungal flora and for the *A*. *fumigatus* species according to sample location in the SICU and LTU. In SICU, 11,766 mold colonies and 1,328 colonies of *Aspergillus* spp were recovered, whereas, 31,758 mold colonies and 5,962 colonies of *Aspergillus* spp were recovered in LTU.

Overall, a significant correlation was found between air and surface contamination for total fungal flora (R^2^ = 0.11, p<0.002); between total fungal flora and air contamination by *Aspergillus* (R^2^ = 0.17, p<0.001); and between air contamination by *Aspergillus* and *A*. *fumigatus* on surfaces (R^2^ = 0.30, p<0.001).

Details of fungal flora in the two units are presented in S2 Fig Among 43,524 mold colonies identified in the SICU (n = 11,766) and LTU (n = 31,758), the main genera were *Penicillium* and *Aspergillus*. Among identified species of *Aspergillus*, (1,328 in the SICU and 5,962 in LTU), the section *Nidulantes* was predominant. *A*. *fumigatus* was the second most frequent species in the SICU (15.8%) and *A*. *niger* in the LTU (12.4%). We noted the presence of *Aspergillus* of the *Usti* section in both units.

### Colonisation/Environment Relationship

In 11 colonised patients, *Aspergillus* spp. was detected in the environment on dates close to (less than 2 weeks) those of AC diagnosis.

DNA genotyping of related clinical and environmental isolates was performed in 9 AC cases. In 5 cases, AC was due to non-*A*. *fumigatus* species. In two out of these 5 cases, molecular analysis enabled differentiation of clinical and environmental isolates. In three cases of AC contamination by *A*. *sydowii*, *A*. *niger* and *A*. *calidoustus*, the percentage of similarity between clinical and environmental isolates was >99%, supporting identity of isolates and an environmental source of AC (Table 3). In 4 cases, AC was due to *A*. *fumigatus*. Comparative analysis of isolates with microsatellites showed 2 identities between clinical and environmental isolates. An identical microsatellite type for the 4 loci studied was evidenced for patient 6’s isolate while he was hospitalized in the LTU and an environmental isolate from the LTU corridor collected several months later. Another identical microsatellite type for the 4 loci studied was observed between patient 22’s isolate collected while he was hospitalized in the LTU and 3 environmental isolates collected 1.5 months later in the SICU. An identical microsatellite profile was also noted for two environmental isolates collected in the LTU and in the SICU.

**Table 3:** Comparison of ITS, beta-tubulin and actin sequences of clinical isolates of *Aspergillus* and isolates of the same species collected from the environment.

			ITS	Beta-tubulin	Actin
Reference: clinical isolate, patient no 3 —*A*. *sydowii—* 06/02/2010					
Environmental isolates					
	Unit's care station	06/01/2010	1*	0	0
	Corridor	06/01/2010	3	0	0
	Television	06/15/2010	1	2	0
	Corridor	06/15/2010	3	0	0
	Unit's care station	06/15/2010	3	1	0
	Ramp	06/15/2010	3	1	0
	Corridor	06/22/2010	3	1	0
	Television	06/22/2010	1*	0	0
Reference: clinical isolate, patient no 25 —*A*. *niger—* 01/25/2011					
Environmental isolates					
	Television	01/18/2011	D	D	D
	Corridor	02/08/2011	0	1*	ND
	Unit's care station	02/08/2011	D	D	D
Reference: clinical isolate, patient no 26 –*A*. *calidoustus–* 03/16/2011					
Environmental isolates					
	Ramp	03/15/2011	D	D	D
	Floor	03/22/2011	3	2	1
	Ramp	03/29/2011	0	0	0

Results are expressed in number of single nucleotide polymorphisms (or deletion when indicated), compared with the sequence of the clinical isolate

*Deletion of one nucleotide; D: sequence with more than three single nucleotide polymorphisms; ND: not determined

### Statistical Analysis

Analysis of the relationship between environmental fungal contamination and the occurrence of AC was performed over a 359-day follow-up, from inclusion until AC or hospital discharge (Table 4). In univariate analysis, only floor contamination by *A*. *fumigatus* was found to be significantly associated with AC, and two other variables were close to significance: the presence of mold in the air (grouped data) and the presence of non-*A*. *fumigatus* in the room air. In multivariate analysis, the presence of *Aspergillus spp*. in room air and the presence of *A*. *fumigatus* on the floor of the room were significantly associated with the occurrence of AC.

**Table 4:** Univariate and multivariate analysis of relationships between fungal contamination and *Aspergillus* colonisation (Cox proportional hazards model).

		Univariable analysis		Multivariable analysis	
		HR (IC_95%_)	P-value	aHR (IC_95%_)	P-value
**All filamentous fungi except *Aspergillus* spp.**					
Air					
	Patient room	0.50 (0.16–1.62)	0.25	0.42 (0.12–1.46)	0.17
	Corridor	0.78 (0.23–2.72)	0.7		
	Nurse’s station	0.93 (0.24–3.58)	0.92		
	Air (all 3)	0.32 (0.08–1.30)	**0.11**		
Surface (patient room)					
	Bed	1.57 (0.45–5.49)	0.48		
	Table	1.24 (0.26–5.89)	0.79		
	Fluid supply ramp	1.71 (0.37–7.89)	0.49		
	Floor	1.17 (0.35–3.93)	0.8		
	Window/TV	1.62 (0.47–5.61)	0.45		
	Any (among 5)	0.61 (0.07–5.14)	0.66		
***Aspergillus* spp. except *A*. *fumigatus***					
Air					
	Patient room	2.71 (0.85–8.63)	**0.09**	3.79 (1.07–13.5)	**0.03**
	Corridor	0.52 (0.11–2.47)	0.42		
	Nurse’s station	1.07 (0.28–4.03)	0.92		
	Air (all 3)	1.25 (0.39–4.01)	0.7		
Surface (patient room)					
	Bed	1.06 (0.13–8.67)	0.95		
	Table	1.89 (0.24–15.2)	0.55		
	Fluid supply ramp	1.57 (0.47–5.21)	0.46		
	Floor	1.88 (0.49–7.19)	0.36		
	Window/TV	0.87 (0.19–4.08)	0.86		
	Any (among 5)	1.58 (0.46–5.41)	0.46		
***Aspergillus fumigatus***					
Air					
	Patient room	Undefined	1		
	Corridor	0.54 (0.07–4.32)	0.56		
	Nurse’s station	2.45 (0.49–12.2)	0.27		
	Air (all 3)	0.65 (0.14–3.03)	0.59		
Surface (patient room)					
	Bed	Undefined	1		
	Table	Undefined	1		
	Fluid supply ramp	Undefined	1		
	Floor	9.7 (0.98–96.3)	**0.05**	10.52 (0.95–117)	**0.05**
	Window/TV	3.5 (0.39–31.1)	0.26		
	Any (among 5)	2.75 (0.58–13.0)	0.2		

## Discussion

The follow-up of 44 LT recipients over an average period of 10 weeks confirmed the high risk of AC in the first few weeks after transplant [5]. The duration of hospital stay in the postoperative period in our transplantation centre was longer than usually reported, likely because our transplantation program includes aged patients with severe pre-transplant comorbidities [15].

In accordance with previous studies, *A*. *fumigatus* was the most frequently represented species, followed by *A*. *niger* and *Aspergillus* of the *Nidulantes* section. In the study period, none of the colonised patients developed invasive pulmonary infection or local bronchial complications. Two patients developed infection during the 2-year follow-up, but neither was colonised during the initial hospitalization period, suggesting that AC could not be associated with higher risk of late aspergillosis. Moreover, the mortality rate two years after transplant was not altered by the occurrence of AC during the early post-transplant period. However, non-colonised patients had a higher mortality than colonised patients. One explanation is that non-colonised patients died early after their transplantation (during the first 12 weeks for 8 patients) and were therefore less exposed to aspergillosis contamination.

Mycological follow-up of the environment near the patients showed that fungal contamination of the units was in keeping with type of ventilation, since significantly less air and surface contamination was observed in the SICU, which used HEPA-filtered air and was maintained under positive pressure, whereas the LTU was fed regular filtered air. Mycological analysis showed a high predominance of common fungal species in the hospital environment, considered to be non-pathogenic [16,17].However, *Aspergillus* spp. represented 11.3 and 18.8% of fungal species found in the SICU and LTU, respectively, and *A*. *fumigatus* accounted for 15.8 and 5.8% of the *Aspergillus spp*. found in the SICU and LTU, respectively.

Multivariate analysis showed a significant association between occurrence of AC and fungal contamination of the patient’s room, either by *Aspergillus* in the air or by *A*. *fumigatus* on the floor. This confirms prior observation on the relationship between surface fungal contamination and the incidence of invasive aspergillosis in hematology [18]. This correlation argues for mycological environmental surveillance in units receiving patients at high risk for aspergillosis [17].Sequence analysis and genotyping of clinical and environmental isolates provided information on the relationship between environmental contamination and AC. One supporting argument in favor of a relationship was the demonstration of uncommon non-*A*. *fumigatus* species in environmental and clinical samples obtained on the same dates, such as for *A*. *sydowii* and *A*. *calidoustus*, with homology in molecular analysis. These two species are potentially pathogenic in humans, with risk of resistance to antifungals for *A*. *calidoustus* [19,20]. For *A*. *fumigatus* isolates collected under similar conditions, microsatellite typing showed long persistence and circulation of some isolates in the same hospital ward or in different wards of the hospital, as previously reported. [21,22].

Together, genotype links and multivariate analysis support the environmental origin of patients’ lung colonisation by *Aspergillus* and justify preventive measures. Because of lack of power of the central ventilation system in our hospital, the filtration efficiency and air renewal rate cannot be improved in the LTU or in the SICU. Therefore, major preventive efforts should be focused on limiting patient exposure to fungal spores, especially in the LTU unit where high levels of fungal contamination of air and surface were recorded. Indeed, LT patients should not be exposed to activities that might cause aerosolization of fungal spores. Moreover, part of the prevention measures that are recommended for HSCT patients could be proposed *e*.*g*. strategies to minimize dust and routinely cleaning devices [23].

This study had several limitations: first, the follow-up was performed once weekly, thereby limiting the possibility to evaluate the genotypic diversity of *Aspergillus* spp. or comparison with clinical isolates. It is possible that more AC may be linked to environmental contamination. Second, our study was single-centered. However, air treatment of the SICU and LTU in our hospital was probably similar to that of most ICU and medical wards housing LT recipients.

Nonetheless, our study provides additional information on the environmental risk of *Aspergillus* colonisation in lung transplant recipients at the hospital. Both the identity of fungal species in clinical and environmental samples, and the significant relationship between *Aspergillus* spp. or *A*. *fumigatus* contamination of the patient’s room and AC, support the risk of hospital acquisition of AC in LT recipients via contamination of the patient's environment.

## Supporting Information

S1 Dataset Anonymized Data. (PDF)

S1 Fig Percentage of positive cultures. (A) for all molds and (B) for *Aspergillus* species in air (patient room, corridor and nurse’s station) or surface samples (bed, table, fluid supply ramp, floor, window and TV) collected in SICU (black bars) and LTU (grey bars).
(TIF)

S2 Fig Percentage of positive samples in air and on surfaces. (A) in the SICU and (B) in the LTU.
(TIF)

## Abbreviations

AC: aspergillus colonisation
LT: lung transplantation
SICU: surgical intensive care unit
LTU: lung transplant unit
GM: galactomannan
HSCT: Hematopoietic Stem Cell Transplantation
