# Supplementary material for: Relationship between Fungal Colonisation of the Respiratory Tract in Lung Transplant Recipients and Fungal Contamination of the Hospital Environment
Source: PLoS One. 2015 Dec 2;10(12):e0144044. doi: 10.1371/journal.pone.0144044 (PMC4667873; doi:10.1371/journal.pone.0144044)
Supplement: S1 Dataset — (PDF) [file pone.0144044.s001.pdf]

Pt	sem	serv	tt_af	type_tt_af	tt_is	type_tt_is	tt_ab	tazo
50	1	1	FAUX		VRAI	1-Neoral;3-Corticoïde	VRAI	FAUX
50	2	1	FAUX		VRAI	1-Neoral;3-Corticoïde	FAUX	FAUX
50	3	1	FAUX		VRAI	1-Neoral;3-Corticoïde	VRAI	FAUX
50	4	2	FAUX		VRAI	2-Tacrolimus;3-Corticoïde	VRAI	FAUX
50	5	2	FAUX		VRAI	2-Tacrolimus;3-Corticoïde	VRAI	FAUX
50	6	2	FAUX		VRAI	2-Tacrolimus;3-Corticoïde	VRAI	FAUX
50	7	2	FAUX		VRAI	2-Tacrolimus;3-Corticoïde	VRAI	FAUX
50	8	2	FAUX		VRAI	2-Tacrolimus;3-Corticoïde	VRAI	FAUX
50	9	2	FAUX		VRAI	2-Tacrolimus;3-Corticoïde	VRAI	FAUX
50	10	2	FAUX		VRAI	2-Tacrolimus;3-Corticoïde	VRAI	FAUX
51	1	1	FAUX		VRAI	1-Neoral;3-Corticoïde	VRAI	FAUX
51	2	1	FAUX		VRAI	2-Tacrolimus;3-Corticoïde	VRAI	FAUX
51	3	1	FAUX		VRAI	2-Tacrolimus;3-Corticoïde	VRAI	FAUX
51	4	2	FAUX		VRAI	2-Tacrolimus;3-Corticoïde	VRAI	FAUX
51	5	2	FAUX		VRAI	2-Tacrolimus;3-Corticoïde	VRAI	FAUX
51	6	2	FAUX		VRAI	2-Tacrolimus;3-Corticoïde	VRAI	FAUX
51	7	2	FAUX		VRAI	2-Tacrolimus;3-Corticoïde	VRAI	FAUX
51	8	2	FAUX		VRAI	2-Tacrolimus;3-Corticoïde	VRAI	FAUX
51	9	2	FAUX		VRAI	2-Tacrolimus;3-Corticoïde	VRAI	FAUX
51	10	2	FAUX		VRAI	2-Tacrolimus;3-Corticoïde	VRAI	FAUX
51	11	2	FAUX		VRAI	2-Tacrolimus;3-Corticoïde	VRAI	FAUX
51	12	2	FAUX		VRAI	2-Tacrolimus;3-Corticoïde	VRAI	FAUX
51	13	2	FAUX		VRAI	2-Tacrolimus;3-Corticoïde	VRAI	FAUX
51	14	2	FAUX		VRAI	2-Tacrolimus;3-Corticoïde	VRAI	FAUX
51	15	2	FAUX		VRAI	2-Tacrolimus;3-Corticoïde	VRAI	FAUX
51	16	2	FAUX		VRAI	2-Tacrolimus;3-Corticoïde	VRAI	FAUX
52	1	1	FAUX		VRAI	2-Tacrolimus;3-Corticoïde	VRAI	FAUX
52	2	1	FAUX		VRAI	2-Tacrolimus;3-Corticoïde	VRAI	FAUX
52	3	1	VRAI	2-Fluconazole	VRAI	2-Tacrolimus;3-Corticoïde	VRAI	FAUX
52	4	2	FAUX		VRAI	2-Tacrolimus;3-Corticoïde	VRAI	FAUX
52	5	2	FAUX		VRAI	2-Tacrolimus;3-Corticoïde	VRAI	FAUX
52	6	2	FAUX		VRAI	2-Tacrolimus;3-Corticoïde	VRAI	FAUX
53	1	1	FAUX		VRAI	1-Neoral;3-Corticoïde	VRAI	FAUX
53	2	1	FAUX		VRAI	1-Neoral;3-Corticoïde	VRAI	FAUX
53	3	1	FAUX		VRAI	1-Neoral;3-Corticoïde	VRAI	FAUX
54	1	1	FAUX		FAUX		FAUX	FAUX
54	2	2	FAUX		VRAI	1-Neoral;3-Corticoïde	VRAI	FAUX
54	3	2	FAUX		VRAI	1-Neoral;3-Corticoïde	VRAI	FAUX
54	4	2	FAUX		VRAI	1-Neoral;3-Corticoïde	VRAI	FAUX
54	5	2	FAUX		VRAI	1-Neoral;3-Corticoïde	VRAI	FAUX
54	6	2	FAUX		VRAI	1-Neoral;3-Corticoïde	VRAI	FAUX
55	1	1	FAUX		VRAI	1-Neoral;3-Corticoïde	FAUX	FAUX
55	2	2	FAUX		VRAI	1-Neoral;3-Corticoïde	VRAI	VRAI
55	3	2	FAUX		VRAI	1-Neoral;3-Corticoïde	FAUX	FAUX
55	4	2	FAUX		VRAI	2-Tacrolimus;3-Corticoïde	VRAI	FAUX
55	5	2	FAUX		VRAI	2-Tacrolimus;3-Corticoïde	VRAI	FAUX
55	6	2	FAUX		VRAI	2-Tacrolimus;3-Corticoïde	VRAI	FAUX
55	7	2	FAUX		VRAI	2-Tacrolimus;3-Corticoïde	VRAI	FAUX
55	8	2	FAUX		VRAI	2-Tacrolimus;3-Corticoïde	VRAI	FAUX
55	9	2	FAUX		VRAI	2-Tacrolimus;3-Corticoïde	VRAI	FAUX
56	1	1	VRAI	2-Fluconazole	VRAI	1-Neoral;3-Corticoïde	VRAI	VRAI
56	2	1	VRAI	2-Fluconazole	VRAI	1-Neoral;3-Corticoïde	VRAI	FAUX
56	3	1	VRAI	2-Fluconazole	VRAI	2-Tacrolimus;3-Corticoïde	VRAI	VRAI
56	4	1	FAUX		VRAI	2-Tacrolimus;3-Corticoïde	VRAI	FAUX
56	5	1	VRAI	2-Fluconazole	VRAI	2-Tacrolimus;3-Corticoïde	VRAI	FAUX

56	6	1	FAUX		VRAI	2-Tacrolimus;3-Corticoïde	VRAI	FAUX
56	7	1	FAUX		VRAI	2-Tacrolimus;3-Corticoïde	VRAI	FAUX
56	8	1	FAUX		VRAI	2-Tacrolimus;3-Corticoïde	VRAI	FAUX
56	9	1	FAUX		VRAI	2-Tacrolimus;3-Corticoïde	VRAI	FAUX
56	10	1	FAUX		VRAI	2-Tacrolimus;3-Corticoïde	VRAI	FAUX
56	11	2	FAUX		VRAI	2-Tacrolimus;3-Corticoïde	FAUX	FAUX
56	12	2	FAUX		VRAI	2-Tacrolimus;3-Corticoïde	FAUX	FAUX
56	13	2	FAUX		VRAI	2-Tacrolimus;3-Corticoïde	FAUX	FAUX
56	14	2	FAUX		VRAI	2-Tacrolimus;3-Corticoïde	FAUX	FAUX
56	15	2	FAUX		VRAI	2-Tacrolimus;3-Corticoïde	FAUX	FAUX
56	16	2	FAUX		VRAI	2-Tacrolimus;3-Corticoïde	FAUX	FAUX
56	17	2	FAUX		VRAI	2-Tacrolimus;3-Corticoïde	FAUX	FAUX
56	18	2	FAUX		VRAI	2-Tacrolimus;3-Corticoïde	FAUX	FAUX
56	19	2	FAUX		VRAI	2-Tacrolimus;3-Corticoïde	FAUX	FAUX
56	20	2	FAUX		VRAI	2-Tacrolimus;3-Corticoïde	FAUX	FAUX
57	1	1	FAUX		VRAI	1-Neoral;3-Corticoïde	VRAI	VRAI
57	2	1	VRAI	2-Fluconazole	VRAI	1-Neoral;3-Corticoïde	FAUX	FAUX
57	3	1	VRAI	2-Fluconazole	VRAI	2-Tacrolimus;3-Corticoïde	VRAI	FAUX
57	4	1	FAUX		VRAI	2-Tacrolimus;3-Corticoïde	VRAI	FAUX
57	5	2	VRAI	2-Fluconazole	VRAI	2-Tacrolimus;3-Corticoïde	VRAI	FAUX
57	6	2	VRAI	2-Fluconazole	VRAI	2-Tacrolimus;3-Corticoïde	VRAI	FAUX
57	7	2	VRAI	2-Fluconazole	VRAI	2-Tacrolimus;3-Corticoïde	VRAI	FAUX
57	8	2	VRAI	2-Fluconazole	VRAI	2-Tacrolimus;3-Corticoïde	VRAI	FAUX
57	9	2	VRAI	2-Fluconazole	VRAI	2-Tacrolimus;3-Corticoïde	VRAI	FAUX
57	10	2	VRAI	2-Fluconazole	VRAI	2-Tacrolimus;3-Corticoïde	VRAI	FAUX
57	11	2	FAUX		VRAI	2-Tacrolimus;3-Corticoïde	VRAI	FAUX
57	12	2	FAUX		VRAI	2-Tacrolimus;3-Corticoïde	VRAI	FAUX
57	13	2	FAUX		VRAI	2-Tacrolimus;3-Corticoïde	VRAI	FAUX
57	14	2	FAUX		VRAI	2-Tacrolimus;3-Corticoïde	VRAI	FAUX
57	15	2	FAUX		VRAI	2-Tacrolimus;3-Corticoïde	VRAI	FAUX
57	16	2	FAUX		VRAI	2-Tacrolimus;3-Corticoïde	VRAI	FAUX
57	17	2	FAUX		VRAI	2-Tacrolimus;3-Corticoïde	VRAI	FAUX
57	18	1	FAUX		VRAI	2-Tacrolimus;3-Corticoïde	VRAI	FAUX
58	1	1	FAUX		VRAI	1-Neoral;3-Corticoïde	VRAI	VRAI
58	2	1	FAUX		VRAI	1-Neoral;3-Corticoïde	VRAI	FAUX
58	3	1	FAUX		VRAI	1-Neoral;3-Corticoïde	VRAI	FAUX
58	4	1	FAUX		VRAI	1-Neoral;3-Corticoïde	VRAI	FAUX
59	1	1	FAUX		VRAI	1-Neoral;3-Corticoïde	VRAI	FAUX
59	2	1	FAUX		VRAI	1-Neoral;3-Corticoïde	VRAI	FAUX
59	3	2	FAUX		VRAI	2-Tacrolimus;3-Corticoïde	VRAI	FAUX
59	4	2	FAUX		VRAI	2-Tacrolimus;3-Corticoïde	FAUX	FAUX
59	5	2	FAUX		VRAI	2-Tacrolimus;3-Corticoïde	FAUX	FAUX
59	6	2	FAUX		VRAI	2-Tacrolimus;3-Corticoïde	VRAI	FAUX
59	7	2	FAUX		VRAI	2-Tacrolimus;3-Corticoïde	VRAI	FAUX
59	8	2	FAUX		VRAI	2-Tacrolimus;3-Corticoïde	VRAI	FAUX
59	9	2	FAUX		VRAI	2-Tacrolimus;3-Corticoïde	VRAI	FAUX
59	10	2	FAUX		VRAI	2-Tacrolimus;3-Corticoïde	VRAI	FAUX
59	11	2	FAUX		VRAI	2-Tacrolimus;3-Corticoïde	VRAI	FAUX
59	12	2	FAUX		VRAI	2-Tacrolimus;3-Corticoïde	VRAI	FAUX
59	13	2	FAUX		VRAI	2-Tacrolimus;3-Corticoïde	VRAI	FAUX
60	1	1	FAUX		VRAI	1-Neoral;3-Corticoïde	VRAI	FAUX
60	2	1	VRAI	2-Fluconazole	VRAI	1-Neoral;3-Corticoïde	VRAI	FAUX
60	3	2	VRAI	2-Fluconazole	VRAI	1-Neoral;3-Corticoïde	VRAI	FAUX
60	4	2	VRAI	2-Fluconazole	VRAI	1-Neoral;3-Corticoïde	FAUX	FAUX
60	5	2	FAUX		VRAI	1-Neoral;3-Corticoïde	FAUX	FAUX
61	1	1	FAUX		VRAI	1-Neoral;3-Corticoïde	VRAI	FAUX

61	2	1	FAUX		VRAI	1-Neoral;3-Corticoïde	FAUX	FAUX
61	3	1	VRAI	2-Fluconazole	VRAI	2-Tacrolimus;3-Corticoïde	VRAI	FAUX
61	4	1	VRAI	2-Fluconazole	VRAI	2-Tacrolimus;3-Corticoïde	VRAI	FAUX
61	5	1	VRAI	2-Fluconazole	VRAI	2-Tacrolimus;3-Corticoïde	VRAI	FAUX
61	6	1	VRAI	2-Fluconazole	VRAI	2-Tacrolimus;3-Corticoïde	FAUX	FAUX
61	7	1	VRAI	2-Fluconazole	VRAI	2-Tacrolimus;3-Corticoïde	VRAI	FAUX
61	8	1	VRAI	2-Fluconazole	VRAI	2-Tacrolimus;3-Corticoïde	VRAI	FAUX
61	9	1	VRAI	2-Fluconazole	VRAI	2-Tacrolimus;3-Corticoïde	VRAI	FAUX
61	10	1	FAUX		VRAI	2-Tacrolimus;3-Corticoïde	VRAI	FAUX
61	11	1	VRAI	2-Fluconazole	VRAI	2-Tacrolimus;3-Corticoïde	VRAI	FAUX
61	12	1	VRAI	2-Fluconazole	VRAI	2-Tacrolimus;3-Corticoïde	VRAI	FAUX
61	13	1	VRAI	2-Fluconazole	VRAI	2-Tacrolimus;3-Corticoïde	VRAI	FAUX
61	14	1	VRAI	2-Fluconazole	VRAI	2-Tacrolimus;3-Corticoïde	VRAI	FAUX
61	15	1	VRAI	2-Fluconazole	VRAI	2-Tacrolimus;3-Corticoïde	VRAI	FAUX
61	16	1	FAUX		VRAI	2-Tacrolimus;3-Corticoïde	VRAI	VRAI
61	17	1	FAUX		VRAI	2-Tacrolimus;3-Corticoïde	VRAI	FAUX
61	18	2	FAUX		VRAI	2-Tacrolimus;3-Corticoïde	VRAI	FAUX
61	19	2	FAUX		VRAI	2-Tacrolimus;3-Corticoïde	VRAI	FAUX
61	20	2	FAUX		VRAI	2-Tacrolimus;3-Corticoïde	VRAI	FAUX
61	21	2	FAUX		VRAI	2-Tacrolimus;3-Corticoïde	VRAI	FAUX
61	22	2	FAUX		VRAI	2-Tacrolimus;3-Corticoïde	VRAI	FAUX
61	23	2	FAUX		VRAI	2-Tacrolimus;3-Corticoïde	VRAI	FAUX
61	24	2	FAUX		VRAI	2-Tacrolimus;3-Corticoïde	VRAI	FAUX
61	25	2	FAUX		VRAI	2-Tacrolimus;3-Corticoïde	VRAI	FAUX
61	26	2	FAUX		VRAI	2-Tacrolimus;3-Corticoïde	VRAI	FAUX
64	1	1	FAUX		VRAI	1-Neoral;3-Corticoïde	VRAI	VRAI
64	2	1	FAUX		VRAI	1-Neoral;3-Corticoïde	VRAI	FAUX
64	3	2	FAUX		VRAI	1-Neoral;3-Corticoïde	VRAI	FAUX
64	4	2	FAUX		VRAI	1-Neoral;3-Corticoïde	VRAI	FAUX
65	1	1	VRAI	1-Voriconazole	VRAI	1-Neoral;3-Corticoïde	VRAI	FAUX
65	2	1	VRAI	1-Voriconazole	VRAI	2-Tacrolimus;3-Corticoïde	VRAI	VRAI
65	3	2	VRAI	1-Voriconazole	VRAI	2-Tacrolimus;3-Corticoïde	FAUX	FAUX
65	4	2	VRAI	1-Voriconazole	VRAI	2-Tacrolimus;3-Corticoïde	FAUX	FAUX
65	5	2	VRAI	1-Voriconazole	VRAI	2-Tacrolimus;3-Corticoïde	VRAI	FAUX
65	6	2	VRAI	1-Voriconazole	VRAI	2-Tacrolimus;3-Corticoïde	VRAI	FAUX
65	7	2	FAUX		FAUX		FAUX	FAUX
66	1	1	FAUX		VRAI	1-Neoral;3-Corticoïde	VRAI	VRAI
66	2	1	FAUX		VRAI	1-Neoral;3-Corticoïde	VRAI	FAUX
66	3	2	FAUX		VRAI	2-Tacrolimus;3-Corticoïde	VRAI	FAUX
66	4	2	FAUX		VRAI	2-Tacrolimus;3-Corticoïde	VRAI	FAUX
66	5	2	FAUX		VRAI	2-Tacrolimus;3-Corticoïde	VRAI	FAUX
66	6	2	FAUX		VRAI	2-Tacrolimus;3-Corticoïde	VRAI	FAUX
66	7	2	FAUX		VRAI	2-Tacrolimus;3-Corticoïde	VRAI	FAUX
66	8	2	FAUX		VRAI	2-Tacrolimus;3-Corticoïde	VRAI	FAUX
66	9	2	FAUX		VRAI	2-Tacrolimus;3-Corticoïde	VRAI	FAUX
67	1	1	FAUX		VRAI	1-Neoral;3-Corticoïde	VRAI	VRAI
67	2	2	FAUX		VRAI	1-Neoral;3-Corticoïde	VRAI	VRAI
67	3	2	FAUX		VRAI	1-Neoral;3-Corticoïde	VRAI	FAUX
67	4	2	FAUX		VRAI	1-Neoral;3-Corticoïde	VRAI	FAUX
67	5	2	FAUX		VRAI	2-Tacrolimus;3-Corticoïde	VRAI	FAUX
67	6	2	FAUX		VRAI	1-Neoral;3-Corticoïde	VRAI	FAUX
67	1	1	FAUX		VRAI	1-Neoral;3-Corticoïde	VRAI	VRAI
67	2	1	FAUX		VRAI	1-Neoral;3-Corticoïde	VRAI	FAUX
67	3	2	FAUX		VRAI	2-Tacrolimus;3-Corticoïde	VRAI	FAUX
67	4	2	FAUX		VRAI	2-Tacrolimus;3-Corticoïde	VRAI	FAUX
67	5	2	FAUX		VRAI	2-Tacrolimus;3-Corticoïde	VRAI	FAUX

67	6	2	FAUX		VRAI	2-Tacrolimus;3-Corticoïde	VRAI	FAUX
67	7	2	FAUX		VRAI	2-Tacrolimus;3-Corticoïde	VRAI	FAUX
67	8	2	VRAI	2-Fluconazole	VRAI	2-Tacrolimus;3-Corticoïde	VRAI	FAUX
67	9	2	VRAI	2-Fluconazole	VRAI	2-Tacrolimus;3-Corticoïde	VRAI	FAUX
67	10	2	VRAI	2-Fluconazole	VRAI	2-Tacrolimus;3-Corticoïde	VRAI	FAUX
70	1	1	FAUX		VRAI	1-Neoral;3-Corticoïde	VRAI	FAUX
70	2	2	FAUX		VRAI	1-Neoral;3-Corticoïde	FAUX	FAUX
70	3	2	FAUX		VRAI	1-Neoral;3-Corticoïde	FAUX	FAUX
70	4	2	FAUX		VRAI	2-Tacrolimus;3-Corticoïde	VRAI	FAUX
70	5	2	FAUX		VRAI	2-Tacrolimus;3-Corticoïde	FAUX	FAUX
70	6	2	FAUX		VRAI	2-Tacrolimus;3-Corticoïde	FAUX	FAUX
71	1	2	FAUX		VRAI	1-Neoral;3-Corticoïde	VRAI	FAUX
71	2	2	FAUX		VRAI	1-Neoral;3-Corticoïde	FAUX	FAUX
71	3	2	FAUX		VRAI	1-Neoral;3-Corticoïde	FAUX	FAUX
71	4	2	FAUX		VRAI	1-Neoral;3-Corticoïde	FAUX	FAUX
72	1	1	VRAI	2-Fluconazole	VRAI	1-Neoral;3-Corticoïde	VRAI	VRAI
72	2	1	FAUX		VRAI	2-Tacrolimus;3-Corticoïde	VRAI	VRAI
72	3	2	VRAI	2-Fluconazole	VRAI	2-Tacrolimus;3-Corticoïde	VRAI	FAUX
72	4		VRAI	1-Voriconazole	VRAI	2-Tacrolimus;3-Corticoïde	VRAI	FAUX
72	5	2	VRAI	1-Voriconazole	VRAI	2-Tacrolimus;3-Corticoïde	FAUX	FAUX
72	6	2	VRAI	1-Voriconazole	VRAI	2-Tacrolimus;3-Corticoïde	FAUX	FAUX
72	7	2	VRAI	1-Voriconazole	VRAI	2-Tacrolimus;3-Corticoïde	VRAI	FAUX
72	8	2	VRAI	1-Voriconazole	VRAI	2-Tacrolimus;3-Corticoïde	VRAI	FAUX
72	9	2	VRAI	1-Voriconazole	VRAI	2-Tacrolimus;3-Corticoïde	VRAI	FAUX
73	1	1	FAUX		VRAI	1-Neoral;3-Corticoïde	VRAI	VRAI
73	2	1	FAUX		VRAI	1-Neoral;3-Corticoïde	VRAI	VRAI
73	3	1	FAUX		VRAI	1-Neoral;3-Corticoïde	VRAI	FAUX
73	4	1	FAUX		VRAI	2-Tacrolimus;3-Corticoïde	VRAI	FAUX
73	5	1	FAUX		VRAI	2-Tacrolimus;3-Corticoïde	VRAI	FAUX
73	6	1	FAUX	2-Fluconazole	VRAI	2-Tacrolimus;3-Corticoïde	VRAI	FAUX
73	7	1	VRAI	2-Fluconazole	VRAI	2-Tacrolimus;3-Corticoïde	VRAI	FAUX
74	1	1	VRAI	1-Voriconazole	VRAI	1-Neoral;3-Corticoïde	VRAI	VRAI
74	2	2	VRAI	4-Caspofongine	VRAI	1-Neoral;3-Corticoïde	VRAI	FAUX
74	3	2	VRAI	4-Caspofongine	VRAI	1-Neoral;3-Corticoïde	VRAI	FAUX
74	4	2	VRAI	4-Caspofongine	VRAI	1-Neoral;3-Corticoïde	VRAI	FAUX
74	5	2	VRAI	4-Caspofongine	VRAI	2-Tacrolimus;3-Corticoïde	VRAI	FAUX
74	6	2	VRAI	4-Caspofongine	VRAI	2-Tacrolimus;3-Corticoïde	VRAI	FAUX
74	7	2	VRAI	1-Voriconazole	VRAI	2-Tacrolimus;3-Corticoïde	VRAI	FAUX
74	8	2	VRAI	1-Voriconazole	VRAI	2-Tacrolimus;3-Corticoïde	VRAI	FAUX
74	9	2	VRAI	1-Voriconazole	VRAI	2-Tacrolimus;3-Corticoïde	VRAI	FAUX
74	10	2	VRAI	1-Voriconazole	VRAI	2-Tacrolimus;3-Corticoïde	VRAI	FAUX
74	11	2	VRAI	1-Voriconazole	VRAI	2-Tacrolimus;3-Corticoïde	VRAI	FAUX
75	1	1	FAUX		VRAI	2-Tacrolimus;3-Corticoïde	VRAI	FAUX
75	2	1	FAUX		VRAI	1-Neoral;3-Corticoïde	VRAI	VRAI
75	3	2	FAUX		VRAI	2-Tacrolimus;3-Corticoïde	VRAI	FAUX
75	4	2	FAUX		VRAI	2-Tacrolimus;3-Corticoïde	VRAI	FAUX
75	5	2	FAUX		VRAI	2-Tacrolimus;3-Corticoïde	VRAI	FAUX
77	1	1	FAUX		VRAI	1-Neoral;3-Corticoïde	VRAI	VRAI
77	2	2	FAUX		VRAI	1-Neoral;3-Corticoïde	VRAI	FAUX
77	3	2	VRAI	2-Fluconazole	VRAI	1-Neoral;3-Corticoïde	VRAI	FAUX
77	4	2	VRAI	2-Fluconazole	VRAI	2-Tacrolimus;3-Corticoïde	VRAI	FAUX
77	5	2	VRAI	2-Fluconazole	VRAI	2-Tacrolimus;3-Corticoïde	VRAI	FAUX
77	6	2	VRAI	2-Fluconazole	VRAI	2-Tacrolimus;3-Corticoïde	VRAI	FAUX
77	7	2	VRAI	2-Fluconazole	VRAI	2-Tacrolimus;3-Corticoïde	VRAI	FAUX
77	8	2	VRAI	2-Fluconazole	VRAI	2-Tacrolimus;3-Corticoïde	VRAI	FAUX
78	1	1	FAUX		VRAI	1-Neoral;3-Corticoïde	VRAI	FAUX

78	2	1	VRAI	2-Fluconazole	VRAI	1-Neoral;3-Corticoïde	VRAI	FAUX
78	3	1	VRAI	2-Fluconazole	VRAI	1-Neoral;3-Corticoïde	VRAI	FAUX
80	1	1	FAUX		VRAI	1-Neoral;3-Corticoïde	VRAI	FAUX
80	2	2	FAUX		VRAI	1-Neoral;3-Corticoïde	VRAI	FAUX
80	3	2	FAUX		VRAI	1-Neoral;3-Corticoïde	VRAI	FAUX
80	4	1	FAUX		VRAI	1-Neoral;3-Corticoïde	VRAI	FAUX
80	5	1	VRAI	4-Caspofongine	VRAI	2-Tacrolimus;3-Corticoïde	VRAI	FAUX
80	6	1	VRAI	4-Caspofongine	VRAI	2-Tacrolimus;3-Corticoïde	VRAI	FAUX
80	7	1	VRAI	4-Caspofongine	VRAI	2-Tacrolimus;3-Corticoïde	VRAI	FAUX
80	8	1	VRAI	1-Voriconazole	VRAI	2-Tacrolimus;3-Corticoïde	VRAI	FAUX
80	9	1	VRAI	1-Voriconazole	VRAI	2-Tacrolimus;3-Corticoïde	VRAI	FAUX
80	10	2	VRAI	1-Voriconazole	VRAI	2-Tacrolimus;3-Corticoïde	VRAI	FAUX
80	11	1	VRAI	1-Voriconazole	VRAI	2-Tacrolimus;3-Corticoïde	VRAI	FAUX
80	12	1	VRAI	1-Voriconazole	VRAI	2-Tacrolimus;3-Corticoïde	VRAI	FAUX
80	13	2	VRAI	1-Voriconazole	VRAI	2-Tacrolimus;3-Corticoïde	VRAI	FAUX
80	14	2	VRAI	1-Voriconazole	VRAI	2-Tacrolimus;3-Corticoïde	VRAI	FAUX
80	15	2	VRAI		VRAI	2-Tacrolimus;3-Corticoïde	VRAI	FAUX
80	16	2	VRAI	1-Voriconazole	VRAI	2-Tacrolimus;3-Corticoïde	VRAI	FAUX
80	17	2	VRAI	1-Voriconazole	VRAI	2-Tacrolimus;3-Corticoïde	VRAI	FAUX
80	18	2	VRAI	1-Voriconazole	VRAI	2-Tacrolimus;3-Corticoïde	VRAI	FAUX
80	19	2	VRAI	1-Voriconazole	VRAI	2-Tacrolimus;3-Corticoïde	VRAI	FAUX
81	1	1	FAUX		VRAI	1-Neoral;3-Corticoïde	VRAI	FAUX
81	2	1	FAUX		VRAI	1-Neoral;3-Corticoïde	VRAI	FAUX
81	3	2	FAUX		VRAI	1-Neoral;3-Corticoïde	VRAI	FAUX
81	4	2	FAUX		VRAI	1-Neoral;3-Corticoïde	VRAI	FAUX
81	5	2	FAUX		VRAI	1-Neoral;3-Corticoïde	VRAI	FAUX
82	1	1	FAUX		VRAI	1-Neoral;3-Corticoïde	VRAI	FAUX
82	2	1	FAUX		VRAI	2-Tacrolimus;3-Corticoïde	VRAI	FAUX
82	3	1	FAUX		VRAI	2-Tacrolimus;3-Corticoïde	VRAI	FAUX
82	4	1	FAUX		VRAI	2-Tacrolimus;3-Corticoïde	VRAI	FAUX
82	5	1	VRAI	2-Fluconazole	VRAI	2-Tacrolimus;3-Corticoïde	VRAI	FAUX
82	6	1	VRAI	2-Fluconazole	VRAI	2-Tacrolimus;3-Corticoïde	VRAI	FAUX
82	7	1	FAUX		VRAI	2-Tacrolimus;3-Corticoïde	VRAI	FAUX
82	8	1	FAUX		VRAI	2-Tacrolimus;3-Corticoïde	VRAI	FAUX
82	9	1	FAUX		VRAI	2-Tacrolimus;3-Corticoïde	VRAI	FAUX
82	10	1	FAUX		VRAI	2-Tacrolimus;3-Corticoïde	VRAI	FAUX
82	11	1	FAUX		VRAI	2-Tacrolimus;3-Corticoïde	VRAI	FAUX
82	12	1	FAUX		VRAI	2-Tacrolimus;3-Corticoïde	VRAI	FAUX
82	13	1	FAUX		VRAI	2-Tacrolimus;3-Corticoïde	VRAI	FAUX
83	1	1	FAUX		VRAI	1-Neoral;3-Corticoïde	VRAI	FAUX
83	2	1	VRAI	2-Fluconazole	VRAI	2-Tacrolimus;3-Corticoïde	VRAI	FAUX
83	3	1	VRAI	2-Fluconazole	VRAI	2-Tacrolimus;3-Corticoïde	VRAI	FAUX
83	4	1	VRAI	2-Fluconazole	VRAI	2-Tacrolimus;3-Corticoïde	VRAI	FAUX
83	5	1	FAUX		VRAI	2-Tacrolimus;3-Corticoïde	VRAI	FAUX
83	6	1	FAUX		VRAI	2-Tacrolimus;3-Corticoïde	VRAI	FAUX
83	7	1	FAUX		VRAI	2-Tacrolimus;3-Corticoïde	VRAI	FAUX
83	8	1	FAUX		VRAI	2-Tacrolimus;3-Corticoïde	VRAI	FAUX
83	9	2	FAUX		VRAI	2-Tacrolimus;3-Corticoïde	VRAI	FAUX
83	10	2	FAUX		VRAI	2-Tacrolimus;3-Corticoïde	VRAI	FAUX
83	11	2	FAUX		VRAI	2-Tacrolimus;3-Corticoïde	VRAI	FAUX
83	12	2	FAUX		VRAI	2-Tacrolimus;3-Corticoïde	VRAI	FAUX
84	1	1	FAUX		VRAI	1-Neoral;3-Corticoïde	VRAI	FAUX
84	2	1	FAUX		VRAI	1-Neoral;3-Corticoïde	VRAI	FAUX
84	3	1	FAUX		VRAI	1-Neoral;3-Corticoïde	VRAI	FAUX
84	4	1	FAUX		VRAI	1-Neoral;3-Corticoïde	VRAI	FAUX
84	5	1	FAUX		VRAI	1-Neoral;3-Corticoïde	VRAI	FAUX

85	1	1	VRAI	2-Fluconazole	VRAI		VRAI	FAUX
85	2	1	VRAI	2-Fluconazole	VRAI		FAUX	FAUX
85	3	1	VRAI	2-Fluconazole	VRAI	2-Tacrolimus;3-Corticoïde	VRAI	FAUX
85	4	1	FAUX		VRAI	2-Tacrolimus;3-Corticoïde	VRAI	FAUX
85	5	1	VRAI	1-Voriconazole	VRAI	2-Tacrolimus;3-Corticoïde	VRAI	FAUX
85	6	1	VRAI	1-Voriconazole	VRAI	2-Tacrolimus;3-Corticoïde	VRAI	FAUX
85	7	1	VRAI	1-Voriconazole	VRAI	2-Tacrolimus	VRAI	FAUX
85	8	1	VRAI	1-Voriconazole	VRAI		VRAI	FAUX
85	9	1	VRAI	1-Voriconazole	VRAI		VRAI	FAUX
85	10	2	VRAI	1-Voriconazole	VRAI	2-Tacrolimus;3-Corticoïde	FAUX	FAUX
85	11	2	VRAI	1-Voriconazole	VRAI	2-Tacrolimus;3-Corticoïde	VRAI	VRAI
85	12	2	VRAI	1-Voriconazole	VRAI	2-Tacrolimus;3-Corticoïde	VRAI	FAUX
85	13	2	VRAI	1-Voriconazole	VRAI	2-Tacrolimus;3-Corticoïde	FAUX	FAUX
85	14	2	VRAI	1-Voriconazole	VRAI	2-Tacrolimus;3-Corticoïde	FAUX	FAUX
85	15	2	VRAI	1-Voriconazole	VRAI	2-Tacrolimus;3-Corticoïde	FAUX	FAUX
85	16	2	VRAI	1-Voriconazole	VRAI	2-Tacrolimus;3-Corticoïde	FAUX	FAUX
85	17	2	VRAI	1-Voriconazole	VRAI	2-Tacrolimus;3-Corticoïde	FAUX	FAUX
85	18	2	VRAI	1-Voriconazole	VRAI	2-Tacrolimus;3-Corticoïde	VRAI	FAUX
85	19	2	FAUX		FAUX		FAUX	FAUX
86	1	1	FAUX		VRAI	1-Neoral;3-Corticoïde	VRAI	FAUX
86	2	1	VRAI	4-Caspofongine	VRAI	2-Tacrolimus;3-Corticoïde	VRAI	FAUX
86	3	1	VRAI	1-Voriconazole	VRAI	2-Tacrolimus;3-Corticoïde	VRAI	FAUX
86	4	2	VRAI	1-Voriconazole	VRAI	2-Tacrolimus;3-Corticoïde	VRAI	FAUX
86	5	2	VRAI	1-Voriconazole	VRAI	2-Tacrolimus;3-Corticoïde;4-Autre	VRAI	FAUX
86	6	2	VRAI	1-Voriconazole	VRAI	2-Tacrolimus;3-Corticoïde;4-Autre	VRAI	FAUX
86	7	2	VRAI	1-Voriconazole	VRAI	2-Tacrolimus;3-Corticoïde	FAUX	FAUX
86	8	2	VRAI	1-Voriconazole	VRAI	2-Tacrolimus;3-Corticoïde	FAUX	FAUX
87	1	1	FAUX		VRAI	1-Neoral;3-Corticoïde	VRAI	FAUX
87	2	1	VRAI	2-Fluconazole	VRAI	2-Tacrolimus;3-Corticoïde	VRAI	FAUX
87	3	1	VRAI	1-Voriconazole	VRAI	3-Corticoïde;4-Autre	FAUX	FAUX
87	4	1	VRAI	1-Voriconazole	VRAI	2-Tacrolimus;3-Corticoïde	FAUX	FAUX
87	5	2	VRAI	1-Voriconazole	VRAI	2-Tacrolimus;3-Corticoïde	VRAI	FAUX
87	6		VRAI	1-Voriconazole	VRAI	2-Tacrolimus;3-Corticoïde	VRAI	FAUX
87	7		VRAI	1-Voriconazole	VRAI	2-Tacrolimus;3-Corticoïde	VRAI	FAUX
87	8		VRAI	1-Voriconazole	VRAI	2-Tacrolimus;3-Corticoïde	VRAI	FAUX
87	9		VRAI	1-Voriconazole	VRAI	2-Tacrolimus;3-Corticoïde	VRAI	FAUX
87	10		VRAI	1-Voriconazole	VRAI	2-Tacrolimus;3-Corticoïde	VRAI	FAUX
88	1	1	FAUX		VRAI	2-Tacrolimus;3-Corticoïde	VRAI	VRAI
88	2	1	VRAI	2-Fluconazole	VRAI	2-Tacrolimus;3-Corticoïde	VRAI	FAUX
88	3	1	VRAI	2-Fluconazole	VRAI	2-Tacrolimus;3-Corticoïde	VRAI	FAUX
88	4	1	VRAI	2-Fluconazole	VRAI	2-Tacrolimus;3-Corticoïde	VRAI	FAUX
88	5	2	VRAI	2-Fluconazole	VRAI	2-Tacrolimus;3-Corticoïde	VRAI	FAUX
88	6	2	VRAI	2-Fluconazole	VRAI	2-Tacrolimus;3-Corticoïde	VRAI	FAUX
89	1	1	FAUX		VRAI	1-Neoral;3-Corticoïde	VRAI	FAUX
89	2	1	FAUX		VRAI	2-Tacrolimus;3-Corticoïde	VRAI	FAUX
89	3	1	VRAI	2-Fluconazole	VRAI	2-Tacrolimus;3-Corticoïde	VRAI	FAUX
89	4	1	VRAI	2-Fluconazole	VRAI	2-Tacrolimus;3-Corticoïde	VRAI	FAUX
89	5	1	VRAI	2-Fluconazole	VRAI	2-Tacrolimus;3-Corticoïde	VRAI	FAUX
89	6	1	VRAI	2-Fluconazole	VRAI	2-Tacrolimus;3-Corticoïde	VRAI	FAUX
89	7	1	VRAI	2-Fluconazole	VRAI	2-Tacrolimus;3-Corticoïde	VRAI	FAUX
89	8	1	VRAI	2-Fluconazole	VRAI	2-Tacrolimus;3-Corticoïde	VRAI	FAUX
89	9	1	VRAI	2-Fluconazole	VRAI	2-Tacrolimus;3-Corticoïde	VRAI	FAUX
89	10	1	VRAI	2-Fluconazole	VRAI	2-Tacrolimus;3-Corticoïde	VRAI	FAUX
89	11	1	VRAI	2-Fluconazole	VRAI	2-Tacrolimus;3-Corticoïde	VRAI	FAUX
90	1	1	FAUX		VRAI	1-Neoral;3-Corticoïde	VRAI	VRAI
90	2	2	FAUX		VRAI	2-Tacrolimus;3-Corticoïde	VRAI	FAUX

90	3	2	FAUX		VRAI	2-Tacrolimus;3-Corticoïde	VRAI	FAUX
90	4	2	FAUX		VRAI	2-Tacrolimus;3-Corticoïde	VRAI	FAUX
90	5	2	FAUX		VRAI	2-Tacrolimus;3-Corticoïde	VRAI	FAUX
90	6	2	FAUX		VRAI	2-Tacrolimus;3-Corticoïde	VRAI	FAUX
91	1	1	FAUX		VRAI	1-Neoral;3-Corticoïde	VRAI	FAUX
91	2	1	FAUX		VRAI	1-Neoral;3-Corticoïde	VRAI	FAUX
91	3	2	FAUX		VRAI	1-Neoral;3-Corticoïde	VRAI	FAUX
91	4	2	FAUX		VRAI	1-Neoral;3-Corticoïde	VRAI	FAUX
91	5	2	FAUX		VRAI	1-Neoral;3-Corticoïde	VRAI	FAUX
91	6	2	FAUX		VRAI	2-Tacrolimus;3-Corticoïde	VRAI	FAUX
91	7	2	FAUX		VRAI	2-Tacrolimus;3-Corticoïde	VRAI	FAUX
91	8	2	FAUX		VRAI	2-Tacrolimus;3-Corticoïde	VRAI	FAUX
91	9	2	FAUX		VRAI	1-Neoral;3-Corticoïde	VRAI	FAUX
91	10	2	FAUX		VRAI	2-Tacrolimus;3-Corticoïde	VRAI	FAUX
91	11	2	FAUX		VRAI	2-Tacrolimus;3-Corticoïde	VRAI	FAUX
91	12	2	FAUX		VRAI	2-Tacrolimus;3-Corticoïde	VRAI	FAUX
92	1	1	VRAI	2-Fluconazole	VRAI	1-Neoral;3-Corticoïde	VRAI	FAUX
92	2	1	VRAI	2-Fluconazole	VRAI	1-Neoral;3-Corticoïde	VRAI	FAUX
92	3	1	FAUX		VRAI	2-Tacrolimus;3-Corticoïde	VRAI	FAUX
92	4	1	FAUX		VRAI	2-Tacrolimus;3-Corticoïde	VRAI	FAUX
92	5	2	FAUX		VRAI	1-Neoral;3-Corticoïde	VRAI	FAUX
92	6	2	FAUX		VRAI	2-Tacrolimus;3-Corticoïde;4-Autre	VRAI	FAUX
92	7	2	VRAI	2-Fluconazole	VRAI		FAUX	FAUX
92	8	2	VRAI	2-Fluconazole	VRAI	2-Tacrolimus;3-Corticoïde	VRAI	FAUX
92	9	2	VRAI	2-Fluconazole	VRAI	2-Tacrolimus;3-Corticoïde	FAUX	FAUX
92	10	2	VRAI	2-Fluconazole	VRAI	2-Tacrolimus;3-Corticoïde	VRAI	FAUX
92	11	2	VRAI	2-Fluconazole	VRAI	2-Tacrolimus;3-Corticoïde	VRAI	FAUX
92	12	2	VRAI	2-Fluconazole	VRAI	2-Tacrolimus;3-Corticoïde	VRAI	FAUX
92	13		FAUX		FAUX		FAUX	FAUX
93	1	1	VRAI	1-Voriconazole	VRAI	1-Neoral;3-Corticoïde	VRAI	FAUX
93	2	1	VRAI	1-Voriconazole	VRAI	2-Tacrolimus;3-Corticoïde	VRAI	FAUX
93	3	2	VRAI	1-Voriconazole	VRAI	2-Tacrolimus;3-Corticoïde	VRAI	FAUX
93	4	2	VRAI	1-Voriconazole	VRAI	2-Tacrolimus;3-Corticoïde	VRAI	FAUX
93	5	1	VRAI	1-Voriconazole	VRAI	2-Tacrolimus;3-Corticoïde	VRAI	VRAI
93	6	1	FAUX		VRAI	2-Tacrolimus;3-Corticoïde	VRAI	FAUX
93	7	1	FAUX		VRAI	2-Tacrolimus;3-Corticoïde	VRAI	FAUX
93	8	1	VRAI	1-Voriconazole	VRAI	2-Tacrolimus;3-Corticoïde	FAUX	FAUX
93	9	1	VRAI	1-Voriconazole	VRAI	2-Tacrolimus;3-Corticoïde	VRAI	FAUX
93	10	2	VRAI	1-Voriconazole	VRAI	2-Tacrolimus;3-Corticoïde	VRAI	FAUX
93	11	2	VRAI	1-Voriconazole	VRAI	2-Tacrolimus;3-Corticoïde	VRAI	VRAI
93	12	1	VRAI	1-Voriconazole	VRAI	2-Tacrolimus;3-Corticoïde	VRAI	FAUX
93	13	2	VRAI	1-Voriconazole	VRAI	2-Tacrolimus;3-Corticoïde	VRAI	FAUX
93	14	2	VRAI	1-Voriconazole	VRAI	2-Tacrolimus;3-Corticoïde	VRAI	FAUX
93	15	2	VRAI	1-Voriconazole	VRAI	2-Tacrolimus	VRAI	FAUX
93	16	2	VRAI	1-Voriconazole	VRAI	2-Tacrolimus;3-Corticoïde	VRAI	FAUX
93	17	1	VRAI	1-Voriconazole	VRAI	2-Tacrolimus;3-Corticoïde	VRAI	FAUX
93	18	1	VRAI	1-Voriconazole	VRAI	2-Tacrolimus;3-Corticoïde	VRAI	FAUX
93	19	1	VRAI	1-Voriconazole	VRAI	2-Tacrolimus;3-Corticoïde	VRAI	FAUX
93	20	1	VRAI	1-Voriconazole	VRAI	2-Tacrolimus;3-Corticoïde	VRAI	FAUX
93	21	1	VRAI	1-Voriconazole	VRAI	2-Tacrolimus;3-Corticoïde	VRAI	FAUX
93	22	2	VRAI	1-Voriconazole	VRAI	2-Tacrolimus;3-Corticoïde	VRAI	FAUX
93	23	2	VRAI	1-Voriconazole	VRAI	2-Tacrolimus;3-Corticoïde	VRAI	FAUX
93	24	2	VRAI	1-Voriconazole	VRAI	2-Tacrolimus;3-Corticoïde	VRAI	FAUX
93	25	2	VRAI	1-Voriconazole	VRAI	2-Tacrolimus;3-Corticoïde	VRAI	FAUX
93	26	2	VRAI	1-Voriconazole	VRAI	2-Tacrolimus;3-Corticoïde	VRAI	FAUX
93	27	2	VRAI	1-Voriconazole	VRAI	2-Tacrolimus;3-Corticoïde	VRAI	FAUX

93	28	2	VRAI	3-Posaconazole	VRAI	2-Tacrolimus;3-Corticoïde	VRAI	FAUX
93	29	2	VRAI	3-Posaconazole	VRAI	2-Tacrolimus;3-Corticoïde	VRAI	FAUX
93	30	2	VRAI	3-Posaconazole	VRAI	2-Tacrolimus;3-Corticoïde	VRAI	FAUX
94	1	1	VRAI	4-Caspofongine	VRAI	1-Neoral;3-Corticoïde	VRAI	VRAI
94	2	1	VRAI	2-Fluconazole	VRAI	1-Neoral;3-Corticoïde	VRAI	FAUX
94	3	1	VRAI	2-Fluconazole	VRAI	1-Neoral;3-Corticoïde	VRAI	FAUX
94	4	1	VRAI	4-Caspofongine	VRAI	2-Tacrolimus;3-Corticoïde	VRAI	FAUX
94	5	1	FAUX		VRAI	2-Tacrolimus;3-Corticoïde	FAUX	FAUX
94	6	1	FAUX		VRAI	2-Tacrolimus;3-Corticoïde	VRAI	FAUX
95	1	1	FAUX		VRAI	1-Neoral;3-Corticoïde	VRAI	FAUX
95	2	1	FAUX		VRAI	1-Neoral;3-Corticoïde	VRAI	VRAI
95	3	1	VRAI	2-Fluconazole	VRAI	1-Neoral;3-Corticoïde	VRAI	FAUX
95	4	1	FAUX		VRAI	2-Tacrolimus;3-Corticoïde	VRAI	FAUX
95	5	1	VRAI	4-Caspofongine	VRAI	2-Tacrolimus;3-Corticoïde	VRAI	FAUX
95	6	1	VRAI	4-Caspofongine	VRAI	2-Tacrolimus;3-Corticoïde	VRAI	FAUX
95	7	1	VRAI	4-Caspofongine	VRAI	2-Tacrolimus;3-Corticoïde	VRAI	FAUX
95	8	1	FAUX		VRAI	2-Tacrolimus;3-Corticoïde	VRAI	FAUX
95	9	1	FAUX		VRAI	2-Tacrolimus;3-Corticoïde	VRAI	FAUX
95	10	1	FAUX		VRAI	2-Tacrolimus;3-Corticoïde	VRAI	FAUX
95	11	1	FAUX		VRAI	2-Tacrolimus;3-Corticoïde	VRAI	FAUX
95	12	1	VRAI	4-Caspofongine	VRAI	2-Tacrolimus;3-Corticoïde	VRAI	FAUX
95	13	1	FAUX		VRAI	2-Tacrolimus;3-Corticoïde	VRAI	FAUX
95	14	1	VRAI	2-Fluconazole	VRAI	2-Tacrolimus;3-Corticoïde	VRAI	FAUX
95	15	1	VRAI	2-Fluconazole	VRAI	2-Tacrolimus;3-Corticoïde	VRAI	FAUX
95	16	1	FAUX		VRAI	2-Tacrolimus;3-Corticoïde	VRAI	FAUX
95	17	1	FAUX		VRAI	2-Tacrolimus;3-Corticoïde	VRAI	FAUX
95	18	1	FAUX		VRAI	2-Tacrolimus;3-Corticoïde	VRAI	FAUX
95	19	1	FAUX		VRAI	2-Tacrolimus;3-Corticoïde	VRAI	FAUX
96	1	1	FAUX		VRAI	1-Neoral;3-Corticoïde	VRAI	FAUX
96	2	1	FAUX		VRAI	1-Neoral;3-Corticoïde	VRAI	FAUX
96	3	1	FAUX		VRAI	1-Neoral;3-Corticoïde	VRAI	FAUX
96	4	1	FAUX		VRAI	1-Neoral;3-Corticoïde	VRAI	FAUX
96	5	1	FAUX		VRAI	2-Tacrolimus;3-Corticoïde	VRAI	FAUX
96	6	1	VRAI	1-Voriconazole	VRAI	2-Tacrolimus;3-Corticoïde	VRAI	FAUX
96	7	2	FAUX		VRAI	2-Tacrolimus;3-Corticoïde	FAUX	FAUX
96	8	2	FAUX		VRAI	2-Tacrolimus;3-Corticoïde	VRAI	FAUX
96	9	2	FAUX		VRAI	2-Tacrolimus;3-Corticoïde	VRAI	FAUX
96	10	2	FAUX		VRAI	2-Tacrolimus;3-Corticoïde	VRAI	FAUX
96	11	2	FAUX		VRAI	2-Tacrolimus;3-Corticoïde	VRAI	FAUX
96	12	2	FAUX		VRAI	2-Tacrolimus;3-Corticoïde	VRAI	FAUX
97	1	1	FAUX		VRAI	1-Neoral;3-Corticoïde	VRAI	FAUX
97	2	2	VRAI	2-Fluconazole	VRAI	1-Neoral;3-Corticoïde	VRAI	FAUX
97	3	2	VRAI	2-Fluconazole	VRAI	1-Neoral;3-Corticoïde	VRAI	FAUX
97	4	2	VRAI	2-Fluconazole	VRAI	2-Tacrolimus;3-Corticoïde	VRAI	FAUX
97	5	2	VRAI	2-Fluconazole	VRAI	2-Tacrolimus;3-Corticoïde	VRAI	FAUX
97	6	2	VRAI	2-Fluconazole	VRAI	2-Tacrolimus;3-Corticoïde	VRAI	FAUX
97	7	2	FAUX		FAUX		FAUX	FAUX
99	1	2	FAUX		VRAI	1-Neoral;3-Corticoïde	VRAI	FAUX
99	2	2	FAUX		VRAI	1-Neoral;3-Corticoïde	VRAI	FAUX
99	3		VRAI	2-Fluconazole	VRAI	1-Neoral;3-Corticoïde	VRAI	FAUX
99	4	1	VRAI	2-Fluconazole	VRAI	2-Tacrolimus;3-Corticoïde	VRAI	VRAI
99	5	2	VRAI	2-Fluconazole	VRAI	2-Tacrolimus;3-Corticoïde	VRAI	FAUX
99	6	2	FAUX		VRAI	2-Tacrolimus;3-Corticoïde	VRAI	FAUX
99	7	2	FAUX		VRAI	2-Tacrolimus;3-Corticoïde	VRAI	FAUX

date_tazo	intub	bronch	type_bronch	ed	ed_pos	cult_pos	asp_fumi_bronch	sct_nid_bronch
	FAUX	VRAI	1-Aspiration	FAUX	FAUX	VRAI	FAUX	FAUX
	FAUX	FAUX		FAUX	FAUX	FAUX	FAUX	FAUX
	FAUX	VRAI	1-Aspiration	FAUX	FAUX	FAUX	FAUX	FAUX
	FAUX	VRAI	1-Aspiration	VRAI	FAUX	FAUX	FAUX	FAUX
	FAUX	VRAI	1-Aspiration	VRAI	FAUX	VRAI	FAUX	FAUX
	FAUX	VRAI	1-Aspiration	VRAI	FAUX	VRAI	FAUX	FAUX
	FAUX	VRAI	1-Aspiration	VRAI	FAUX	VRAI	FAUX	FAUX
	FAUX	FAUX		FAUX	FAUX	FAUX	FAUX	FAUX
	FAUX	FAUX		FAUX	FAUX	FAUX	FAUX	FAUX
	FAUX	VRAI	1-Aspiration	VRAI	FAUX	VRAI	VRAI	FAUX
	VRAI	VRAI	1-Aspiration	FAUX	FAUX	VRAI	FAUX	FAUX
	FAUX	VRAI	1-Aspiration	FAUX	FAUX	VRAI	FAUX	FAUX
	FAUX	VRAI	1-Aspiration	FAUX	FAUX	VRAI	FAUX	FAUX
	FAUX	VRAI	1-Aspiration	VRAI	VRAI	VRAI	FAUX	FAUX
	FAUX	VRAI	1-Aspiration	VRAI	VRAI	VRAI	FAUX	FAUX
	FAUX	FAUX		FAUX	FAUX	FAUX	FAUX	FAUX
	FAUX	VRAI	1-Aspiration	VRAI	VRAI	VRAI	FAUX	FAUX
	FAUX	VRAI	1-Aspiration	VRAI	VRAI	VRAI	FAUX	FAUX
	FAUX	VRAI	1-Aspiration	VRAI	FAUX	VRAI	FAUX	FAUX
	FAUX	FAUX		FAUX	FAUX	FAUX	FAUX	FAUX
	FAUX	VRAI	1-Aspiration	VRAI	FAUX	VRAI	FAUX	FAUX
	FAUX	FAUX		FAUX	FAUX	FAUX	FAUX	FAUX
	FAUX	VRAI	1-Aspiration	FAUX	FAUX	VRAI	FAUX	FAUX
	FAUX	FAUX		FAUX	FAUX	FAUX	FAUX	FAUX
	FAUX	FAUX		FAUX	FAUX	FAUX	FAUX	FAUX
	FAUX	FAUX		FAUX	FAUX	FAUX	FAUX	FAUX
	VRAI	VRAI	1-Aspiration	FAUX	FAUX	VRAI	FAUX	FAUX
	FAUX	VRAI	1-Aspiration	FAUX	FAUX	VRAI	FAUX	FAUX
	FAUX	FAUX		FAUX	FAUX	FAUX	FAUX	FAUX
	FAUX	VRAI	1-Aspiration	VRAI	FAUX	VRAI	FAUX	FAUX
	FAUX	VRAI	1-Aspiration	VRAI	FAUX	VRAI	FAUX	FAUX
	FAUX	FAUX		FAUX	FAUX	FAUX	FAUX	FAUX
	VRAI	VRAI	1-Aspiration	FAUX	FAUX	FAUX	FAUX	FAUX
	VRAI	VRAI	1-Aspiration	FAUX	FAUX	VRAI	VRAI	FAUX
	VRAI	VRAI	2-LBA	VRAI	FAUX	FAUX	FAUX	FAUX
	FAUX	VRAI	1-Aspiration	FAUX	FAUX	VRAI	FAUX	FAUX
	FAUX	VRAI	1-Aspiration	FAUX	FAUX	VRAI	FAUX	FAUX
	FAUX	VRAI	1-Aspiration	VRAI	FAUX	VRAI	FAUX	FAUX
	FAUX	VRAI	1-Aspiration	VRAI	FAUX	VRAI	FAUX	FAUX
	FAUX	VRAI	1-Aspiration	VRAI	FAUX	VRAI	FAUX	FAUX
	FAUX	FAUX		FAUX	FAUX	FAUX	FAUX	FAUX
	VRAI	VRAI	1-Aspiration	VRAI	VRAI	VRAI	FAUX	FAUX
18/08/2010	FAUX	VRAI	1-Aspiration	VRAI	FAUX	VRAI	FAUX	FAUX
	FAUX	VRAI	1-Aspiration	FAUX	FAUX	VRAI	VRAI	FAUX
	FAUX	FAUX		FAUX	FAUX	FAUX	FAUX	FAUX
	FAUX	FAUX		FAUX	FAUX	FAUX	FAUX	FAUX
	FAUX	FAUX		FAUX	FAUX	FAUX	FAUX	FAUX
	FAUX	VRAI	1-Aspiration	VRAI	VRAI	VRAI	FAUX	FAUX
	FAUX	VRAI	1-Aspiration	VRAI	FAUX	VRAI	FAUX	FAUX
	FAUX	FAUX		FAUX	FAUX	FAUX	FAUX	FAUX
25/07/2011	VRAI	VRAI	1-Aspiration	FAUX	FAUX	VRAI	FAUX	FAUX
	VRAI	VRAI	1-Aspiration	FAUX	FAUX	VRAI	FAUX	FAUX
17/08/2011	VRAI	VRAI	1-Aspiration	FAUX	FAUX	VRAI	FAUX	FAUX
	FAUX	VRAI	1-Aspiration	FAUX	FAUX	VRAI	FAUX	FAUX
	FAUX	VRAI	1-Aspiration	FAUX	FAUX	FAUX	FAUX	FAUX

	FAUX	VRAI		FAUX	FAUX	VRAI	FAUX	FAUX
	FAUX	VRAI	1-Aspiration	FAUX	FAUX	FAUX	FAUX	FAUX
	FAUX	VRAI	1-Aspiration	FAUX	FAUX	FAUX	FAUX	FAUX
	FAUX	VRAI	1-Aspiration	FAUX	FAUX	FAUX	FAUX	FAUX
	FAUX	VRAI	1-Aspiration	FAUX	FAUX	FAUX	FAUX	FAUX
	FAUX	VRAI	1-Aspiration	VRAI	VRAI	VRAI	FAUX	FAUX
	FAUX	VRAI	1-Aspiration	FAUX	FAUX	FAUX	FAUX	FAUX
	FAUX	FAUX		FAUX	FAUX	FAUX	FAUX	FAUX
	FAUX	VRAI	1-Aspiration	VRAI	VRAI	VRAI	FAUX	FAUX
	FAUX	FAUX		FAUX	FAUX	FAUX	FAUX	FAUX
	FAUX	FAUX		FAUX	FAUX	FAUX	FAUX	FAUX
	FAUX	VRAI	1-Aspiration	VRAI	FAUX	VRAI	FAUX	FAUX
	FAUX	VRAI	1-Aspiration	FAUX	FAUX	FAUX	FAUX	FAUX
	FAUX	VRAI	1-Aspiration	VRAI	FAUX	VRAI	FAUX	FAUX
	FAUX	FAUX		FAUX	FAUX	FAUX	FAUX	FAUX
01/03/2011	FAUX	VRAI	1-Aspiration	FAUX	FAUX	VRAI	FAUX	FAUX
	FAUX	VRAI	1-Aspiration	FAUX	FAUX	VRAI	FAUX	FAUX
	VRAI	VRAI	1-Aspiration	VRAI	FAUX	VRAI	FAUX	FAUX
	VRAI	VRAI	1-Aspiration	VRAI	FAUX	VRAI	FAUX	FAUX
	FAUX	VRAI	1-Aspiration	FAUX	FAUX	VRAI	FAUX	FAUX
	FAUX	VRAI		VRAI	FAUX	VRAI	FAUX	FAUX
	FAUX	FAUX		FAUX	FAUX	FAUX	FAUX	FAUX
	FAUX	VRAI	1-Aspiration	VRAI	FAUX	VRAI	FAUX	FAUX
	FAUX	VRAI	1-Aspiration	VRAI	FAUX	VRAI	FAUX	FAUX
	FAUX	VRAI	1-Aspiration	VRAI	FAUX	VRAI	FAUX	FAUX
	FAUX	VRAI	1-Aspiration	VRAI	FAUX	VRAI	FAUX	FAUX
	FAUX	VRAI	1-Aspiration	VRAI	FAUX	VRAI	FAUX	FAUX
	FAUX	VRAI	1-Aspiration	VRAI	FAUX	VRAI	FAUX	FAUX
	FAUX	FAUX		FAUX	FAUX	FAUX	FAUX	FAUX
	FAUX	FAUX		FAUX	FAUX	FAUX	FAUX	FAUX
	FAUX	VRAI	1-Aspiration	VRAI	FAUX	VRAI	FAUX	FAUX
	FAUX	FAUX		FAUX	FAUX	FAUX	FAUX	FAUX
	FAUX	VRAI	1-Aspiration	VRAI	VRAI	VRAI	FAUX	FAUX
04/05/2011	VRAI	VRAI	1-Aspiration	FAUX	FAUX	VRAI	FAUX	FAUX
	VRAI	VRAI	1-Aspiration	FAUX	FAUX	VRAI	FAUX	FAUX
	VRAI	VRAI	1-Aspiration	FAUX	FAUX	VRAI	FAUX	FAUX
	VRAI	VRAI	1-Aspiration	FAUX	FAUX	VRAI	FAUX	FAUX
	VRAI	VRAI	1-Aspiration	FAUX	FAUX	FAUX	FAUX	FAUX
	FAUX	VRAI	2-LBA	VRAI	FAUX	VRAI	FAUX	FAUX
	FAUX	VRAI	1-Aspiration	VRAI	FAUX	VRAI	FAUX	FAUX
	FAUX	VRAI	1-Aspiration	VRAI	FAUX	VRAI	FAUX	FAUX
	FAUX	VRAI	1-Aspiration	VRAI	FAUX	FAUX	FAUX	FAUX
	FAUX	VRAI	1-Aspiration	VRAI	FAUX	FAUX	FAUX	FAUX
	FAUX	FAUX		FAUX	FAUX	FAUX	FAUX	FAUX
	FAUX	VRAI	1-Aspiration	VRAI	FAUX	FAUX	FAUX	FAUX
	FAUX	VRAI	1-Aspiration	VRAI	FAUX	FAUX	FAUX	FAUX
	FAUX	VRAI	1-Aspiration	VRAI	FAUX	FAUX	FAUX	FAUX
	FAUX	VRAI	1-Aspiration	VRAI	FAUX	FAUX	FAUX	FAUX
	FAUX	VRAI	1-Aspiration	VRAI	FAUX	FAUX	FAUX	FAUX
	FAUX	VRAI	1-Aspiration	VRAI	FAUX	FAUX	FAUX	FAUX
	VRAI	VRAI	1-Aspiration	FAUX	FAUX	VRAI	FAUX	FAUX
	FAUX	VRAI	1-Aspiration	VRAI	VRAI	VRAI	FAUX	FAUX
	FAUX	VRAI	1-Aspiration	VRAI	FAUX	VRAI	FAUX	FAUX
	FAUX	VRAI	1-Aspiration	VRAI	FAUX	VRAI	FAUX	FAUX
	FAUX	VRAI	1-Aspiration	FAUX	FAUX	FAUX	FAUX	FAUX
	VRAI	VRAI	1-Aspiration	FAUX	FAUX	VRAI	FAUX	FAUX

	FAUX	VRAI	1-Aspiration	VRAI	VRAI	VRAI	FAUX	FAUX
	VRAI	VRAI	1-Aspiration	VRAI	FAUX	VRAI	FAUX	FAUX
	VRAI	VRAI	1-Aspiration	VRAI	FAUX	VRAI	FAUX	FAUX
	VRAI	VRAI	1-Aspiration	FAUX	FAUX	VRAI	FAUX	FAUX
	VRAI	VRAI	1-Aspiration	FAUX	FAUX	VRAI	FAUX	FAUX
	FAUX	VRAI	1-Aspiration	FAUX	FAUX	VRAI	FAUX	FAUX
	FAUX	VRAI	1-Aspiration	FAUX	FAUX	VRAI	FAUX	FAUX
	VRAI	VRAI	1-Aspiration	FAUX	FAUX	VRAI	FAUX	FAUX
	FAUX	VRAI	1-Aspiration	FAUX	FAUX	VRAI	FAUX	FAUX
	FAUX	VRAI		FAUX	FAUX	FAUX	FAUX	FAUX
	FAUX	VRAI	1-Aspiration	FAUX	FAUX	VRAI	FAUX	FAUX
	FAUX	VRAI	1-Aspiration	FAUX	FAUX	VRAI	FAUX	FAUX
	FAUX	VRAI	1-Aspiration	FAUX	FAUX	VRAI	FAUX	FAUX
	FAUX	VRAI	1-Aspiration	FAUX	FAUX	VRAI	FAUX	FAUX
21/02/2011	FAUX	VRAI	1-Aspiration	FAUX	FAUX	VRAI	FAUX	FAUX
	FAUX	VRAI	1-Aspiration	FAUX	FAUX	VRAI	FAUX	FAUX
	FAUX	VRAI	1-Aspiration	FAUX	FAUX	FAUX	FAUX	FAUX
	FAUX	FAUX		FAUX	FAUX	FAUX	FAUX	FAUX
	FAUX	VRAI	1-Aspiration	FAUX	FAUX	FAUX	FAUX	FAUX
	FAUX	VRAI	1-Aspiration	VRAI	FAUX	FAUX	FAUX	FAUX
	FAUX	VRAI	1-Aspiration	VRAI	FAUX	FAUX	FAUX	FAUX
	FAUX	VRAI	1-Aspiration	VRAI	FAUX	FAUX	FAUX	FAUX
	FAUX	VRAI	1-Aspiration	VRAI	FAUX	VRAI	FAUX	FAUX
	FAUX	VRAI	1-Aspiration	VRAI	FAUX	FAUX	FAUX	FAUX
	FAUX	VRAI	1-Aspiration	VRAI	FAUX	VRAI	FAUX	FAUX
21/09/2010	FAUX	VRAI	1-Aspiration	VRAI	FAUX	VRAI	FAUX	FAUX
	FAUX	VRAI	1-Aspiration	FAUX	FAUX	VRAI	FAUX	FAUX
	FAUX	VRAI	1-Aspiration	VRAI	VRAI	VRAI	FAUX	FAUX
	FAUX	VRAI	1-Aspiration	VRAI	FAUX	FAUX	FAUX	FAUX
	FAUX	VRAI	1-Aspiration	VRAI	VRAI	VRAI	FAUX	FAUX
	FAUX	VRAI	1-Aspiration	VRAI	VRAI	VRAI	FAUX	FAUX
	FAUX	VRAI	1-Aspiration	VRAI	FAUX	VRAI	FAUX	FAUX
	FAUX	VRAI	1-Aspiration	VRAI	FAUX	VRAI	FAUX	FAUX
	FAUX	VRAI	1-Aspiration	VRAI	FAUX	VRAI	FAUX	FAUX
	FAUX	VRAI	1-Aspiration	FAUX	FAUX	VRAI	FAUX	FAUX
	FAUX	FAUX		FAUX	FAUX	FAUX	FAUX	FAUX
26/05/2011	VRAI	VRAI	1-Aspiration	FAUX	FAUX	FAUX	FAUX	FAUX
	FAUX	VRAI	1-Aspiration	FAUX	FAUX	FAUX	FAUX	FAUX
	FAUX	VRAI	1-Aspiration	FAUX	FAUX	FAUX	FAUX	FAUX
	FAUX	VRAI	1-Aspiration	VRAI	FAUX	FAUX	FAUX	FAUX
	FAUX	VRAI	1-Aspiration	VRAI	FAUX	FAUX	FAUX	FAUX
	FAUX	FAUX		FAUX	FAUX	FAUX	FAUX	FAUX
	FAUX	VRAI	1-Aspiration	VRAI	FAUX	VRAI	FAUX	FAUX
	FAUX	VRAI	1-Aspiration	VRAI	FAUX	FAUX	FAUX	FAUX
	FAUX	VRAI	1-Aspiration	VRAI	FAUX	FAUX	FAUX	FAUX
09/03/2011	FAUX	VRAI	1-Aspiration	FAUX	FAUX	VRAI	FAUX	FAUX
17/03/2011	FAUX	VRAI	1-Aspiration	VRAI	VRAI	VRAI	FAUX	FAUX
	FAUX	VRAI	1-Aspiration	VRAI	VRAI	VRAI	FAUX	FAUX
	FAUX	FAUX		FAUX	FAUX	FAUX	FAUX	FAUX
	FAUX	VRAI	1-Aspiration	VRAI	VRAI	VRAI	FAUX	FAUX
	FAUX	FAUX		FAUX	FAUX	FAUX	FAUX	FAUX
15/04/2011	FAUX	VRAI	1-Aspiration	FAUX	FAUX	VRAI	FAUX	FAUX
	FAUX	VRAI	1-Aspiration	FAUX	FAUX	VRAI	FAUX	FAUX
	FAUX	VRAI	1-Aspiration	VRAI	VRAI	VRAI	FAUX	FAUX
	FAUX	VRAI	1-Aspiration	VRAI	VRAI	VRAI	FAUX	FAUX
	FAUX	VRAI	1-Aspiration	FAUX	FAUX	VRAI	FAUX	FAUX

	FAUX	VRAI	1-Aspiration	FAUX	FAUX	VRAI	FAUX	FAUX
	FAUX	VRAI	1-Aspiration	VRAI	VRAI	VRAI	FAUX	FAUX
	FAUX	VRAI	1-Aspiration	VRAI	FAUX	VRAI	FAUX	FAUX
	FAUX	VRAI	1-Aspiration	VRAI	FAUX	VRAI	FAUX	FAUX
	FAUX	VRAI	1-Aspiration	VRAI	FAUX	VRAI	FAUX	FAUX
	FAUX	VRAI	1-Aspiration	FAUX	FAUX	VRAI	FAUX	FAUX
	FAUX	VRAI	1-Aspiration	VRAI	VRAI	VRAI	FAUX	FAUX
	FAUX	VRAI	1-Aspiration	FAUX	FAUX	VRAI	FAUX	FAUX
	FAUX	VRAI	1-Aspiration	FAUX	FAUX	VRAI	FAUX	FAUX
	FAUX	VRAI	1-Aspiration	VRAI	FAUX	VRAI	FAUX	FAUX
	FAUX	FAUX		FAUX	FAUX	FAUX	FAUX	FAUX
	FAUX	VRAI	1-Aspiration	VRAI	VRAI	VRAI	FAUX	FAUX
	FAUX	FAUX		FAUX	FAUX	FAUX	FAUX	FAUX
	FAUX	FAUX		FAUX	FAUX	FAUX	FAUX	FAUX
	FAUX	VRAI	1-Aspiration	VRAI	VRAI	VRAI	FAUX	FAUX
16/07/2010	VRAI	VRAI	1-Aspiration	FAUX	FAUX	VRAI	FAUX	FAUX
25/07/2010	FAUX	VRAI	2-LBA	VRAI	VRAI	VRAI	FAUX	FAUX
	FAUX	VRAI	2-LBA	VRAI	VRAI	VRAI	FAUX	FAUX
	FAUX	VRAI	1-Aspiration	VRAI	VRAI	VRAI	FAUX	FAUX
	FAUX	VRAI	1-Aspiration	VRAI	FAUX	VRAI	FAUX	FAUX
	FAUX	VRAI	1-Aspiration	FAUX	FAUX	FAUX	FAUX	FAUX
	FAUX	VRAI	1-Aspiration	FAUX	FAUX	FAUX	FAUX	FAUX
	FAUX	FAUX		FAUX	FAUX	FAUX	FAUX	FAUX
	FAUX	FAUX		FAUX	FAUX	FAUX	FAUX	FAUX
29/04/2011	VRAI	VRAI	1-Aspiration	FAUX	FAUX	VRAI	FAUX	FAUX
30/04/2011	VRAI	VRAI	1-Aspiration	FAUX	FAUX	VRAI	FAUX	FAUX
	VRAI	VRAI	1-Aspiration	FAUX	FAUX	VRAI	FAUX	FAUX
	FAUX	VRAI	1-Aspiration	FAUX	FAUX	VRAI	FAUX	FAUX
	FAUX	VRAI	1-Aspiration	FAUX	FAUX	VRAI	FAUX	FAUX
	VRAI	VRAI	1-Aspiration	FAUX	FAUX	VRAI	FAUX	FAUX
	VRAI	VRAI	1-Aspiration	FAUX	FAUX	FAUX	FAUX	FAUX
16/10/2010	FAUX	VRAI	1-Aspiration	FAUX	FAUX	VRAI	FAUX	FAUX
	FAUX	VRAI	1-Aspiration	VRAI	VRAI	VRAI	FAUX	FAUX
	FAUX	VRAI	1-Aspiration	VRAI	FAUX	VRAI	FAUX	FAUX
	FAUX	VRAI	1-Aspiration	VRAI	FAUX	VRAI	FAUX	FAUX
	FAUX	VRAI	1-Aspiration	VRAI	FAUX	VRAI	FAUX	FAUX
	FAUX	VRAI	1-Aspiration	VRAI	FAUX	VRAI	FAUX	FAUX
	FAUX	VRAI	1-Aspiration	VRAI	FAUX	VRAI	FAUX	FAUX
	FAUX	VRAI	1-Aspiration	VRAI	FAUX	VRAI	FAUX	FAUX
	FAUX	VRAI	1-Aspiration	VRAI	FAUX	VRAI	FAUX	FAUX
	FAUX	VRAI	1-Aspiration	VRAI	VRAI	VRAI	FAUX	FAUX
	FAUX	VRAI	1-Aspiration	VRAI	FAUX	VRAI	FAUX	FAUX
	VRAI	VRAI	1-Aspiration	VRAI	FAUX	VRAI	FAUX	FAUX
27/10/2010	FAUX	VRAI	2-LBA	FAUX	FAUX	VRAI	FAUX	FAUX
	FAUX	FAUX		FAUX	FAUX	FAUX	FAUX	FAUX
	FAUX	VRAI	1-Aspiration	VRAI	FAUX	VRAI	FAUX	FAUX
	FAUX	FAUX		FAUX	FAUX	FAUX	FAUX	FAUX
15/03/2011	FAUX	VRAI	1-Aspiration	FAUX	FAUX	FAUX	FAUX	FAUX
	FAUX	VRAI	1-Aspiration	VRAI	FAUX	VRAI	FAUX	FAUX
	FAUX	VRAI	1-Aspiration	VRAI	VRAI	VRAI	FAUX	FAUX
	FAUX	VRAI	1-Aspiration	VRAI	VRAI	VRAI	FAUX	FAUX
	FAUX	VRAI	1-Aspiration	VRAI	FAUX	VRAI	FAUX	FAUX
	FAUX	VRAI	1-Aspiration	VRAI	FAUX	VRAI	FAUX	FAUX
	FAUX	VRAI	1-Aspiration	VRAI	FAUX	VRAI	FAUX	FAUX
	FAUX	VRAI	1-Aspiration	VRAI	FAUX	VRAI	FAUX	FAUX
	VRAI	VRAI	1-Aspiration	FAUX	FAUX	VRAI	FAUX	FAUX

VRAI	VRAI	1-Aspiration	FAUX	FAUX	VRAI	FAUX	FAUX
VRAI	VRAI	1-Aspiration	FAUX	FAUX	VRAI	FAUX	FAUX
FAUX	VRAI	1-Aspiration	FAUX	FAUX	VRAI	FAUX	FAUX
FAUX	VRAI	1-Aspiration	FAUX	FAUX	VRAI	FAUX	FAUX
FAUX	FAUX		FAUX	FAUX	FAUX	FAUX	FAUX
VRAI	VRAI	1-Aspiration	FAUX	FAUX	VRAI	FAUX	FAUX
VRAI	VRAI	1-Aspiration	FAUX	FAUX	VRAI	FAUX	FAUX
VRAI	VRAI	1-Aspiration	FAUX	FAUX	VRAI	FAUX	FAUX
FAUX	VRAI	1-Aspiration	FAUX	FAUX	VRAI	FAUX	FAUX
FAUX	VRAI	1-Aspiration	VRAI	FAUX	VRAI	FAUX	FAUX
FAUX	VRAI	1-Aspiration	FAUX	FAUX	VRAI	FAUX	FAUX
FAUX	VRAI	1-Aspiration	VRAI	FAUX	VRAI	FAUX	FAUX
FAUX	VRAI	1-Aspiration	FAUX	FAUX	VRAI	FAUX	FAUX
FAUX	VRAI	1-Aspiration	FAUX	FAUX	VRAI	FAUX	FAUX
FAUX	VRAI	1-Aspiration	VRAI	FAUX	VRAI	FAUX	FAUX
FAUX	VRAI	1-Aspiration	VRAI	FAUX	VRAI	FAUX	FAUX
FAUX	VRAI	1-Aspiration	VRAI	FAUX	VRAI	FAUX	FAUX
FAUX	VRAI	1-Aspiration	VRAI	FAUX	VRAI	FAUX	FAUX
FAUX	VRAI	1-Aspiration	VRAI	FAUX	VRAI	FAUX	FAUX
FAUX	VRAI	1-Aspiration	VRAI	FAUX	FAUX	FAUX	FAUX
FAUX	FAUX		FAUX	FAUX	FAUX	FAUX	FAUX
VRAI	VRAI	1-Aspiration	VRAI	FAUX	FAUX	FAUX	FAUX
FAUX	VRAI	1-Aspiration	VRAI	FAUX	FAUX	FAUX	FAUX
FAUX	VRAI	1-Aspiration	VRAI	VRAI	VRAI	FAUX	FAUX
FAUX	FAUX		FAUX	FAUX	FAUX	FAUX	FAUX
FAUX	VRAI	1-Aspiration	VRAI	FAUX	VRAI	FAUX	FAUX
VRAI	VRAI	1-Aspiration	FAUX	FAUX	VRAI	FAUX	FAUX
FAUX	VRAI	1-Aspiration	FAUX	FAUX	VRAI	FAUX	FAUX
VRAI	VRAI	1-Aspiration	FAUX	FAUX	VRAI	FAUX	FAUX
VRAI	VRAI	1-Aspiration	FAUX	FAUX	FAUX	FAUX	FAUX
VRAI	VRAI	1-Aspiration	FAUX	FAUX	FAUX	FAUX	FAUX
VRAI	VRAI	1-Aspiration	FAUX	FAUX	FAUX	FAUX	FAUX
VRAI	VRAI	1-Aspiration	FAUX	FAUX	FAUX	FAUX	FAUX
VRAI	VRAI	1-Aspiration	VRAI	FAUX	VRAI	FAUX	FAUX
FAUX	VRAI	1-Aspiration	FAUX	FAUX	FAUX	FAUX	FAUX
VRAI	VRAI	1-Aspiration	FAUX	FAUX	FAUX	FAUX	FAUX
VRAI	VRAI	1-Aspiration	VRAI	FAUX	VRAI	FAUX	FAUX
VRAI	VRAI	1-Aspiration	FAUX	FAUX	VRAI	FAUX	FAUX
VRAI	VRAI	1-Aspiration	FAUX	FAUX	VRAI	FAUX	FAUX
VRAI	VRAI	1-Aspiration	VRAI	VRAI	VRAI	FAUX	FAUX
VRAI	VRAI	1-Aspiration	FAUX	FAUX	VRAI	FAUX	FAUX
VRAI	VRAI	1-Aspiration	FAUX	FAUX	VRAI	FAUX	FAUX
FAUX	VRAI	1-Aspiration	FAUX	FAUX	VRAI	FAUX	FAUX
VRAI	VRAI	1-Aspiration	FAUX	FAUX	VRAI	FAUX	FAUX
VRAI	VRAI	1-Aspiration	VRAI	FAUX	FAUX	FAUX	FAUX
VRAI	VRAI	1-Aspiration	FAUX	FAUX	VRAI	FAUX	FAUX
FAUX	VRAI	2-LBA	VRAI	FAUX	VRAI	FAUX	FAUX
FAUX	VRAI	1-Aspiration	VRAI	FAUX	VRAI	FAUX	FAUX
FAUX	VRAI	1-Aspiration	VRAI	FAUX	VRAI	FAUX	VRAI
FAUX	FAUX		FAUX	FAUX	FAUX	FAUX	FAUX
FAUX	VRAI	1-Aspiration	VRAI	FAUX	VRAI	FAUX	FAUX
VRAI	VRAI	2-LBA	VRAI	FAUX	VRAI	FAUX	FAUX
FAUX	VRAI	1-Aspiration	FAUX	FAUX	FAUX	FAUX	FAUX
VRAI	VRAI	1-Aspiration	FAUX	FAUX	VRAI	FAUX	FAUX
VRAI	VRAI	2-LBA	VRAI	FAUX	VRAI	FAUX	FAUX
VRAI	VRAI	1-Aspiration	FAUX	FAUX	VRAI	FAUX	FAUX

	VRAI	VRAI	1-Aspiration	VRAI	FAUX	VRAI	FAUX	FAUX
	VRAI	VRAI	1-Aspiration	VRAI	FAUX	VRAI	FAUX	FAUX
	FAUX	VRAI	1-Aspiration	VRAI	FAUX	VRAI	FAUX	FAUX
	FAUX	VRAI	1-Aspiration	VRAI	FAUX	VRAI	FAUX	FAUX
	FAUX	VRAI	1-Aspiration	VRAI	VRAI	VRAI	VRAI	FAUX
	FAUX	VRAI	1-Aspiration	FAUX	FAUX	VRAI	FAUX	FAUX
	FAUX	VRAI	2-LBA	VRAI	FAUX	FAUX	FAUX	FAUX
	FAUX	FAUX		FAUX	FAUX	FAUX	FAUX	FAUX
	FAUX	VRAI	1-Aspiration	VRAI	VRAI	VRAI	FAUX	FAUX
	FAUX	VRAI	1-Aspiration	VRAI	FAUX	FAUX	FAUX	FAUX
08/07/2010	FAUX	VRAI	1-Aspiration	VRAI	FAUX	FAUX	FAUX	FAUX
	FAUX	VRAI	1-Aspiration	VRAI	FAUX	FAUX	FAUX	FAUX
	FAUX	VRAI	1-Aspiration	VRAI	FAUX	FAUX	FAUX	FAUX
	FAUX	VRAI	1-Aspiration	VRAI	FAUX	FAUX	FAUX	FAUX
	FAUX	VRAI	1-Aspiration	VRAI	FAUX	FAUX	FAUX	FAUX
	FAUX	VRAI	1-Aspiration	VRAI	FAUX	FAUX	FAUX	FAUX
	FAUX	VRAI	1-Aspiration	VRAI	FAUX	FAUX	FAUX	FAUX
	FAUX	VRAI	1-Aspiration	VRAI	FAUX	FAUX	FAUX	FAUX
	FAUX	VRAI	1-Aspiration	VRAI	FAUX	FAUX	FAUX	FAUX
	FAUX	VRAI	1-Aspiration	VRAI	VRAI	VRAI	FAUX	FAUX
	FAUX	VRAI	1-Aspiration	VRAI	VRAI	VRAI	FAUX	FAUX
	FAUX	VRAI	1-Aspiration	VRAI	FAUX	VRAI	FAUX	FAUX
	FAUX	VRAI	1-Aspiration	FAUX	FAUX	VRAI	FAUX	FAUX
	FAUX	VRAI	1-Aspiration	FAUX	FAUX	VRAI	FAUX	FAUX
	FAUX	VRAI	1-Aspiration	VRAI	FAUX	VRAI	FAUX	FAUX
	FAUX	VRAI	1-Aspiration	VRAI	FAUX	VRAI	FAUX	FAUX
	FAUX	VRAI	1-Aspiration	VRAI	FAUX	VRAI	FAUX	FAUX
	VRAI	FAUX		FAUX	FAUX	FAUX	FAUX	FAUX
	FAUX	VRAI	1-Aspiration	FAUX	FAUX	VRAI	VRAI	FAUX
	FAUX	FAUX		FAUX	FAUX	FAUX	FAUX	FAUX
	FAUX	VRAI	1-Aspiration	VRAI	FAUX	FAUX	FAUX	FAUX
	FAUX	VRAI	2-LBA	VRAI	FAUX	VRAI	FAUX	FAUX
	FAUX	VRAI	2-LBA	VRAI	FAUX	FAUX	FAUX	FAUX
	FAUX	FAUX		FAUX	FAUX	FAUX	FAUX	FAUX
	FAUX	FAUX		FAUX	FAUX	FAUX	FAUX	FAUX
	FAUX	VRAI	1-Aspiration	VRAI	FAUX	FAUX	FAUX	FAUX
	FAUX	VRAI	1-Aspiration	VRAI	FAUX	FAUX	FAUX	FAUX
01/11/2010	VRAI	VRAI	1-Aspiration	FAUX	FAUX	VRAI	FAUX	FAUX
	FAUX	VRAI	1-Aspiration	VRAI	FAUX	VRAI	FAUX	FAUX
	FAUX	VRAI	1-Aspiration	VRAI	FAUX	VRAI	FAUX	FAUX
	FAUX	VRAI	1-Aspiration	VRAI	FAUX	VRAI	FAUX	FAUX
	FAUX	VRAI	1-Aspiration	VRAI	FAUX	FAUX	FAUX	FAUX
	FAUX	VRAI	1-Aspiration	VRAI	FAUX	FAUX	FAUX	FAUX
	FAUX	VRAI	1-Aspiration	FAUX	FAUX	VRAI	FAUX	FAUX
	VRAI	VRAI	1-Aspiration	FAUX	FAUX	VRAI	FAUX	FAUX
	FAUX	VRAI	1-Aspiration	FAUX	FAUX	VRAI	FAUX	FAUX
	VRAI	VRAI	1-Aspiration	FAUX	FAUX	VRAI	FAUX	FAUX
	FAUX	VRAI	1-Aspiration	FAUX	FAUX	VRAI	FAUX	FAUX
	FAUX	VRAI	1-Aspiration	FAUX	FAUX	VRAI	FAUX	FAUX
	VRAI	VRAI	1-Aspiration	FAUX	FAUX	VRAI	FAUX	FAUX
	FAUX	VRAI	1-Aspiration	FAUX	FAUX	VRAI	FAUX	FAUX
	FAUX	VRAI	1-Aspiration	FAUX	FAUX	FAUX	FAUX	FAUX
	FAUX	VRAI	1-Aspiration	FAUX	FAUX	FAUX	FAUX	FAUX
	VRAI	VRAI	1-Aspiration	FAUX	FAUX	FAUX	FAUX	FAUX
	FAUX	FAUX		FAUX	FAUX	FAUX	FAUX	FAUX
	FAUX	VRAI	1-Aspiration	FAUX	FAUX	VRAI	FAUX	FAUX

	FAUX	VRAI	1-Aspiration	VRAI	FAUX	VRAI	FAUX	FAUX
	FAUX	VRAI	1-Aspiration	VRAI	FAUX	VRAI	FAUX	FAUX
	FAUX	FAUX		FAUX	FAUX	FAUX	FAUX	FAUX
	FAUX	FAUX		FAUX	FAUX	FAUX	FAUX	FAUX
	FAUX	VRAI	1-Aspiration	FAUX	FAUX	VRAI	FAUX	FAUX
	FAUX	VRAI	1-Aspiration	VRAI	FAUX	VRAI	FAUX	FAUX
	FAUX	VRAI	1-Aspiration	VRAI	FAUX	VRAI	FAUX	FAUX
	FAUX	FAUX		FAUX	FAUX	FAUX	FAUX	FAUX
	FAUX	FAUX		FAUX	FAUX	FAUX	FAUX	FAUX
	FAUX	VRAI	1-Aspiration	VRAI	FAUX	VRAI	FAUX	FAUX
	FAUX	FAUX		FAUX	FAUX	FAUX	FAUX	FAUX
	FAUX	FAUX		FAUX	FAUX	FAUX	FAUX	FAUX
	FAUX	VRAI	1-Aspiration	VRAI	FAUX	VRAI	FAUX	FAUX
	FAUX	FAUX		FAUX	FAUX	FAUX	FAUX	FAUX
	FAUX	FAUX		FAUX	FAUX	FAUX	FAUX	FAUX
	FAUX	VRAI	1-Aspiration	FAUX	FAUX	VRAI	FAUX	FAUX
	FAUX	VRAI	1-Aspiration	VRAI	VRAI	VRAI	FAUX	FAUX
	FAUX	VRAI	1-Aspiration	FAUX	FAUX	VRAI	FAUX	FAUX
	FAUX	VRAI	1-Aspiration	VRAI	FAUX	VRAI	FAUX	FAUX
	FAUX	VRAI	2-LBA	VRAI	FAUX	VRAI	FAUX	FAUX
	FAUX	VRAI	1-Aspiration	FAUX	FAUX	VRAI	FAUX	FAUX
	FAUX	VRAI	1-Aspiration	FAUX	FAUX	VRAI	FAUX	FAUX
	FAUX	VRAI	1-Aspiration	VRAI	FAUX	VRAI	FAUX	FAUX
	FAUX	VRAI	2-LBA	VRAI	VRAI	VRAI	FAUX	FAUX
	FAUX	FAUX		FAUX	FAUX	FAUX	FAUX	FAUX
	FAUX	VRAI	1-Aspiration	FAUX	FAUX	FAUX	FAUX	FAUX
	FAUX	VRAI	1-Aspiration	VRAI	FAUX	VRAI	FAUX	FAUX
	FAUX	VRAI	1-Aspiration	VRAI	FAUX	VRAI	FAUX	FAUX
	FAUX	VRAI	1-Aspiration	VRAI	FAUX	VRAI	FAUX	FAUX
	FAUX	VRAI	1-Aspiration	VRAI	FAUX	FAUX	FAUX	FAUX
	FAUX	VRAI	1-Aspiration	FAUX	FAUX	FAUX	FAUX	FAUX
	FAUX	VRAI	1-Aspiration	VRAI	FAUX	FAUX	FAUX	FAUX
	FAUX	VRAI	1-Aspiration	VRAI	FAUX	FAUX	FAUX	FAUX
10/06/2010	VRAI	VRAI	2-LBA	VRAI	FAUX	FAUX	FAUX	FAUX
	FAUX	VRAI	2-LBA	VRAI	FAUX	FAUX	FAUX	FAUX
	FAUX	VRAI	1-Aspiration	FAUX	FAUX	FAUX	FAUX	FAUX
	VRAI	VRAI	1-Aspiration	VRAI	FAUX	FAUX	FAUX	FAUX
	FAUX	VRAI	1-Aspiration	VRAI	FAUX	FAUX	FAUX	FAUX
	FAUX	VRAI	1-Aspiration	VRAI	FAUX	FAUX	FAUX	FAUX
28/07/2010	FAUX	VRAI	1-Aspiration	VRAI	FAUX	FAUX	FAUX	FAUX
	FAUX	VRAI	1-Aspiration	VRAI	FAUX	FAUX	FAUX	FAUX
	FAUX	VRAI	1-Aspiration	VRAI	FAUX	VRAI	VRAI	FAUX
	FAUX	VRAI	1-Aspiration	VRAI	FAUX	FAUX	FAUX	FAUX
	FAUX	FAUX		FAUX	FAUX	FAUX	FAUX	FAUX
	FAUX	FAUX		FAUX	FAUX	FAUX	FAUX	FAUX
	VRAI	VRAI	1-Aspiration	FAUX	FAUX	FAUX	FAUX	FAUX
	FAUX	VRAI	1-Aspiration	FAUX	FAUX	FAUX	FAUX	FAUX
	FAUX	VRAI	1-Aspiration	FAUX	FAUX	FAUX	FAUX	FAUX
	FAUX	VRAI	1-Aspiration	FAUX	FAUX	FAUX	FAUX	FAUX
	FAUX	VRAI	1-Aspiration	FAUX	FAUX	FAUX	FAUX	FAUX
	FAUX	VRAI	1-Aspiration	FAUX	FAUX	FAUX	FAUX	FAUX
	FAUX	VRAI	1-Aspiration	VRAI	FAUX	FAUX	FAUX	FAUX
	FAUX	VRAI	1-Aspiration	VRAI	FAUX	FAUX	FAUX	FAUX
	FAUX	VRAI	1-Aspiration	VRAI	VRAI	FAUX	FAUX	FAUX
	FAUX	VRAI	1-Aspiration	FAUX	FAUX	FAUX	FAUX	FAUX
	FAUX	VRAI	1-Aspiration	FAUX	FAUX	FAUX	FAUX	FAUX

	FAUX	VRAI	1-Aspiration	VRAI	FAUX	FAUX	FAUX	FAUX
	FAUX	VRAI	1-Aspiration	VRAI	FAUX	FAUX	FAUX	FAUX
	FAUX	FAUX		FAUX	FAUX	FAUX	FAUX	FAUX
21/10/2010	VRAI	VRAI	1-Aspiration	FAUX	FAUX	VRAI	FAUX	FAUX
	VRAI	VRAI	1-Aspiration	FAUX	FAUX	VRAI	FAUX	FAUX
	VRAI	VRAI	1-Aspiration	FAUX	FAUX	VRAI	FAUX	FAUX
	VRAI	VRAI	1-Aspiration	FAUX	FAUX	VRAI	FAUX	FAUX
	VRAI	VRAI	1-Aspiration	FAUX	FAUX	VRAI	FAUX	FAUX
	VRAI	VRAI	1-Aspiration	FAUX	FAUX	VRAI	FAUX	FAUX
	VRAI	VRAI	1-Aspiration	FAUX	FAUX	VRAI	FAUX	FAUX
20/04/2011	VRAI	VRAI	1-Aspiration	VRAI	VRAI	VRAI	FAUX	FAUX
	FAUX	VRAI	1-Aspiration	FAUX	FAUX	VRAI	FAUX	FAUX
	VRAI	VRAI	1-Aspiration	FAUX	FAUX	VRAI	FAUX	FAUX
	FAUX	VRAI	1-Aspiration	VRAI	FAUX	VRAI	FAUX	FAUX
	FAUX	VRAI	1-Aspiration	FAUX	FAUX	VRAI	FAUX	FAUX
	FAUX	VRAI	1-Aspiration	FAUX	FAUX	VRAI	FAUX	FAUX
	FAUX	VRAI	1-Aspiration	FAUX	FAUX	VRAI	FAUX	FAUX
	FAUX	VRAI	1-Aspiration	FAUX	FAUX	VRAI	FAUX	FAUX
	FAUX	VRAI	1-Aspiration	FAUX	FAUX	VRAI	FAUX	FAUX
	FAUX	VRAI	1-Aspiration	FAUX	FAUX	VRAI	FAUX	FAUX
	FAUX	VRAI	1-Aspiration	FAUX	FAUX	VRAI	FAUX	FAUX
	VRAI	VRAI	1-Aspiration	VRAI	VRAI	VRAI	FAUX	FAUX
	VRAI	VRAI	1-Aspiration	FAUX	FAUX	VRAI	FAUX	FAUX
	VRAI	VRAI	1-Aspiration	VRAI	FAUX	VRAI	FAUX	FAUX
	VRAI	VRAI	1-Aspiration	FAUX	FAUX	VRAI	FAUX	FAUX
	VRAI	VRAI	1-Aspiration	FAUX	FAUX	VRAI	FAUX	FAUX
	VRAI	VRAI	1-Aspiration	FAUX	FAUX	FAUX	FAUX	FAUX
	VRAI	VRAI	1-Aspiration	FAUX	FAUX	VRAI	FAUX	FAUX
	FAUX	VRAI	1-Aspiration	FAUX	FAUX	VRAI	FAUX	FAUX
	FAUX	VRAI	1-Aspiration	FAUX	FAUX	VRAI	FAUX	FAUX
	FAUX	VRAI	1-Aspiration	VRAI	FAUX	VRAI	FAUX	FAUX
	FAUX	VRAI	1-Aspiration	FAUX	FAUX	VRAI	FAUX	FAUX
	FAUX	FAUX		FAUX	FAUX	FAUX	FAUX	FAUX
	FAUX	FAUX		FAUX	FAUX	FAUX	FAUX	FAUX
	FAUX	FAUX		FAUX	FAUX	FAUX	FAUX	FAUX
	FAUX	VRAI	1-Aspiration	VRAI	FAUX	VRAI	FAUX	FAUX
	FAUX	VRAI	1-Aspiration	VRAI	FAUX	VRAI	FAUX	FAUX
	FAUX	FAUX		FAUX	FAUX	FAUX	FAUX	FAUX
	FAUX	VRAI	1-Aspiration	VRAI	FAUX	FAUX	FAUX	FAUX
	FAUX	FAUX		FAUX	FAUX	FAUX	FAUX	FAUX
	FAUX	VRAI	1-Aspiration	VRAI	FAUX	VRAI	FAUX	FAUX
	FAUX	VRAI	1-Aspiration	VRAI	FAUX	VRAI	FAUX	FAUX
	FAUX	FAUX		FAUX	FAUX	FAUX	FAUX	FAUX
	FAUX	VRAI	1-Aspiration	VRAI	FAUX	VRAI	FAUX	FAUX
	FAUX	VRAI	1-Aspiration	VRAI	FAUX	VRAI	FAUX	VRAI
	FAUX	VRAI	1-Aspiration	VRAI	FAUX	VRAI	FAUX	FAUX
	FAUX	VRAI	1-Aspiration	VRAI	FAUX	VRAI	FAUX	FAUX
	FAUX	VRAI	1-Aspiration	VRAI	FAUX	FAUX	FAUX	FAUX
	FAUX	VRAI		VRAI	FAUX	FAUX	FAUX	FAUX
	FAUX	VRAI	1-Aspiration	FAUX	FAUX	FAUX	FAUX	FAUX
21/07/2011	VRAI	VRAI	1-Aspiration	FAUX	FAUX	FAUX	FAUX	FAUX
	FAUX	FAUX		FAUX	FAUX	FAUX	FAUX	FAUX
	FAUX	VRAI	1-Aspiration	VRAI	FAUX	FAUX	FAUX	FAUX
	FAUX	FAUX		FAUX	FAUX	FAUX	FAUX	FAUX

asp_nid_bronch	asp_niger_bronch	candida_bronch	fusa_bronch	serum	valeur_agasp	agasp_pos
FAUX	FAUX	VRAI	FAUX	VRAI	0,09	FAUX
FAUX	FAUX	FAUX	FAUX	VRAI	0,09	FAUX
FAUX	FAUX	FAUX	FAUX	VRAI	0,21	FAUX
FAUX	FAUX	FAUX	FAUX	VRAI	0,15	FAUX
FAUX	FAUX	VRAI	FAUX	VRAI	0,09	FAUX
FAUX	FAUX	VRAI	FAUX	VRAI	0,25	FAUX
FAUX	FAUX	VRAI	FAUX	VRAI	0,19	FAUX
FAUX	FAUX	FAUX	FAUX	VRAI	0,13	FAUX
FAUX	FAUX	FAUX	FAUX	VRAI	0,29	FAUX
FAUX	FAUX	FAUX	FAUX	VRAI	0,09	FAUX
FAUX	FAUX	VRAI	FAUX	VRAI	0,37	FAUX
FAUX	FAUX	VRAI	FAUX	VRAI	0,44	FAUX
FAUX	FAUX	VRAI	FAUX	VRAI	0,14	FAUX
FAUX	FAUX	VRAI	FAUX	VRAI	0,15	FAUX
FAUX	FAUX	VRAI	FAUX	VRAI	0,09	FAUX
FAUX	FAUX	FAUX	FAUX	VRAI	0,14	FAUX
FAUX	FAUX	VRAI	FAUX	VRAI	0,41	FAUX
FAUX	FAUX	VRAI	FAUX	VRAI	0,34	FAUX
FAUX	FAUX	VRAI	FAUX	VRAI	0,24	FAUX
FAUX	FAUX	FAUX	FAUX	VRAI	0,79	VRAI
FAUX	FAUX	VRAI	FAUX	VRAI	0,22	FAUX
FAUX	FAUX	FAUX	FAUX	VRAI	0,22	FAUX
FAUX	FAUX	VRAI	FAUX	VRAI	0,42	FAUX
FAUX	FAUX	FAUX	FAUX	VRAI	0,09	FAUX
FAUX	FAUX	FAUX	FAUX	VRAI	0,46	FAUX
FAUX	FAUX	FAUX	FAUX	FAUX	0	FAUX
FAUX	FAUX	VRAI	FAUX	FAUX	0	FAUX
FAUX	FAUX	VRAI	FAUX	VRAI	0,15	FAUX
FAUX	FAUX	FAUX	FAUX	VRAI	0,09	FAUX
FAUX	FAUX	VRAI	FAUX	VRAI	0,13	FAUX
FAUX	FAUX	VRAI	FAUX	VRAI	0,27	FAUX
FAUX	FAUX	FAUX	FAUX	VRAI	0,3	FAUX
FAUX	FAUX	FAUX	FAUX	FAUX	0	FAUX
FAUX	FAUX	FAUX	FAUX	VRAI	0,17	FAUX
FAUX	FAUX	FAUX	FAUX	VRAI	0,17	FAUX
FAUX	FAUX	VRAI	FAUX	VRAI	0,26	FAUX
FAUX	FAUX	VRAI	FAUX	VRAI	0,09	FAUX
FAUX	FAUX	VRAI	FAUX	VRAI	0,14	FAUX
FAUX	FAUX	VRAI	FAUX	VRAI	0,09	FAUX
FAUX	FAUX	VRAI	FAUX	VRAI	0,09	FAUX
FAUX	FAUX	FAUX	FAUX	VRAI	0,11	FAUX
FAUX	FAUX	VRAI	FAUX	VRAI	0,58	VRAI
FAUX	FAUX	VRAI	FAUX	VRAI	0,18	FAUX
FAUX	FAUX	FAUX	FAUX	VRAI	0,19	FAUX
FAUX	FAUX	FAUX	FAUX	VRAI	0,15	FAUX
FAUX	FAUX	FAUX	FAUX	FAUX	0	FAUX
FAUX	FAUX	FAUX	FAUX	VRAI	0,1	FAUX
FAUX	FAUX	VRAI	FAUX	VRAI	0,1	FAUX
FAUX	FAUX	VRAI	FAUX	FAUX	0	FAUX
FAUX	FAUX	FAUX	FAUX	VRAI	0,12	FAUX
FAUX	FAUX	VRAI	FAUX	VRAI	0,09	FAUX
FAUX	FAUX	VRAI	FAUX	VRAI	0,18	FAUX
FAUX	FAUX	VRAI	FAUX	VRAI	0,24	FAUX
FAUX	FAUX	VRAI	FAUX	VRAI	0,1	FAUX
FAUX	FAUX	FAUX	FAUX	VRAI	0,25	FAUX

FAUX	FAUX	VRAI	FAUX	VRAI	0,09	FAUX
FAUX	FAUX	FAUX	FAUX	VRAI	0,1	FAUX
FAUX	FAUX	FAUX	FAUX	VRAI	0,12	FAUX
FAUX	FAUX	FAUX	FAUX	VRAI	0,09	FAUX
FAUX	FAUX	FAUX	FAUX	VRAI	0,09	FAUX
FAUX	FAUX	VRAI	FAUX	VRAI	0,09	FAUX
FAUX	FAUX	FAUX	FAUX	VRAI	0,21	FAUX
FAUX	FAUX	FAUX	FAUX	VRAI	0,23	FAUX
FAUX	FAUX	VRAI	FAUX	VRAI	0,15	FAUX
FAUX	FAUX	FAUX	FAUX	VRAI	0,37	FAUX
FAUX	FAUX	FAUX	FAUX	VRAI	0,2	FAUX
FAUX	FAUX	VRAI	FAUX	VRAI	0,14	FAUX
FAUX	FAUX	FAUX	FAUX	VRAI	0,09	FAUX
FAUX	FAUX	VRAI	FAUX	VRAI	0,09	FAUX
FAUX	FAUX	FAUX	FAUX	VRAI	0,09	FAUX
FAUX	FAUX	VRAI	FAUX	VRAI	0,49	FAUX
FAUX	FAUX	VRAI	FAUX	VRAI	0,98	VRAI
FAUX	FAUX	VRAI	FAUX	VRAI	0,13	FAUX
FAUX	FAUX	VRAI	FAUX	VRAI	0,26	FAUX
FAUX	FAUX	VRAI	FAUX	VRAI	0,21	FAUX
FAUX	FAUX	VRAI	FAUX	FAUX	0	FAUX
FAUX	FAUX	FAUX	FAUX	VRAI	0,21	FAUX
FAUX	FAUX	VRAI	FAUX	VRAI	0,09	FAUX
FAUX	FAUX	VRAI	FAUX	VRAI	0,64	VRAI
FAUX	FAUX	VRAI	FAUX	VRAI	0,49	FAUX
FAUX	FAUX	VRAI	FAUX	VRAI	0,44	FAUX
FAUX	FAUX	VRAI	FAUX	VRAI	0,66	VRAI
FAUX	FAUX	VRAI	FAUX	VRAI	1,97	VRAI
FAUX	FAUX	FAUX	FAUX	VRAI	1,51	VRAI
FAUX	FAUX	FAUX	FAUX	VRAI	0,4	FAUX
FAUX	FAUX	VRAI	FAUX	VRAI	0,42	FAUX
FAUX	FAUX	FAUX	FAUX	VRAI	0,95	VRAI
FAUX	FAUX	VRAI	FAUX	VRAI	0,28	FAUX
FAUX	FAUX	VRAI	FAUX	VRAI	0,47	FAUX
FAUX	FAUX	VRAI	FAUX	VRAI	0,1	FAUX
FAUX	FAUX	VRAI	FAUX	VRAI	0,09	FAUX
FAUX	FAUX	VRAI	FAUX	VRAI	0,09	FAUX
FAUX	FAUX	FAUX	FAUX	VRAI	0,13	FAUX
FAUX	FAUX	VRAI	FAUX	VRAI	0,19	FAUX
FAUX	FAUX	VRAI	FAUX	VRAI	0,1	FAUX
FAUX	FAUX	VRAI	FAUX	VRAI	0,1	FAUX
FAUX	FAUX	FAUX	FAUX	VRAI	0,12	FAUX
FAUX	FAUX	FAUX	FAUX	VRAI	0,22	FAUX
FAUX	FAUX	FAUX	FAUX	VRAI	0,17	FAUX
FAUX	FAUX	FAUX	FAUX	FAUX		FAUX
FAUX	FAUX	FAUX	FAUX	FAUX	0	FAUX
FAUX	FAUX	FAUX	FAUX	VRAI	0,14	FAUX
FAUX	FAUX	FAUX	FAUX	VRAI	0,09	FAUX
FAUX	FAUX	FAUX	FAUX	VRAI	0,12	FAUX
FAUX	FAUX	FAUX	FAUX	VRAI	0,13	FAUX
FAUX	FAUX	VRAI	FAUX	VRAI	0,1	FAUX
FAUX	FAUX	VRAI	FAUX	VRAI	0,21	FAUX
FAUX	FAUX	VRAI	FAUX	VRAI	0,2	FAUX
FAUX	FAUX	VRAI	FAUX	VRAI	0,19	FAUX
FAUX	FAUX	FAUX	FAUX	FAUX	0	FAUX
FAUX	FAUX	VRAI	FAUX	VRAI	0,15	FAUX

FAUX	FAUX	VRAI	FAUX	VRAI	0,16	FAUX
FAUX	FAUX	VRAI	FAUX	VRAI	0,15	FAUX
FAUX	FAUX	VRAI	FAUX	FAUX	0	FAUX
FAUX	FAUX	VRAI	FAUX	VRAI	0,1	FAUX
FAUX	FAUX	VRAI	FAUX	VRAI	0,09	FAUX
FAUX	FAUX	VRAI	FAUX	VRAI	0,14	FAUX
FAUX	FAUX	VRAI	FAUX	VRAI	0,12	FAUX
FAUX	FAUX	VRAI	FAUX	VRAI	0,18	FAUX
FAUX	FAUX	VRAI	FAUX	VRAI	0,18	FAUX
FAUX	FAUX	FAUX	FAUX	VRAI	0,1	FAUX
FAUX	FAUX	VRAI	FAUX	VRAI	0,32	FAUX
FAUX	FAUX	VRAI	FAUX	VRAI	0,31	FAUX
FAUX	FAUX	VRAI	FAUX	VRAI	0,21	FAUX
FAUX	FAUX	VRAI	FAUX	VRAI	0,56	VRAI
FAUX	FAUX	VRAI	FAUX	VRAI	3,01	VRAI
FAUX	FAUX	VRAI	FAUX	VRAI	0,47	FAUX
FAUX	FAUX	FAUX	FAUX	VRAI	0,19	FAUX
FAUX	FAUX	FAUX	FAUX	VRAI	0,18	FAUX
FAUX	FAUX	FAUX	FAUX	VRAI	0,26	FAUX
FAUX	FAUX	FAUX	FAUX	VRAI	0,48	FAUX
FAUX	FAUX	FAUX	FAUX	VRAI	0,34	FAUX
FAUX	FAUX	FAUX	FAUX	VRAI	0,34	FAUX
FAUX	FAUX	VRAI	FAUX	VRAI	0,23	FAUX
FAUX	FAUX	FAUX	FAUX	VRAI	0,14	FAUX
FAUX	FAUX	VRAI	FAUX	VRAI	0,19	FAUX
FAUX	FAUX	VRAI	FAUX	VRAI	0,98	VRAI
FAUX	FAUX	VRAI	FAUX	VRAI	0,3	FAUX
FAUX	FAUX	VRAI	FAUX	VRAI	0,25	FAUX
FAUX	FAUX	FAUX	FAUX	VRAI	0,18	FAUX
FAUX	FAUX	VRAI	FAUX	VRAI	0,45	FAUX
FAUX	FAUX	VRAI	FAUX	VRAI	0,13	FAUX
FAUX	FAUX	VRAI	FAUX	VRAI	0,21	FAUX
FAUX	FAUX	VRAI	FAUX	VRAI	0,23	FAUX
FAUX	FAUX	VRAI	FAUX	VRAI	0,18	FAUX
FAUX	FAUX	VRAI	FAUX	FAUX	0	FAUX
FAUX	FAUX	FAUX	FAUX	FAUX	0	FAUX
FAUX	FAUX	FAUX	FAUX	FAUX	0	FAUX
FAUX	FAUX	FAUX	FAUX	VRAI	0,09	FAUX
FAUX	FAUX	FAUX	FAUX	VRAI	0,09	FAUX
FAUX	FAUX	FAUX	FAUX	VRAI	0,15	FAUX
FAUX	FAUX	FAUX	FAUX	VRAI	0,2	FAUX
FAUX	FAUX	FAUX	FAUX	VRAI	0,09	FAUX
FAUX	FAUX	VRAI	FAUX	VRAI	0,21	FAUX
FAUX	FAUX	FAUX	FAUX	VRAI	0,09	FAUX
FAUX	FAUX	FAUX	FAUX	VRAI	0,09	FAUX
FAUX	FAUX	VRAI	FAUX	VRAI	0,4	FAUX
FAUX	FAUX	VRAI	FAUX	VRAI	0,82	VRAI
FAUX	FAUX	VRAI	FAUX	VRAI	0,09	FAUX
FAUX	FAUX	FAUX	FAUX	VRAI	0,13	FAUX
FAUX	FAUX	VRAI	FAUX	VRAI	1,08	VRAI
FAUX	FAUX	FAUX	FAUX	VRAI	0,61	VRAI
FAUX	FAUX	VRAI	FAUX	VRAI	0,16	FAUX
FAUX	FAUX	VRAI	FAUX	VRAI	0,15	FAUX
FAUX	FAUX	VRAI	FAUX	VRAI	0,12	FAUX
FAUX	FAUX	VRAI	FAUX	VRAI	0,34	FAUX
FAUX	FAUX	VRAI	FAUX	VRAI	0,12	FAUX

FAUX	VRAI	VRAI	FAUX	VRAI	0,32	FAUX
FAUX	FAUX	VRAI	FAUX	VRAI	1,19	VRAI
FAUX	FAUX	VRAI	FAUX	VRAI	0,84	VRAI
FAUX	FAUX	VRAI	FAUX	VRAI	0,1	FAUX
FAUX	FAUX	VRAI	FAUX	VRAI	1,42	VRAI
FAUX	FAUX	VRAI	FAUX	VRAI	0,22	FAUX
FAUX	FAUX	VRAI	FAUX	VRAI	0,15	FAUX
FAUX	FAUX	VRAI	FAUX	VRAI	0,14	FAUX
FAUX	FAUX	VRAI	FAUX	FAUX	0	FAUX
FAUX	FAUX	VRAI	FAUX	FAUX	0	FAUX
FAUX	FAUX	FAUX	FAUX	VRAI	0,18	FAUX
FAUX	FAUX	VRAI	FAUX	VRAI	0,39	FAUX
FAUX	FAUX	FAUX	FAUX	VRAI	0,22	FAUX
FAUX	FAUX	FAUX	FAUX	VRAI	0,5	VRAI
FAUX	FAUX	VRAI	FAUX	VRAI	0,14	FAUX
FAUX	FAUX	VRAI	FAUX	VRAI	0,28	FAUX
FAUX	FAUX	VRAI	FAUX	VRAI	0,94	VRAI
FAUX	FAUX	VRAI	FAUX	VRAI	1,29	VRAI
FAUX	FAUX	VRAI	FAUX	VRAI	0,23	FAUX
FAUX	FAUX	VRAI	FAUX	VRAI	0,8	VRAI
FAUX	FAUX	FAUX	FAUX	VRAI	0,34	FAUX
FAUX	FAUX	FAUX	FAUX	VRAI	0,45	FAUX
FAUX	FAUX	FAUX	FAUX	VRAI	0,46	FAUX
FAUX	FAUX	FAUX	FAUX	FAUX	0	FAUX
FAUX	FAUX	VRAI	FAUX	VRAI	0,17	FAUX
FAUX	FAUX	VRAI	FAUX	VRAI	0,14	FAUX
FAUX	FAUX	VRAI	FAUX	VRAI	0,44	FAUX
FAUX	FAUX	VRAI	FAUX	VRAI	0,44	FAUX
FAUX	FAUX	VRAI	FAUX	VRAI	0,09	FAUX
FAUX	FAUX	VRAI	FAUX	VRAI	0,09	FAUX
FAUX	FAUX	FAUX	FAUX	VRAI	0,28	FAUX
FAUX	FAUX	VRAI	FAUX	VRAI	0,28	FAUX
FAUX	FAUX	VRAI	FAUX	VRAI	0,22	FAUX
FAUX	FAUX	VRAI	FAUX	VRAI	0,12	FAUX
FAUX	FAUX	VRAI	FAUX	VRAI	0,12	FAUX
FAUX	FAUX	VRAI	FAUX	VRAI	0,09	FAUX
FAUX	FAUX	VRAI	FAUX	VRAI	0,31	FAUX
FAUX	FAUX	VRAI	FAUX	VRAI	0,09	FAUX
FAUX	FAUX	VRAI	FAUX	VRAI	0,13	FAUX
FAUX	FAUX	VRAI	FAUX	VRAI	0,16	FAUX
FAUX	FAUX	VRAI	FAUX	VRAI	0,28	FAUX
FAUX	FAUX	VRAI	FAUX	VRAI	0,32	FAUX
FAUX	FAUX	VRAI	FAUX	VRAI	0,23	FAUX
FAUX	FAUX	VRAI	FAUX	VRAI	0,09	FAUX
FAUX	FAUX	FAUX	FAUX	VRAI	0,13	FAUX
FAUX	FAUX	VRAI	FAUX	VRAI	0,09	FAUX
FAUX	FAUX	FAUX	FAUX	VRAI	0,09	FAUX
FAUX	FAUX	FAUX	FAUX	VRAI	0,26	FAUX
FAUX	FAUX	VRAI	FAUX	VRAI	0,18	FAUX
FAUX	FAUX	VRAI	FAUX	VRAI	0,09	FAUX
FAUX	FAUX	VRAI	FAUX	VRAI	0,21	FAUX
FAUX	FAUX	VRAI	FAUX	VRAI	0,14	FAUX
FAUX	FAUX	VRAI	FAUX	VRAI	0,09	FAUX
FAUX	FAUX	VRAI	FAUX	VRAI	0,09	FAUX
FAUX	FAUX	VRAI	FAUX	VRAI	0,16	FAUX
FAUX	FAUX	VRAI	FAUX	VRAI	0,14	FAUX

FAUX	FAUX	VRAI	FAUX	VRAI	0,14	FAUX
FAUX	FAUX	VRAI	FAUX	FAUX	0	FAUX
FAUX	VRAI	VRAI	FAUX	VRAI	0,22	FAUX
FAUX	VRAI	VRAI	FAUX	VRAI	0,18	FAUX
FAUX	FAUX	FAUX	FAUX	VRAI	0,11	FAUX
FAUX	FAUX	VRAI	FAUX	VRAI	6,43	VRAI
FAUX	FAUX	VRAI	FAUX	VRAI	7,77	VRAI
FAUX	FAUX	VRAI	FAUX	VRAI	6,66	VRAI
FAUX	FAUX	VRAI	FAUX	VRAI	4,55	VRAI
FAUX	FAUX	VRAI	FAUX	VRAI	2	VRAI
FAUX	FAUX	VRAI	FAUX	VRAI	3,11	VRAI
FAUX	FAUX	VRAI	FAUX	VRAI	0,38	FAUX
FAUX	FAUX	VRAI	FAUX	VRAI	2,83	VRAI
FAUX	FAUX	VRAI	FAUX	VRAI	2,86	VRAI
FAUX	FAUX	VRAI	FAUX	VRAI	1,65	VRAI
FAUX	FAUX	VRAI	FAUX	FAUX	0	FAUX
FAUX	FAUX	VRAI	FAUX	VRAI	1,04	VRAI
FAUX	FAUX	VRAI	FAUX	VRAI	0,94	VRAI
FAUX	FAUX	VRAI	FAUX	VRAI	1	VRAI
FAUX	FAUX	FAUX	FAUX	VRAI	2,27	VRAI
FAUX	FAUX	FAUX	FAUX	VRAI	0,67	VRAI
FAUX	FAUX	FAUX	FAUX	VRAI	0,77	VRAI
FAUX	FAUX	FAUX	FAUX	VRAI	0,13	FAUX
FAUX	FAUX	VRAI	FAUX	VRAI	0,23	FAUX
FAUX	FAUX	FAUX	FAUX	VRAI	0,17	FAUX
FAUX	FAUX	VRAI	FAUX	VRAI	0,15	FAUX
FAUX	FAUX	VRAI	FAUX	VRAI	0,09	FAUX
FAUX	FAUX	VRAI	FAUX	VRAI	0,21	FAUX
FAUX	FAUX	VRAI	FAUX	VRAI	0,18	FAUX
FAUX	FAUX	FAUX	FAUX	VRAI	0,15	FAUX
FAUX	FAUX	FAUX	FAUX	VRAI	0,46	FAUX
FAUX	FAUX	FAUX	FAUX	VRAI	0,14	FAUX
FAUX	FAUX	FAUX	FAUX	VRAI	0,09	FAUX
FAUX	FAUX	VRAI	FAUX	VRAI	0,49	FAUX
FAUX	FAUX	FAUX	FAUX	VRAI	0,09	FAUX
FAUX	FAUX	FAUX	FAUX	VRAI	0,09	FAUX
FAUX	FAUX	VRAI	FAUX	VRAI	0,09	FAUX
FAUX	FAUX	VRAI	FAUX	VRAI	0,09	FAUX
FAUX	FAUX	VRAI	FAUX	VRAI	0,09	FAUX
FAUX	FAUX	VRAI	FAUX	VRAI	0,55	VRAI
FAUX	FAUX	VRAI	FAUX	VRAI	0,27	FAUX
FAUX	FAUX	VRAI	FAUX	VRAI	0,22	FAUX
FAUX	FAUX	VRAI	FAUX	VRAI	0,21	FAUX
FAUX	FAUX	VRAI	FAUX	VRAI	0,9	VRAI
FAUX	FAUX	FAUX	FAUX	VRAI	0,62	VRAI
FAUX	FAUX	VRAI	FAUX	VRAI	0,1	FAUX
FAUX	FAUX	VRAI	FAUX	VRAI	0,2	FAUX
FAUX	FAUX	VRAI	FAUX	VRAI	0,69	VRAI
FAUX	FAUX	FAUX	FAUX	VRAI	1,26	VRAI
FAUX	FAUX	FAUX	FAUX	VRAI	1,22	VRAI
FAUX	FAUX	VRAI	FAUX	VRAI	0,4	FAUX
FAUX	FAUX	VRAI	FAUX	VRAI	0,14	FAUX
FAUX	FAUX	FAUX	FAUX	VRAI	0,19	FAUX
FAUX	FAUX	VRAI	FAUX	VRAI	0,19	FAUX
FAUX	FAUX	VRAI	FAUX	VRAI	0,17	FAUX
FAUX	FAUX	VRAI	FAUX	VRAI	0,19	FAUX

FAUX	FAUX	VRAI	FAUX	FAUX	0	FAUX
FAUX	FAUX	VRAI	FAUX	VRAI	0,51	VRAI
FAUX	FAUX	VRAI	FAUX	VRAI	1,46	VRAI
FAUX	FAUX	VRAI	FAUX	VRAI	0,64	VRAI
FAUX	FAUX	VRAI	FAUX	VRAI	0,36	FAUX
FAUX	FAUX	VRAI	FAUX	VRAI	0,56	VRAI
FAUX	FAUX	FAUX	FAUX	VRAI	0,51	VRAI
FAUX	FAUX	FAUX	FAUX	VRAI	0,15	FAUX
FAUX	FAUX	VRAI	FAUX	VRAI	0,55	VRAI
FAUX	FAUX	FAUX	FAUX	VRAI	0,19	FAUX
FAUX	FAUX	FAUX	FAUX	VRAI	0,19	FAUX
FAUX	FAUX	FAUX	FAUX	VRAI	0,42	FAUX
FAUX	FAUX	FAUX	FAUX	VRAI	0,22	FAUX
FAUX	FAUX	FAUX	FAUX	VRAI	0,15	FAUX
FAUX	FAUX	FAUX	FAUX	VRAI	0,32	FAUX
FAUX	FAUX	FAUX	FAUX	VRAI	0	FAUX
FAUX	FAUX	FAUX	FAUX	VRAI	0,13	FAUX
FAUX	FAUX	FAUX	FAUX	VRAI	0,1	FAUX
FAUX	FAUX	FAUX	FAUX	VRAI	0,21	FAUX
FAUX	FAUX	VRAI	FAUX	VRAI	0,38	FAUX
FAUX	FAUX	VRAI	FAUX	VRAI	0,2	FAUX
FAUX	FAUX	VRAI	FAUX	VRAI	0,13	FAUX
FAUX	FAUX	VRAI	FAUX	VRAI	0,34	FAUX
VRAI	FAUX	VRAI	FAUX	VRAI	0,16	FAUX
VRAI	FAUX	VRAI	FAUX	VRAI	0,16	FAUX
FAUX	FAUX	VRAI	FAUX	VRAI	0,23	FAUX
FAUX	FAUX	VRAI	FAUX	VRAI	0,2	FAUX
FAUX	FAUX	FAUX	FAUX	VRAI	0,2	FAUX
FAUX	FAUX	FAUX	FAUX	VRAI	0,09	FAUX
FAUX	FAUX	FAUX	FAUX	VRAI	0,11	FAUX
FAUX	FAUX	FAUX	FAUX	VRAI	0,22	FAUX
FAUX	FAUX	VRAI	FAUX	VRAI	0,27	FAUX
FAUX	FAUX	FAUX	FAUX	VRAI	0,32	FAUX
FAUX	FAUX	FAUX	FAUX	VRAI	0,2	FAUX
FAUX	FAUX	FAUX	FAUX	VRAI	0,15	FAUX
FAUX	FAUX	FAUX	FAUX	VRAI	0,16	FAUX
FAUX	FAUX	FAUX	FAUX	VRAI	0,17	FAUX
FAUX	FAUX	VRAI	FAUX	VRAI	0,12	FAUX
FAUX	FAUX	VRAI	FAUX	VRAI	0,11	FAUX
FAUX	FAUX	VRAI	FAUX	VRAI	0,09	FAUX
FAUX	FAUX	VRAI	FAUX	VRAI	0,09	FAUX
FAUX	FAUX	FAUX	FAUX	FAUX	0	FAUX
FAUX	FAUX	FAUX	FAUX	VRAI	0,09	FAUX
FAUX	FAUX	VRAI	FAUX	FAUX	0	FAUX
FAUX	FAUX	VRAI	FAUX	VRAI	0,1	FAUX
FAUX	FAUX	VRAI	FAUX	VRAI	0,09	FAUX
FAUX	FAUX	VRAI	FAUX	VRAI	0,1	FAUX
FAUX	FAUX	VRAI	FAUX	VRAI	0,11	FAUX
FAUX	FAUX	VRAI	FAUX	VRAI	0,11	FAUX
FAUX	FAUX	VRAI	FAUX	VRAI	0,24	FAUX
FAUX	FAUX	VRAI	FAUX	VRAI	0,31	FAUX
FAUX	FAUX	FAUX	FAUX	VRAI	0,09	FAUX
FAUX	FAUX	FAUX	FAUX	VRAI	0,09	FAUX
FAUX	FAUX	FAUX	FAUX	VRAI	0,48	FAUX
FAUX	FAUX	FAUX	FAUX	VRAI	1,59	VRAI
FAUX	FAUX	VRAI	FAUX	VRAI	0,11	FAUX

FAUX	FAUX	VRAI	FAUX	VRAI	0,09	FAUX
FAUX	FAUX	VRAI	FAUX	VRAI	0,09	FAUX
FAUX	FAUX	FAUX	FAUX	VRAI	0,09	FAUX
FAUX	FAUX	FAUX	FAUX	VRAI	0,09	FAUX
FAUX	FAUX	VRAI	FAUX	VRAI	0,29	FAUX
FAUX	FAUX	VRAI	FAUX	VRAI	0,17	FAUX
FAUX	FAUX	VRAI	FAUX	VRAI	0,19	FAUX
FAUX	FAUX	FAUX	FAUX	VRAI	0,15	FAUX
FAUX	FAUX	FAUX	FAUX	VRAI	0,09	FAUX
FAUX	FAUX	VRAI	FAUX	VRAI	0,14	FAUX
FAUX	FAUX	FAUX	FAUX	VRAI	0,19	FAUX
FAUX	FAUX	FAUX	FAUX	VRAI	0,09	FAUX
FAUX	FAUX	VRAI	FAUX	VRAI	0,09	FAUX
FAUX	FAUX	FAUX	FAUX	VRAI	0,09	FAUX
FAUX	FAUX	FAUX	FAUX	VRAI	0,14	FAUX
FAUX	FAUX	VRAI	FAUX	VRAI	0,3	FAUX
FAUX	FAUX	VRAI	FAUX	VRAI	0,13	FAUX
FAUX	FAUX	VRAI	FAUX	FAUX	0	FAUX
FAUX	FAUX	VRAI	FAUX	VRAI	0,23	FAUX
FAUX	FAUX	VRAI	FAUX	VRAI	0,26	FAUX
FAUX	FAUX	VRAI	FAUX	VRAI	0,18	FAUX
FAUX	FAUX	VRAI	FAUX	VRAI	0,19	FAUX
FAUX	FAUX	VRAI	FAUX	VRAI	0,34	FAUX
FAUX	FAUX	VRAI	FAUX	VRAI	0,24	FAUX
FAUX	FAUX	FAUX	FAUX	VRAI	0,11	FAUX
FAUX	FAUX	FAUX	FAUX	VRAI	0,53	VRAI
FAUX	FAUX	VRAI	FAUX	VRAI	0,45	FAUX
FAUX	FAUX	VRAI	FAUX	VRAI	0,24	FAUX
FAUX	FAUX	VRAI	FAUX	VRAI	0,23	FAUX
FAUX	FAUX	FAUX	FAUX	VRAI	0,15	FAUX
FAUX	FAUX	FAUX	FAUX	VRAI	0,23	FAUX
FAUX	FAUX	FAUX	FAUX	VRAI	0,34	FAUX
FAUX	FAUX	FAUX	FAUX	VRAI	0,24	FAUX
FAUX	FAUX	FAUX	FAUX	VRAI	0,24	FAUX
FAUX	FAUX	FAUX	FAUX	VRAI	0,17	FAUX
FAUX	FAUX	FAUX	FAUX	VRAI	0,13	FAUX
FAUX	FAUX	FAUX	FAUX	VRAI	0,16	FAUX
FAUX	FAUX	FAUX	FAUX	VRAI	0,11	FAUX
FAUX	FAUX	FAUX	FAUX	VRAI	0,13	FAUX
FAUX	FAUX	FAUX	FAUX	VRAI	0,2	FAUX
FAUX	FAUX	FAUX	FAUX	VRAI	0,36	FAUX
FAUX	FAUX	FAUX	FAUX	VRAI	0,09	FAUX
FAUX	FAUX	FAUX	FAUX	VRAI	0,12	FAUX
FAUX	FAUX	FAUX	FAUX	VRAI	0,22	FAUX
FAUX	FAUX	FAUX	FAUX	VRAI	0,19	FAUX
FAUX	FAUX	FAUX	FAUX	FAUX	0	FAUX
FAUX	FAUX	FAUX	FAUX	FAUX	0	FAUX
FAUX	FAUX	FAUX	FAUX	VRAI	0,1	FAUX
FAUX	FAUX	FAUX	FAUX	VRAI	0,15	FAUX
FAUX	FAUX	FAUX	FAUX	VRAI	0,19	FAUX
FAUX	FAUX	FAUX	FAUX	VRAI	0,61	VRAI
FAUX	FAUX	FAUX	FAUX	VRAI	0,17	FAUX
FAUX	FAUX	FAUX	FAUX	VRAI	0,1	FAUX
FAUX	FAUX	FAUX	FAUX	FAUX	0	FAUX
FAUX	FAUX	FAUX	FAUX	VRAI	0	FAUX
FAUX	FAUX	FAUX	FAUX	FAUX	0	FAUX

FAUX	FAUX	FAUX	FAUX	VRAI	0,1	FAUX
FAUX	FAUX	FAUX	FAUX	VRAI	0,1	FAUX
FAUX	FAUX	FAUX	FAUX	VRAI	0	FAUX
FAUX	FAUX	VRAI	FAUX	VRAI	0,2	FAUX
FAUX	FAUX	VRAI	FAUX	VRAI	0,14	FAUX
FAUX	FAUX	VRAI	FAUX	VRAI	0,21	FAUX
FAUX	FAUX	VRAI	FAUX	VRAI	0,09	FAUX
FAUX	FAUX	VRAI	FAUX	VRAI	0,14	FAUX
FAUX	FAUX	VRAI	FAUX	FAUX	0	FAUX
FAUX	FAUX	VRAI	FAUX	VRAI	0,18	FAUX
FAUX	FAUX	VRAI	FAUX	VRAI	0,18	FAUX
FAUX	FAUX	VRAI	FAUX	VRAI	0,16	FAUX
FAUX	FAUX	VRAI	FAUX	VRAI	0,16	FAUX
FAUX	FAUX	VRAI	FAUX	VRAI	0,1	FAUX
FAUX	FAUX	VRAI	FAUX	VRAI	0,39	FAUX
FAUX	FAUX	VRAI	FAUX	VRAI	0,1	FAUX
FAUX	FAUX	VRAI	FAUX	VRAI	0,09	FAUX
FAUX	FAUX	VRAI	FAUX	VRAI	0,09	FAUX
FAUX	FAUX	VRAI	FAUX	FAUX	0	FAUX
FAUX	FAUX	VRAI	FAUX	VRAI	0,2	FAUX
FAUX	FAUX	VRAI	FAUX	VRAI	0,13	FAUX
FAUX	FAUX	VRAI	FAUX	VRAI	0,24	FAUX
FAUX	FAUX	VRAI	FAUX	VRAI	0,38	FAUX
FAUX	FAUX	VRAI	FAUX	VRAI	0,09	FAUX
FAUX	FAUX	VRAI	FAUX	VRAI	0,09	FAUX
FAUX	FAUX	VRAI	FAUX	VRAI	0,24	FAUX
FAUX	FAUX	FAUX	FAUX	VRAI	0,17	FAUX
FAUX	FAUX	VRAI	FAUX	VRAI	0,12	FAUX
FAUX	FAUX	VRAI	FAUX	VRAI	0,3	FAUX
FAUX	FAUX	VRAI	FAUX	VRAI	0,25	FAUX
FAUX	FAUX	VRAI	FAUX	VRAI	0,14	FAUX
FAUX	FAUX	VRAI	FAUX	VRAI	0,13	FAUX
FAUX	FAUX	FAUX	FAUX	VRAI	0,12	FAUX
FAUX	FAUX	FAUX	FAUX	VRAI	0,12	FAUX
FAUX	FAUX	FAUX	FAUX	VRAI	0,09	FAUX
VRAI	FAUX	FAUX	FAUX	VRAI	0,09	FAUX
FAUX	FAUX	FAUX	FAUX	VRAI	0,12	FAUX
FAUX	FAUX	FAUX	FAUX	VRAI	0,34	FAUX
FAUX	FAUX	FAUX	FAUX	VRAI	0,16	FAUX
FAUX	FAUX	FAUX	FAUX	VRAI	1,18	VRAI
FAUX	FAUX	VRAI	FAUX	VRAI	0,22	FAUX
FAUX	FAUX	VRAI	FAUX	VRAI	0,2	FAUX
FAUX	FAUX	FAUX	FAUX	VRAI	0,1	FAUX
FAUX	FAUX	VRAI	FAUX	VRAI	0,32	FAUX
FAUX	FAUX	FAUX	FAUX	VRAI	0,2	FAUX
FAUX	FAUX	VRAI	FAUX	VRAI	0,21	FAUX
FAUX	FAUX	VRAI	FAUX	VRAI	0,21	FAUX
FAUX	FAUX	FAUX	FAUX	VRAI	0,12	FAUX
FAUX	FAUX	FAUX	FAUX	VRAI	0,38	FAUX
FAUX	FAUX	FAUX	FAUX	VRAI	0,52	VRAI
FAUX	FAUX	FAUX	FAUX	VRAI	0,09	FAUX
FAUX	FAUX	FAUX	FAUX	VRAI	0,14	FAUX
FAUX	FAUX	FAUX	FAUX	VRAI	0,13	FAUX
FAUX	FAUX	FAUX	FAUX	VRAI	0,17	FAUX

colo	num_chamb	plasmair	porte_ouv	air_traité	asp_fumi_chamb	sct_nid_chamb
0-Pas de colonisation	5	VRAI	FAUX	VRAI	0	0
0-Pas de colonisation	6	VRAI	FAUX	VRAI	0	0
0-Pas de colonisation	9	VRAI	FAUX	VRAI	0	0
0-Pas de colonisation	26	FAUX	VRAI	FAUX	0	0
0-Pas de colonisation	26	FAUX	VRAI	FAUX	0	0
0-Pas de colonisation	26	FAUX	VRAI	FAUX	8	0
0-Pas de colonisation	26	FAUX	VRAI	FAUX	1	0
0-Pas de colonisation	26	FAUX	VRAI	FAUX	0	0
0-Pas de colonisation	26	FAUX	VRAI	FAUX	2	0
1-Colo probable	4	FAUX	VRAI	FAUX	0	0
0-Pas de colonisation	6	FAUX	VRAI	VRAI	0	0
0-Pas de colonisation	6	VRAI	FAUX	VRAI	0	0
0-Pas de colonisation	6	VRAI	FAUX	VRAI	0	0
0-Pas de colonisation	25	FAUX	VRAI	FAUX	1	0
0-Pas de colonisation	25	FAUX	VRAI	FAUX	0	0
0-Pas de colonisation	25	FAUX	VRAI	FAUX	0	2
0-Pas de colonisation	25	FAUX	VRAI	FAUX	1	0
0-Pas de colonisation	25	FAUX	VRAI	FAUX	0	0
0-Pas de colonisation	25	FAUX	VRAI	FAUX	0	0
0-Pas de colonisation	25	FAUX	VRAI	FAUX	0	2
0-Pas de colonisation	25	FAUX	VRAI	FAUX	0	0
0-Pas de colonisation	25	FAUX	FAUX	FAUX	0	1
0-Pas de colonisation	25	FAUX	VRAI	FAUX	0	0
0-Pas de colonisation	25	FAUX	FAUX	FAUX	0	1
0-Pas de colonisation	25	FAUX	VRAI	FAUX	0	0
0-Pas de colonisation	25	FAUX	VRAI	FAUX	0	0
0-Pas de colonisation	5	FAUX	VRAI	VRAI	0	0
0-Pas de colonisation	5	FAUX	VRAI	VRAI	0	0
0-Pas de colonisation	8	VRAI	VRAI	VRAI	0	0
0-Pas de colonisation	25	FAUX	VRAI	FAUX	0	0
0-Pas de colonisation	25	FAUX	VRAI	FAUX	0	0
0-Pas de colonisation	25	FAUX	VRAI	FAUX	0	1
0-Pas de colonisation	6	FAUX	VRAI	VRAI	0	1
1-Colo probable	6	FAUX	VRAI	VRAI	0	0
0-Pas de colonisation	6	FAUX	VRAI	VRAI	0	0
0-Pas de colonisation	5	VRAI	FAUX	VRAI	1	0
0-Pas de colonisation	23	FAUX	VRAI	FAUX	0	0
0-Pas de colonisation	23	FAUX	VRAI	FAUX	1	0
0-Pas de colonisation	23	FAUX	VRAI	FAUX	0	0
0-Pas de colonisation	23	FAUX	VRAI	FAUX	0	0
0-Pas de colonisation	23	FAUX	VRAI	FAUX	0	0
0-Pas de colonisation	2	VRAI	FAUX	VRAI	0	0
0-Pas de colonisation	1	FAUX	FAUX	FAUX	0	0
1-Colo probable	1	FAUX	VRAI	FAUX	0	0
0-Pas de colonisation	1	FAUX	VRAI	FAUX	0	0
0-Pas de colonisation	1	FAUX	VRAI	FAUX	0	0
0-Pas de colonisation	1	FAUX	VRAI	FAUX	1	0
0-Pas de colonisation	1	FAUX	VRAI	FAUX	0	0
0-Pas de colonisation	1	FAUX	VRAI	FAUX	0	0
0-Pas de colonisation	1	FAUX	VRAI	FAUX	0	0
0-Pas de colonisation	5	FAUX	VRAI	VRAI	0	0
0-Pas de colonisation	5	FAUX	VRAI	VRAI	1	0
0-Pas de colonisation	5	FAUX	VRAI	VRAI	0	0
0-Pas de colonisation	7	VRAI	VRAI	VRAI	0	0
0-Pas de colonisation	7	VRAI	VRAI	VRAI	0	0

0-Pas de colonisation	7	VRAI	FAUX	VRAI	0	0
0-Pas de colonisation	2	VRAI	FAUX	VRAI	2	0
0-Pas de colonisation	7	VRAI	VRAI	VRAI	0	0
0-Pas de colonisation	7	VRAI	VRAI	VRAI	1	0
0-Pas de colonisation	7	VRAI	VRAI	VRAI	0	0
0-Pas de colonisation	1	FAUX	VRAI	FAUX	0	0
0-Pas de colonisation	1	FAUX	VRAI	FAUX	2	3
0-Pas de colonisation	3	FAUX	VRAI	FAUX	1	0
0-Pas de colonisation	3	FAUX	VRAI	FAUX	0	0
0-Pas de colonisation	3	FAUX	VRAI	FAUX	0	0
0-Pas de colonisation	3	FAUX	VRAI	FAUX	1	0
0-Pas de colonisation	3	FAUX	VRAI	FAUX	0	1
0-Pas de colonisation	10	FAUX	VRAI	FAUX	0	0
0-Pas de colonisation	10	FAUX	VRAI	FAUX	0	0
0-Pas de colonisation	1	FAUX	VRAI	FAUX	0	0
0-Pas de colonisation	3	VRAI	FAUX	VRAI	0	0
0-Pas de colonisation	3	FAUX	FAUX	VRAI	0	0
0-Pas de colonisation	3	FAUX	VRAI	VRAI	0	0
0-Pas de colonisation	3	FAUX	VRAI	VRAI	3	1
0-Pas de colonisation	18	FAUX	FAUX	FAUX	0	0
0-Pas de colonisation	18	FAUX	FAUX	FAUX	0	0
0-Pas de colonisation	18	FAUX	VRAI	FAUX	4	0
0-Pas de colonisation	18	FAUX	VRAI	FAUX	0	0
0-Pas de colonisation	18	FAUX	VRAI	FAUX	0	0
0-Pas de colonisation	18	FAUX	VRAI	FAUX	0	0
0-Pas de colonisation	18	FAUX	VRAI	FAUX	0	1
0-Pas de colonisation	18	FAUX	VRAI	FAUX	0	0
0-Pas de colonisation	18	FAUX	VRAI	FAUX	0	15
0-Pas de colonisation	18	FAUX	VRAI	FAUX	0	0
0-Pas de colonisation	18	FAUX	VRAI	FAUX	0	0
0-Pas de colonisation	18	FAUX	VRAI	FAUX	0	0
0-Pas de colonisation	18	FAUX	VRAI	FAUX	0	0
0-Pas de colonisation	8	VRAI	VRAI	VRAI	0	0
0-Pas de colonisation	6	FAUX	VRAI	VRAI	0	0
0-Pas de colonisation	6	FAUX	VRAI	VRAI	0	0
0-Pas de colonisation	6	FAUX	VRAI	VRAI	0	0
0-Pas de colonisation						
0-Pas de colonisation	6	FAUX	VRAI	VRAI	0	0
0-Pas de colonisation	6	VRAI	FAUX	VRAI	0	0
0-Pas de colonisation	24	FAUX	VRAI	FAUX	0	0
0-Pas de colonisation	24	FAUX	VRAI	FAUX	0	21
0-Pas de colonisation	24	FAUX	VRAI	FAUX	0	0
0-Pas de colonisation	24	FAUX	VRAI	FAUX	0	0
0-Pas de colonisation	3	FAUX	VRAI	FAUX	0	0
0-Pas de colonisation	3	FAUX	VRAI	FAUX	0	0
0-Pas de colonisation	3	FAUX	VRAI	FAUX	0	0
0-Pas de colonisation	3	FAUX	VRAI	FAUX	0	0
0-Pas de colonisation	3	FAUX	VRAI	FAUX	1	0
0-Pas de colonisation	3	FAUX	VRAI	FAUX	0	0
0-Pas de colonisation	3	FAUX	VRAI	FAUX	0	0
0-Pas de colonisation	2	FAUX	VRAI	VRAI	0	1
0-Pas de colonisation	7	VRAI	FAUX	VRAI	0	0
0-Pas de colonisation	3	FAUX	VRAI	FAUX	2	1
0-Pas de colonisation	3	FAUX	VRAI	FAUX	1	0
0-Pas de colonisation	3	FAUX	VRAI	FAUX	0	0
0-Pas de colonisation	1	FAUX	VRAI	VRAI	0	0

0-Pas de colonisation	1	VRAI	FAUX	VRAI	0	0
0-Pas de colonisation	2	FAUX	VRAI	VRAI	0	0
0-Pas de colonisation	2	FAUX	VRAI	VRAI	0	0
0-Pas de colonisation	2	FAUX	VRAI	VRAI	0	0
0-Pas de colonisation	2	VRAI	FAUX	VRAI	0	0
0-Pas de colonisation	2	VRAI	FAUX	VRAI	0	0
0-Pas de colonisation	2	VRAI	FAUX	VRAI	0	0
0-Pas de colonisation	2	VRAI	FAUX	VRAI	0	0
0-Pas de colonisation	2	VRAI	FAUX	VRAI	3	0
0-Pas de colonisation	2	VRAI	FAUX	VRAI	0	0
0-Pas de colonisation	8	VRAI	FAUX	VRAI	0	0
0-Pas de colonisation	8	VRAI	FAUX	VRAI	0	0
0-Pas de colonisation	8	VRAI	FAUX	VRAI	0	0
0-Pas de colonisation	8	VRAI	FAUX	VRAI	0	0
0-Pas de colonisation	6	VRAI	FAUX	VRAI	1	0
0-Pas de colonisation	7	VRAI	FAUX	VRAI	0	0
0-Pas de colonisation	26	FAUX	FAUX	FAUX	0	0
0-Pas de colonisation	26	FAUX	VRAI	FAUX	0	0
0-Pas de colonisation	26	FAUX	VRAI	FAUX	0	0
0-Pas de colonisation	26	FAUX	VRAI	FAUX	2	0
0-Pas de colonisation	26	FAUX	VRAI	FAUX	1	0
0-Pas de colonisation	26	FAUX	VRAI	FAUX	0	0
0-Pas de colonisation	26	FAUX	VRAI	FAUX	10	0
0-Pas de colonisation	26	FAUX	VRAI	FAUX	0	0
0-Pas de colonisation	26	FAUX	VRAI	FAUX	0	0
0-Pas de colonisation	2	VRAI	FAUX	VRAI	0	0
0-Pas de colonisation	2	VRAI	FAUX	VRAI	0	0
0-Pas de colonisation	5	FAUX	VRAI	FAUX	0	0
0-Pas de colonisation	5	FAUX	VRAI	FAUX	0	0
0-Pas de colonisation	6	VRAI	FAUX	VRAI	0	0
0-Pas de colonisation	6	VRAI	FAUX	VRAI	0	0
0-Pas de colonisation	3	FAUX	VRAI	FAUX	0	3
0-Pas de colonisation	3	FAUX	VRAI	FAUX	0	0
0-Pas de colonisation	3	FAUX	VRAI	FAUX	0	0
0-Pas de colonisation	3	FAUX	VRAI	FAUX	0	2
0-Pas de colonisation						
0-Pas de colonisation	2	FAUX	VRAI	VRAI	0	0
0-Pas de colonisation	2	VRAI	FAUX	VRAI	0	1
0-Pas de colonisation	3	FAUX	VRAI	FAUX	0	0
0-Pas de colonisation	3	FAUX	VRAI	FAUX	0	1
0-Pas de colonisation	3	FAUX	VRAI	FAUX	0	1
0-Pas de colonisation	3	FAUX	VRAI	FAUX	0	0
0-Pas de colonisation	3	FAUX	VRAI	FAUX	0	0
0-Pas de colonisation	3	FAUX	VRAI	FAUX	0	1
0-Pas de colonisation	3	FAUX	VRAI	FAUX	0	1
0-Pas de colonisation	5	FAUX	FAUX	VRAI	0	0
0-Pas de colonisation	24	FAUX	VRAI	FAUX	0	0
0-Pas de colonisation	24	FAUX	VRAI	FAUX	2	0
0-Pas de colonisation	24	FAUX	VRAI	FAUX	0	0
0-Pas de colonisation	24	FAUX	VRAI	FAUX	0	0
0-Pas de colonisation						
0-Pas de colonisation	2	VRAI	FAUX	VRAI	0	0
0-Pas de colonisation	2	VRAI	FAUX	VRAI	0	0
0-Pas de colonisation	25	FAUX	VRAI	FAUX	0	1
0-Pas de colonisation	25	FAUX	VRAI	FAUX	0	0
0-Pas de colonisation	4	FAUX	VRAI	FAUX	0	0

1-Colo probable	4	FAUX	VRAI	FAUX	0	0
0-Pas de colonisation	4	FAUX	VRAI	FAUX	0	0
0-Pas de colonisation	4	FAUX	VRAI	FAUX	1	0
0-Pas de colonisation	4	FAUX	VRAI	FAUX	0	0
0-Pas de colonisation	4	FAUX	VRAI	FAUX	0	0
0-Pas de colonisation	3	VRAI	FAUX	VRAI	0	0
0-Pas de colonisation	6	FAUX	VRAI	FAUX	0	0
0-Pas de colonisation	6	FAUX	VRAI	FAUX	0	0
0-Pas de colonisation	6	FAUX	VRAI	FAUX	0	0
0-Pas de colonisation	6	FAUX	FAUX	FAUX	0	0
0-Pas de colonisation	6	FAUX	FAUX	FAUX	0	0
0-Pas de colonisation	3	FAUX	FAUX	FAUX	0	0
0-Pas de colonisation	3	FAUX	VRAI	FAUX	0	1
0-Pas de colonisation	3	FAUX	FAUX	FAUX	0	1
0-Pas de colonisation						
0-Pas de colonisation	3	FAUX	VRAI	VRAI	0	1
0-Pas de colonisation	3	FAUX	VRAI	VRAI	0	0
0-Pas de colonisation	2	FAUX	VRAI	FAUX	0	0
0-Pas de colonisation	2	FAUX	VRAI	FAUX	0	0
0-Pas de colonisation	2	FAUX	VRAI	FAUX	0	0
0-Pas de colonisation	2	FAUX	VRAI	FAUX	0	0
0-Pas de colonisation	2	FAUX	VRAI	FAUX	0	0
0-Pas de colonisation	2	FAUX	VRAI	FAUX	0	0
0-Pas de colonisation	2	FAUX	VRAI	FAUX	0	1
0-Pas de colonisation	3	FAUX	VRAI	VRAI		
0-Pas de colonisation	3	FAUX	VRAI	VRAI	0	0
0-Pas de colonisation	3	FAUX	VRAI	VRAI	0	0
0-Pas de colonisation	3	VRAI	FAUX	VRAI	0	0
0-Pas de colonisation	3	VRAI	FAUX	VRAI	0	0
0-Pas de colonisation	6	FAUX	VRAI	VRAI	0	0
0-Pas de colonisation	6	FAUX	VRAI	VRAI	0	0
0-Pas de colonisation	1	VRAI	FAUX	VRAI	0	0
0-Pas de colonisation	1	FAUX	VRAI	FAUX	0	0
0-Pas de colonisation	1	FAUX	VRAI	FAUX	0	1
0-Pas de colonisation	1	FAUX	VRAI	FAUX	0	0
0-Pas de colonisation	1	FAUX	VRAI	FAUX	0	0
0-Pas de colonisation	1	FAUX	VRAI	FAUX	0	0
0-Pas de colonisation	1	FAUX	FAUX	FAUX	0	1
0-Pas de colonisation	1	FAUX	VRAI	FAUX	2	0
0-Pas de colonisation	1	FAUX	VRAI	FAUX	0	0
0-Pas de colonisation	1	FAUX	VRAI	FAUX	0	0
0-Pas de colonisation	1	FAUX	VRAI	FAUX	0	0
0-Pas de colonisation	3	VRAI	FAUX	VRAI	0	0
0-Pas de colonisation	3	VRAI	FAUX	VRAI	0	0
0-Pas de colonisation	17	FAUX	VRAI	FAUX	0	0
0-Pas de colonisation	11	FAUX	VRAI	FAUX	0	0
0-Pas de colonisation	11	FAUX	VRAI	FAUX	0	0
0-Pas de colonisation	6	VRAI	FAUX	VRAI	0	0
0-Pas de colonisation	2	FAUX	VRAI	FAUX	4	0
0-Pas de colonisation	2	FAUX	VRAI	FAUX	0	0
0-Pas de colonisation	2	FAUX	VRAI	FAUX	1	0
0-Pas de colonisation	2	FAUX	VRAI	FAUX	0	0
0-Pas de colonisation	2	FAUX	VRAI	FAUX	0	0
0-Pas de colonisation	2	FAUX	VRAI	FAUX	0	0
0-Pas de colonisation						
0-Pas de colonisation	6	FAUX	VRAI	VRAI		

0-Pas de colonisation	6	FAUX	VRAI	VRAI	0	0
0-Pas de colonisation	6	FAUX	VRAI	VRAI	0	0
1-Colo probable	25	VRAI	FAUX	VRAI	0	0
2-Colo certaine	2	FAUX	VRAI	VRAI	1	0
0-Pas de colonisation	2	FAUX	VRAI	FAUX	0	0
0-Pas de colonisation	2	FAUX	FAUX	VRAI	0	0
0-Pas de colonisation	2	FAUX	VRAI	VRAI	0	0
0-Pas de colonisation	2	FAUX	VRAI	VRAI	0	0
0-Pas de colonisation	3	FAUX	FAUX	VRAI	0	0
0-Pas de colonisation	2	FAUX	FAUX	VRAI	0	0
0-Pas de colonisation	8	VRAI	FAUX	VRAI	0	0
0-Pas de colonisation	3	FAUX	VRAI	FAUX	0	0
0-Pas de colonisation	7	VRAI	FAUX	VRAI	0	0
0-Pas de colonisation	4	FAUX	VRAI	VRAI	0	0
0-Pas de colonisation		FAUX	VRAI	FAUX	0	0
0-Pas de colonisation	1	FAUX	VRAI	FAUX	0	0
0-Pas de colonisation	1	FAUX	VRAI	FAUX	1	0
0-Pas de colonisation	1	FAUX	VRAI	FAUX	0	0
0-Pas de colonisation	1	FAUX	VRAI	FAUX	0	0
0-Pas de colonisation	1	FAUX	VRAI	FAUX	0	0
0-Pas de colonisation	1	FAUX	VRAI	FAUX	0	1
0-Pas de colonisation	3	VRAI	FAUX	VRAI	0	0
0-Pas de colonisation	12	VRAI	FAUX	VRAI	0	0
0-Pas de colonisation	26	FAUX	VRAI	FAUX	0	1
0-Pas de colonisation	26	FAUX	VRAI	FAUX	0	0
0-Pas de colonisation						
0-Pas de colonisation	5	FAUX	VRAI	VRAI	3	0
0-Pas de colonisation	5	VRAI	FAUX	VRAI	0	0
0-Pas de colonisation	5	FAUX	VRAI	VRAI	0	0
0-Pas de colonisation	5	FAUX	VRAI	VRAI	0	0
0-Pas de colonisation	5	FAUX	VRAI	VRAI	0	0
0-Pas de colonisation	5	FAUX	VRAI	VRAI	0	0
0-Pas de colonisation	11	FAUX	VRAI	VRAI	0	0
0-Pas de colonisation	11	FAUX	VRAI	VRAI	0	0
0-Pas de colonisation	5	VRAI	FAUX	VRAI	0	0
0-Pas de colonisation	3	FAUX	VRAI	VRAI	0	0
0-Pas de colonisation	12	FAUX	VRAI	VRAI	0	1
0-Pas de colonisation	12	FAUX	VRAI	VRAI	0	0
0-Pas de colonisation	12	FAUX	VRAI	VRAI	0	0
0-Pas de colonisation	2	FAUX	VRAI	VRAI	2	0
0-Pas de colonisation	2	FAUX	VRAI	VRAI	0	0
0-Pas de colonisation	2	FAUX	VRAI	VRAI	0	0
0-Pas de colonisation	7	FAUX	FAUX	VRAI	1	0
0-Pas de colonisation	12	FAUX	VRAI	VRAI	0	0
0-Pas de colonisation	12	FAUX	VRAI	VRAI	0	0
0-Pas de colonisation	12	FAUX	FAUX	VRAI	0	0
0-Pas de colonisation	5	VRAI	FAUX	VRAI	0	0
0-Pas de colonisation	25	FAUX	VRAI	FAUX	0	0
1-Colo probable	25	FAUX	VRAI	FAUX	0	1
0-Pas de colonisation	25	FAUX	VRAI	FAUX	1	0
0-Pas de colonisation						
0-Pas de colonisation	5	FAUX	FAUX	VRAI	0	0
0-Pas de colonisation	5	VRAI	FAUX	VRAI	0	0
0-Pas de colonisation	5	FAUX	VRAI	VRAI	0	0
0-Pas de colonisation	5	FAUX	VRAI	VRAI	0	0
0-Pas de colonisation	5	FAUX	VRAI	VRAI	0	0

0-Pas de colonisation	3	FAUX	VRAI	VRAI	0	0
0-Pas de colonisation	2	FAUX	FAUX	VRAI	0	0
0-Pas de colonisation	2	VRAI	FAUX	VRAI	0	0
0-Pas de colonisation	2	VRAI	FAUX	VRAI	0	0
1-Colo probable	2	VRAI	FAUX	VRAI	0	0
0-Pas de colonisation	2	VRAI	FAUX	VRAI	0	0
0-Pas de colonisation	2	VRAI	FAUX	VRAI	0	0
0-Pas de colonisation	2	VRAI	FAUX	VRAI	0	0
0-Pas de colonisation	6	VRAI	FAUX	VRAI	0	0
0-Pas de colonisation	26	FAUX	VRAI	FAUX	0	2
0-Pas de colonisation	26	FAUX	VRAI	FAUX	0	0
0-Pas de colonisation	26	FAUX	VRAI	FAUX	0	2
0-Pas de colonisation	26	FAUX	VRAI	FAUX	0	0
0-Pas de colonisation	26	FAUX	VRAI	FAUX	0	0
0-Pas de colonisation	26	FAUX	VRAI	FAUX	0	0
0-Pas de colonisation	26	FAUX	VRAI	FAUX	0	0
0-Pas de colonisation	26	FAUX	VRAI	FAUX	0	0
0-Pas de colonisation	26	FAUX	VRAI	FAUX	0	0
0-Pas de colonisation	26	FAUX	VRAI	FAUX	0	0
0-Pas de colonisation	5	VRAI	FAUX	VRAI	0	0
0-Pas de colonisation	11	VRAI	FAUX	VRAI	0	0
0-Pas de colonisation	6	VRAI	FAUX	VRAI	0	0
0-Pas de colonisation	12	FAUX	VRAI	FAUX	0	14
1-Colo probable	12	FAUX	VRAI	FAUX	0	1
2-Colo certaine	12	FAUX	VRAI	FAUX	0	0
0-Pas de colonisation	12	FAUX	VRAI	FAUX	0	0
0-Pas de colonisation	12	FAUX	VRAI	VRAI	0	0
0-Pas de colonisation	3	FAUX	FAUX	VRAI	0	0
1-Colo probable	5	VRAI	FAUX	VRAI	0	0
0-Pas de colonisation	5	VRAI	FAUX	VRAI	0	0
0-Pas de colonisation	5	VRAI	FAUX	VRAI	0	0
0-Pas de colonisation	1	FAUX	FAUX	FAUX	0	0
0-Pas de colonisation						
0-Pas de colonisation						
0-Pas de colonisation						
0-Pas de colonisation						
0-Pas de colonisation	21	FAUX	FAUX	FAUX	0	0
0-Pas de colonisation	5	FAUX	VRAI	VRAI	0	0
0-Pas de colonisation	5	VRAI	FAUX	VRAI	0	0
0-Pas de colonisation	12	VRAI	FAUX	VRAI	0	0
0-Pas de colonisation	12	VRAI	FAUX	VRAI	0	0
0-Pas de colonisation	7	FAUX	VRAI	FAUX	0	0
0-Pas de colonisation	7	FAUX	VRAI	FAUX	0	0
0-Pas de colonisation	1	FAUX	FAUX	VRAI	0	0
0-Pas de colonisation	4	FAUX	VRAI	VRAI	0	0
0-Pas de colonisation	4	VRAI	FAUX	VRAI	0	0
0-Pas de colonisation	4	FAUX	VRAI	VRAI	0	1
0-Pas de colonisation	5	VRAI	FAUX	VRAI	0	0
0-Pas de colonisation	5	FAUX	FAUX	VRAI	1	0
0-Pas de colonisation	5	FAUX	VRAI	VRAI	0	0
0-Pas de colonisation	5	VRAI	FAUX	VRAI	0	0
0-Pas de colonisation	5	VRAI	FAUX	VRAI	0	0
0-Pas de colonisation	7	VRAI	VRAI	VRAI	0	0
0-Pas de colonisation	10	FAUX	VRAI	VRAI	0	0
0-Pas de colonisation	5	VRAI	FAUX	VRAI	0	0
0-Pas de colonisation	13	FAUX	VRAI	FAUX	0	0

0-Pas de colonisation	13	FAUX	VRAI	FAUX	0	0
0-Pas de colonisation	13	FAUX	VRAI	FAUX	0	0
0-Pas de colonisation	13	FAUX	VRAI	FAUX	0	0
0-Pas de colonisation	13	FAUX	VRAI	FAUX	0	2
0-Pas de colonisation	6	VRAI	FAUX	VRAI	0	0
0-Pas de colonisation	6	VRAI	FAUX	VRAI	1	0
0-Pas de colonisation	3	FAUX	VRAI	FAUX	0	0
0-Pas de colonisation	3	FAUX	VRAI	FAUX	0	0
0-Pas de colonisation	3	FAUX	VRAI	FAUX	1	0
0-Pas de colonisation	3	FAUX	VRAI	FAUX	0	0
0-Pas de colonisation	2	FAUX	VRAI	FAUX	1	13
0-Pas de colonisation	3	FAUX	VRAI	FAUX	0	0
0-Pas de colonisation	3	FAUX	VRAI	FAUX	0	0
0-Pas de colonisation	3	FAUX	VRAI	FAUX	0	0
0-Pas de colonisation	3	FAUX	VRAI	FAUX	0	1
0-Pas de colonisation	3	FAUX	VRAI	FAUX	0	0
0-Pas de colonisation	3	VRAI	FAUX	VRAI	0	0
0-Pas de colonisation	12	VRAI	FAUX	VRAI	0	0
0-Pas de colonisation	12	VRAI	VRAI	VRAI	0	0
0-Pas de colonisation	12	FAUX	FAUX	VRAI	0	0
0-Pas de colonisation	16	FAUX	VRAI	FAUX	0	9
0-Pas de colonisation	16	FAUX	VRAI	FAUX	0	0
0-Pas de colonisation	16	FAUX	VRAI	FAUX	0	0
0-Pas de colonisation	16	FAUX	VRAI	FAUX	0	0
0-Pas de colonisation	16	FAUX	VRAI	FAUX	0	0
0-Pas de colonisation	16	FAUX	VRAI	FAUX	1	4
0-Pas de colonisation	16	FAUX	VRAI	FAUX	0	0
0-Pas de colonisation	16	FAUX	VRAI	FAUX	0	1
0-Pas de colonisation						
0-Pas de colonisation	8	VRAI	FAUX	VRAI	0	0
0-Pas de colonisation	8	VRAI	FAUX	VRAI	0	0
0-Pas de colonisation	5	FAUX	VRAI	FAUX	0	1
0-Pas de colonisation	5	FAUX	VRAI	FAUX	0	0
0-Pas de colonisation	9	FAUX	VRAI	VRAI	0	0
0-Pas de colonisation	9	VRAI	FAUX	VRAI	0	0
0-Pas de colonisation	6	VRAI	FAUX	VRAI	0	0
0-Pas de colonisation	9	FAUX	FAUX	VRAI	0	2
0-Pas de colonisation	7	VRAI	FAUX	VRAI	0	0
0-Pas de colonisation	2	FAUX	VRAI	FAUX	0	0
0-Pas de colonisation	2	FAUX	VRAI	FAUX	0	0
0-Pas de colonisation	2	VRAI	FAUX	VRAI	0	0
1-Colo probable	20	FAUX	VRAI	FAUX	0	0
0-Pas de colonisation	20	FAUX	VRAI	FAUX	1	0
0-Pas de colonisation	20	FAUX	VRAI	FAUX	0	1
0-Pas de colonisation	20	FAUX	VRAI	FAUX	0	0
0-Pas de colonisation	12	FAUX	VRAI	VRAI	0	0
0-Pas de colonisation	12	VRAI	FAUX	VRAI	0	0
0-Pas de colonisation	12	VRAI	FAUX	VRAI	0	0
0-Pas de colonisation	12	VRAI	FAUX	VRAI	0	0
0-Pas de colonisation	7	VRAI	FAUX	VRAI	0	0
0-Pas de colonisation	22	FAUX	VRAI	FAUX	0	0
0-Pas de colonisation	26	FAUX	VRAI	FAUX	0	0
0-Pas de colonisation	26	FAUX	FAUX	FAUX	0	0
0-Pas de colonisation	26	FAUX	FAUX	FAUX	0	0
0-Pas de colonisation	26	FAUX	VRAI	FAUX	0	0
0-Pas de colonisation	26	FAUX	FAUX	FAUX	0	0

0-Pas de colonisation	26	FAUX	FAUX	FAUX	0	0
0-Pas de colonisation	26	FAUX	FAUX	FAUX	1	2
0-Pas de colonisation						
0-Pas de colonisation	6	FAUX	VRAI	VRAI	0	0
0-Pas de colonisation	6	FAUX	VRAI	VRAI	0	0
0-Pas de colonisation	6	FAUX	VRAI	VRAI	0	0
0-Pas de colonisation	6	FAUX	VRAI	VRAI	0	0
0-Pas de colonisation	6	FAUX	VRAI	VRAI	0	1
0-Pas de colonisation	6	FAUX	VRAI	VRAI	0	0
0-Pas de colonisation	1	FAUX	VRAI	VRAI	0	0
0-Pas de colonisation	1	FAUX	VRAI	VRAI	0	0
0-Pas de colonisation	1	VRAI	FAUX	VRAI	0	0
0-Pas de colonisation	1	FAUX	VRAI	VRAI	0	0
0-Pas de colonisation	1	VRAI	FAUX	VRAI	0	0
0-Pas de colonisation	11	VRAI	FAUX	VRAI	0	0
0-Pas de colonisation	11	VRAI	FAUX	VRAI	0	0
0-Pas de colonisation	11	VRAI	VRAI	VRAI	0	0
0-Pas de colonisation	11	VRAI	FAUX	VRAI	0	0
0-Pas de colonisation	11	VRAI	FAUX	VRAI	0	0
0-Pas de colonisation	5	VRAI	FAUX	VRAI	0	0
0-Pas de colonisation	11	VRAI	FAUX	VRAI	0	0
0-Pas de colonisation	11	FAUX	VRAI	VRAI	0	0
0-Pas de colonisation	11	FAUX	VRAI	VRAI	0	0
0-Pas de colonisation	11	FAUX	FAUX	VRAI	0	0
0-Pas de colonisation	11	FAUX	FAUX	VRAI	0	0
0-Pas de colonisation	11	FAUX	VRAI	VRAI	0	0
0-Pas de colonisation	11	FAUX	VRAI	VRAI	0	0
0-Pas de colonisation	11	FAUX	VRAI	VRAI	0	0
0-Pas de colonisation	5	FAUX	FAUX	VRAI	0	0
0-Pas de colonisation	6	VRAI	FAUX	VRAI	1	0
0-Pas de colonisation	1	VRAI	FAUX	VRAI	0	0
0-Pas de colonisation	1	VRAI	FAUX	VRAI	0	0
0-Pas de colonisation	5	FAUX	FAUX	VRAI	0	0
0-Pas de colonisation	5	FAUX	FAUX	VRAI	0	0
0-Pas de colonisation	22	FAUX	VRAI	FAUX	0	0
1-Colo probable	22	FAUX	VRAI	FAUX	0	0
0-Pas de colonisation	22	FAUX	VRAI	FAUX	4	0
0-Pas de colonisation	22	FAUX	VRAI	FAUX	0	0
0-Pas de colonisation	22	FAUX	VRAI	FAUX	0	0
0-Pas de colonisation	22	FAUX	VRAI	FAUX	5	0
0-Pas de colonisation	2	VRAI	VRAI	VRAI	0	0
0-Pas de colonisation	24	FAUX	VRAI	FAUX	0	0
0-Pas de colonisation	24	FAUX	VRAI	FAUX	0	0
0-Pas de colonisation	24	FAUX	VRAI	FAUX	0	0
1-Colo probable	24	FAUX	VRAI	FAUX	0	0
0-Pas de colonisation	24	FAUX	VRAI	FAUX	0	0
0-Pas de colonisation						
0-Pas de colonisation	24	FAUX	VRAI	FAUX	0	1
0-Pas de colonisation	24	FAUX	VRAI	FAUX	0	0
0-Pas de colonisation	24	FAUX	VRAI	FAUX	1	0
0-Pas de colonisation	9	FAUX	VRAI	VRAI	0	0
0-Pas de colonisation	24	FAUX	VRAI	FAUX	0	0
0-Pas de colonisation	24	FAUX	VRAI	FAUX	0	0
0-Pas de colonisation	24	FAUX	VRAI	FAUX	0	0

asp_nid_chamb	asp_ustus_chamb	asp_niger_chamb	asp_sp_chamb	asp_terreus_chamb
0	0	0	0	0
0	0	0	0	0
0	0	0	0	0
0	0	0	0	0
0	0	0	0	0
0	0	0	0	0
0	0	0	0	0
0	0	0	0	0
0	0	0	0	0
0	1	0	16	0
0	0	0	0	0
0	0	0	0	0
0	0	0	0	0
0	0	0	0	0
0	0	0	0	0
0	0	0	0	0
1	1	0	0	0
0	0	0	0	0
0	0	0	0	0
0	0	0	0	0
0	0	0	0	0
0	0	0	0	0
0	0	0	0	0
0	0	0	0	0
0	0	0	0	0
0	0	0	0	0
0	0	0	0	0
0	0	0	0	0
0	0	0	0	0
0	0	0	1	0
0	0	0	0	0
0	0	0	0	0
0	0	0	0	0
0	0	0	0	0
0	0	0	0	0
0	0	0	0	0
0	0	0	2	0
0	0	0	0	0
0	0	0	0	0
0	0	0	0	0
0	0	0	0	0
0	0	0	0	0
0	0	0	0	0
0	0	0	1	0
0	0	0	0	0
0	0	0	3	0
0	0	0	0	0
0	0	0	0	0
0	0	0	0	0
0	0	0	0	0
0	0	0	0	0
0	0	0	0	0
0	0	0	0	0
0	0	0	0	0
0	0	0	0	0

0	0	0	0	0
0	0	0	0	0
0	0	0	0	0
0	0	0	0	0
0	0	0	0	0
0	0	0	0	0
0	0	0	0	0
0	0	1	1	0
0	0	0	4	0
0	0	0	3	0
0	0	0	0	0
0	0	0	1	0
0	0	0	0	0
0	0	0	0	0
0	1	0	0	0
0	0	0	0	0
0	0	0	0	0
0	0	0	0	0
0	0	1	0	0
0	0	0	0	0
0	0	0	0	0
0	0	0	0	0
0	0	0	0	0
0	0	0	0	0
0	0	0	0	0
0	0	0	0	0
0	0	0	0	0
0	0	0	0	0
0	0	0	0	0
0	0	0	0	0
0	0	0	0	0
0	0	0	0	0
0	0	0	0	0
0	0	0	0	0
0	0	0	0	0
0	0	0	0	0

0	0	0	0	0
0	0	0	0	0
0	0	0	0	0
0	0	0	2	0
0	0	0	3	0
0	0	0	0	0
0	0	0	0	0
0	0	0	1	0
0	0	0	0	0
0	0	0	0	0
0	0	0	0	0
0	0	0	2	0
0	0	0	0	0
0	0	0	1	0
0	0	0	1	0
0	0	0	0	0
0	0	0	1	0
0	0	0	0	0
0	0	0	0	0

0	0	0	0	0
0	0	0	0	0
0	0	0	0	0
0	0	0	0	0
0	0	0	0	0
0	0	0	0	0
0	0	0	0	0
0	0	0	0	0
0	0	0	1	0
0	0	0	0	0
0	0	0	0	0
0	0	0	0	0
0	0	0	0	0
0	0	0	0	0
0	0	0	0	0
0	0	0	0	0
0	0	0	0	0
0	0	0	0	0
0	0	0	0	0
0	0	0	0	0
0	0	0	0	0
0	0	0	0	0
0	0	0	0	0
0	0	0	0	0
0	0	0	0	0
0	0	0	0	0
0	0	0	0	0
0	0	0	0	0
0	0	0	0	0
0	0	0	0	0
0	0	0	0	0
0	0	0	0	0
0	0	0	0	0
0	0	1	0	0
0	0	0	0	0
0	0	0	0	0
0	0	0	0	0
0	0	0	0	0
0	0	0	0	0
0	0	0	0	0
0	0	0	0	0
0	0	0	0	0
0	0	0	0	0
0	0	0	0	0
0	0	0	0	0
0	0	0	0	0
0	0	0	2	0
0	0	0	0	0
0	0	0	0	0
0	0	0	0	0
0	0	0	0	0
0	0	0	0	0
0	0	0	0	0
0	0	1	0	0

0	0	0	0	0
5	0	0	0	0
0	0	0	0	0
0	0	0	0	0
0	0	0	0	0
0	0	0	0	0
0	0	0	0	0
0	0	0	1	0
0	0	0	0	0
0	0	0	0	0
0	0	0	0	0
0	0	0	0	0
0	0	0	0	0
0	0	0	0	0

0	0	0	0	0
0	0	0	0	0
0	0	0	0	0
0	0	0	0	0
0	0	0	0	0
0	0	0	3	0
0	0	0	1	0
0	0	0	2	0
0	0	0	0	0

0	0	0	0	0
0	0	0	0	0
0	0	0	0	0
0	0	0	0	0
0	0	0	0	0
0	0	0	0	0
0	0	0	0	0
0	0	0	0	0
0	0	0	0	0
0	0	0	0	0
1	0	0	0	0
0	0	0	1	0
0	0	0	0	0
0	0	0	0	0
1	0	0	0	0
0	0	3	0	0
0	0	0	0	0
0	0	0	0	0
0	0	0	0	0
0	0	0	1	0
0	0	0	1	0
0	0	0	0	0
0	0	0	0	0
0	0	0	0	0
0	0	0	0	0
0	0	0	0	0
0	0	0	0	0
0	0	0	0	0
0	0	0	0	0

0	0	0	0	0
0	0	0	0	0
0	0	0	0	0
0	0	0	5	0
0	0	0	0	0
0	0	0	0	0
0	0	0	0	0
0	0	0	0	0
0	0	0	2	0
0	0	0	0	0
0	0	0	0	0
0	0	0	1	0
0	0	0	0	0
0	0	0	0	0
0	0	0	0	0
0	0	0	0	0
0	0	0	0	0
0	0	0	0	0
0	0	0	0	0
0	0	0	0	0
0	0	0	0	0
0	0	0	0	0
0	0	0	0	0
0	0	0	0	0
0	0	0	0	0

0	0	0	0	0
0	0	0	0	0
0	0	0	0	0
0	0	0	0	0
0	0	0	0	0
0	0	0	0	0
0	0	0	0	0
0	0	0	0	0
0	0	0	0	0
0	0	0	0	0
0	0	0	0	0
0	0	0	0	0
0	0	0	0	0
0	0	1	0	0
0	0	0	0	0
0	0	0	0	0
0	0	0	1	0
0	0	0	0	0
0	0	0	0	0
0	0	0	0	0
0	0	0	0	0
0	0	0	0	0
0	0	0	4	0
0	0	0	0	0

0	0	0	0	0
0	0	0	0	0
0	0	0	0	0
0	0	0	0	0
0	0	0	0	0

0	0	0	0	0
0	0	0	0	0
0	0	0	0	0
0	0	0	0	0
0	0	1	0	0
0	0	0	0	0
0	0	0	0	0
0	0	0	0	0
0	0	0	0	0
0	0	0	0	0
0	0	0	0	0
0	0	0	0	0
0	0	0	0	0
0	0	0	0	0
0	0	0	1	0
0	0	0	0	0
0	0	0	0	0
0	0	0	0	0
0	0	0	0	0
0	0	0	0	0
0	0	0	0	0
0	0	0	0	0
0	0	0	0	0
0	0	0	0	0
0	0	0	0	0
0	0	0	0	0
0	1	0	0	0
0	0	0	0	0
0	0	0	0	0
0	0	0	0	0
0	0	0	0	0
0	0	0	0	0

0	0	0	0	0
0	0	1	0	0
0	0	0	0	0
0	0	0	0	0
0	0	0	0	0
0	0	0	0	0
0	0	0	0	0
0	0	0	0	0
0	0	0	0	0
0	0	0	0	0
0	0	0	0	0
0	0	0	0	0
0	0	0	0	0
0	0	0	0	0
0	0	0	0	0
0	0	0	0	0
0	0	0	0	0
0	0	0	0	0
0	0	0	0	0
0	0	0	0	0

0	0	0	0	0
0	0	0	0	0
0	0	0	0	0
0	0	0	0	0
0	0	0	1	0
0	0	0	1	0
0	0	0	0	0
0	0	0	0	0
0	0	0	2	0
0	0	0	9	0
0	0	0	10	0
0	0	0	0	0
0	0	0	0	0
0	0	0	0	0
0	0	0	0	0
0	0	0	0	0
0	0	0	0	0
0	0	0	0	1
0	0	0	0	0
0	0	0	0	0
0	0	0	0	0
0	0	0	0	0
0	0	0	0	0
0	0	0	0	0
2	0	0	0	0
0	0	0	0	0
0	0	0	0	0
0	0	0	0	0

0	0	0	0	0
0	0	0	0	0
0	0	0	0	0
0	0	0	0	0
0	1	0	0	0
0	0	0	0	0
0	0	0	0	0
0	0	0	0	0
0	0	0	0	0
0	0	0	0	0
0	0	0	0	0
0	0	0	0	0
0	0	0	1	0
0	0	0	0	0
0	0	0	0	0
0	0	0	1	0
0	0	0	0	0
0	0	0	0	0
0	0	0	0	0
0	0	0	0	0
0	0	0	0	0
0	0	0	0	0
0	0	0	0	0
0	0	0	0	0
0	0	0	0	0
0	0	0	1	0
0	0	0	0	0

0	0	0	0	0
0	0	0	1	0
0	0	0	0	0
0	0	0	0	0
0	0	0	0	0
0	0	0	0	0
0	1	0	0	0
0	0	0	0	0
0	0	0	0	0
0	0	0	0	0
0	0	0	0	0
0	0	0	0	0
0	0	0	0	0
0	0	0	0	0
0	0	0	0	0
0	0	0	0	0
0	0	0	0	0
0	0	0	0	0
0	0	0	0	0
0	0	0	0	0
0	0	0	0	0
0	0	0	0	0
0	0	0	0	0
0	0	0	0	0
0	0	0	0	0
0	0	0	0	0
0	0	0	0	0
0	0	0	0	0
0	0	0	0	0
0	0	0	0	0
0	0	0	0	0
0	0	0	0	0
0	0	0	0	0
0	0	0	0	0
0	0	3	0	0
0	0	0	0	0
0	1	0	0	0
0	0	0	1	0
0	0	0	0	0
0	0	0	1	0
0	0	0	0	0
0	0	1	0	0
0	0	0	0	0
0	0	0	0	0
0	0	0	0	0
0	0	0	0	0
0	0	0	0	0
0	0	0	0	0
0	0	0	1	0
0	0	0	0	0
0	0	0	0	0

asp_flavus_chamb	peni_chamb	clado_chamb	alterna_chamb	ulocla_chamb	chaeto_chamb
0	0	0	0	0	0
0	1	0	0	0	0
0	0	0	0	0	0
0	0	0	0	0	0
0	0	0	0	0	0
0	2	0	0	0	0
0	0	0	0	0	0
0	2	0	0	0	0
0	4	0	0	0	0
0	3	0	0	0	0
0	1	3	0	0	0
0	0	0	0	0	0
0	0	1	0	0	0
0	0	2	1	0	0
0	4	0	0	0	0
0	12	5	0	0	0
0	31	2	0	0	0
0	15	1	0	0	0
0	66	3	0	0	0
0	4	0	0	0	0
0	2	0	0	0	0
0	0	0	0	0	1
0	0	2	0	0	0
0	0	1	0	0	0
0	4	0	1	0	0
0	4	0	0	0	0
0	0	0	0	0	0
0	0	0	0	0	0
0	1	0	0	0	0
0	14	0	0	0	0
0	2	0	0	0	0
0	0	1	0	0	0
0	0	0	0	0	0
0	0	0	0	0	0
0	1	0	0	0	0
0	0	0	0	0	0
0	6	0	0	0	0
0	0	0	0	0	0
0	0	0	0	0	0
0	0	0	0	0	0
0	5	0	0	0	0
0	0	1	0	0	0
0	1	4	1	0	0
0	0	0	0	0	0
0	0	0	0	0	0
0	1	3	1	0	0
0	1	0	1	0	0
0	15	1	0	0	0
0	25	0	0	0	0
0	1	1	0	0	0
0	0	1	1	0	0
0	1	0	1	0	0
0	0	0	0	0	0
0	2	0	0	0	0
0	0	0	0	0	0

0	0	0	0	0	0
0	0	0	0	0	0
0	1	0	0	0	0
0	0	0	0	0	0
0	0	2	1	0	0
0	0	0	0	0	0
0	0	2	1	0	0
0	1	0	0	0	0
0	1	0	1	0	0
0	0	0	0	0	0
0	0	3	0	0	0
0	0	0	0	0	0
0	2	0	0	0	0
0	1	0	0	0	0
0	5	0	0	0	0
0	1	0	0	0	0
0	0	0	0	0	0
0	0	0	0	0	0
0	0	0	0	0	0
0	0	1	0	0	0
0	0	0	0	0	0
0	0	1	0	0	0
0	0	0	0	0	0
0	0	0	0	0	0
0	0	0	0	0	0
0	0	0	1	0	0
0	0	2	0	0	0
0	0	0	0	0	0
0	0	2	0	0	0
0	1	0	0	0	0
0	0	1	0	0	0
0	1	1	0	0	0
0	0	0	0	0	0
0	0	0	0	0	0
0	0	0	0	0	0
0	0	0	0	0	0

0	0	0	0	0	0
0	0	0	0	0	0
0	1	2	1	0	0
0	0	3	0	0	0
0	2	3	1	0	0
0	0	0	0	0	0
0	1	3	0	0	0
0	2	1	0	0	0
0	8	0	0	0	0
0	14	3	0	0	0
0	3	0	0	0	0
0	0	0	0	0	0
0	50	3	2	0	0
0	1	1	0	0	0
0	0	1	0	0	0
0	2	1	0	0	0
0	0	0	0	0	0
0	0	0	0	0	0
0	0	0	0	0	0

0	0	0	0	0	0
0	0	0	0	0	0
0	0	0	0	0	0
0	0	0	0	0	0
0	0	0	0	0	0
0	0	0	0	0	0
0	0	0	0	0	0
0	0	0	0	0	0
0	1	0	0	0	0
0	0	0	0	0	0
0	0	0	0	0	0
0	0	0	0	0	0
0	0	0	0	0	0
0	0	0	0	0	0
0	0	0	0	0	0
0	0	0	0	0	0
0	16	0	0	0	0
0	5	0	0	0	0
0	0	0	0	0	0
0	0	0	0	0	0
0	0	0	0	0	0
0	1	0	0	0	0
0	0	0	0	0	0
0	0	0	0	0	0
0	2	0	0	0	0
0	0	0	0	0	0
0	0	0	0	0	0
0	1	0	0	0	0
0	10	3	0	0	0
0	0	1	0	0	0
0	0	1	0	0	0
0	0	0	0	0	0
0	1	0	0	0	0
0	1	0	0	0	0
0	2	1	1	0	0
0	0	0	0	0	0
0	0	0	0	0	0
0	0	0	0	0	0
0	0	1	0	0	0
0	0	4	1	0	0
0	0	2	0	0	0
0	0	1	1	0	0
0	23	0	0	0	0
0	0	0	0	0	0
0	2	0	0	0	0
0	0	0	0	0	0
0	0	0	0	0	0
0	0	0	0	0	0
0	1	0	0	0	0
0	0	0	0	0	0
0	0	0	0	0	0
0	0	0	0	0	0
0	0	0	0	0	0
0	0	0	0	0	0

0	1	2	1	0	0
0	0	0	0	0	0
0	0	2	0	0	0
0	0	0	0	0	0
0	1	0	2	0	0
0	0	4	0	0	0
0	1	0	1	0	0
0	0	0	0	0	0
0	1	1	0	0	0
0	0	0	0	0	0
0	6	2	0	0	0
0	1	1	0	0	0
0	0	0	0	0	0
0	0	1	1	0	0
0	1	0	0	0	0
0	0	0	0	0	0
0	1	3	0	0	0
0	1	4	1	0	0
0	0	10	0	0	0
0	2	2	1	0	0
0	1	3	0	0	0
0	1	3	1	0	0
0	0	0	0	0	0
0	0	0	0	0	0
0	0	1	0	0	0
0	0	0	0	0	0
0	0	2	0	0	0
0	3	0	0	0	0
0	0	1	0	0	0
0	0	0	0	0	0
0	18	1	0	0	0
0	0	0	1	0	0
0	1	3	0	0	0
0	0	0	0	0	0
0	0	8	0	0	0
0	2	0	0	0	0
0	0	0	0	0	0
0	1	0	0	0	0
0	0	0	0	0	0
0	3	0	0	0	0
0	0	0	0	0	0
0	1	0	0	0	0
0	2	0	0	0	0
0	1	0	0	0	0
0	0	4	0	0	0
0	0	0	0	0	0
0	0	0	0	0	0
0	0	1	0	0	0
0	0	0	0	0	0
0	0	1	0	0	0
0	0	0	0	0	0
0	1	1	0	0	0

0	0	0	0	0	0
0	0	0	0	0	0
0	0	0	0	0	0
0	2	0	0	0	0
0	8	0	0	0	0
0	5	0	0	0	0
0	0	0	0	0	0
0	0	0	0	0	0
0	10	0	0	0	0
0	0	0	0	0	0
0	0	0	0	0	0
0	2	0	0	0	0
0	0	0	0	0	0
0	0	0	0	0	0
0	0	0	0	0	0
0	0	0	0	0	0
0	0	2	0	0	0
0	0	0	0	0	0
0	0	1	1	0	0
0	6	7	1	0	0
0	1	0	0	0	0
0	0	1	0	0	0
0	0	0	0	0	0
0	2	0	0	0	0
0	1	0	0	0	0
0	0	0	0	0	0
0	0	0	0	0	0
0	0	0	0	0	0
0	0	0	0	0	0
0	0	0	0	0	0
0	0	0	0	0	0
0	0	0	0	0	0
0	0	2	0	0	0
0	0	0	0	0	0
0	0	0	0	0	0
0	0	0	0	0	0
0	0	0	1	0	0
0	1	0	0	0	0
0	0	0	0	0	0
0	0	0	0	0	0
0	0	0	0	0	0
0	0	0	0	0	0
0	0	0	0	0	0
0	0	0	0	0	0
0	1	0	0	0	0
0	0	0	0	0	0
0	2	1	0	0	0
0	0	0	0	0	0
0	6	14	3	0	0
0	0	0	0	0	0
0	0	0	0	0	0
0	0	0	0	0	0
0	1	1	0	0	0
0	0	0	1	0	0

0	0	0	0	0	0
0	0	1	0	0	0
0	1	0	0	0	0
0	0	0	0	0	0
0	0	0	0	0	0
0	0	0	0	0	0
0	0	5	0	0	0
0	0	0	0	0	0
0	0	0	0	0	0
0	0	1	0	0	0
0	1	2	0	0	0
0	2	0	0	0	0
0	0	0	3	0	0
0	1	0	0	0	0
0	0	1	1	0	0
0	1	4	0	0	0
0	0	12	1	0	0
0	0	1	0	0	0
0	0	2	2	0	0
0	3	5	0	0	0
0	6	0	0	0	0
0	0	0	0	0	0
0	0	0	0	0	0
0	0	0	0	0	0
0	0	3	0	0	0
0	0	0	0	0	0
0	1	0	0	0	0
0	0	0	0	0	0
0	0	0	0	0	0
0	0	0	0	0	0
0	0	0	0	0	0
0	0	0	0	0	0

0	0	0	0	0	0
0	1	0	0	0	0
0	0	0	0	0	0
0	0	0	0	0	0
0	0	0	0	0	0
0	3	0	0	0	0
0	0	0	0	0	0
0	0	0	0	0	0
0	0	0	0	0	0
0	0	0	0	0	0
0	0	0	0	0	0
0	0	4	0	0	0
0	0	0	0	0	0
0	0	0	0	0	0
0	7	0	0	0	0
0	2	0	1	0	0
0	0	0	1	0	0
0	1	2	0	0	0
0	0	1	0	0	0
0	0	0	0	0	0

0	0	0	0	0	0
0	6	0	0	0	0
0	2	0	0	0	1
0	39	0	0	0	0
0	0	0	0	0	0
0	0	0	0	0	0
0	1	0	0	0	0
0	0	0	0	0	0
0	4	0	0	0	0
0	7	0	0	0	0
0	1	0	0	0	0
0	0	0	0	0	0
0	0	0	0	0	0
0	0	0	0	0	0
0	1	0	1	0	0
0	0	0	0	0	0
0	37	0	0	0	0
0	0	1	0	0	0
0	0	12	0	0	0
0	0	0	0	0	0
0	0	1	0	0	0
0	0	0	0	0	0
0	1	0	0	0	0
0	2	1	0	0	0
0	1	0	0	0	0
0	1	0	0	0	0
0	0	0	0	0	0
0	0	0	0	0	0
0	0	0	0	0	0
0	0	0	0	0	0
0	0	0	0	0	0
0	0	0	0	0	0
0	0	2	0	0	0
0	0	0	0	0	0
0	0	0	0	0	0
0	0	0	0	0	0
0	4	1	0	0	0
0	0	0	1	0	0
0	0	1	2	0	0
0	0	0	0	0	0
0	0	0	0	0	0
0	0	15	1	0	0
0	2	0	0	0	0
0	1	0	0	0	0
0	0	0	0	0	0
0	0	0	0	0	0
0	1	0	0	0	0
0	0	0	0	0	0
0	0	0	0	0	0
0	62	5	1	0	0
0	20	1	0	0	0
0	2	0	0	0	0
0	1	2	0	0	0
0	1	1	0	0	0
0	0	0	0	0	0

0	0	1	0	0	0
0	2	1	0	0	0
0	0	3	0	0	0
0	0	0	0	0	0
0	0	0	0	0	0
0	1	0	0	0	0
0	1	0	0	0	0
0	0	0	0	0	0
0	0	1	0	0	0
0	0	0	0	0	0
0	0	0	0	0	0
0	1	0	0	0	0
0	0	0	0	0	0
0	0	1	0	0	0
0	0	0	0	0	0
0	0	0	0	0	0
0	0	0	0	0	0
0	0	0	0	0	0
0	0	0	0	0	0
0	0	0	0	0	0
0	0	0	0	0	0
0	0	1	2	0	0
0	0	5	0	0	0
0	0	1	2	0	0
0	0	1	1	0	0
0	3	1	0	0	0
0	4	0	0	0	0
0	0	0	0	0	0
0	0	0	0	0	0
0	0	0	0	0	0
0	0	0	0	0	0
0	0	0	0	0	0
0	1	0	0	0	0
0	0	1	0	0	0
0	0	0	0	0	0
0	0	0	0	0	0
0	0	0	0	0	0
0	0	0	0	0	0
0	1	0	0	0	0
0	0	0	0	0	0
0	3	0	0	0	0
0	14	1	0	0	1
0	159	0	1	0	0
0	9	0	0	0	0
0	65	5	3	0	0
0	0	3	0	0	0
0	1	0	0	0	0
0	34	0	0	0	0
0	0	0	0	0	0
0	2	3	5	0	0
0	2	0	1	0	0
0	1	0	0	0	0

scopula_chamb	acremo_chamb	fusa_chamb	rhizo_chamb	mucor_chamb	geotri_chamb
0	0	0	0	0	0
0	0	0	0	0	0
0	0	0	0	0	0
0	0	0	1000	0	0
0	0	0	1000	0	0
0	0	0	1000	0	0
0	0	0	1000	0	0
0	0	0	0	0	0
0	0	0	0	0	0
0	0	0	0	0	0
0	0	0	0	0	0
0	0	0	0	0	0
0	0	0	0	0	0
0	0	0	0	0	0
0	0	0	0	0	0
0	0	0	0	0	0
0	0	0	0	0	0
0	0	0	0	0	0
0	0	0	0	0	0
0	0	0	0	0	0
0	0	0	0	0	0
0	0	0	0	0	0
0	0	0	0	0	0
0	0	0	0	0	0
1	0	0	0	0	0
0	0	0	0	0	0
0	0	0	0	0	0
0	0	0	0	0	0
0	0	0	0	0	0
0	0	0	0	0	0
0	0	0	1000	0	0
0	1	0	0	0	0
0	0	0	0	0	0
0	0	0	0	0	0
0	0	0	0	0	0
0	0	0	0	0	0
0	0	0	1	0	0
0	0	0	1000	0	0
0	0	0	0	0	0
0	0	0	1000	0	0
0	0	0	0	0	0
0	0	0	0	0	0
0	0	0	0	0	0
0	0	0	0	0	0
0	0	0	0	0	0
0	0	0	0	0	0
0	0	0	0	0	0
0	0	0	0	0	0
0	0	0	0	0	0
0	0	0	0	0	0
0	0	0	0	0	0
0	0	0	0	0	0
0	0	0	0	0	0
0	0	0	0	0	0
0	0	0	0	0	0

0	0	0	0	0	0
0	0	0	0	0	0
0	0	0	0	0	0
0	0	0	0	0	0
0	0	0	0	0	0
0	0	0	0	0	0
0	0	0	0	0	0
0	0	0	0	0	0
0	0	0	0	0	0
0	0	0	0	0	0
0	0	0	0	0	0
0	0	0	0	0	0
0	0	1	0	0	0
0	0	0	0	0	0
0	0	0	0	0	0
0	0	0	0	0	0
0	0	0	0	0	0
0	0	0	0	0	0
0	0	0	0	0	0
0	0	0	0	0	0
0	0	0	0	0	0
0	0	0	0	0	0
0	0	0	0	0	0
0	0	0	0	0	0
0	0	0	0	0	0
0	0	0	0	0	0
0	0	0	0	0	0
0	0	0	0	0	0
0	0	0	0	0	0
0	0	0	0	0	0
0	0	0	0	0	0
0	0	0	0	0	0
0	0	0	0	0	0
0	0	0	0	0	0
0	0	0	0	0	0
0	0	0	0	0	0
0	0	0	0	0	0
0	0	0	0	0	0
0	0	0	0	0	0
0	0	0	0	0	0
0	0	0	0	0	0
0	0	0	0	0	0
0	0	0	0	0	0
0	0	0	0	0	0
0	0	0	0	0	0
0	0	0	0	0	0
0	0	0	0	0	0
0	0	0	0	0	0
0	0	0	0	0	0
0	0	0	0	0	0
0	0	0	0	0	0
0	0	0	0	0	0
0	0	0	0	0	0
0	0	0	0	0	0
0	0	0	0	0	0

0	0	0	0	0	0
0	0	0	0	0	0
0	0	0	0	0	0
0	0	0	0	0	0
0	0	0	0	0	0
0	0	0	1000	0	0
0	0	0	1000	0	0
0	0	0	0	0	0
0	0	0	0	0	0
0	0	0	1000	0	0
0	0	0	1000	0	0
0	0	0	0	0	0
0	0	0	0	0	0
0	0	0	1000	0	0
0	0	0	0	0	0
0	0	0	1000	0	0
0	0	0	0	0	0
0	0	0	0	0	0
0	0	0	0	0	0
0	0	0	0	1	0
0	0	0	0	0	0
0	0	0	0	0	0
0	0	0	0	0	0
0	0	0	0	0	0
0	0	0	0	0	0
0	0	0	0	0	0
0	0	0	0	0	0
0	0	0	0	0	0
0	0	0	0	0	0
0	0	0	0	0	0
0	0	0	0	0	0
0	0	0	0	0	0
0	0	0	0	0	0
0	0	0	0	0	0
0	0	0	0	0	0
0	0	0	0	0	0
0	0	0	0	0	0
0	0	0	0	0	0
0	0	0	0	0	0
0	1	0	0	0	0
0	0	0	0	0	0
0	0	0	0	0	0
0	0	0	0	0	0
0	0	0	0	0	0
0	0	0	0	0	0
0	0	0	0	0	0
0	0	0	0	0	0
0	0	0	0	0	0
0	0	0	1000	0	0
0	0	0	0	0	0
0	0	0	0	0	0
0	0	0	0	0	0
0	0	0	0	0	0
0	0	0	0	0	0

0	0	0	0	0	0
0	0	0	0	0	0
0	0	0	0	0	0
0	0	0	0	0	0
0	0	0	0	0	0
0	0	0	0	0	0
0	0	0	0	0	0
0	0	0	0	0	0
0	0	0	0	0	0
0	0	0	0	0	0
0	0	0	0	0	0
0	0	0	0	0	0
0	0	0	0	1	0
0	0	0	0	0	0

0	0	0	0	0	0
0	0	0	0	0	0
0	0	0	0	0	0
0	0	0	0	0	0
0	0	0	0	0	0
0	0	0	0	0	0
0	0	0	0	0	0
0	0	0	0	0	0
0	0	0	0	0	0

0	0	0	0	0	0
0	0	0	0	0	0
0	0	0	0	0	0
0	0	0	0	0	0
0	0	0	0	0	0
0	0	0	0	0	0
0	0	0	0	0	0
0	0	0	0	0	0
0	0	0	0	0	0
0	0	0	0	0	0
0	0	0	0	0	0
2	0	0	0	0	0
0	0	0	0	0	0
0	0	0	0	0	0
0	0	0	0	0	0
0	0	0	1000	0	0
0	0	0	1000	0	0
0	0	0	0	0	0
0	0	0	0	0	0
0	0	0	0	0	0
0	0	0	0	0	0
0	0	0	0	0	0
0	0	0	0	0	0
0	0	0	0	0	0
0	0	0	0	0	0
1	0	0	0	0	0
0	0	0	0	0	0
0	0	0	0	1	0
0	0	0	0	0	0

0	0	0	0	0	0
0	0	0	0	0	0
0	0	0	0	0	0
0	0	0	0	0	0
0	0	0	1000	0	0
0	0	0	0	0	0
0	0	0	0	0	0
0	0	0	1000	0	0
0	0	0	0	0	0
0	0	0	0	0	0
0	0	0	0	0	0
0	0	0	0	0	0
0	0	0	0	0	0
0	0	0	0	0	0
0	0	0	0	0	0
0	0	0	0	0	0
0	0	0	0	0	0
0	0	0	0	0	0
0	0	0	0	0	0
0	0	0	0	0	0
0	0	0	0	0	0
0	0	0	0	0	0
0	0	0	0	0	0
0	0	0	0	0	0
0	0	0	0	0	0
0	0	0	0	0	0
0	0	0	0	0	0
0	0	0	0	0	0
0	0	0	0	0	0
0	0	0	0	0	0
0	0	0	0	0	0
0	0	0	0	0	0
0	0	0	0	0	0
0	0	0	0	0	0
0	0	0	0	0	0
0	0	0	0	0	0
0	0	0	0	0	0
0	0	0	0	0	0
0	0	0	0	0	0
0	0	0	0	0	0
0	0	0	0	0	0
0	0	0	0	0	0
0	0	0	0	0	0
0	0	0	0	0	0
0	0	0	0	0	0
0	0	0	0	0	0
0	0	0	0	0	0
0	0	0	0	0	0
0	0	0	0	0	0
0	0	0	0	0	0
0	0	0	0	0	0
0	0	0	0	0	0
0	0	0	0	0	0
0	0	0	0	0	0

0	0	0	0	0	0
0	0	1	0	0	0
0	0	0	0	0	0
0	0	0	0	0	0
0	0	0	0	0	0
0	0	0	0	0	0
0	0	0	0	0	0
0	0	0	0	0	0
0	0	0	0	0	0
0	0	0	0	0	0
0	0	0	0	0	0
0	0	0	0	0	0
0	0	0	0	0	0
0	0	0	0	0	0
0	0	0	0	0	0
0	0	0	0	0	0
0	0	0	0	0	0
0	0	0	0	0	0
0	0	0	0	0	0
0	0	0	0	0	0
0	0	0	0	0	0
0	0	0	0	0	0
0	0	0	0	0	0
0	0	0	0	0	0
0	0	0	0	0	0
0	0	0	0	0	0
0	0	0	0	0	0
0	0	0	0	0	0
0	0	0	0	0	0
0	0	0	0	0	0
0	0	0	0	0	0
0	0	0	0	0	0

0	0	0	0	0	0
0	0	0	0	0	0
0	0	0	0	0	0
0	0	0	0	0	0
0	0	0	0	0	0
0	0	0	0	0	0
0	1	0	0	0	0
0	0	0	0	0	0
0	0	0	0	0	0
0	0	0	0	0	0
0	0	0	0	0	0
0	0	0	0	0	0
0	0	0	0	0	0
0	0	0	0	0	0
0	0	0	0	0	0
0	0	0	0	0	0
0	0	0	0	0	0
0	0	0	0	0	0
0	0	0	0	0	0
0	0	0	0	0	0

0	0	0	1000	0	0
0	0	0	0	0	0
0	0	0	0	0	0
0	0	0	0	0	0
0	0	0	0	0	0
0	0	0	1000	0	0
0	0	0	1000	0	0
0	0	0	1000	0	0
0	0	0	0	0	0
0	0	0	0	0	0
0	0	0	0	0	0
0	0	0	0	0	0
0	0	0	0	0	0
0	0	0	1000	0	0
0	0	0	0	0	0
0	0	0	0	0	0
0	0	0	0	0	0
0	0	0	0	0	0
0	0	0	0	0	0
0	0	0	0	0	0
0	0	0	0	0	0
0	0	0	0	0	0
0	0	0	0	0	0
0	0	0	0	0	0
0	0	0	0	0	0
0	0	0	0	0	0
0	0	0	0	0	0
0	0	0	0	0	0

0	0	0	0	0	0
0	0	0	0	0	0
0	0	0	0	0	0
0	0	0	0	0	0
0	0	0	0	0	0
0	0	0	0	0	0
0	0	0	0	0	0
0	0	0	0	0	0
0	0	0	0	0	0
0	0	0	0	0	0
0	0	0	0	0	0
0	0	0	0	0	0
0	0	0	0	0	0
0	0	0	0	0	0
0	0	0	0	0	0
0	0	0	0	0	0
0	0	0	0	0	0
0	0	0	0	0	0
0	0	0	0	0	0
0	0	0	0	0	0
0	0	0	0	0	0
0	0	0	0	0	0
0	0	0	0	0	0
0	0	0	0	0	0
0	0	0	0	0	0
0	0	0	0	0	0
0	0	0	0	0	0

0	0	0	0	0	0
0	0	0	0	0	0
0	0	1	0	0	0
0	0	0	0	0	0
0	0	0	0	0	0
0	0	0	0	0	0
0	0	0	0	0	0
0	0	0	0	0	0
0	0	0	0	0	0
0	0	0	0	0	0
0	0	0	0	0	0
0	0	0	0	0	0
0	0	0	0	0	0
0	0	0	0	0	0
0	0	0	0	0	0
0	0	0	0	0	0
0	0	0	0	0	0
0	0	0	0	0	0
0	0	0	0	0	0
0	0	0	0	0	0
0	0	0	0	0	0
0	0	0	0	0	0
0	0	0	0	0	0
0	0	0	0	0	0
0	0	0	0	0	0
0	0	0	0	0	0
0	0	0	0	0	0
0	0	0	0	0	0
0	0	0	0	0	0
0	0	0	0	0	0
0	0	0	0	0	0
0	0	0	1000	0	0
0	0	0	0	0	0
0	0	0	0	0	0
0	0	0	0	0	0
0	0	0	0	0	0
0	0	0	0	0	0
0	0	0	0	0	0
0	0	0	0	0	1
0	0	0	0	0	0
0	0	0	0	0	0
0	0	0	0	0	0
0	0	0	0	0	0
0	1	0	0	0	0
0	0	0	0	0	0
0	0	0	0	0	0
0	0	0	0	0	0
0	0	0	0	0	0
0	0	0	0	0	0
0	0	0	0	0	0
0	0	0	0	0	0
0	0	0	0	0	0

trichoderm_chamb	paecilo_chamb	autres_chamb	csd_chamb	levure_chamb	asp_fumi_coul
0	0	0	0	0	0
0	0	0	0	0	0
0	0	0	0	0	0
0	0	0	0	0	0
0	0	0	0	0	0
0	0	0	0	0	0
0	0	0	0	0	0
0	0	0	0	1	0
0	0	0	1	0	2
0	3	0	0	26	0
0	0	0	4	0	0
0	0	0	0	0	0
0	0	0	1	0	0
0	0	0	2	1	0
0	0	0	2	23	1
0	0	2	3	0	0
0	0	0	1	0	0
0	0	0	1	0	0
0	0	0	3	2	0
0	0	0	1	0	1
0	0	0	1	2	0
0	1	0	0	1	0
0	0	0	1	0	0
0	0	0	1	0	0
0	0	0	2	1	0
0	0	0	1	0	0
0	0	0	1	0	0
0	0	0	0	0	0
0	0	0	0	1	0
0	0	0	4	0	0
0	0	0	0	0	1
0	0	0	0	0	0
0	0	0	0	0	1
0	0	0	0	0	1
0	0	0	0	0	0
0	0	0	0	0	0
0	0	0	1	0	0
0	0	0	0	0	0
0	0	0	0	1	0
0	0	0	0	0	0
0	2	0	0	2	0
0	0	0	0	0	0
0	0	0	5	0	0
0	0	0	3	0	0
0	0	1	0	0	0
0	0	0	4	1	0
0	0	0	0	0	0
0	0	0	3	0	0
0	0	0	0	0	0
0	0	0	2	0	0
0	0	1	0	1	0
0	0	0	0	0	0
0	0	0	0	1	0
0	0	0	0	0	0
0	0	0	0	0	0

0	0	1	0	0	0
0	0	0	1	0	0
0	0	0	0	0	0
0	0	0	1	0	0
0	0	0	3	0	1
0	0	0	0	2	1
0	0	0	2	2	0
0	0	0	0	1	0
0	0	0	1	0	0
0	0	0	2	0	3
0	0	0	0	0	0
0	0	0	0	0	0
0	0	0	0	1	0
0	1	0	0	0	1
0	0	0	0	2	0
0	0	0	0	0	0
0	0	0	0	0	0
0	0	0	0	0	0
0	0	0	0	0	6
0	0	0	0	0	0
0	0	0	1	0	0
0	0	0	0	0	0
0	0	0	0	0	1
0	0	0	1	0	0
0	0	0	1	0	0
0	0	0	3	0	0
0	1	0	0	1	0
0	0	0	0	1	0
0	0	0	0	0	0
0	0	2	0	0	1
0	0	0	0	0	0
0	0	0	1	0	0
0	0	0	0	0	0
0	0	0	0	0	0
0	0	0	0	0	0
0	0	0	0	0	0

0	0	1	0	0	0
0	0	0	1	0	0
0	0	0	8	2	0
0	0	0	2	0	0
0	0	0	6	0	0
0	0	0	2	0	0
0	0	0	5	2	0
0	0	0	0	0	0
0	0	0	0	0	0
0	0	0	2	1	0
0	0	0	1	1	0
0	0	0	0	2	0
0	0	0	3	0	0
0	0	0	0	0	0
0	0	0	1	0	0
0	0	0	3	2	0
0	0	0	1	1	1
0	0	0	0	0	0
0	0	0	0	0	0

0	0	0	0	0	0
0	0	0	0	1	0
0	0	0	0	0	0
0	0	0	0	0	1
0	0	0	0	0	0
0	0	0	0	0	0
0	0	0	0	0	0
0	0	0	0	0	0
0	0	0	0	0	4
0	0	0	0	0	0
0	0	0	0	1	0
0	0	0	0	0	0
0	0	0	0	0	1
0	1	0	43	0	1
0	0	0	0	0	0
0	0	0	0	0	0
0	22	0	0	0	0
0	0	0	0	0	0
0	1	0	1	0	0
0	0	0	0	3	1
0	0	0	1	1	0
0	0	0	0	1	0
0	0	0	1	1	1
0	0	0	1	1	0
0	0	0	2	0	1
0	0	0	0	0	0
0	0	0	0	0	0
0	0	0	0	0	0
0	0	0	1	1	0
0	0	0	1	0	0
0	0	0	2	0	0
0	0	0	0	0	0
0	0	0	2	1	0
0	0	0	0	0	0
0	0	0	0	0	0

0	0	0	0	0	0
0	0	0	0	0	0
0	0	0	0	0	0
0	1	1	0	0	0
0	0	0	3	0	0
0	0	0	1	0	0
0	0	1	1	0	0
0	0	0	3	0	2
0	1	0	0	0	0
0	0	0	0	0	0
0	1	0	0	1	0
0	0	0	1	0	1
0	0	0	0	1	0
0	0	0	0	0	0

0	0	0	0	0	0
0	0	0	0	0	0
0	0	0	2	0	0
0	0	0	0	2	0
0	0	0	0	0	0

0	0	0	1	1	0
0	0	0	0	1	0
0	0	0	2	1	1
0	1	1	0	0	0
0	0	0	1	0	0
0	0	0	1	0	0
0	0	0	4	0	0
0	0	0	0	0	0
0	0	0	2	0	0
0	0	0	1	2	1
0	0	0	1	0	0
0	0	0	0	0	0
0	0	0	1	1	0
0	0	0	1	0	0
0	0	0	0	0	0
0	0	0	1	0	0
0	1	0	0	0	0
0	0	0	6	0	0
0	0	0	4	1	0
0	0	0	2	3	0
0	0	0	8	1	0
0	0	0	3	1	0
0	0	0	1	1	0
0	0	1	0	0	0
0	0	0	0	0	0
0	0	0	0	0	0
0	0	0	0	0	0
0	0	0	14	0	0
0	0	0	0	0	0
0	0	0	0	0	0
0	0	0	0	15	0
0	1	0	1	2	0
0	0	0	0	0	0
0	0	0	0	1	0
0	0	1	3	17	0
0	0	0	1	1	0
0	1	1	0	33	1
0	0	0	0	2	0
0	0	0	0	0	0
0	0	0	0	0	0
0	0	0	0	1	0
0	0	0	0	0	0
0	0	0	0	0	0
0	0	0	24	0	0
0	0	0	0	5	0
0	0	0	0	0	0
0	0	0	0	0	1
0	0	0	0	1	0
0	0	0	0	0	0
0	0	0	0	0	1
0	0	0	0	2	0
0	0	0	0	0	0

0	0	0	0	0	0
0	0	0	0	0	0
0	0	0	0	0	0
0	0	0	0	2	0
0	0	0	0	0	1
0	0	0	0	0	0
0	0	0	1	1	0
0	0	3	0	0	0
0	0	0	0	0	0
0	0	0	0	0	0
0	0	1	0	0	0
0	0	0	0	0	1
0	0	0	0	0	0
0	0	0	0	0	0
0	0	0	1	1	0
0	0	0	0	0	0
0	0	0	0	1	0
0	0	0	0	0	0
0	0	0	0	0	0
0	2	0	3	1	0
0	0	0	0	0	0
0	0	1	0	0	0
0	0	0	0	0	1
0	0	0	0	0	1
0	0	0	0	0	0

0	0	0	0	5	0
0	0	0	0	0	0
0	0	0	0	0	0
0	0	0	0	0	0
0	0	0	0	0	0
0	0	0	0	0	0
0	0	0	0	1	0
0	0	0	0	0	0
0	0	0	0	0	0
0	0	0	0	0	0
0	0	0	0	1	0
0	1	0	0	0	0
0	0	0	0	1	0
0	0	0	0	0	3
0	0	0	0	0	0
0	0	0	0	1	0
0	0	0	0	0	2
0	0	0	0	0	0
0	0	0	0	0	0
0	0	0	0	0	0
0	0	0	1	0	0
0	0	0	0	1	0
0	0	0	0	0	0
0	0	0	2	5	0

0	0	0	0	0	0
0	0	0	0	0	0
0	0	0	0	0	0
0	0	0	1	0	0
0	0	0	1	0	1

0	0	1	0	0	2
0	0	0	0	0	0
0	0	1	0	0	1
0	0	0	0	0	0
0	0	0	0	0	0
0	0	0	0	0	0
0	0	0	0	0	0
0	0	0	0	0	0
0	0	0	0	0	0
0	0	0	1	0	0
0	0	0	2	0	0
0	0	0	2	0	0
0	0	0	0	0	0
0	0	0	0	0	0
0	0	0	4	0	0
0	0	0	6	0	0
0	0	0	2	1	0
0	0	0	2	0	0
0	0	0	6	0	0
0	0	0	0	4	0
0	0	0	0	0	0
0	0	0	0	0	0
0	0	0	1	0	0
0	0	0	2	0	0
0	0	0	2	1	0
0	0	0	2	0	0
0	0	0	0	0	0
0	0	0	0	0	0
0	0	0	0	0	0
0	0	0	0	0	0
0	0	0	0	0	0
0	0	0	0	0	0

0	0	0	0	0	0
0	0	0	1	0	0
0	0	0	0	1	0
0	0	0	0	0	0
0	0	0	0	2	0
0	0	0	1	0	0
0	0	0	2	1	0
0	0	0	0	0	0
0	0	0	0	0	0
0	0	1	0	0	0
0	0	0	0	0	0
0	0	0	2	0	0
0	0	0	0	0	1
0	0	0	0	0	0
0	0	0	2	0	2
0	0	0	0	0	0
0	0	0	2	0	0
0	0	0	0	0	0
0	0	0	0	0	0
0	0	0	0	0	0

0	0	80	0	0	0
0	0	0	1	0	1
0	0	0	1	1	0
0	0	0	2	0	0
0	0	0	2	1	0
0	0	0	0	1	4
0	0	0	0	0	2
0	0	0	0	0	0
0	0	0	1	1	1
0	0	0	0	17	0
0	0	0	1	1	0
0	0	0	0	0	0
0	0	0	0	0	0
0	0	8	0	0	0
0	0	0	0	1	0
0	0	0	0	0	1
0	0	0	0	0	2
0	0	0	0	0	0
0	0	1	0	0	1
0	0	0	0	0	0
0	0	0	0	0	0
0	0	0	0	0	0
0	0	0	0	0	0
0	0	0	0	0	0
0	0	0	0	0	0
0	0	0	0	0	0
0	0	2	1	0	0
0	0	1	0	0	0
0	0	0	0	0	0
0	0	0	0	0	0
0	0	0	0	0	0
0	0	0	2	0	0
0	0	0	0	0	0
0	0	0	0	0	0
0	0	0	0	0	1
0	0	0	7	0	0
0	0	0	1	0	0
0	0	0	1	0	0
0	0	0	2	6	0
0	0	0	2	0	0
0	0	0	4	0	0
0	0	0	7	0	0
0	0	0	1	0	0
0	0	0	3	3	0
0	0	0	1	0	0
0	0	0	0	0	0
0	0	0	2	0	0
0	0	0	0	1	0
0	0	0	0	0	0
0	0	0	0	1	0
0	0	0	0	1	0
0	0	0	0	1	0
0	1	0	0	0	0
0	0	0	0	0	0
0	0	0	1	1	0

0	0	0	4	0	0
1	0	0	2	0	0
0	0	0	0	0	0
0	0	0	1	0	0
0	0	0	1	0	0
0	0	0	0	0	0
0	0	0	3	5	0
0	0	0	0	0	0
0	0	0	0	1	0
0	0	0	0	0	0
0	0	0	7	0	0
0	0	0	0	1	0
0	0	0	0	0	0
0	0	0	0	0	0
0	0	0	0	0	0
0	0	0	0	0	0
0	0	0	0	0	0
0	0	0	0	1	0
0	0	0	0	1	0
0	0	0	0	0	0
0	0	0	0	0	0
0	0	0	22	0	0
0	0	0	2	0	0
0	0	0	0	0	0
0	0	0	0	0	0
0	0	0	0	0	0
0	0	0	0	0	0
0	0	0	0	0	0
0	0	0	0	0	0
0	0	0	0	0	0
0	0	0	0	0	0
0	0	0	0	0	0
0	0	0	0	0	0
0	0	0	0	0	0
0	0	0	0	0	0
0	0	0	1	0	1
0	0	0	1	0	0
0	0	0	0	1	0
0	0	0	0	0	0
0	0	0	0	0	0
0	0	0	3	0	0
0	0	0	0	0	0
0	0	0	1	0	0
0	0	1	2	0	0
0	0	0	3	0	0
0	0	0	0	2	0
0	0	0	1	0	0
0	0	0	0	0	0
0	0	0	1	0	0
0	0	0	3	0	0
0	0	0	2	3	0
0	0	0	1	0	0

sct_nid_coul	asp_nid_coul	asp_ustus_coul	asp_niger_coul	asp_sp_coul	asp_terreus_coul
1	0	0	0	0	0
0	0	0	0	0	0
0	0	0	0	0	0
0	0	0	0	0	0
0	0	0	0	0	0
0	0	0	0	0	0
0	0	0	0	0	0
2	0	0	0	0	0
0	0	0	0	0	0
0	0	0	0	0	0
0	0	0	0	0	0
0	0	0	0	0	0
0	0	0	0	1	0
3	0	0	0	1	0
1	0	0	0	0	0
0	0	0	0	0	0
0	0	0	0	0	0
0	0	0	0	4	0
0	0	0	0	0	0
0	0	0	0	0	0
0	0	0	0	1	0
1	0	0	0	0	0
0	0	0	0	0	0
2	0	0	0	0	0
0	0	0	0	1	0
0	0	0	0	1	0
0	0	0	0	1	0
0	0	0	0	0	0
0	0	0	0	0	0
0	0	0	0	0	0
0	0	0	0	0	0
0	0	0	0	0	0
0	0	0	0	0	0
0	0	0	0	0	0
0	0	0	0	0	0
0	0	0	0	0	0
0	0	0	0	1	0
6	0	0	0	0	0
0	0	0	0	0	0
0	0	2	0	0	0
0	0	0	0	1	0
0	0	0	0	0	0
0	0	0	0	0	0
0	0	0	0	0	0
0	0	0	0	0	0
0	0	0	0	1	0
0	0	0	0	0	0
0	0	0	0	0	0
0	0	0	0	2	0
0	0	0	0	0	0
0	0	0	0	0	0
0	0	1	0	0	0
0	0	0	0	0	0
0	0	0	0	2	0
0	0	0	0	0	0

0	0	1	0	0	0
0	0	0	0	0	0
0	0	0	0	1	0
0	0	0	0	0	0
0	0	0	0	0	0
0	0	0	0	0	0
1	0	0	0	0	0
0	0	0	0	0	0
0	0	0	0	0	0
0	0	0	0	0	0
0	0	0	0	1	0
1	0	0	0	0	0
0	0	0	0	0	0
0	0	0	0	0	0
0	0	0	0	0	0
0	0	0	0	0	0
0	0	0	0	0	0
0	0	0	0	0	0
0	0	0	1	0	0
0	0	0	0	0	0
0	0	0	0	0	0
0	0	0	0	1	0
0	0	0	1	0	0
0	0	0	0	0	0
0	0	0	0	0	0
0	0	1	0	0	0
0	0	0	0	0	0
3	0	0	0	0	0
0	0	0	0	0	0
0	0	0	0	0	0
0	0	0	0	0	0
0	0	0	0	1	0
0	0	0	0	0	0
0	0	0	0	1	0
0	0	0	0	0	0
0	0	0	0	0	0

0	0	0	0	0	0
0	0	0	0	0	0
2	0	0	0	0	0
0	0	0	0	0	0
0	0	0	0	0	0
0	0	0	0	0	0
0	0	0	0	2	0
0	0	0	0	0	0
4	0	0	0	0	0
0	0	0	0	0	0
0	0	0	0	0	0
0	0	0	0	0	0
2	0	0	0	0	0
0	0	0	0	2	0
1	0	0	0	0	0
6	0	0	0	0	0
0	0	0	0	2	0
0	0	0	0	1	0
0	0	0	0	0	0

0	0	1	0	0	0
0	0	0	0	0	0
0	0	0	0	0	0
0	1	0	0	0	0
0	0	0	0	0	0
0	0	0	0	0	0
0	0	0	0	0	0
0	0	0	0	0	0
0	0	0	0	0	0
0	0	1	0	0	0
0	0	0	0	0	0
0	0	0	0	0	0
0	0	0	0	0	0
0	0	0	0	2	0
0	0	0	0	0	0
0	0	0	0	0	0
0	0	0	0	0	0
0	0	0	0	1	0
0	0	0	0	0	0
0	0	0	2	0	0
0	0	0	0	0	0
0	0	0	0	0	0
0	0	0	0	0	0
0	0	0	0	0	0
0	0	0	0	0	0
0	0	0	0	0	0
0	0	0	0	0	0
0	0	0	0	1	0
1	0	0	0	0	0
0	0	0	0	0	0
8	0	2	0	0	0
2	0	0	0	0	0
0	0	0	0	0	0
0	0	0	0	0	0
2	0	0	0	0	0
0	0	0	0	1	0
1	0	0	0	0	0
0	0	0	0	0	0
0	0	0	0	1	0
1	0	0	0	0	0
0	0	0	0	0	0
0	0	0	0	0	0
0	0	0	1	0	0
0	0	0	0	0	0
0	0	0	0	0	0
0	0	0	0	0	0
0	0	0	0	0	0
0	0	0	0	0	0
0	0	0	0	0	0
0	0	0	0	0	0
0	0	0	0	0	0
0	0	0	0	0	0
1	0	0	0	0	0
1	0	0	1	0	0

0	0	0	0	0	0
0	0	0	0	0	0
0	0	0	0	0	0
1	0	0	0	0	0
0	0	0	0	0	0
0	0	0	0	0	0
0	0	0	0	0	0
0	0	0	0	0	0
0	0	0	0	0	0
0	0	0	0	0	0
0	0	0	0	0	0
0	0	0	0	0	0
1	0	0	0	0	0
0	0	0	0	0	0
0	0	0	0	3	0
0	0	0	1	0	0
0	0	0	0	0	0
0	0	0	0	0	0
3	0	0	0	0	0
0	0	1	0	2	0
0	0	0	0	1	0
0	0	1	0	6	0
1	0	0	0	0	0
0	0	0	0	0	0
0	0	0	0	0	0
1	0	0	0	0	0
0	0	0	0	0	0
0	0	0	0	0	0
0	0	0	0	0	0
0	0	0	0	0	0
0	0	0	0	0	0
0	0	0	0	0	0
0	0	0	0	0	0
1	0	0	0	0	0
0	0	0	0	0	0
3	0	0	0	0	0
0	0	0	0	0	0
0	0	0	0	0	0
0	0	0	0	0	0
0	0	0	0	0	0
0	0	0	0	0	0
0	0	0	0	0	0
0	0	0	0	1	0
0	0	0	0	0	0
1	0	0	0	0	0
0	0	0	0	0	0
3	0	0	0	0	0
0	0	0	0	0	0
2	0	0	0	0	0
0	0	0	0	0	0
0	0	0	0	0	0
0	0	0	0	0	0

1	0	0	0	0	0
1	0	0	0	0	0
0	0	0	0	0	0
0	0	0	0	5	0
3	0	0	1	0	0
0	0	0	0	0	0
0	0	0	0	0	0
0	0	0	0	0	0
0	0	0	0	3	0
0	0	0	0	0	0
0	0	0	0	0	0
3	0	0	1	0	0
0	0	0	0	0	0
0	0	0	0	0	0
0	0	0	0	2	0
1	0	0	0	0	0
1	0	0	0	0	0
1	0	0	0	0	0
0	0	1	0	0	0
0	0	0	0	0	0
1	0	0	0	0	0
0	0	0	0	0	0
0	0	0	0	0	0
2	0	0	0	0	0
0	0	0	0	0	0
0	0	0	1	0	0
0	0	0	0	0	0
0	0	0	0	0	0
0	0	0	0	0	0
0	0	0	0	0	0
0	0	0	0	0	0
0	0	0	0	0	0
0	0	0	0	0	0
0	0	0	0	0	0
0	0	0	0	0	0
0	0	0	0	0	0
0	0	0	0	0	0
0	0	0	0	0	0
0	0	0	0	0	0
0	0	0	0	0	0
0	0	0	0	0	0
0	0	0	0	0	0
0	0	0	0	0	0
0	0	0	0	0	0
0	0	0	0	0	0
0	0	0	0	0	0
0	0	0	0	0	0
9	0	0	0	0	0
0	0	0	0	0	0
0	0	0	0	0	0
2	0	0	0	0	0
0	0	0	0	0	0
0	0	0	0	0	0
0	0	0	0	0	0

2	0	0	0	0	0
0	0	0	0	0	0
0	0	0	0	0	0
0	0	0	0	0	0
0	0	0	0	0	0
0	0	0	0	0	0
0	0	1	0	0	0
0	0	0	0	0	0
0	0	0	0	0	0
0	0	0	0	0	0
0	0	0	0	0	0
3	0	0	0	0	0
0	0	0	0	0	0
1	0	0	0	0	0
0	0	0	0	1	0
0	0	0	0	0	0
0	0	0	0	0	0
0	0	0	0	0	0
0	0	0	0	0	0
0	0	0	0	0	0
0	0	0	0	0	0
0	0	0	0	1	0
36	0	0	0	0	0
4	0	0	0	0	0
5	0	0	0	0	0
0	0	0	0	0	0
2	0	0	0	0	0
0	0	0	0	0	0
0	0	0	0	0	0
0	0	0	0	0	0
0	0	0	0	0	0
0	0	0	0	0	0

0	0	0	0	0	0
0	0	0	0	0	0
0	0	0	0	0	0
0	0	0	0	0	0
0	0	0	0	1	0
0	0	0	0	0	0
0	0	0	0	0	0
0	0	0	0	0	0
0	0	0	0	0	0
0	1	0	0	0	0
0	0	0	0	0	0
1	0	0	1	0	0
0	0	0	0	0	0
0	0	0	0	0	0
0	0	0	0	0	0
0	0	0	0	0	0
0	0	0	0	0	0
0	0	0	0	0	0
0	0	0	0	0	0
0	0	0	0	0	0

5	0	7	0	0	0
0	0	0	0	0	0
0	0	0	0	0	0
0	0	0	0	0	0
0	0	0	0	0	0
0	0	0	0	0	0
0	0	0	0	1	0
0	0	0	0	0	0
1	0	1	0	6	0
0	0	1	0	2	0
0	0	0	0	10	0
0	0	0	0	0	0
0	0	0	0	0	0
0	0	0	0	1	0
0	0	0	0	0	0
0	0	0	0	0	0
2	0	0	0	0	0
0	0	0	0	0	0
0	0	0	0	0	0
0	0	0	0	0	0
0	0	0	0	0	0
0	0	0	0	0	0
2	0	0	0	0	0
0	0	0	0	0	0
0	0	0	0	0	0
0	0	0	0	0	0
0	0	0	0	0	0
1	0	0	0	0	0
0	0	0	0	0	0
0	0	0	0	0	0
1	0	0	0	0	0
0	0	0	0	0	0
4	0	0	0	0	0
0	0	0	0	0	0
0	0	0	0	0	0
2	0	0	0	0	0
0	0	0	0	0	0
0	0	0	0	0	0
0	0	0	0	0	0
0	0	0	0	0	0
0	0	0	0	0	0
1	0	0	0	0	0
0	0	0	0	0	0
0	0	0	0	1	0
0	0	0	0	0	0
0	0	0	0	0	0
0	0	0	0	0	0
0	0	0	0	0	0
0	0	0	0	0	0
0	0	0	0	0	0
0	0	0	0	0	0
0	0	0	0	1	0
0	0	0	0	0	0
0	0	0	0	0	0
0	0	0	1	0	0

0	0	0	0	0	0
0	0	0	0	2	0
0	0	0	0	0	0
0	0	0	0	0	0
0	0	0	0	0	0
0	0	0	0	0	0
0	0	0	0	0	0
0	0	0	0	0	0
0	0	0	0	0	0
0	0	0	0	0	0
0	0	0	0	0	0
0	0	0	0	0	0
0	0	0	0	0	0
0	0	0	0	0	0
0	0	0	0	0	0
0	0	0	0	0	0
0	0	0	0	0	0
0	0	0	0	0	0
0	0	0	0	0	0
0	0	0	0	0	0
0	0	0	0	0	0
1	0	0	0	0	0
0	0	0	0	0	0
0	0	0	0	0	0
0	0	0	0	0	0
0	0	0	0	0	0
0	0	0	0	0	0
0	0	0	0	0	0
0	0	0	0	0	0
0	0	0	0	0	0
0	0	0	0	0	0
0	0	0	0	0	0
0	0	0	0	0	0
0	0	0	0	1	0
0	0	0	0	0	0
0	0	0	0	0	0
0	0	0	0	1	0
0	0	0	0	0	0
0	0	0	0	0	0
0	0	0	0	0	0
0	0	0	0	1	0
0	0	0	0	0	0
0	0	0	0	0	0
0	0	0	0	0	0
0	0	0	0	0	0
0	0	0	0	0	0
0	0	0	0	0	0
0	0	0	0	0	0
0	0	0	0	0	0
0	0	0	0	0	0
0	0	0	0	0	0
0	0	0	1	0	0

asp_flavus_coul	peni_coul	clado_coul	alterna_coul	ulocla_coul	chaeto_coul	scopula_coul
0	0	0	0	0	0	0
0	3	0	0	0	0	0
0	0	0	0	0	0	0
0	0	0	0	0	0	0
0	0	0	0	0	0	0
0	1	1	0	0	0	0
0	0	0	0	0	0	0
0	0	0	0	0	0	0
0	5	0	0	0	0	0
0	9	0	0	0	0	0
0	0	1	0	0	0	0
0	0	0	0	0	0	0
0	1	0	0	0	0	0
0	0	6	1	0	0	0
0	0	20	1	0	0	0
0	15	2	0	0	0	0
0	37	2	0	0	0	0
0	1	1	0	0	0	0
0	20	1	0	0	0	0
0	2	0	0	0	0	0
0	0	0	0	0	0	0
0	1	0	0	0	1	0
0	1	0	0	0	0	0
0	1	0	0	0	0	0
0	4	1	0	0	0	0
0	1	1	0	0	0	0
0	0	0	0	0	0	0
0	0	0	0	0	0	0
0	2	0	0	0	0	0
0	2	0	0	0	0	0
0	0	0	0	0	0	0
0	2	0	0	0	0	0
0	0	0	0	0	0	0
0	0	0	0	0	0	0
0	1	0	0	0	0	0
0	0	1	0	0	0	0
0	8	2	0	0	0	0
0	1	1	0	0	0	0
0	0	0	0	0	0	0
0	1	0	0	0	1	0
0	19	1	0	0	0	0
0	1	5	0	0	0	0
0	0	1	1	0	0	0
0	1	0	0	0	0	0
0	0	0	0	0	0	0
0	0	0	0	0	0	0
0	0	0	1	0	0	0
0	2	1	1	0	0	0
0	36	1	0	0	0	0
0	0	2	0	0	0	0
0	1	0	0	0	0	0
0	0	0	0	1	0	0
0	1	0	1	0	0	0
0	9	0	0	0	0	0
0	0	0	0	0	0	0

0	1	0	0	0	0	0
0	0	0	0	0	0	0
0	0	0	0	0	0	0
1	0	0	0	0	0	0
0	1	0	1	0	0	0
0	0	0	0	0	0	0
0	1	3	0	0	0	0
0	0	0	0	0	0	0
0	1	10	0	0	0	0
1	3	0	0	0	0	0
0	0	0	0	0	0	0
0	1	0	0	0	0	0
0	2	1	0	0	0	0
0	2	0	0	0	0	0
0	4	0	0	0	0	0
0	1	0	0	0	0	0
0	1	0	0	0	0	0
0	0	0	0	0	0	0
0	0	0	0	0	0	0
0	0	0	0	0	0	0
0	0	0	0	0	0	0
0	0	0	0	0	0	0
0	1	0	0	0	0	0
0	0	0	0	0	0	0
0	0	1	0	0	0	0
0	0	0	1	0	0	0
0	2	0	0	0	0	0
0	0	0	0	0	0	0
0	2	2	0	0	0	0
0	2	0	0	0	0	0
0	0	0	0	0	0	0
0	1	2	0	0	0	0
0	0	0	0	0	0	0
0	0	0	0	0	0	0
0	0	0	0	0	0	0
0	0	0	0	0	0	0

0	0	0	0	0	0	0
0	0	0	1	0	0	0
0	0	2	1	0	0	0
0	0	0	0	0	0	0
0	1	6	3	0	0	0
0	0	0	0	0	0	0
0	0	0	0	0	0	0
0	1	0	1	0	0	0
0	3	1	0	0	0	0
0	0	0	0	0	0	0
0	14	0	0	0	0	0
0	0	0	0	0	0	0
0	28	1	0	0	0	0
0	3	0	0	0	0	0
0	0	0	2	0	0	0
0	0	1	0	0	0	0
0	0	0	0	0	0	0
0	1	0	0	0	0	0
0	1	0	0	0	0	0

0	0	0	0	0	0	0
0	2	0	0	0	0	0
0	1	0	0	0	0	0
0	0	0	0	0	0	0
0	1	0	0	0	0	0
0	0	0	0	0	0	0
0	0	0	0	0	0	0
0	0	0	0	0	0	0
0	1	0	0	0	0	0
0	1	0	0	0	0	0
0	0	0	0	0	0	0
0	0	0	0	0	0	0
0	0	0	0	0	0	0
0	1	1	0	0	0	0
0	0	0	0	0	0	0
0	2	0	0	0	0	0
0	0	0	0	0	0	0
0	1	0	0	0	0	0
0	1	1	0	0	0	0
0	0	0	0	0	0	0
0	1	0	0	0	0	0
0	0	0	0	0	0	0
0	0	0	0	0	0	0
0	0	0	0	0	0	0
0	1	1	0	0	0	0
0	5	1	0	0	0	0
0	0	0	0	0	0	0
0	0	0	0	0	0	0
0	65	7	1	0	0	0
0	0	0	0	0	0	0
0	8	0	0	0	0	0
0	0	0	0	0	0	0
0	0	0	0	0	0	0
0	0	0	0	0	0	0
0	0	0	0	0	0	0
0	0	0	0	0	0	0
0	0	0	0	0	0	0
0	0	0	1	0	0	0
0	0	2	0	0	0	0
0	1	6	0	0	0	0
0	1	1	1	0	0	0
0	1	1	2	0	0	0
0	32	0	0	0	0	0
0	1	0	1	0	0	0
0	0	0	0	0	0	0
0	0	0	0	0	0	0
0	0	0	0	0	0	0
0	0	2	0	0	0	0
0	0	0	0	0	0	0
0	2	0	0	0	0	0
0	0	0	0	0	0	0
0	0	0	0	0	0	0
0	1	0	0	0	0	0
0	0	0	0	0	0	0

0	0	7	0	0	0	0
0	0	0	0	0	0	0
0	0	2	0	0	0	0
0	0	0	0	0	0	0
0	0	0	0	0	0	0
0	0	5	0	0	0	0
0	0	0	0	0	0	0
0	0	2	0	0	0	0
0	0	0	0	0	0	0
0	0	2	0	0	0	0
0	5	0	0	0	0	0
0	0	0	0	0	0	0
0	1	0	1	0	0	0
0	3	0	0	0	0	0
0	2	0	0	0	0	0
0	1	1	0	0	0	0
0	2	6	0	0	0	0
0	1	1	3	0	0	0
0	0	9	0	0	0	0
0	1	0	0	0	0	0
0	1	1	0	0	0	0
0	0	3	0	0	0	0
0	7	1	0	0	0	0
0	0	0	0	0	0	0
0	0	0	0	0	0	0
0	0	0	0	0	0	0
0	0	0	0	0	0	0
0	0	0	0	0	0	0
0	0	0	1	0	0	0
0	1	0	0	0	0	0
0	65	0	0	0	0	0
0	1	1	0	0	0	0
0	5	3	0	0	0	0
0	0	0	0	0	0	0
0	0	0	0	0	0	0
0	0	1	0	0	0	0
0	0	0	0	0	0	0
0	0	1	0	0	0	0
0	0	0	0	0	0	0
0	5	0	0	0	0	0
0	0	0	0	0	0	0
0	0	0	0	0	0	0
0	2	0	0	0	0	0
0	0	0	0	0	0	0
0	0	0	0	0	0	0
0	0	0	0	0	0	0
0	0	0	0	0	0	0
0	2	0	0	0	0	0
0	0	1	0	0	0	0
0	0	0	0	0	0	0
0	0	0	0	0	0	0
0	0	0	0	0	0	0

0	0	0	0	0	0	0
0	0	0	0	0	0	0
0	0	0	0	0	0	0
0	3	0	0	0	0	0
0	12	0	0	0	0	0
0	0	0	0	0	0	0
0	0	0	0	0	0	0
0	0	0	0	0	0	0
0	8	0	0	0	0	0
0	1	0	0	0	0	0
0	0	0	0	0	0	0
0	12	0	0	0	0	0
0	1	0	0	0	0	0
0	0	0	0	0	0	0
0	1	0	0	0	0	0
0	1	1	0	0	0	0
0	1	0	1	0	0	0
0	0	2	0	0	0	0
0	0	3	0	0	0	0
0	4	2	0	0	0	0
0	5	0	0	0	0	0
0	0	1	0	0	0	0
0	0	0	0	0	0	0
0	1	1	0	0	0	0
0	3	0	0	0	0	0
0	0	0	1	0	0	0
0	0	0	0	0	0	0
0	0	0	0	0	0	0
0	1	0	0	0	0	0
0	0	0	0	0	0	0
0	1	0	0	0	0	0
0	1	1	0	0	0	0
0	0	1	0	0	0	0
0	0	0	0	0	0	0
0	0	0	0	0	0	0
0	1	3	0	0	0	0
0	3	1	1	0	0	0
0	1	0	0	0	0	0
0	0	0	0	0	0	0
0	0	0	0	0	0	0
0	0	0	0	0	0	0
0	1	0	0	0	0	0
0	0	0	0	0	0	0
0	0	1	0	0	0	0
0	1	2	0	0	0	0
0	0	0	0	0	0	0
0	0	1	0	0	0	0
0	0	0	0	0	0	0
0	2	4	0	0	0	0
0	0	0	0	0	0	0
0	0	0	0	0	0	0
0	0	0	0	0	0	0
0	0	0	0	0	0	0
0	0	0	0	0	0	0

0	6	0	0	0	0	0
0	1	1	0	0	0	0
0	0	0	0	0	0	0
0	0	0	0	0	0	0
0	0	0	0	0	0	0
0	0	0	0	0	0	0
0	0	1	0	1	0	0
0	0	0	0	0	0	0
0	0	0	0	0	0	0
0	3	0	0	0	0	0
0	0	0	0	0	0	0
0	3	0	0	0	0	0
0	1	0	0	0	0	0
0	0	0	0	0	0	0
0	1	5	0	0	0	0
0	0	2	2	0	0	0
0	1	0	0	0	0	0
0	1	0	0	0	0	0
0	4	1	0	0	0	0
0	3	0	0	0	0	0
0	0	1	0	0	0	0
0	0	0	0	0	0	0
0	0	1	0	0	0	0
0	1	0	0	0	0	0
0	0	0	0	0	0	0
0	0	0	0	0	0	0
0	0	0	0	0	0	0
0	0	0	0	0	0	0
0	0	0	0	0	0	0
0	0	0	0	0	0	0
0	0	0	0	0	0	0
0	0	0	0	0	0	0

0	0	0	0	0	0	0
0	0	1	0	0	0	0
0	0	1	0	0	0	0
0	0	0	0	0	0	0
0	0	1	0	0	0	0
0	0	0	0	0	0	0
0	0	0	0	0	0	0
0	1	0	0	0	0	0
0	0	2	0	0	0	0
0	0	0	0	0	0	0
0	0	0	0	0	0	0
0	4	9	0	0	0	0
0	0	1	0	0	0	0
0	0	1	0	1	0	0
0	19	0	0	0	0	0
0	4	1	0	0	0	0
0	0	1	2	0	0	0
0	0	1	0	0	0	0
0	0	0	0	0	0	0
0	0	0	0	0	0	0

0	74	0	0	0	0	0
0	6	0	0	0	0	0
0	1	0	0	0	0	0
0	12	0	0	0	0	0
0	0	0	0	0	0	0
0	0	0	0	0	0	0
0	0	0	0	0	0	0
0	0	0	0	0	0	0
0	2	0	0	0	0	0
0	4	2	0	0	0	0
0	2	0	0	0	0	0
0	0	0	0	0	0	0
0	0	0	0	0	0	0
0	3	0	0	0	0	0
0	1	1	0	0	0	0
0	0	0	0	0	0	0
0	6	0	0	0	0	0
0	1	3	0	0	0	0
0	0	2	0	0	0	0
0	0	0	0	0	0	0
0	0	0	0	0	0	0
0	3	0	0	0	0	0
0	3	0	0	0	0	0
0	3	1	0	0	0	0
0	0	0	1	0	0	0
0	0	0	0	0	0	0
0	0	0	0	0	0	0
0	0	0	0	0	0	0

0	0	0	1	0	0	0
0	0	0	1	0	0	0
0	0	0	0	0	0	0
0	1	2	0	0	0	0
0	4	0	0	0	0	0
0	2	0	0	0	0	0
0	1	0	0	0	0	0
0	0	0	0	0	0	0
0	2	0	0	0	0	0
0	2	0	0	0	0	0
0	0	1	1	0	0	0
0	1	1	0	0	0	0
0	0	0	0	0	0	0
0	1	7	0	0	0	0
0	1	0	0	0	0	0
0	2	0	1	0	0	0
0	0	0	0	0	0	0
0	0	0	0	0	0	0
0	9	0	1	0	0	0
0	0	0	0	0	0	0
0	0	1	0	0	0	0
0	45	0	2	0	0	0
0	1	0	0	0	0	0
0	1	0	0	0	0	0
0	1	0	2	0	0	0
0	1	1	0	0	0	0
0	1	0	0	0	0	0

0	1	0	0	0	0	0
1	4	0	0	0	0	0
0	0	0	0	0	0	0
0	1	1	0	0	0	0
0	0	0	0	0	1	0
0	0	0	0	0	0	1
0	0	1	0	0	0	0
0	0	0	0	0	0	0
0	0	1	0	0	0	0
0	0	0	0	0	0	0
0	1	1	0	0	0	0
0	0	0	1	0	0	0
0	0	0	0	0	0	0
0	2	1	0	0	0	0
0	0	0	0	0	0	0
0	0	1	0	0	0	0
0	0	0	1	0	0	0
0	1	1	0	0	0	0
0	0	2	0	0	0	0
0	1	0	0	0	0	0
0	1	0	1	0	0	0
0	0	1	0	0	1	0
0	3	0	0	0	0	0
0	1	1	1	0	0	0
0	0	2	0	0	0	0
0	3	0	1	0	0	0
0	9	0	0	0	0	0
0	0	0	0	0	0	0
0	0	0	0	0	0	0
0	0	0	0	0	0	0
0	0	0	0	0	0	0
0	0	0	0	0	0	0
0	4	0	0	0	0	0
0	0	1	0	0	0	0
0	0	0	0	0	0	0
0	0	0	0	0	0	0
0	0	0	0	0	0	0
0	0	0	0	0	0	0
0	0	0	0	0	0	0
0	1	0	1	0	0	0
0	2	0	0	0	0	0
0	1	2	0	0	0	0
0	76	2	0	0	0	0
0	2	1	0	0	0	0
0	30	4	2	0	0	0
0	0	4	0	0	0	0
0	0	0	0	0	0	0
0	15	1	2	0	0	0
0	3	0	1	0	0	0
0	2	3	7	0	0	0
0	3	2	0	0	0	0
0	1	2	1	0	0	0

acremo_coul	fusa_coul	rhizo_coul	mucor_coul	geotri_coul	trichoderm_coul	paecilo_coul	autres_coul
0	0	0	0	0	0	0	0
0	0	0	0	0	0	0	0
0	0	1000	0	0	0	0	0
0	0	1000	0	0	0	0	0
0	0	1000	0	0	0	0	0
0	0	0	0	0	0	0	0
0	0	1000	0	0	0	0	0
0	0	0	0	0	0	0	0
0	0	0	0	0	0	0	0
0	0	1000	0	0	0	0	0
0	0	0	0	0	0	0	0
0	0	0	0	0	0	0	0
0	0	0	0	0	0	0	0
0	0	0	0	0	0	0	0
0	0	0	0	0	0	0	0
0	0	0	0	0	0	0	0
0	0	0	1	0	0	2	0
0	0	0	0	0	0	0	0
0	0	0	0	0	0	0	0
0	0	0	0	0	0	0	0
0	0	0	0	0	0	0	0
0	0	0	0	0	0	0	0
0	0	0	0	0	0	0	0
0	0	0	0	0	0	0	0
0	0	0	0	0	0	0	0
0	0	0	0	0	0	0	0
0	0	0	0	0	0	0	0
0	0	0	0	0	0	0	0
0	0	0	0	0	0	0	0
0	0	0	1	0	0	0	0
0	0	1000	0	0	0	0	0
0	0	1000	0	0	0	0	0
0	0	0	0	0	0	0	0
0	0	0	0	0	0	0	0
0	0	0	0	0	0	0	0
0	0	0	0	0	0	0	0
0	0	0	0	0	0	0	0
0	0	0	0	0	0	0	0
0	0	0	0	0	0	0	0
0	0	0	0	0	0	0	0
0	0	0	0	0	0	0	0
0	0	0	0	0	0	0	0
0	0	0	0	0	0	0	0
0	0	0	0	0	0	0	0
0	0	0	0	0	0	0	0
0	0	0	0	0	0	0	0
0	0	0	1	0	0	0	1
0	0	0	0	0	0	0	0
0	0	0	0	0	0	0	0
0	0	0	0	0	0	0	0
0	0	0	0	0	0	0	0
0	0	0	0	0	0	0	0
0	0	0	0	0	0	0	0
0	0	0	0	0	0	0	0
0	0	0	0	0	0	0	0

0	0	0	0	0	0	0	0
0	0	0	0	0	0	0	0
0	0	0	0	0	0	0	0
0	0	0	0	0	0	0	0
0	0	0	0	0	0	0	0
0	0	0	0	0	0	0	0
0	0	0	0	0	0	0	0
0	0	0	0	0	0	0	0
0	0	0	0	0	0	0	0
0	0	0	0	0	0	0	0
0	0	0	0	0	0	0	0
0	0	0	0	0	0	0	0
0	0	0	0	0	0	0	0
0	0	0	0	0	0	0	0
0	0	0	0	0	0	0	0
0	0	0	0	0	0	0	0
0	0	0	0	0	0	0	0
0	0	0	0	0	0	0	0
0	0	0	0	0	0	0	0
0	0	0	0	0	0	0	0
0	0	0	0	0	0	0	0
0	0	0	0	0	0	0	0
0	0	0	0	0	0	0	0
0	0	1000	0	0	0	0	0
0	0	0	0	0	0	0	0
0	0	0	0	0	0	0	0
0	0	0	0	0	0	0	0
0	0	0	0	0	0	0	0
0	0	0	0	0	0	0	0
0	0	0	0	0	0	0	0
0	0	0	0	0	0	0	0
0	0	0	0	0	0	1	0
0	0	0	0	0	0	0	0
0	0	0	0	0	0	0	0
0	0	0	0	0	0	0	0
0	0	0	0	0	0	0	0

0	0	0	0	0	0	0	0
0	0	0	0	0	0	0	0
0	0	0	0	0	0	0	0
0	0	0	0	0	0	0	0
0	0	0	0	0	0	0	0
0	0	0	0	0	0	0	0
0	0	0	0	0	0	0	0
0	0	0	0	0	0	0	0
0	0	0	0	0	0	0	0
0	0	0	0	0	0	0	0
0	0	0	0	0	0	0	0
0	0	0	0	0	0	0	0
0	0	0	0	0	0	0	0
0	0	0	0	0	0	0	0
0	0	0	0	0	0	0	0
0	0	0	0	0	0	0	0
0	0	0	0	0	0	0	0
0	0	0	0	0	0	0	0
0	0	0	0	0	0	0	0

0	0	0	0	0	0	0	0
0	0	0	0	0	0	0	0
0	0	0	0	0	0	0	0
0	0	0	0	0	0	0	0
0	0	0	0	0	0	0	0
0	0	1000	0	0	0	0	0
0	0	0	0	0	0	0	0
0	0	1000	0	0	0	0	0
0	0	0	0	0	0	0	0
0	0	0	0	0	0	0	0
0	0	1000	0	0	0	0	0
0	0	1000	0	0	0	0	0
0	0	0	0	0	0	0	0
0	0	0	0	0	0	0	0
0	0	0	0	0	0	0	0
0	0	0	0	0	0	0	0
0	0	1000	0	0	0	0	14
0	0	0	0	0	0	0	0
0	0	0	0	0	0	0	0
0	0	0	0	0	0	0	0
0	0	0	0	0	1	0	0
0	0	0	0	0	0	0	0
0	0	0	0	0	0	0	0
0	0	0	0	0	0	0	0
0	0	0	0	0	0	0	0
0	0	0	0	0	0	0	0
0	0	0	0	0	0	0	0
0	0	0	0	0	0	0	0
0	0	0	0	0	0	0	0
0	0	0	0	0	0	0	0
0	0	0	0	0	0	0	0
0	0	0	0	0	0	0	0
0	0	0	0	1	0	0	0
0	0	0	0	0	0	0	0
0	0	0	0	0	0	0	0

0	0	0	0	0	0	0	0
0	0	0	0	0	0	0	0
0	0	0	0	0	0	0	0
0	0	0	0	0	0	0	0
3	0	0	0	0	0	0	0
0	0	0	0	0	0	0	0
0	0	0	0	0	0	0	0
0	0	0	0	0	0	0	0
0	0	0	0	0	0	0	0
1	0	0	0	0	0	0	0
0	0	0	0	0	0	1	0
0	0	1000	0	0	0	0	0
0	0	0	0	0	0	0	0
0	0	0	0	0	0	0	0

0	0	0	0	0	0	0	0
0	0	0	0	0	0	0	0
0	0	0	0	0	0	0	0
0	0	0	0	0	1	0	0
0	0	0	0	0	0	0	0

0	0	0	0	0	0	3	0
0	0	0	0	0	0	0	0
0	0	0	0	0	0	0	0
0	0	0	0	0	0	1	0
0	0	0	0	0	0	0	1
0	0	0	0	0	1	0	0
0	0	0	0	0	0	0	1
0	0	0	0	0	0	0	0
0	0	0	0	0	0	0	0
0	0	0	0	0	0	0	0
0	0	0	0	0	0	0	0
0	1	0	0	0	0	0	0
0	0	0	0	0	0	0	0
0	0	0	0	0	0	0	0

0	0	0	0	0	0	0	0
0	0	0	0	0	0	0	0
0	0	0	0	0	0	0	0
0	0	0	0	0	0	0	0
0	0	0	0	0	0	0	0
0	0	0	0	0	0	0	0
0	0	0	0	0	0	0	0
0	0	0	0	0	0	0	0
0	0	0	0	0	0	0	0

0	0	0	0	0	0	0	0
0	0	0	0	0	0	0	0
0	0	0	0	0	0	0	0
0	0	0	0	0	0	0	0
0	0	0	0	0	0	0	0
0	0	0	0	0	0	0	0
0	0	0	0	0	0	0	0
0	0	0	0	0	0	0	0
0	0	0	0	0	0	2	0
0	0	0	0	0	0	0	0
0	0	0	0	0	0	0	0
0	0	0	0	0	0	0	1
0	0	0	0	0	0	0	0
0	0	0	0	0	0	1	0
0	0	0	0	0	0	0	0
0	0	1000	0	0	0	0	0
0	0	1000	0	0	0	0	0
0	0	0	0	0	0	0	0
0	0	0	1	0	0	0	0
0	0	0	0	0	0	0	0
0	0	0	0	0	0	53	0
0	0	0	0	0	0	0	1
0	0	0	0	0	0	0	0
0	0	0	1	0	0	0	0
0	0	0	0	0	0	1	0
0	0	0	0	0	0	0	0
0	0	0	0	0	0	0	0
0	0	0	0	0	0	0	0
0	0	0	0	0	0	0	1

0	0	0	0	0	0	0	0
0	0	0	0	0	0	0	0
0	0	0	0	0	0	0	0
0	0	0	0	0	0	0	0
0	0	0	0	0	0	0	0
0	0	1000	0	0	0	0	0
0	0	0	0	0	0	0	0
0	0	1000	0	0	0	0	2
0	0	0	0	0	0	0	0
0	0	0	0	0	0	0	0
0	1	0	0	0	0	0	0
0	0	0	0	0	0	0	0
0	0	0	0	0	0	0	0
0	0	0	0	0	0	0	0
0	0	0	0	0	0	1	1
0	0	0	0	0	0	0	0
0	0	0	0	0	0	0	0
0	0	0	0	0	0	0	0
0	0	0	0	0	0	0	0
0	0	0	0	0	0	2	0
0	0	0	0	0	0	0	0
0	0	0	0	0	0	0	1
0	0	0	0	0	0	0	0
0	0	0	0	0	0	0	0
0	0	0	0	0	0	0	0

0	0	0	0	0	0	0	0
0	0	0	0	0	0	0	0
0	0	0	0	0	0	0	0
0	0	0	0	0	0	0	0
0	0	0	0	0	0	0	0
0	0	0	0	0	0	0	0
0	0	0	0	0	0	0	0
0	0	0	0	0	0	0	0
0	0	0	0	0	0	0	0
0	0	0	0	0	0	0	0
0	0	0	0	0	0	0	0
0	0	0	0	0	0	0	0
0	0	0	0	0	0	0	0
0	0	0	0	0	0	0	0
0	0	0	0	0	0	0	0
0	0	0	0	0	0	0	0
0	0	0	0	0	0	0	0
0	0	0	0	0	0	0	0
0	0	0	0	0	0	0	0
0	0	0	0	0	0	0	0
0	0	0	0	0	0	0	0
0	0	0	0	0	0	0	0
0	0	0	0	0	0	0	0
0	0	0	0	0	0	0	0

0	0	0	0	0	0	0	0
0	0	0	0	0	0	0	0
0	0	0	0	0	0	0	0
0	0	0	0	0	0	0	0
0	0	0	0	0	0	0	0

0	0	0	0	0	0	0	0
0	0	0	0	0	0	0	0
0	0	0	0	0	0	0	0
0	0	0	0	0	0	0	0
0	0	0	0	0	0	0	0
0	0	0	0	0	0	0	0
0	0	0	0	0	0	0	0
0	0	0	0	0	0	0	0
0	1	0	0	0	0	0	0
0	0	0	0	0	0	0	0
0	0	0	0	0	0	1	0
0	0	0	0	0	0	0	0
0	0	0	0	0	0	0	0
0	0	0	0	0	0	0	0
0	0	0	0	0	0	0	0
0	0	0	0	0	0	0	0
0	0	1	0	0	0	0	0
0	0	0	0	0	0	0	0
0	0	0	0	0	0	0	0
0	0	0	0	0	0	0	0
0	0	0	0	0	0	0	1
0	0	0	0	0	0	0	0
0	0	0	0	0	0	1	0
0	0	0	0	0	0	0	0
0	0	0	0	0	0	0	0
0	0	0	0	0	0	0	0
0	0	0	0	0	0	0	0
0	0	0	0	0	0	0	0
0	0	0	0	0	0	0	0
0	0	0	0	0	0	0	0
0	0	0	0	0	0	0	0
0	0	0	0	0	0	0	0

0	0	0	0	0	0	0	0
0	0	0	0	0	0	0	0
0	0	0	0	0	0	0	0
0	0	0	0	0	0	0	0
0	0	0	0	0	0	0	0
0	0	0	0	0	0	0	0
0	0	0	0	0	0	0	0
0	0	0	0	0	0	0	0
0	0	0	0	0	0	0	0
0	0	0	0	0	0	0	0
0	0	0	0	0	0	0	0
0	0	0	0	0	0	0	0
0	0	0	0	0	0	0	0
0	0	0	0	0	0	0	0
0	0	0	0	0	0	0	0
0	0	0	0	0	0	0	0
0	0	0	0	0	0	0	0
1	0	0	0	0	0	0	0
0	0	0	0	0	0	0	0
0	0	0	0	0	0	0	0

0	0	0	0	0	0	0	0
0	0	0	0	0	0	0	0
0	0	0	0	0	0	0	0
0	0	0	0	0	0	0	0
0	0	1000	0	0	0	0	0
0	0	1000	0	0	0	0	0
0	0	0	0	0	0	0	0
0	0	0	0	0	0	0	0
0	0	0	0	0	0	0	0
0	0	0	0	0	0	0	0
1	0	0	0	0	0	0	0
0	0	0	0	0	0	1	0
0	0	0	0	0	0	0	0
0	0	0	0	0	0	0	0
0	0	0	0	0	0	0	0
0	0	0	0	0	0	0	0
0	0	0	0	0	0	0	0
0	0	0	0	0	0	0	0
0	0	0	0	0	0	0	0
0	0	0	0	0	0	0	0
0	0	0	0	0	0	1	0
0	0	0	0	0	0	0	0
0	0	0	0	0	0	0	0
0	0	0	0	0	0	0	0
0	0	0	0	0	0	0	0
0	0	0	0	0	0	0	0
0	0	0	0	0	0	0	0
0	0	0	0	0	0	0	0

0	0	0	0	0	0	0	0
0	0	0	0	0	0	0	0
0	0	0	0	0	0	0	0
0	0	0	0	0	0	0	0
0	0	0	0	0	0	0	0
0	0	0	0	0	0	0	1
0	0	0	0	0	0	0	0
0	0	0	0	0	1	0	0
0	0	0	0	0	0	0	0
0	0	0	0	0	0	0	1
0	0	0	0	0	0	0	0
0	0	0	0	0	0	0	0
0	0	0	0	0	0	0	0
0	0	0	0	0	0	0	0
0	0	0	0	0	0	0	0
0	0	0	0	0	0	1	0
0	0	0	0	0	0	0	0
0	0	0	0	0	0	0	0
0	0	0	0	0	0	0	2
0	0	0	0	0	0	0	0
0	0	0	0	0	0	0	0
0	0	0	0	0	0	0	0
0	0	0	0	0	0	0	0
0	0	0	0	0	0	0	0
0	0	0	0	0	0	0	0
0	0	0	0	0	0	0	0
0	0	0	1	0	0	0	0

0	0	0	0	0	0	0	0
0	0	0	0	0	0	0	0
0	0	0	0	0	0	0	0
0	0	0	0	0	0	0	0
0	0	0	0	0	0	0	0
0	0	0	0	0	0	0	0
0	0	0	0	0	0	0	0
0	0	0	0	0	0	0	0
0	0	0	0	0	0	0	0
0	0	0	0	0	0	0	0
0	0	0	0	0	0	0	0
0	0	0	0	0	0	0	0
0	0	0	0	0	0	0	0
0	0	0	0	0	0	0	0
0	0	0	0	0	0	0	0
0	0	0	0	0	0	0	0
0	0	0	0	0	0	0	0
0	0	0	0	0	0	0	0
0	0	0	0	0	0	0	0
0	0	0	0	0	0	0	0
0	0	0	0	0	0	0	0
0	0	0	0	0	0	0	0
0	0	0	0	0	0	1	0
0	0	0	0	0	0	0	0
0	0	0	0	0	0	0	0
0	0	0	0	0	0	0	0
0	0	0	0	0	0	0	0
0	0	0	0	0	0	0	0
0	0	0	0	0	0	0	0
0	0	1000	0	0	0	0	0
0	0	0	0	0	0	0	0
0	0	1000	0	0	0	0	0
0	0	0	0	0	0	0	0
0	0	0	0	0	0	0	0
0	0	0	0	0	0	1	0
0	0	0	0	0	0	0	0
0	0	0	0	0	0	0	0
0	0	0	0	0	0	0	0
0	0	0	0	0	0	0	0
0	0	0	0	0	0	0	0
0	0	0	0	0	0	0	0
0	0	0	0	0	0	0	0
0	0	1	0	0	0	0	0
0	0	0	0	0	0	0	1
0	0	0	0	0	0	0	0
0	0	0	0	0	0	0	0
0	0	0	0	0	0	0	0
0	0	0	0	0	0	0	0
0	0	0	0	0	0	0	0
0	0	0	0	0	0	0	0
0	0	0	0	0	0	0	0
0	0	0	0	0	0	0	0

csd_coul	levure_coul	asp_fumi_ps	sct_nid_ps	asp_nid_ps	asp_ustus_ps	asp_niger_ps	asp_sp_ps
1	0	0	0	0	0	0	0
0	1	0	0	0	0	0	0
0	0	0	0	0	0	0	0
0	0	0	0	0	1	0	0
0	0	0	0	0	0	0	0
0	2	3	0	0	0	0	0
0	0	1	0	0	0	1	0
0	0	0	0	0	0	0	0
1	1	0	0	0	0	0	0
0	14	0	0	0	0	0	0
3	0	0	0	0	0	0	0
2	0	0	0	0	0	0	0
1	0	0	0	0	0	0	0
2	0	0	0	0	0	0	1
0	0	0	0	0	0	0	0
0	1	0	0	0	0	0	0
0	0	0	0	0	0	0	2
1	3	0	0	0	0	0	0
0	0	0	0	0	0	0	0
0	0	0	0	0	0	0	0
0	0	0	0	0	0	0	1
0	1	0	0	0	0	0	0
1	0	0	0	0	0	0	0
2	0	0	1	0	0	0	4
0	1	0	0	0	0	0	0
1	0	0	3	0	0	0	0
1	0	0	0	0	0	0	0
0	0	0	0	0	0	0	0
0	0	0	0	0	0	0	0
9	0	0	0	0	0	0	0
0	0	0	0	0	0	0	0
0	0	0	0	0	0	0	0
0	0	3	0	0	0	2	0
0	0	1	0	0	0	0	0
0	0	0	0	0	0	0	0
0	0	0	0	0	0	0	0
0	2	0	0	0	0	0	0
0	1	0	36	0	5	0	0
0	0	0	0	0	0	0	0
1	1	0	0	0	0	0	0
3	2	0	0	0	0	0	3
3	3	0	0	0	0	0	0
1	1	0	0	0	0	0	0
3	2	1	0	0	0	0	0
1	0	0	1	0	0	1	0
1	0	0	0	0	0	0	1
1	23	0	0	0	0	0	0
5	0	0	0	0	0	0	0
2	1	0	0	0	0	0	2
2	1	0	0	0	0	0	0
1	0	0	0	0	0	0	0
0	1	0	0	0	0	0	0
0	0	0	0	0	0	0	0
1	0	0	0	0	0	0	0
0	0	0	0	0	0	0	0

0	0	0	0	0	0	0	0
2	0	0	0	0	0	0	0
0	0	0	0	0	0	0	0
1	0	0	0	0	0	0	0
4	0	0	0	0	0	0	0
0	7	0	0	0	0	0	0
5	3	2	1	0	0	0	0
3	1	0	0	0	0	0	0
4	1	0	0	0	0	0	0
2	0	0	0	0	0	0	0
0	0	1	0	0	0	0	0
0	0	0	0	0	1	0	0
1	3	0	0	0	0	0	2
0	0	0	0	0	0	0	0
1	2	0	0	0	0	0	0
0	0	1	0	0	0	0	1
2	0	0	1	0	0	0	0
0	0	0	0	0	0	0	0
1	0	3	0	0	0	2	0
0	0	1	0	0	0	0	0
0	4	0	1	0	0	0	0
0	0	0	0	0	0	0	0
0	1	0	0	0	0	0	0
0	0	1	2	0	0	0	0
1	2	0	0	0	0	0	0
0	0	0	0	0	0	0	0
2	0	0	0	0	0	0	0
0	0	0	10	0	0	0	0
2	0	0	0	0	0	0	0
0	0	0	0	0	0	0	0
1	0	0	0	0	0	0	0
3	0	1	0	0	0	2	0
0	0	0	0	0	0	0	0
1	0	0	0	0	0	0	0
1	0	0	0	0	0	0	0
0	0	0	0	0	0	0	0

1	1	0	0	0	1	0	6
0	0	0	0	0	0	0	0
0	0	0	0	0	0	1	0
1	0	0	0	0	0	0	0
6	0	0	0	0	0	0	0
0	0	1	0	0	0	0	0
1	0	0	1	0	0	1	0
3	3	0	0	0	0	0	1
3	1	0	0	0	0	0	0
0	0	0	0	0	0	0	0
1	0	0	0	0	0	0	2
4	2	0	0	0	0	0	0
2	0	0	0	0	0	0	0
0	0	0	0	0	0	0	0
0	0	0	4	0	0	0	0
2	2	0	1	0	0	0	0
0	1	1	0	0	0	0	1
6	0	0	0	0	0	0	0
0	0	0	0	0	0	0	0

0	0	0	0	0	0	0	0
1	0	0	0	0	0	0	0
0	0	0	0	0	0	0	0
0	0	0	0	0	0	0	0
0	0	0	0	0	0	0	0
0	0	0	0	0	0	0	0
1	1	0	0	0	1	0	0
0	0	0	1	0	0	0	0
0	0	0	0	0	0	0	2
0	1	1	0	0	0	0	0
0	0	0	0	0	0	0	0
0	0	0	0	0	0	0	0
1	0	1	0	0	0	0	0
0	0	0	0	0	0	0	0
0	0	0	0	0	0	0	0
1	0	0	0	0	0	0	0
0	0	0	0	0	0	0	3
0	0	0	4	0	0	0	0
0	0	0	0	0	0	0	1
1	5	1	0	1	0	1	0
0	0	1	0	0	0	0	0
1	1	0	1	0	0	0	0
0	0	0	0	0	0	0	0
1	1	0	0	0	0	0	0
1	1	1	2	0	0	0	0
0	0	0	0	0	0	0	0
0	0	0	0	0	0	0	0
1	0	1	0	0	0	0	0
3	0	0	0	0	0	0	0
0	0	0	0	0	0	0	0
2	1	0	0	0	0	1	0
0	0	0	0	0	1	0	0
1	1	0	2	0	0	0	0
0	0	0	0	0	0	0	0
2	0	2	0	0	0	0	0
0	0	0	0	0	0	0	0
1	0	0	0	0	0	0	0
0	0	0	0	0	0	0	0
0	5	0	0	0	0	0	0
0	2	1	0	0	0	2	0
4	0	0	0	0	0	0	0
0	0	0	0	0	0	0	1
0	0	0	0	0	0	0	0
0	0	0	0	0	0	0	1
0	0	0	1	0	0	0	0
0	1	0	0	0	0	0	1
0	3	1	0	1	0	1	0
0	0	1	0	0	0	0	0
0	1	0	1	0	0	0	0
0	0	1	0	0	0	0	0
0	0	0	0	0	0	0	0
9	2	0	0	0	0	0	0
0	0	0	0	0	0	0	0
0	0	0	0	0	0	0	0

1	3	0	0	0	0	0	0
0	1	0	10	0	0	0	0
0	0	0	0	0	0	0	0
2	0	0	0	0	0	0	0
1	0	0	0	0	0	0	0
3	0	0	0	0	0	0	0
0	0	0	0	0	0	0	1
6	0	0	0	0	0	0	0
1	1	0	0	0	0	0	0
0	0	0	0	0	0	0	0
0	0	0	0	0	0	0	0
2	1	0	0	0	0	0	1
3	0	0	0	0	0	0	0
2	1	0	0	0	0	0	0
1	0	0	0	0	1	0	6
2	0	0	0	0	0	0	0
3	2	0	0	0	0	1	0
13	2	0	0	0	0	0	0
4	1	0	0	0	0	0	0
2	0	1	0	0	0	0	0
1	0	0	1	0	0	1	0
2	0	0	0	0	0	0	1
0	9	0	0	0	0	0	0
0	0	0	0	0	0	0	0
0	0	0	0	0	0	0	0
0	0	0	0	0	0	0	0
0	1	0	0	0	0	0	0
6	1	0	0	0	0	0	0
0	1	0	0	0	0	0	0
0	0	0	0	0	0	0	0
0	0	0	0	0	0	0	1
0	2	0	0	0	0	0	0
0	0	0	0	0	0	0	0
0	0	0	1	0	0	0	4
0	9	0	0	0	0	0	0
0	0	0	3	0	0	0	0
4	10	0	1	0	0	0	0
0	0	1	0	0	0	0	0
0	0	0	0	0	0	0	0
0	0	0	0	0	0	0	0
0	0	0	0	0	0	0	0
0	0	0	0	0	0	0	0
2	1	0	0	0	0	0	1
0	2	0	0	0	0	0	0
0	1	0	0	0	0	0	0
0	0	0	0	0	0	0	0
0	0	1	0	1	0	1	0
1	0	1	0	0	0	0	0
2	2	0	1	0	0	0	0
0	0	0	0	0	0	0	0
0	0	0	0	0	0	0	0
0	0	1	2	0	0	0	0

0	0	0	0	0	0	0	0
0	0	1	0	2	0	0	0
0	0	0	0	0	0	0	0
1	3	0	0	0	0	0	0
3	3	0	0	0	0	0	0
0	0	0	0	0	0	0	0
1	0	0	0	0	0	0	0
0	0	0	0	0	0	0	0
1	0	1	0	0	0	0	1
0	0	0	0	1	0	0	0
0	1	0	0	0	0	0	0
3	3	1	0	1	0	1	0
1	0	0	0	0	0	0	0
0	0	0	0	0	0	0	0
0	0	0	0	0	0	0	0
1	1	0	0	0	0	0	0
0	0	1	2	0	0	0	0
0	0	0	0	0	0	0	0
1	0	0	0	0	0	0	0
1	1	0	0	0	0	0	0
2	0	0	10	0	0	0	0
0	0	0	0	0	0	0	0
0	0	0	0	0	0	0	0
2	0	0	0	0	0	0	0
0	0	0	1	0	0	0	0

0	2	3	0	0	0	2	0
0	0	1	0	0	0	0	0
0	0	0	0	0	0	0	0
0	0	1	0	0	0	0	0
0	0	0	0	0	0	0	0
0	0	0	0	0	0	0	0
0	1	0	0	0	0	0	0
0	0	0	0	0	0	0	0
0	0	0	0	0	0	0	0
1	0	0	0	0	0	0	0
0	0	0	0	0	0	0	0
0	0	0	0	0	0	0	0
0	2	0	0	0	0	0	0
0	1	3	0	0	0	2	0
0	0	1	0	0	0	0	0
0	1	0	0	0	0	0	0
0	0	1	0	0	0	0	0
0	0	0	0	0	0	0	0
0	0	0	0	0	0	0	0
0	0	0	0	0	0	0	0
2	0	0	0	0	0	0	0
1	0	0	0	0	0	0	0
0	1	0	10	0	0	0	0
0	0	0	0	0	0	0	0

0	1	0	1	0	0	0	0
0	0	0	0	0	0	0	0
0	0	0	0	0	0	0	0
1	0	0	0	0	0	0	0
0	0	0	1	0	0	0	0

0	0	1	3	0	0	0	0
1	0	0	0	0	0	0	0
0	0	0	0	0	0	0	0
0	0	0	0	0	0	0	0
1	0	0	0	0	0	0	0
0	0	0	0	0	0	0	0
0	0	0	0	0	0	0	0
0	0	0	0	0	0	1	0
0	0	0	0	0	0	0	0
0	0	0	2	0	0	0	0
0	0	0	0	0	0	0	0
0	5	5	0	0	0	0	0
2	1	0	0	0	0	0	0
0	0	0	0	0	0	0	0
2	0	0	0	0	0	1	0
3	0	0	0	0	0	0	0
1	0	0	0	0	0	0	0
0	0	1	0	0	0	0	0
3	0	0	1	0	0	1	0
0	0	0	0	0	0	0	0
0	0	0	0	0	0	0	0
0	0	0	0	0	0	0	0
0	0	0	0	0	0	0	0
0	0	0	1	0	0	0	0
31	1	0	0	0	1	0	0
0	0	0	1	0	0	0	0
1	0	0	0	0	1	0	0
0	0	0	0	0	0	0	0
0	0	0	0	0	0	0	0
0	0	0	0	0	0	0	0
0	0	0	0	0	0	0	0
0	0	0	0	0	0	0	0

0	0	0	0	0	0	0	0
1	2	0	0	0	0	0	0
3	0	0	0	0	0	0	0
0	0	0	0	0	0	0	0
0	3	0	0	0	0	0	0
0	0	0	0	0	0	0	0
0	1	0	0	0	0	0	0
1	0	0	0	0	0	0	0
0	0	0	0	0	0	0	0
0	0	0	0	0	0	0	0
0	0	0	0	0	0	0	0
0	0	0	0	0	0	0	0
2	0	0	0	0	0	0	0
1	0	0	0	0	0	0	0
0	0	0	0	0	0	0	0
3	0	0	0	0	0	0	0
2	0	0	1	0	0	0	0
0	0	0	0	0	0	0	0
0	0	0	0	0	0	0	0
0	0	0	1	0	0	0	0

12	0	0	7	0	4	0	0
0	1	0	0	0	0	0	3
0	0	0	0	0	0	0	14
1	0	0	3	0	1	0	0
0	0	0	1	0	0	0	0
0	0	1	0	0	0	0	0
1	0	1	0	0	0	1	0
0	0	0	0	0	0	0	0
1	1	0	0	0	0	0	0
1	7	0	0	0	0	0	0
1	1	0	36	0	5	0	0
0	0	0	0	0	0	0	0
0	0	0	0	0	0	0	0
1	1	0	0	0	0	0	3
0	0	0	4	0	0	0	0
0	0	0	0	0	0	0	1
0	0	1	3	0	0	0	0
0	0	0	0	0	0	0	0
0	0	0	0	0	0	0	0
0	0	1	0	0	0	0	0
0	0	0	0	0	0	0	0
1	0	0	1	0	0	0	0
6	3	0	0	0	1	0	0
0	0	0	1	0	0	0	0
1	0	0	0	0	1	0	0
3	1	0	2	0	0	0	0
0	0	0	0	0	0	0	0
0	1	2	0	0	0	0	0
1	0	0	0	0	0	0	0
0	0	0	0	0	0	0	0
0	0	0	1	0	0	0	0
0	0	0	0	0	1	0	0
2	0	0	0	0	0	0	0
0	0	0	0	0	0	0	0
0	0	0	0	0	0	0	0
1	0	1	0	0	0	0	0
0	0	0	0	0	0	0	0
1	1	0	0	0	0	0	0
0	3	0	0	0	0	0	0
0	0	0	0	0	0	0	0
1	1	0	0	0	0	0	0
0	1	0	0	0	0	0	0
3	0	1	0	0	0	0	0
0	1	0	1	0	0	1	0
1	1	0	0	0	0	0	2
0	0	0	0	0	0	0	0
0	0	0	0	0	0	0	0
1	0	1	0	0	0	0	0
0	0	0	0	0	0	0	0
2	0	0	0	0	0	0	0
0	1	0	0	0	0	0	0
1	0	0	0	0	0	0	1
2	2	0	0	0	0	0	0
1	1	0	0	0	0	0	0
1	0	0	1	0	0	0	4

0	0	0	0	0	0	0	0
0	0	0	3	0	0	0	0
0	0	0	0	0	0	0	0
1	0	0	0	0	0	0	0
0	0	0	0	0	0	0	0
0	0	0	0	0	0	0	0
1	6	0	0	0	0	0	0
1	0	0	0	0	0	0	0
0	0	1	0	0	0	0	0
0	0	0	0	0	0	0	0
0	0	0	0	0	0	0	0
0	0	0	0	0	0	0	0
0	0	0	0	0	0	0	0
0	0	0	0	0	0	0	0
1	0	0	0	0	0	0	0
2	0	0	0	0	0	0	0
1	0	0	0	0	0	0	0
0	0	0	0	0	0	0	0
1	0	0	0	0	0	0	0
19	0	0	0	0	0	0	0
2	1	0	0	0	0	0	0
35	0	0	0	0	0	0	0
2	0	0	0	0	0	0	0
12	0	0	0	0	0	0	0
1	0	0	0	0	0	0	0
0	2	0	0	0	0	0	0
2	0	0	0	0	0	0	0
0	0	0	0	0	0	0	0
0	0	1	0	0	0	0	0
0	0	0	0	0	0	0	0
1	0	0	0	0	0	0	0
0	0	0	0	0	0	0	0
0	0	0	0	0	0	0	0
1	0	0	4	0	0	0	0
0	0	0	0	0	0	0	1
3	5	1	1	0	0	1	0
0	1	1	0	0	0	0	0
1	2	0	1	0	0	0	0
0	0	0	0	0	0	0	0
0	0	0	0	0	0	0	0
0	11	0	0	0	0	0	0
0	0	0	0	0	0	0	0
0	0	0	0	0	0	0	2
2	0	0	0	0	0	0	0
0	0	0	0	0	0	0	0
2	0	0	0	0	0	0	0
2	0	0	0	0	0	0	1
0	0	0	0	0	0	0	0
2	1	0	0	0	0	0	0
4	0	0	0	0	2	0	0
3	0	0	1	0	0	0	1
1	0	0	0	0	0	0	0

asp_terreus_ps	asp_flavus_ps	peni_ps	clado_ps	alterna_ps	ulocla_ps	chaeto_ps	scopula_ps
0	0	0	0	0	0	0	0
0	0	3	0	0	0	0	0
0	0	2	0	0	0	0	0
0	0	0	0	0	0	0	0
0	0	0	0	0	0	0	0
0	0	2	0	0	0	0	0
0	0	1	0	0	0	0	0
0	0	0	0	0	0	0	0
0	0	3	0	0	0	0	0
0	0	4	1	0	0	0	0
0	0	1	1	0	0	0	0
0	0	0	0	0	0	0	0
0	0	0	1	1	0	0	0
0	0	1	0	0	0	0	0
0	0	4	0	0	0	0	0
0	0	3	2	0	0	0	0
0	0	90	2	0	0	0	0
0	0	0	0	0	0	0	0
0	0	14	0	0	0	0	0
0	0	0	0	0	0	0	0
0	0	0	0	0	0	0	0
0	0	2	0	0	0	0	0
0	0	2	5	0	0	0	0
0	0	0	1	0	0	0	0
0	0	7	4	0	0	0	0
0	0	0	0	0	0	0	0
0	0	0	0	0	0	0	0
0	0	0	0	0	0	0	0
0	0	0	0	0	0	0	0
0	0	8	0	0	0	0	0
0	0	5	0	0	0	0	0
0	0	0	0	0	0	0	0
0	0	0	0	0	0	0	0
0	0	0	0	0	0	0	0
0	0	0	0	0	0	0	0
0	0	2	0	0	0	0	0
0	0	4	1	0	0	0	0
0	0	1	0	0	0	0	1
0	0	1	0	0	0	0	0
0	0	0	0	0	0	0	0
0	0	9	1	0	0	0	0
0	0	0	0	0	0	0	0
0	0	0	2	0	0	0	0
0	0	1	2	1	0	0	0
0	0	3	0	0	0	0	0
0	0	1	0	0	0	0	0
0	0	4	0	0	0	0	0
0	0	3	2	0	0	0	0
0	0	90	2	0	0	0	0
0	0	0	0	0	0	0	0
0	0	0	0	0	0	0	0
0	0	0	0	1	0	0	0
0	0	3	0	0	0	0	0
0	0	9	1	0	0	0	0
0	0	0	0	0	0	0	0

0	0	10	0	0	0	0	0
0	0	0	0	0	0	0	0
0	0	0	0	0	0	0	0
0	0	1	0	0	0	0	0
0	0	0	2	0	0	0	0
0	0	1	5	2	0	0	0
0	0	0	0	0	0	0	0
0	0	3	0	0	0	0	0
0	0	0	0	0	0	0	0
0	0	0	0	0	0	0	0
0	0	3	0	0	0	0	0
0	0	4	0	0	0	0	0
0	0	2	2	0	0	0	0
0	0	1	1	0	0	0	0
0	0	2	0	0	0	0	0
0	0	0	0	0	0	0	0
0	0	0	0	0	0	0	0
0	0	0	0	0	0	0	0
0	0	0	0	0	0	0	0
0	0	0	1	0	0	0	1
0	0	0	0	0	0	0	0
0	0	0	0	0	0	0	0
0	0	1	1	0	0	0	0
0	0	0	0	0	0	0	0
0	0	0	0	0	0	0	0
0	0	0	0	0	0	0	0
0	0	3	1	0	0	0	0
0	0	1	0	0	0	0	0
0	0	5	7	0	0	0	1
0	0	1	2	0	0	0	0
0	0	1	0	0	0	0	0
0	0	3	20	0	0	0	0
0	0	0	1	0	0	0	0
0	0	0	1	0	0	0	0
0	0	0	0	0	0	0	0
0	0	0	1	0	0	0	0
0	0	4	0	0	0	0	0
0	0	1	0	0	0	0	0
0	0	0	4	1	0	0	0
0	0	1	1	1	0	0	0
0	0	0	2	0	0	0	0
0	0	1	2	1	0	0	0
0	0	3	0	0	0	0	0
0	0	1	0	0	0	0	0
0	0	4	0	0	0	0	0
0	0	3	2	0	0	0	0
0	0	90	2	0	0	0	0
0	0	0	0	0	0	0	0
0	0	14	0	0	0	0	0
0	0	9	1	0	0	0	0
0	0	0	0	1	0	0	0
0	0	0	0	0	0	0	0
0	0	1	1	0	0	0	0
0	0	0	0	0	0	0	0
0	0	0	1	0	0	0	0

0	0	0	0	0	0	0	0
0	0	0	1	0	0	0	0
0	0	0	0	0	0	0	0
0	0	0	0	0	0	0	0
0	0	3	0	0	0	0	0
0	0	0	0	0	0	0	0
0	0	0	0	0	0	0	0
0	0	0	0	0	0	0	0
0	0	0	0	0	0	0	0
0	0	0	0	0	0	0	0
0	0	0	0	0	0	0	0
0	0	2	0	0	0	0	0
0	0	1	0	0	0	0	0
0	0	3	0	0	0	0	0
0	0	0	0	0	0	0	0
0	0	0	2	0	0	0	0
0	0	9	1	0	0	0	0
0	0	0	0	0	0	0	0
0	0	1	0	0	0	0	0
0	0	0	0	0	0	0	0
0	0	0	1	0	0	0	1
0	0	0	0	0	0	0	0
0	0	0	0	0	0	0	0
0	0	1	1	0	0	0	0
0	0	0	0	0	0	0	0
0	0	6	1	0	0	0	0
0	0	1	0	0	0	0	0
0	0	7	2	0	0	0	0
0	0	22	2	2	0	0	0
0	0	0	0	0	0	0	0
0	0	0	0	0	0	0	1
0	0	0	0	2	0	0	0
0	0	0	0	0	0	0	0
0	0	2	0	0	0	0	0
0	0	5	16	5	0	0	0

0	0	0	0	0	0	0	0
0	0	2	0	0	0	0	0
0	0	1	2	0	0	0	0
0	0	1	0	0	0	0	0
0	0	3	20	0	0	0	0
0	0	3	0	0	0	0	0
0	0	2	1	0	0	0	0
0	0	8	0	1	0	0	0
0	0	2	0	0	0	0	0
0	0	0	0	0	0	0	0
0	0	1	0	0	0	0	0
0	0	0	0	0	0	0	0
0	0	0	1	0	0	0	1
0	0	0	0	0	0	0	0

0	0	1	2	0	0	0	0
0	0	0	0	0	0	0	0
0	0	0	0	0	0	0	0
0	0	0	0	0	0	0	0
0	0	0	0	0	0	0	0

0	0	3	1	0	0	0	0
0	0	1	0	0	0	0	0
0	0	5	7	0	0	0	1
0	0	1	2	0	0	0	0
0	0	1	0	0	0	0	0
0	0	1	1	0	0	0	0
0	0	0	0	0	0	0	0
0	0	0	2	0	0	0	0
0	0	0	0	0	0	0	0
0	0	0	0	0	0	0	0
0	0	6	0	0	0	0	0
0	0	0	1	0	0	0	0
0	0	2	0	0	0	0	0
0	0	3	0	0	0	0	0

0	0	4	0	0	0	0	0
0	0	1	0	0	0	0	0
0	0	0	4	1	0	0	0
0	0	1	1	1	0	0	0
0	0	0	2	0	0	0	0
0	0	1	2	1	0	0	0
0	0	3	0	0	0	0	0
0	0	1	0	0	0	0	0
0	0	4	0	0	0	0	0

0	0	0	0	0	0	0	0
0	0	0	1	0	0	0	0
0	0	0	0	0	0	0	0
0	0	0	1	0	0	0	0
0	0	0	0	0	0	0	0
0	0	2	0	0	0	0	0
0	0	1	0	0	0	0	0
0	0	0	0	0	0	0	0
0	0	2	0	0	0	0	0
0	0	2	5	0	0	0	0
0	0	0	1	0	0	0	0
0	0	7	4	0	0	0	0
0	0	0	0	0	0	0	0
0	0	2	0	0	0	0	0
0	0	0	0	0	0	0	0
0	0	8	0	0	0	0	0
0	0	5	0	0	0	0	0
0	0	0	0	0	0	0	0
0	0	0	0	0	0	0	0
0	0	2	0	0	0	0	0
0	0	1	3	0	0	0	0
0	0	0	0	0	0	0	0
0	0	0	0	0	0	0	0
0	0	0	0	0	0	0	0
0	0	0	1	0	0	0	1
0	0	0	0	0	0	0	0
0	0	0	0	0	0	0	0
0	0	1	1	0	0	0	0
0	0	0	0	0	0	0	0

0	0	0	0	0	0	0	0
0	0	0	0	0	0	0	0
0	0	0	0	0	0	0	0
0	0	3	0	0	0	0	0
0	0	4	1	0	0	0	0
0	0	0	0	0	0	0	0
0	0	0	0	0	0	0	0
0	0	2	0	0	0	0	0
0	0	0	0	0	0	0	0
0	0	0	0	0	0	0	0
0	0	0	0	0	0	0	0
0	0	0	0	0	0	0	0
0	0	0	0	0	0	0	0
0	0	0	0	0	0	0	0
0	0	0	0	0	0	0	0
0	0	1	1	0	0	0	0
0	0	0	0	0	0	0	0
0	0	0	0	0	0	0	0
0	0	0	0	0	0	0	0
0	0	3	1	0	0	0	0
0	0	1	0	0	0	0	0
0	0	0	0	0	0	0	0
0	0	0	0	0	0	0	0
0	0	3	0	0	0	0	0
0	0	2	1	0	0	0	0
0	0	0	0	0	0	0	0
0	0	0	0	0	0	0	0
0	0	0	0	0	0	0	0
0	0	1	2	0	0	0	0
0	0	0	0	0	0	0	0
0	0	0	0	0	0	0	0
0	0	0	1	0	0	0	0
0	0	0	0	0	0	0	0
0	0	0	0	0	0	0	0
0	0	0	0	0	0	0	0
0	0	2	0	0	0	0	0
0	0	0	0	0	0	0	0
0	0	0	1	0	0	0	0
0	0	0	0	0	0	0	0
0	0	0	0	0	0	0	0
0	0	0	0	0	0	0	0
0	0	1	2	0	0	0	0
0	0	0	0	0	0	0	0
0	0	0	0	0	0	0	0
0	0	0	1	0	0	0	0
0	0	0	0	0	0	0	0
0	0	3	1	0	0	0	0
0	0	1	0	0	0	0	0
0	0	5	7	0	0	0	1
0	0	8	0	0	0	0	0
0	0	0	0	0	0	0	0
0	0	0	0	0	0	0	0
0	0	0	0	0	0	0	0
0	0	1	0	0	0	0	0

0	0	6	0	0	0	0	0
0	0	1	1	0	0	0	0
0	0	0	0	0	0	0	0
0	0	1	0	0	0	0	0
0	0	0	0	1	0	0	0
0	0	0	0	0	0	0	0
0	0	0	0	0	0	0	0
0	0	0	0	0	0	0	1
0	0	0	0	0	0	0	0
0	0	0	0	0	0	0	0
0	0	2	0	0	0	0	0
0	0	5	16	5	0	0	0
0	0	1	1	0	0	0	0
0	0	0	0	0	0	0	0
0	0	0	4	1	0	0	0
0	0	1	1	1	0	0	0
0	0	0	2	0	0	0	0
0	0	1	2	1	0	0	0
0	0	3	0	0	0	0	0
0	0	1	1	0	0	0	0
0	0	0	0	0	0	0	0
0	0	0	0	0	0	0	0
0	0	3	0	0	0	0	0
0	0	2	1	0	0	0	0
0	0	1	6	0	0	0	0
0	0	8	0	0	0	0	0
0	0	0	0	2	0	0	0
0	0	0	0	0	0	0	0
0	0	0	0	0	0	0	0
0	0	0	0	0	0	0	0
0	0	0	0	0	0	0	0
0	0	0	0	0	0	0	0

0	0	0	0	0	0	0	0
0	0	0	0	0	0	0	0
0	0	0	1	0	0	0	0
0	0	0	0	0	0	0	0
0	0	0	0	0	0	0	0
0	0	1	2	0	0	0	0
0	0	0	0	0	0	0	0
0	0	0	0	0	0	0	0
0	0	2	0	0	0	0	0
0	0	0	0	0	0	0	0
0	0	0	1	0	0	0	0
0	0	1	4	1	0	0	0
0	0	0	0	0	0	0	0
0	0	0	0	0	0	0	0
0	0	0	0	1	0	0	0
0	0	0	3	0	0	0	0
0	0	0	1	0	0	0	0
0	0	0	0	1	0	0	0
0	0	0	0	0	0	0	0
0	0	0	0	0	0	0	0

0	0	53	0	0	0	0	0
0	0	4	0	0	0	0	0
0	0	0	0	0	0	0	0
0	0	3	0	0	0	0	0
0	0	0	0	0	0	0	0
0	0	1	0	0	0	0	0
0	0	1	0	0	0	0	0
0	0	0	0	0	0	0	0
0	0	3	0	0	0	0	0
0	0	4	1	0	0	0	0
0	0	1	0	0	0	0	1
0	0	1	0	0	0	0	0
0	0	0	0	0	0	0	0
0	0	9	1	0	0	0	0
0	0	0	0	0	0	0	0
0	0	1	0	0	0	0	0
0	0	6	0	0	0	0	0
0	0	0	1	0	0	0	0
0	0	0	0	0	0	0	0
0	0	0	0	0	0	0	0
0	0	3	0	0	0	0	0
0	0	2	1	0	0	0	0
0	0	1	6	0	0	0	0
0	0	8	0	0	0	0	0
0	0	0	0	2	0	0	0
0	0	0	0	0	0	0	0
0	0	2	0	0	0	0	0
0	0	5	16	5	0	0	0

0	0	0	0	0	0	0	0
0	0	0	0	0	0	0	0
0	0	2	1	0	0	0	0
0	0	1	6	0	0	0	0
0	0	0	0	0	0	0	0
0	0	0	0	0	0	0	0
0	0	0	0	2	0	0	0
0	0	0	0	0	0	0	0
0	0	0	0	1	0	0	0
0	0	1	1	0	0	0	0
0	0	0	0	0	0	0	0
0	0	0	1	0	0	0	0
0	0	1	1	1	0	0	0
0	0	0	2	0	0	0	0
0	0	1	2	1	0	0	0
0	0	3	0	0	0	0	0
0	0	1	0	0	0	0	0
0	0	1	0	0	0	0	0
0	0	7	0	0	0	0	0
0	0	0	0	0	0	0	0
0	0	0	0	0	0	0	0
0	0	14	0	0	0	0	0
0	0	0	0	0	0	0	0
0	0	0	0	0	0	0	0
0	0	2	0	0	0	0	0
0	0	2	5	0	0	0	0
0	0	0	1	0	0	0	0

0	0	7	4	0	0	0	0
0	0	0	0	0	0	0	0
0	0	0	0	0	0	0	0
0	0	0	0	0	0	0	0
0	0	0	1	0	0	0	0
0	0	0	0	0	0	0	0
0	0	0	1	0	0	0	0
0	0	0	0	0	0	0	0
0	0	1	2	0	0	0	0
0	0	0	0	0	0	0	0
0	0	0	0	0	0	0	0
0	0	0	1	0	0	0	0
0	0	0	0	0	0	0	0
0	0	0	1	0	0	0	0
0	0	0	0	0	0	0	0
0	0	2	0	0	0	0	0
0	0	0	0	0	0	0	0
0	0	0	1	0	0	0	0
0	0	0	2	1	0	0	0
0	0	0	0	0	0	0	0
0	0	0	0	0	0	0	0
0	0	0	0	1	0	0	0
0	0	0	3	0	0	0	0
0	0	0	0	0	0	0	0
0	0	0	0	1	0	0	0
0	0	3	1	0	0	0	0
0	0	9	1	0	0	0	0
0	0	0	0	0	0	0	0
0	0	1	0	0	0	0	0
0	0	0	0	0	0	0	0
0	0	0	0	0	0	0	0
0	0	2	0	0	0	0	0
0	0	3	0	0	0	0	0
0	0	0	0	0	0	0	0
0	0	1	0	0	0	0	0
0	0	0	0	0	0	0	0
0	0	0	1	0	0	0	1
0	0	0	0	0	0	0	0
0	0	0	0	0	0	0	0
0	0	0	0	0	0	0	0
0	0	4	0	0	0	0	0
0	0	3	2	0	0	0	0
0	0	90	2	0	0	0	0
0	0	0	0	0	0	0	0
0	0	14	0	0	0	0	0
0	0	3	0	0	0	0	0
0	0	2	1	0	0	0	0
0	0	8	0	1	0	0	0
0	0	0	3	0	0	0	0
0	0	0	2	5	0	0	0
0	0	0	0	1	0	0	0
0	0	0	0	0	0	0	0

acremo_ps	fusa_ps	rhizo_ps	mucor_ps	geotri_ps	trichoderm_ps	paecilo_ps	autres_ps	csd_ps
0	0	0	0	0	0	0	0	0
0	0	0	0	0	0	0	0	0
0	0	0	0	0	0	0	0	1
0	0	0	0	0	0	0	0	0
0	0	1000	0	0	0	0	0	0
0	0	1000	0	0	0	0	0	0
0	0	0	0	0	0	0	0	0
0	0	1000	0	0	0	0	0	3
0	0	1000	0	0	0	0	0	0
0	0	0	0	0	1	0	0	0
0	0	0	0	0	0	0	0	2
0	0	0	0	0	0	0	0	0
0	0	0	0	0	0	0	0	1
0	0	0	0	0	0	0	0	0
0	0	0	0	0	0	0	0	1
0	0	0	0	0	0	2	0	0
0	0	0	0	0	0	0	0	0
0	0	1	0	0	0	0	0	0
0	0	0	0	0	0	0	0	0
0	0	1000	0	0	0	0	0	0
0	0	0	0	0	0	0	0	0
0	0	0	0	0	0	0	0	0
0	0	0	0	0	0	0	0	0
0	0	0	0	0	0	0	0	0
0	0	0	0	0	0	0	1	1
0	0	0	0	0	0	0	0	3
0	0	0	0	0	0	0	0	0
0	0	0	0	0	0	0	0	1
0	0	0	0	0	0	0	0	0
0	0	1000	0	0	0	0	0	0
0	0	1000	0	0	0	0	0	0
0	0	1000	0	0	0	0	0	0
0	0	1000	0	0	0	0	0	0
0	0	0	0	0	0	0	0	0
0	0	0	0	0	0	0	0	0
0	0	0	0	0	0	0	0	0
0	0	0	0	0	1	0	0	0
0	0	0	0	0	0	0	0	0
0	0	0	0	0	0	0	0	0
0	0	1000	0	0	0	0	1	0
0	0	0	0	0	0	0	0	0
0	0	0	0	0	0	0	0	0
0	0	0	0	0	0	0	0	3
0	0	0	0	0	0	0	0	0
0	0	0	0	0	0	0	0	3
0	0	0	0	0	0	0	0	0
0	0	0	0	0	0	0	0	1
0	0	0	0	0	0	2	0	0
0	0	0	0	0	0	0	0	0
0	0	1	0	0	0	0	0	0
0	0	0	0	0	0	0	0	0
0	0	0	0	0	0	0	0	0
0	0	0	0	0	0	0	0	0
0	0	0	0	0	0	0	0	0
0	0	0	0	0	0	0	0	1

0	0	0	0	0	0	0	0	1
0	0	0	0	0	0	0	0	1
0	0	0	0	0	0	0	0	0
0	0	0	0	0	0	0	0	0
0	0	0	0	0	0	0	0	3
0	0	0	0	0	0	0	0	0
0	0	0	0	0	0	0	0	1
0	0	0	0	0	0	0	0	2
0	0	0	0	0	0	0	0	0
0	0	1000	0	0	0	0	0	0
0	0	0	0	0	0	0	0	1
0	0	0	0	0	0	0	0	1
0	0	0	0	0	0	0	0	0
0	0	0	0	0	0	0	0	1
0	0	0	0	0	0	1	0	1
0	0	0	0	0	0	0	0	0
0	0	0	0	0	0	0	0	0
0	0	0	0	0	0	0	0	0
0	0	1000	0	0	0	0	0	0
0	0	0	0	0	0	1	0	0
0	0	0	0	0	0	0	1	0
0	0	0	0	0	0	0	0	0
0	0	0	0	0	0	0	0	0
0	0	0	0	0	0	1	0	0
0	0	0	0	0	1	0	0	0
0	0	0	0	0	0	0	0	0
0	0	0	0	0	0	3	0	1
0	0	0	0	0	0	0	0	0
0	0	0	0	0	0	0	0	0
0	0	0	0	0	0	0	0	0
0	0	0	0	0	0	0	0	0
0	0	0	0	0	0	0	0	0
0	0	0	0	0	0	0	0	0
0	0	0	0	0	0	0	1	0
0	0	0	0	0	0	0	0	0
0	0	0	0	0	0	0	0	0

0	0	0	0	0	0	0	1	3
0	0	0	0	1	0	0	0	1
0	0	0	0	0	0	1	0	3
0	0	0	0	0	0	0	0	1
0	0	0	0	0	0	0	0	3
0	0	0	0	0	0	0	0	0
0	0	0	0	0	0	0	0	3
0	0	0	0	0	0	0	0	0
0	0	0	0	0	0	0	0	1
0	0	0	0	0	0	2	0	0
0	0	0	0	0	0	0	0	0
0	0	1	0	0	0	0	0	0
0	0	0	0	0	0	0	0	0
0	0	0	0	0	0	0	0	0
0	0	0	0	0	0	0	0	0
0	0	0	0	0	0	0	0	0
0	0	0	0	0	0	0	0	1
0	0	0	0	0	0	1	0	3
0	0	0	0	0	0	0	0	0

0	0	0	0	0	1	0	0	0
0	0	0	0	0	0	0	0	0
0	0	0	0	0	0	0	0	0
0	0	0	0	0	0	0	0	1
0	0	0	0	0	0	0	0	0
0	0	0	0	0	0	0	0	1
0	0	0	0	0	0	0	0	0
0	0	0	0	0	0	0	0	0
0	0	0	0	0	0	0	0	0
0	0	0	0	0	0	0	0	0
0	0	0	0	0	0	0	0	0
0	0	0	0	0	0	0	0	0
0	0	0	0	0	0	0	0	0
0	0	0	0	0	0	0	0	0
0	0	0	0	0	0	0	0	0
0	0	0	0	0	0	0	0	0
0	0	0	0	0	0	0	0	0
0	0	0	0	0	0	1	0	1
0	0	0	0	0	0	0	0	0
0	0	0	0	0	0	0	0	1
0	0	0	0	0	0	1	0	0
0	0	0	0	0	0	0	1	0
0	0	0	0	0	0	0	0	0
0	0	0	0	0	0	0	0	0
0	0	0	0	0	0	1	0	0
0	0	0	0	0	0	0	0	0
0	0	0	0	0	0	0	0	0
0	0	0	0	0	0	0	0	9
0	0	0	0	0	0	0	0	0
0	0	0	0	0	0	0	0	1
0	0	0	0	0	0	0	0	0
0	0	0	0	0	0	0	0	0
0	0	0	0	0	0	0	0	1
0	0	0	0	0	0	0	0	0
0	0	0	0	0	0	0	0	4
0	0	0	0	0	0	0	0	0
0	0	0	0	0	0	0	0	0
0	0	0	0	0	0	0	0	0
0	0	0	0	0	0	0	0	0
0	0	0	0	0	0	0	0	0
0	0	0	0	0	0	0	0	7
0	0	0	0	0	1	0	0	0
0	0	0	1	0	0	0	0	0
0	0	0	1	0	0	0	0	0
0	0	0	0	0	0	0	0	0
0	0	0	0	0	0	0	0	0
0	0	0	0	0	0	0	0	1
0	0	0	0	0	0	1	0	0
0	0	0	0	0	0	0	1	0
0	0	0	0	0	0	0	0	1
0	0	0	0	0	0	0	0	0
0	0	0	0	0	0	0	0	0
0	0	0	0	0	1	0	0	0
0	0	0	0	0	0	0	0	0

0	0	0	0	0	0	3	0	1
0	0	0	0	0	0	0	0	0
0	0	0	0	0	0	0	0	0
0	0	0	0	0	0	0	0	0
0	0	0	0	0	0	0	0	0
0	0	0	0	0	0	0	0	2
0	0	0	0	0	0	0	0	8
0	0	0	0	0	0	0	0	2
0	0	0	0	0	0	0	0	2
0	0	0	0	0	0	0	0	0
0	0	0	0	0	0	0	0	1
0	0	0	0	0	0	0	0	2
0	0	0	0	0	0	0	0	1
0	0	0	0	0	0	0	0	0

0	0	0	0	0	0	0	1	3
0	0	0	0	1	0	0	0	1
0	0	0	0	0	0	1	0	3
0	0	0	0	0	0	0	0	1
0	0	0	0	0	0	0	0	3
0	0	0	0	0	0	0	0	0
0	0	0	0	0	0	0	0	3
0	0	0	0	0	0	0	0	0
0	0	0	0	0	0	0	0	1

0	0	0	0	0	0	0	0	1
0	0	0	0	0	0	0	1	0
0	0	0	0	0	0	0	0	0
0	0	0	0	0	0	0	0	0
0	0	0	0	0	0	0	0	0
0	0	0	0	0	0	0	0	0
0	0	0	0	0	0	0	0	1
0	0	0	0	0	0	0	0	0
0	0	0	0	0	0	0	0	0
0	0	0	0	0	0	0	0	0
0	0	0	0	0	0	0	0	0
0	0	0	0	0	0	0	1	1
0	0	0	0	0	0	0	0	3
0	0	0	0	0	0	0	0	1
0	0	0	0	0	0	0	0	0
0	0	1000	0	0	0	0	0	0
0	0	1000	0	0	0	0	0	0
0	1	0	0	0	0	0	0	0
0	0	0	0	0	0	0	0	0
0	0	0	0	0	0	0	0	1
0	0	0	0	0	0	35	0	0
0	0	0	0	0	0	0	0	0
0	0	0	0	0	0	0	0	0
0	0	0	0	0	0	0	0	1
0	0	0	0	0	0	1	0	0
0	0	0	0	0	0	0	1	0
0	0	0	0	0	0	0	0	0
0	0	0	0	0	0	0	0	0
0	0	0	0	0	0	1	0	0

0	0	0	0	0	0	0	0	0
0	0	0	0	0	0	1	0	0
0	0	0	0	0	0	0	0	0
0	0	1000	0	0	0	0	0	0
0	0	0	0	0	1	0	0	0
0	0	0	0	0	0	0	0	0
0	0	0	0	0	0	0	0	0
0	0	0	0	0	0	0	0	0
0	0	0	0	0	0	0	0	0
0	0	0	0	0	0	0	0	0
0	0	0	0	0	0	0	0	0
0	0	0	0	0	0	0	0	1
0	0	0	0	0	0	0	0	1
0	0	0	0	0	0	0	0	0
0	0	0	0	0	0	0	0	0
0	0	0	0	0	0	0	0	0
0	0	0	0	0	0	1	0	0
0	0	0	0	0	1	0	0	0
0	0	0	0	0	0	0	0	0
0	0	0	0	0	0	3	0	1
0	0	0	0	0	0	0	0	0
0	0	0	0	0	0	0	0	0
0	0	0	0	0	0	0	0	0
0	0	0	0	0	0	0	0	0
0	0	0	0	0	0	0	0	0

0	0	1000	0	0	0	0	0	0
0	0	0	0	0	0	0	0	0
0	0	0	0	0	0	0	0	0
0	0	0	0	0	0	0	0	1
0	0	0	0	0	0	0	0	0
0	0	0	0	0	0	0	0	1
0	0	0	0	0	0	0	1	0
0	0	0	0	0	0	0	0	0
0	0	0	0	0	0	0	0	0
0	0	0	0	0	0	0	0	0
0	0	0	0	0	0	0	0	0
0	0	0	0	0	0	0	0	0
0	0	0	0	0	0	0	0	0
0	0	1000	0	0	0	0	0	0
0	0	0	0	0	0	0	0	0
0	0	0	0	0	0	0	0	0
0	0	0	0	0	0	0	0	1
0	0	0	0	0	0	0	0	0
0	0	0	0	0	0	0	0	1
0	0	0	0	0	0	0	1	0
0	0	0	0	0	0	0	0	1
0	0	0	0	0	0	3	0	1
0	0	0	0	0	0	0	0	0
0	0	0	0	0	0	0	0	0

0	0	0	0	0	0	0	0	4
0	0	0	0	0	0	0	1	0
0	0	0	0	0	0	0	0	0
0	0	0	0	0	0	0	0	0
0	0	0	0	0	0	0	0	2

0	0	0	0	0	0	0	0	0
0	0	0	0	0	0	0	0	0
0	0	0	0	0	0	0	0	0
0	0	0	0	0	2	0	0	0
0	0	0	0	0	0	0	0	0
0	0	0	0	0	0	0	0	0
0	0	0	0	0	0	0	0	1
0	0	0	0	0	0	0	0	0
0	0	0	0	0	0	0	0	0
0	0	0	0	0	0	0	0	1
0	0	0	0	0	0	0	0	0
0	0	0	0	0	0	0	0	4
0	0	0	0	0	0	0	0	0
0	0	0	0	0	1	0	0	0
0	0	0	0	0	0	1	0	3
0	0	0	0	0	0	0	0	1
0	0	0	0	0	0	0	0	3
0	0	0	0	0	0	0	0	0
0	0	0	0	0	0	0	0	3
0	0	0	0	0	0	0	0	0
0	0	0	0	0	0	0	1	0
0	0	0	0	0	0	0	0	0
0	0	0	0	0	0	0	0	0
0	0	0	0	0	0	0	0	0
0	0	0	0	0	0	0	0	6
0	0	0	0	0	0	0	0	4
0	0	0	0	0	0	0	0	0
0	0	0	0	0	0	0	0	0
0	0	0	0	0	0	0	0	0
0	0	0	0	0	0	0	0	0
0	0	0	0	0	0	0	0	0
0	0	0	0	0	0	0	0	0

0	0	0	0	0	0	0	0	0
0	0	0	0	0	0	0	0	0
0	0	0	0	0	0	0	0	0
0	0	0	0	0	0	0	0	0
0	0	0	0	0	0	0	0	0
0	0	0	0	0	0	0	0	0
0	0	0	0	0	1	0	0	0
0	0	0	0	0	0	0	0	0
0	0	0	0	0	0	0	0	0
0	0	0	0	0	0	0	0	0
0	0	0	0	0	0	0	0	0
0	0	0	0	0	0	1	0	0
0	0	0	0	0	0	0	0	2
0	0	0	0	0	0	0	0	1
0	0	0	0	0	0	0	0	20
0	0	0	0	0	0	0	0	4
0	0	0	0	0	0	0	0	0
0	0	0	0	0	0	0	0	0
0	0	0	0	0	0	0	0	0
0	0	0	0	0	0	0	0	1

0	0	0	0	0	0	0	0	6
0	0	0	0	0	0	0	0	2
0	0	0	0	0	0	0	0	1
0	0	0	0	0	0	0	0	1
0	0	0	0	0	0	0	0	0
0	0	1000	0	0	0	0	0	0
0	0	0	0	0	0	0	0	0
0	0	1000	0	0	0	0	0	3
0	0	1000	0	0	0	0	0	0
0	0	0	0	0	1	0	0	0
0	0	0	0	0	0	0	0	0
0	0	0	0	0	0	0	0	0
0	0	1000	0	0	0	0	1	0
0	0	0	0	0	0	0	0	0
0	0	0	0	0	0	1	0	1
0	0	0	0	0	0	0	0	0
0	0	0	0	0	0	0	0	0
0	0	0	0	0	0	0	0	0
0	0	0	0	0	0	0	1	0
0	0	0	0	0	0	0	0	0
0	0	0	0	0	0	0	0	0
0	0	0	0	0	0	0	0	0
0	0	0	0	0	0	0	0	6
0	0	0	0	0	0	0	0	4
0	0	0	0	0	0	0	0	0
0	0	0	0	0	0	0	0	1
0	0	0	0	0	0	0	0	0
0	0	0	0	0	0	0	0	4

0	0	0	0	0	0	0	0	0
0	0	0	0	0	0	0	0	0
0	0	0	0	0	0	0	0	0
0	0	0	0	0	0	0	0	6
0	0	0	0	0	0	0	0	4
0	0	0	0	0	0	0	0	0
0	0	0	0	0	0	0	0	1
0	0	0	0	0	0	0	0	3
0	0	0	0	0	0	0	0	0
0	0	0	0	0	0	0	0	0
0	0	0	0	0	1	0	0	0
0	0	0	0	0	0	0	0	3
0	0	0	0	0	0	0	0	1
0	0	0	0	0	0	0	0	3
0	0	0	0	0	0	0	0	0
0	0	0	0	0	0	0	0	3
0	0	0	0	0	0	0	0	1
0	0	0	0	0	0	0	0	0
0	0	0	0	0	0	0	0	1
0	0	0	0	0	0	0	0	0
0	0	0	0	0	0	0	0	0
0	0	0	0	0	0	0	0	0
0	0	1000	0	0	0	0	0	0
0	0	0	0	0	0	0	0	0
0	0	0	0	0	0	0	0	0
0	0	0	0	0	0	0	0	0
0	0	0	0	0	0	0	0	0

0	0	0	0	0	0	0	1	1
0	0	0	0	0	0	0	0	3
0	1	0	0	0	0	0	0	0
0	0	0	0	0	0	0	0	0
0	0	0	0	0	0	0	0	0
0	0	0	0	0	1	0	0	0
0	0	0	0	0	0	0	0	0
0	0	0	0	0	0	0	0	0
0	0	0	0	0	0	0	0	1
0	0	0	0	0	0	0	0	0
0	0	0	0	0	0	0	0	1
0	0	0	0	0	0	0	1	0
0	0	0	0	0	0	0	0	0
0	0	0	0	0	0	0	0	0
0	0	0	0	0	0	0	0	0
0	0	0	0	0	0	0	0	0
0	0	0	0	0	0	0	0	0
0	0	0	0	0	0	0	0	0
0	0	0	0	0	0	0	0	4
0	0	0	0	0	0	0	0	2
0	0	0	0	0	0	0	0	1
0	0	0	0	0	0	0	0	20
0	0	0	0	0	0	0	0	4
0	0	0	0	0	0	0	0	0
0	0	0	0	0	0	0	0	0
0	0	0	0	0	0	0	0	0
0	0	0	0	0	0	0	0	0
0	0	0	0	0	1	0	0	0
0	0	0	0	0	0	0	0	0
0	0	0	0	0	0	0	0	0
0	0	0	0	0	0	0	0	0
0	0	0	0	0	0	0	0	0
0	0	0	0	0	0	0	0	1
0	0	0	0	0	0	1	0	1
0	0	0	0	0	0	0	0	0
0	0	0	0	0	0	0	0	1
0	0	0	0	0	0	1	0	0
0	0	0	0	0	0	0	1	0
0	0	0	0	0	0	0	0	0
0	0	0	0	0	0	0	0	0
0	0	0	0	0	0	0	0	1
0	0	0	0	0	0	2	0	0
0	0	0	0	0	0	0	0	0
0	0	1	0	0	0	0	0	0
0	0	0	0	0	0	0	0	0
0	0	0	0	0	0	0	0	7
0	0	0	0	0	1	0	0	0
0	0	0	1	0	0	0	0	0
0	0	0	0	0	0	0	0	4
0	0	0	0	0	0	0	0	3
0	0	0	0	0	0	1	0	3
0	0	0	0	0	0	0	0	2

levure_ps	asp_fumi_lit	sct_nid_lit	asp_nid_lit	asp_ustus_lit	asp_niger_lit	asp_sp_lit	asp_terreus_lit
0	0	0	0	0	0	0	0
0	0	0	0	0	0	0	0
0	0	0	0	0	0	0	0
0	0	0	0	0	0	0	0
0	0	0	0	0	0	2	0
0	0	0	0	0	0	0	0
0	0	0	0	0	0	0	0
1	0	0	0	0	0	0	0
0	0	0	0	0	0	0	0
0	0	0	0	0	0	0	0
1	0	0	0	0	0	0	0
0	0	0	0	0	0	0	0
0	0	0	0	0	0	0	0
1	0	0	0	0	0	0	0
3	0	0	0	0	0	0	0
1	0	0	0	0	0	0	0
0	0	0	0	0	0	0	0
3	0	0	0	0	0	0	0
0	0	1	0	0	0	0	0
0	0	0	0	0	0	0	0
0	0	0	0	0	0	0	0
0	0	0	0	0	0	0	0
3	0	0	0	0	0	0	0
0	0	0	0	0	0	0	0
12	0	0	0	0	0	0	0
0	0	1	0	0	0	0	0
1	0	0	0	0	0	0	0
1	0	0	0	0	0	0	0
2	0	0	0	0	0	0	0
0	0	0	0	0	0	0	0
0	0	0	0	0	0	0	0
0	0	0	0	0	0	0	0
0	0	0	0	0	0	0	0
0	0	0	0	0	0	0	0
0	0	0	0	0	0	0	0
0	0	0	0	0	0	0	0
0	0	0	0	0	0	3	0
0	0	0	0	0	0	0	0
0	0	0	0	0	0	0	0
0	0	1	0	0	0	0	0
0	0	0	0	0	0	0	0
0	0	0	0	0	0	0	0
2	0	0	0	0	0	0	0
0	0	0	0	0	0	0	0
0	0	0	0	0	0	0	0
1	0	0	0	0	0	0	0
3	0	1	0	0	0	0	0
1	0	0	0	0	0	0	0
0	0	0	0	0	0	0	0
3	0	0	0	0	0	0	0
0	0	0	0	0	0	0	0
1	0	0	0	0	0	0	0
0	0	0	0	0	0	0	0
0	0	0	0	0	0	0	0
2	0	0	0	0	0	0	0

0	0	0	0	0	0	0	0
0	0	0	0	0	0	0	0
2	0	0	0	0	0	0	0
0	0	0	0	0	0	0	0
0	0	0	0	0	0	0	0
3	0	0	0	0	0	0	0
1	0	0	0	0	0	0	0
0	0	0	0	0	0	1	0
0	0	0	0	0	0	0	0
0	0	0	0	0	0	0	0
0	0	0	0	0	0	0	0
0	1	1	0	0	2	2	0
0	0	0	0	0	0	0	0
0	0	0	0	0	0	0	0
2	0	0	0	0	0	0	0
0	0	0	0	0	0	0	0
0	0	0	0	0	0	0	0
0	0	0	0	0	0	0	0
0	0	0	0	0	0	0	0
1	0	0	0	0	0	0	0
1	0	0	0	0	0	0	0
0	0	0	0	0	0	0	0
0	0	0	0	0	0	0	0
0	0	0	0	0	0	0	0
2	0	0	0	0	0	0	0
0	0	0	0	0	0	0	0
0	0	0	0	0	0	0	0
0	0	1	0	0	0	0	0
0	0	0	0	0	0	0	0
0	0	0	0	0	0	0	0
1	0	0	0	0	0	0	0
0	0	0	0	0	0	1	0
0	0	0	0	0	0	0	0
0	0	0	0	0	0	0	0
0	0	0	0	0	0	0	0
0	0	0	0	0	0	0	0

2	0	0	0	0	0	0	0
1	0	0	0	0	0	0	0
0	0	0	0	0	0	0	0
0	0	0	0	0	0	0	0
2	0	0	0	0	0	0	0
0	0	0	0	0	0	0	0
0	0	0	0	0	0	0	0
1	0	0	0	0	0	1	0
3	0	0	0	0	0	0	0
1	0	0	0	0	0	0	0
0	0	0	0	0	0	0	0
3	0	0	0	0	0	0	0
0	0	0	0	0	0	0	0
0	0	0	0	0	0	0	0
0	0	0	0	0	0	0	0
0	0	0	0	0	0	0	0
3	0	0	0	0	0	0	0
1	1	0	0	0	0	0	0
0	0	0	0	0	0	0	0

0	0	0	0	0	0	0	0
4	0	0	0	0	0	0	0
1	0	0	0	0	0	0	0
1	0	0	0	0	0	0	0
0	0	0	0	0	0	0	0
0	0	0	0	0	0	0	0
0	0	0	0	0	0	0	0
1	0	0	0	0	0	0	0
0	1	0	0	0	0	0	0
0	0	0	0	0	0	0	0
0	0	0	0	0	0	0	0
0	0	0	0	0	0	0	0
0	0	0	0	0	0	0	0
0	0	0	0	0	0	0	0
0	0	0	0	0	0	0	0
0	0	0	0	0	0	0	0
0	0	0	0	0	0	0	0
1	0	0	0	0	0	0	0
1	0	0	0	0	0	0	0
3	0	0	0	0	0	0	0
1	0	0	0	0	0	0	0
1	0	0	0	0	0	0	0
0	0	0	0	0	0	0	0
0	0	0	0	0	0	0	0
0	0	0	0	0	0	0	0
0	0	0	0	0	0	0	0
0	0	0	0	0	0	0	0
5	0	0	0	0	0	0	0
0	0	0	0	0	0	0	0
0	0	0	0	0	0	0	0
0	0	0	0	0	0	0	0
9	0	0	0	0	0	0	0
0	0	0	0	0	0	0	0
0	0	0	0	0	0	0	0
1	0	1	0	0	0	0	0
0	0	0	0	0	0	0	0
1	0	0	0	0	0	0	0
0	0	0	0	0	0	0	0
1	0	0	0	0	0	0	0
0	0	0	0	0	0	0	0
0	0	0	0	0	0	0	0
0	0	0	0	0	0	0	0
0	0	0	0	0	0	0	0
0	0	0	0	0	0	0	0
0	0	0	0	0	0	0	0
1	0	0	0	0	0	0	0
3	0	0	0	0	0	0	0
1	0	0	0	0	0	0	0
1	0	0	0	0	0	0	0
0	0	0	0	0	0	0	0
0	0	0	0	0	0	0	0
0	0	0	0	0	0	0	0
2	0	0	0	0	0	0	0
0	0	0	0	0	0	0	0

0	0	0	0	0	0	0	0
0	0	0	0	0	0	0	0
0	0	0	0	0	0	0	0
0	0	0	0	0	0	0	0
1	0	0	0	0	0	0	0
0	0	0	0	0	0	0	0
2	0	0	0	0	0	0	0
1	0	0	0	0	0	0	0
0	0	0	0	0	0	0	0
0	0	0	0	0	0	0	0
0	0	0	0	0	0	0	0
6	0	0	0	0	0	0	0
0	0	0	0	0	0	0	0
0	0	0	0	0	0	0	0
2	0	0	0	0	0	0	0
1	0	0	0	0	0	0	0
0	0	0	0	0	0	0	0
0	0	0	0	0	0	0	0
2	0	0	0	0	0	0	0
0	0	0	0	0	0	0	0
0	0	0	0	0	0	0	0
1	0	0	0	0	0	0	0
3	0	5	0	0	1	0	0
0	0	0	0	0	0	0	0
0	0	0	0	0	0	0	0
0	0	0	0	0	0	0	0
0	0	0	0	0	0	0	0
0	0	0	0	0	0	0	0
1	0	0	0	0	0	0	0
0	0	0	0	0	0	0	0
0	0	0	0	0	0	0	0
0	0	0	0	0	0	0	0
3	0	0	0	0	0	0	0
0	0	0	0	0	0	0	0
12	0	0	0	0	0	0	0
0	0	0	0	0	0	0	0
0	0	0	0	0	0	0	0
0	0	0	0	0	0	0	0
0	0	0	0	0	0	0	0
0	0	0	0	0	0	0	0
0	0	0	0	0	0	0	0
2	0	0	0	0	0	0	0
0	0	0	0	0	0	0	0
0	0	0	0	0	0	0	0
1	0	0	0	0	0	1	0
0	0	0	0	0	0	1	0
3	0	0	0	0	0	0	0
1	0	0	0	0	0	0	0
1	0	0	0	0	0	0	0
0	0	0	0	0	0	0	0
0	0	0	0	0	0	0	0
0	0	0	0	0	0	0	0

0	0	0	0	0	0	0	0
0	0	0	0	0	0	0	0
1	0	0	0	0	0	0	0
0	0	0	0	0	0	0	0
0	0	0	0	0	0	1	0
0	0	0	0	0	1	0	0
0	0	0	0	0	0	0	0
1	0	0	0	0	0	0	0
0	0	0	0	0	0	0	0
0	0	0	0	0	0	0	0
0	0	0	0	0	0	0	0
3	0	0	0	0	0	1	0
0	0	0	0	0	0	0	0
0	0	0	0	0	0	0	0
0	0	0	0	0	0	0	0
0	0	0	0	0	0	0	0
0	0	0	0	0	0	0	0
2	0	0	0	0	0	0	0
0	0	0	0	0	0	0	0
0	0	0	0	0	0	0	0
0	0	0	0	0	0	0	0
0	0	0	0	0	0	0	0
0	0	0	0	0	0	0	0
0	0	0	0	0	0	0	0
0	0	0	0	0	0	0	0
0	0	0	0	0	0	0	0
0	0	0	0	0	0	0	0
0	0	0	0	0	0	0	0
0	0	0	0	0	0	0	0
0	0	0	0	0	0	0	0
0	0	0	0	0	0	0	0
0	0	0	0	0	0	0	0
0	0	0	0	0	0	0	0
0	0	0	0	0	0	0	0
0	0	0	0	0	0	0	0
1	0	0	0	0	0	0	0
0	0	0	0	0	0	0	0
0	0	0	0	0	0	0	0
0	0	0	0	0	0	0	0
0	0	0	0	0	0	0	0
0	0	0	0	0	0	0	0
0	0	0	0	0	0	0	0
0	0	0	0	0	0	0	0
0	0	0	0	0	0	0	0
0	0	0	0	0	0	0	0
0	0	0	0	0	0	0	0
0	0	0	0	0	0	0	0
0	0	1	0	0	0	0	0
0	0	1	0	0	0	0	0
1	0	0	0	0	0	0	0
0	0	0	0	0	0	0	0
0	0	0	0	0	0	0	0
0	0	0	0	0	0	0	0
1	0	0	0	0	0	0	0

0	0	0	0	0	0	0	0
0	0	0	0	0	0	0	0
0	0	0	0	0	0	0	0
1	0	0	0	0	0	0	0
0	0	0	0	0	0	0	0
0	0	0	0	0	0	0	0
0	0	0	0	0	0	0	0
0	0	0	0	0	0	0	0
3	0	0	0	0	0	0	0
0	0	0	0	0	0	0	0
0	0	0	0	0	0	0	0
1	0	0	0	0	0	0	0
0	0	0	0	0	0	0	0
1	0	0	0	0	0	0	0
0	0	0	0	0	0	0	0
0	0	0	0	0	0	0	0
2	0	0	0	0	0	0	0
0	0	0	0	0	0	0	0
0	0	0	0	0	0	0	0
0	0	0	0	0	0	0	0
0	0	0	0	0	0	0	0
0	0	0	0	0	0	0	0
0	0	1	0	0	0	0	0
0	0	0	0	0	0	0	0
0	0	1	0	0	0	0	0
1	0	0	0	0	0	0	0
9	0	0	0	0	0	0	0
0	0	0	0	0	0	0	0
0	0	0	0	0	0	0	0
0	0	0	0	0	0	0	0
0	0	0	0	0	0	0	0
0	0	0	0	0	0	0	0

0	0	0	0	0	0	0	0
2	0	0	0	0	0	0	0
0	0	0	0	0	0	0	0
1	0	0	0	0	0	0	0
0	0	0	0	0	0	0	0
1	0	0	0	0	0	0	0
0	0	0	0	0	0	0	0
0	0	0	0	0	0	0	0
1	0	0	0	0	0	0	0
0	0	0	0	0	0	0	0
0	0	0	0	0	0	0	0
0	0	0	0	0	0	0	0
0	0	0	0	0	0	0	0
0	0	0	0	0	0	0	0
0	0	0	0	0	0	0	0
0	0	0	0	0	0	0	0
0	0	0	0	0	0	0	0
1	0	0	0	0	0	0	0
0	0	0	0	0	0	0	0
0	0	0	0	0	0	0	0

0	0	0	0	0	0	0	0
0	0	0	0	0	0	0	0
0	0	0	0	0	0	0	0
0	0	0	0	0	0	0	0
1	0	0	0	0	0	0	0
0	0	0	0	0	0	0	0
0	0	0	0	0	0	0	0
1	0	0	0	0	0	0	0
0	0	0	0	0	0	0	0
0	0	0	0	0	0	0	0
0	0	0	0	0	0	2	0
0	0	0	0	0	0	0	0
0	0	0	0	0	0	0	0
0	0	0	0	0	0	0	0
1	0	0	0	0	0	0	0
1	0	0	0	0	0	0	0
0	0	0	0	0	0	0	0
1	0	0	0	0	0	0	0
0	0	0	0	0	0	0	0
0	0	0	0	0	0	0	0
0	0	0	0	0	0	0	0
0	0	0	0	0	0	0	0
0	0	0	0	0	0	0	0
1	0	0	0	0	0	1	0
9	0	0	0	0	0	0	0
0	0	0	0	0	0	0	0
0	0	0	0	0	0	0	0
1	0	0	0	0	0	0	0

0	0	0	0	0	0	0	0
0	0	0	0	0	0	0	0
0	0	0	0	0	0	0	0
0	0	0	0	0	0	0	0
0	0	0	0	0	1	0	0
0	0	0	0	0	0	0	0
0	0	0	0	0	0	0	0
0	0	0	0	0	0	0	0
0	0	0	0	0	0	0	0
0	0	0	0	0	0	0	0
7	0	0	0	0	0	0	0
0	2	0	0	0	0	0	0
0	0	0	0	0	0	0	0
2	0	0	0	0	0	0	0
0	0	0	0	0	0	0	0
0	0	0	0	0	0	0	0
0	0	0	0	0	0	0	0
0	0	0	0	0	0	0	0
0	0	0	0	0	0	0	0
0	0	0	0	0	0	0	0
0	0	0	0	0	0	0	0
0	0	0	0	0	0	0	0
0	0	0	0	0	0	0	0
0	0	0	0	0	0	0	0
0	0	0	0	0	0	0	0
3	0	0	0	0	0	0	0
0	0	0	0	0	0	0	0

12	0	0	0	0	0	0	0
0	0	0	0	0	0	0	0
0	0	0	0	0	0	0	0
2	0	0	0	0	0	0	0
0	0	0	0	0	0	0	0
0	0	0	0	0	0	0	0
4	0	0	0	0	0	0	0
1	0	0	0	0	0	0	0
0	0	0	0	0	0	0	0
0	0	0	0	0	0	0	0
0	0	0	0	0	0	0	0
0	0	0	0	0	0	0	0
0	0	0	0	0	0	0	0
0	0	0	0	0	0	0	0
0	0	0	0	0	0	0	0
1	0	0	0	0	0	0	0
0	0	0	0	0	0	0	0
0	0	0	0	0	0	0	0
0	0	0	0	0	0	0	0
0	0	0	0	0	0	0	0
0	0	0	0	0	0	0	0
0	0	0	0	0	0	0	0
0	0	0	0	0	0	0	0
0	0	0	0	0	0	0	0
1	0	0	0	0	0	0	0
0	0	0	0	0	0	0	0
0	0	0	0	0	0	0	0
0	0	0	0	0	0	0	0
0	0	0	0	0	0	0	0
0	0	0	0	0	0	0	0
0	0	0	0	0	0	0	0
1	0	0	0	0	0	0	0
0	0	0	0	0	0	0	0
1	0	0	0	0	0	0	0
1	0	0	0	0	0	0	0
3	0	0	0	0	0	0	0
1	0	0	0	0	0	0	0
1	0	0	0	0	0	0	0
0	0	0	0	0	0	0	0
0	0	0	0	0	0	0	0
3	0	0	0	0	0	0	0
1	0	0	0	0	0	0	0
0	0	0	0	0	0	0	0
3	0	0	0	0	0	0	0
0	0	0	0	0	0	0	0
0	0	0	0	0	0	0	0
0	0	0	0	0	0	0	0
0	0	0	0	0	0	0	0
0	0	0	0	0	0	0	0
1	0	0	0	0	0	0	0
0	0	1	0	0	0	0	0
0	0	0	0	0	0	0	0

asp_flavus_lit	peni_lit	clado_lit	alterna_lit	ulocla_lit	chaeto_lit	scopula_lit	acremo_lit	fusa_lit	rhizo_lit
0	0	0	0	0	0	0	0	0	0
0	0	0	0	0	0	0	0	0	0
0	0	0	0	0	0	0	0	0	0
0	0	0	0	0	0	0	0	0	0
0	0	0	0	0	0	0	0	0	0
0	0	0	0	0	0	0	0	0	0
0	1	0	0	0	0	0	0	0	0
0	0	0	0	0	0	0	0	0	0
0	0	0	0	0	0	0	0	0	0
0	0	0	0	0	0	0	0	0	0
0	0	0	0	0	0	0	0	0	0
0	0	0	0	0	0	0	0	0	0
0	1	0	0	0	0	0	0	0	0
0	0	0	0	0	0	0	0	0	0
0	0	0	0	0	0	0	0	0	0
0	0	0	0	0	0	0	0	0	0
0	1	0	0	0	0	0	0	0	0
0	1	0	0	0	0	0	0	0	0
0	0	0	0	0	0	0	0	0	0
0	0	0	0	0	0	0	0	0	0
0	0	0	0	0	0	0	0	0	0
0	0	0	0	0	0	0	0	0	0
0	0	0	0	0	0	0	0	0	0
0	0	0	0	0	0	0	0	0	0
0	0	0	0	0	0	0	0	0	0
0	3	0	0	0	0	0	0	0	0
0	0	0	0	0	0	0	0	0	0
0	0	0	0	0	0	0	0	0	0
0	0	0	0	0	0	0	0	0	0
0	0	0	0	0	0	0	0	0	0
0	0	0	0	0	0	0	0	0	0
0	0	0	0	0	0	0	0	0	0
0	0	0	0	0	0	0	0	0	0
0	0	0	0	0	0	0	0	0	0
0	0	0	0	0	1	0	0	0	0
0	0	0	0	0	0	0	0	0	0
0	0	0	0	0	0	0	0	0	0
0	0	0	0	0	0	0	0	0	0
0	0	0	0	0	0	0	0	0	0
0	0	0	0	1	0	0	0	0	0
0	0	0	0	0	0	0	0	0	0
0	0	0	0	0	0	0	0	0	0
0	0	0	0	0	0	0	0	0	0
0	0	0	0	0	0	0	0	0	0
0	0	0	1	0	0	0	0	0	0
0	1	1	0	0	0	0	0	0	0
0	3	0	1	0	0	0	0	0	0
0	0	0	0	0	0	0	0	0	0
0	3	0	0	0	0	0	0	0	0
0	0	0	0	0	0	0	0	0	0
0	0	0	0	0	0	0	0	0	0
0	0	0	0	0	0	0	0	0	0
0	0	0	0	0	0	0	0	0	0
0	0	0	0	0	0	0	0	0	0
0	0	0	0	0	0	0	0	0	0

0	0	0	0	0	0	0	0	0	0
0	0	0	0	0	0	0	0	0	0
0	0	0	0	0	0	0	0	0	0
0	0	0	0	0	0	0	0	0	0
0	0	0	0	0	0	0	0	0	0
0	0	0	0	0	0	0	0	0	0
0	0	0	0	0	0	0	0	0	0
0	0	0	1	0	0	0	0	0	0
0	0	0	0	0	0	0	0	0	0
0	0	0	0	0	0	0	0	0	0
0	0	0	0	0	0	0	0	0	0
1	1	0	0	0	0	0	0	0	0
0	0	0	0	0	0	0	0	0	0
0	0	0	0	0	0	0	0	0	0
0	0	0	0	0	0	0	0	0	0
0	0	0	0	0	0	0	0	0	0
0	0	0	0	0	0	0	0	0	0
0	0	0	0	0	0	0	0	0	0
0	0	0	0	0	0	0	0	0	0
0	0	0	0	0	0	0	0	0	0
0	0	0	0	0	0	0	0	0	0
0	0	0	0	0	0	0	0	0	0
0	0	0	0	0	0	0	0	0	0
0	0	0	0	0	0	0	0	0	0
0	0	0	0	0	0	0	0	0	0
0	0	0	0	0	0	0	0	0	0
0	0	0	0	0	0	0	0	0	0
0	0	0	0	0	0	0	0	0	0
0	0	0	0	0	0	0	0	0	0
0	0	0	0	0	0	0	0	0	0
0	0	0	0	0	0	0	0	0	0
0	0	0	0	0	0	0	0	0	0
0	0	0	0	0	0	0	0	0	0
0	0	0	0	0	0	0	0	0	0
0	0	0	0	0	0	0	0	0	0
0	0	0	0	0	0	0	0	0	0

0	0	0	0	0	0	0	0	0	0
0	0	0	0	0	0	0	0	0	0
0	0	0	0	0	0	0	0	0	0
0	0	0	0	0	0	0	0	0	0
0	0	0	0	0	0	0	0	0	0
0	0	0	0	0	0	0	0	0	1
0	0	0	0	0	0	0	0	0	0
0	1	0	0	0	0	0	0	0	0
0	2	3	0	0	0	0	0	0	0
0	0	1	0	0	0	0	0	0	0
0	0	0	0	0	0	0	0	0	0
0	2	0	0	0	1	0	0	0	0
0	0	0	0	0	0	0	0	0	0
0	0	0	0	0	0	0	0	0	0
0	0	0	0	0	0	0	0	0	0
0	0	0	0	0	0	0	0	0	0
0	0	0	0	0	0	0	0	0	0
0	0	0	0	0	0	0	0	0	0
0	0	0	0	0	0	0	0	0	0

0	0	0	0	0	0	0	0	0	0
0	0	0	0	0	0	0	0	0	0
0	0	0	0	0	0	0	0	0	0
0	0	0	0	0	0	0	0	0	0
0	0	0	0	0	0	0	0	0	0
0	0	0	0	0	0	0	0	0	0
0	0	0	0	0	0	0	0	0	0
0	0	0	0	0	0	0	0	0	0
0	0	0	0	0	0	0	0	0	0
0	0	0	0	0	0	0	0	0	0
0	0	0	0	0	0	0	0	0	0
0	0	0	0	0	0	0	0	0	0
0	0	0	0	0	0	0	0	0	0
0	0	0	0	0	0	0	0	0	0
0	0	0	0	0	0	0	0	0	0
0	0	0	0	0	0	0	0	0	0
0	0	0	0	0	0	0	0	0	0
0	0	0	1	0	0	0	0	0	0
0	0	0	0	0	0	0	0	0	0
0	0	0	0	0	0	0	0	0	0
0	0	0	0	0	0	0	0	0	0
0	0	0	0	0	0	0	0	0	0
0	0	0	0	0	0	0	0	0	0
0	0	0	0	0	0	0	0	0	0
0	1	0	0	0	0	0	0	0	0
0	0	0	0	0	0	0	0	0	0
0	0	0	0	0	0	0	0	0	0
0	0	0	0	0	0	0	0	0	0
0	0	0	0	0	0	0	0	0	0
0	0	0	0	0	0	0	0	0	0
0	0	0	0	0	0	0	0	0	0
0	1	0	1	0	0	0	0	0	0
0	0	0	0	0	0	0	0	0	0
0	0	0	0	0	0	0	0	0	0
0	0	1	0	0	0	0	0	0	0

0	0	0	0	0	0	0	0	0	0
0	0	0	0	0	0	0	0	0	0
0	1	0	0	0	0	0	0	0	0
0	0	0	0	0	0	0	0	0	0
0	0	0	0	0	0	0	0	0	0
0	0	0	0	0	0	0	0	0	0
0	0	0	0	0	0	0	0	0	0
0	0	0	0	0	0	0	0	0	0
0	0	0	0	0	0	0	0	0	0
0	0	0	0	0	0	0	0	0	0
0	0	0	0	0	0	0	0	0	0
0	0	0	0	0	0	0	0	0	0
0	0	0	0	0	0	0	0	0	0
0	0	0	1	0	0	0	0	0	0

0	0	0	0	0	0	0	0	0	0
0	0	0	0	0	0	0	0	0	0
0	0	0	0	0	1	0	0	0	0
0	0	0	0	0	0	0	0	0	0
0	0	0	0	0	0	0	0	0	0

0	0	0	0	0	0	0	0	0	0
0	0	0	0	0	0	0	0	0	0
0	0	0	0	0	0	0	1	0	0
0	0	0	0	0	0	0	0	0	0
0	1	0	0	0	0	0	0	0	0
0	0	0	0	0	0	0	0	0	0
0	0	0	0	0	0	0	0	0	0
0	0	0	0	0	0	0	0	0	0
0	0	0	0	0	0	0	0	0	0
0	0	0	0	0	0	0	0	0	0
0	0	0	0	0	0	0	0	0	0
0	1	0	0	0	0	0	0	0	0
0	0	0	0	0	0	0	0	0	0
0	0	0	0	0	0	0	0	0	0

0	0	0	0	0	0	0	0	0	0
0	0	0	0	0	0	0	0	0	0
0	0	0	0	0	0	0	0	0	0
0	0	0	1	0	0	0	0	0	0
0	0	0	0	0	0	0	0	0	0
0	0	0	0	0	0	0	0	0	0
0	0	0	0	0	0	0	0	0	0
0	0	0	0	0	0	0	0	0	0
0	0	0	2	0	0	0	0	0	0

0	0	0	0	0	0	0	0	0	0
0	0	0	0	0	1	0	0	0	0
0	0	0	0	0	0	0	0	0	0
0	0	0	0	0	0	0	0	0	0
0	0	0	0	0	0	0	0	0	0
0	0	0	0	0	0	0	0	0	0
0	0	0	0	0	0	0	0	0	0
0	0	0	0	0	0	0	0	0	0
0	1	0	0	0	0	0	0	0	0
0	0	0	0	0	0	0	0	0	0
0	0	1	0	0	0	0	0	0	0
0	0	0	0	0	0	0	0	0	0
0	0	0	0	0	0	0	0	0	0
0	0	0	0	0	0	0	0	0	0
0	0	0	0	0	0	0	0	0	0
0	0	0	0	0	0	0	0	0	0
0	0	0	0	0	0	0	0	0	0
0	0	0	0	0	0	0	0	0	0
0	0	0	0	0	0	0	0	0	0
0	0	0	0	0	0	0	0	0	0
0	1	0	0	0	0	0	0	0	0
0	0	0	0	0	0	0	0	0	0
0	0	0	0	0	0	0	0	0	0
0	0	0	0	0	0	0	0	0	0
0	0	0	0	0	0	0	0	0	0
0	0	0	0	0	0	0	0	0	0
0	0	0	0	0	0	0	0	0	0
0	0	0	0	0	0	0	0	0	0
0	1	0	0	1	0	0	0	0	0

0	0	0	0	0	0	0	0	0	0
0	1	0	0	1	0	0	0	0	0
0	0	0	0	0	0	0	0	0	0
0	1	0	0	0	0	0	0	0	0
0	0	0	0	0	0	0	0	0	0
0	1	0	0	0	0	0	0	0	0
0	0	0	0	0	0	0	0	0	0
0	0	0	0	0	0	0	0	0	0
0	0	0	0	0	0	0	0	0	0
0	0	0	0	0	0	0	0	0	0
0	0	0	0	0	0	0	0	0	0
0	0	0	0	0	0	0	0	0	0
0	0	0	0	0	0	0	0	0	0
0	0	0	0	0	0	0	0	0	0
0	0	0	0	0	0	0	0	0	0
0	1	0	0	0	0	0	0	1	0
0	0	0	0	0	0	0	0	0	0
0	0	0	0	0	0	0	0	0	0
0	0	0	0	0	0	0	0	0	0
0	0	0	0	0	0	0	0	0	0
0	0	0	0	0	0	0	0	0	0
0	0	0	0	0	0	0	0	0	0
0	0	0	0	0	0	0	0	0	0
0	0	0	0	0	0	0	0	0	0
0	0	0	0	0	0	0	0	0	0

0	0	0	0	0	0	0	0	0	0
0	0	0	0	0	0	0	0	0	0
0	0	0	0	0	0	0	0	0	0
0	0	0	0	0	0	0	0	0	0
0	0	0	0	0	0	0	0	0	0
0	0	0	0	0	0	0	0	0	0
0	0	0	0	0	0	0	0	0	0
0	0	0	0	0	0	0	0	0	0
0	0	0	0	0	0	0	0	0	0
0	0	0	0	0	0	0	0	0	0
0	0	0	0	0	0	0	0	0	0
0	0	0	0	0	0	0	0	0	0
0	0	1	0	0	0	0	0	0	0
0	0	0	0	0	0	0	0	0	0
0	0	0	0	0	0	0	0	0	0
0	0	0	0	0	0	0	0	0	0
0	0	0	0	0	0	0	0	0	0
0	0	0	0	0	0	0	0	0	0
0	0	1	0	0	0	0	0	0	0
0	0	0	0	0	0	0	0	0	0
0	0	0	0	0	0	0	0	0	0
0	0	0	0	0	0	0	0	0	0
0	0	0	0	0	0	0	0	0	0
0	0	0	0	0	0	0	0	0	0

0	0	0	0	0	0	0	0	0	0
0	0	0	0	0	0	0	0	0	0
0	0	0	0	0	0	0	0	0	0
0	0	0	0	0	0	0	0	0	0
0	0	0	0	0	0	0	0	0	0

1	0	0	0	0	0	0	0	0	0
0	0	0	0	0	0	0	0	0	0
0	0	0	0	0	0	0	0	0	0
0	0	0	0	0	0	0	0	0	0
0	0	0	0	0	0	0	0	0	0
0	0	0	0	0	0	0	0	0	0
0	0	0	0	0	0	0	0	0	0
0	0	0	0	0	0	0	0	0	0
0	0	0	0	0	0	0	0	0	0
0	0	0	0	0	0	0	0	0	0
0	0	0	0	0	0	0	0	0	0
0	0	0	0	0	0	0	0	0	0
0	0	0	0	0	0	0	0	0	0
0	0	0	0	0	0	0	0	0	0
0	0	0	0	0	0	0	0	0	0
0	0	0	0	0	0	0	0	0	0
0	0	0	0	0	0	0	0	0	0
0	0	0	1	0	0	0	0	0	0
0	0	0	0	0	0	0	0	0	0
0	0	1	0	0	0	0	0	0	0
0	0	0	0	0	0	0	0	0	0
0	0	0	0	0	0	0	0	0	0
0	0	0	0	0	0	0	0	0	0
0	0	0	0	0	0	0	0	0	0
0	0	0	0	0	0	0	0	0	0
0	0	1	0	0	0	0	0	0	0
0	0	0	0	0	0	0	0	0	0
0	0	0	0	0	0	0	0	0	0
0	0	0	0	0	0	0	0	0	0
0	0	0	0	0	0	0	0	0	0
0	0	0	0	0	0	0	0	0	0
0	0	0	0	0	0	0	0	0	0

0	0	0	0	0	0	0	0	0	0
0	0	0	0	0	0	0	0	0	0
0	0	0	0	0	0	0	0	0	0
0	0	0	0	0	0	0	0	0	0
0	0	0	0	0	0	0	0	0	0
0	0	0	0	0	0	0	0	0	0
0	2	0	0	0	0	0	0	0	0
0	0	0	0	0	0	0	0	0	0
0	0	0	0	0	0	0	0	0	0
0	0	0	0	0	0	0	0	0	0
0	0	0	0	0	0	0	0	0	0
0	0	0	0	0	0	0	0	0	0
0	0	0	0	0	0	0	0	0	0
0	0	0	0	0	0	0	0	0	0
0	0	0	0	0	0	0	0	0	0
0	0	0	0	0	0	0	0	0	0
0	0	0	0	0	0	0	0	0	0
0	0	0	0	0	0	0	0	0	0
0	0	9	1	2	0	0	0	0	0
0	1	0	0	0	0	0	0	0	0

0	1	0	0	0	0	0	0	0	0
0	0	0	0	0	0	0	0	0	0
0	0	0	0	0	0	0	0	0	0
0	0	0	0	0	0	0	0	0	0
0	0	0	0	0	0	0	0	0	0
0	0	0	0	0	0	0	0	0	0
0	0	0	0	0	0	0	0	0	0
0	0	0	0	0	0	0	0	0	0
0	0	0	0	0	1	0	0	0	0
0	0	0	0	0	0	0	0	0	0
0	1	0	0	0	0	0	0	0	0
0	0	0	0	0	0	0	0	0	0
0	4	0	0	0	0	0	0	0	0
0	0	0	0	0	0	0	0	0	0
0	1	0	0	0	0	0	0	0	0
0	0	0	0	0	0	0	0	0	0
0	0	0	0	0	0	0	0	0	0
0	0	0	0	0	0	0	0	0	0
0	0	0	0	0	0	0	0	0	0
0	0	0	0	0	0	0	0	0	0
0	6	0	0	0	0	0	0	0	0
0	0	0	0	0	0	0	0	0	0
0	0	0	0	0	0	0	0	0	0
0	0	0	0	0	0	0	0	0	0
0	1	0	0	0	0	0	0	0	0
0	0	0	0	0	0	0	0	0	0
0	0	0	0	0	0	0	0	0	0
0	0	0	0	0	0	0	0	0	0

0	0	0	0	0	0	0	0	0	0
0	0	0	0	0	0	0	0	0	0
0	0	0	0	0	0	0	0	0	0
0	0	0	0	0	0	1	0	0	0
0	0	0	0	0	0	0	0	0	0
0	0	0	1	0	0	0	0	0	0
0	1	0	0	0	0	1	0	0	0
0	0	0	0	0	0	0	0	0	0
0	0	0	0	0	0	0	0	0	0
0	0	0	0	0	0	0	0	0	0
0	0	0	0	0	0	0	0	0	0
0	0	0	0	0	0	0	0	0	0
0	0	0	0	0	0	0	0	0	0
0	0	1	0	0	0	0	0	0	0
0	0	0	0	0	0	0	0	0	0
0	0	2	0	0	0	0	0	0	0
0	0	0	0	0	0	0	0	0	0
0	0	0	0	0	0	0	0	0	0
0	0	0	0	0	0	0	0	0	0
0	0	0	0	0	0	0	0	0	0
0	0	0	0	0	0	0	0	0	0
0	0	0	0	0	0	0	1	0	0
0	0	4	0	0	0	0	0	0	0
0	0	0	0	0	0	0	0	0	0
0	0	0	0	0	0	0	0	0	0
0	4	0	0	0	0	0	0	0	0
0	7	0	0	0	0	0	0	0	0

0	0	3	0	0	0	0	0	0	0
0	4	0	0	0	0	0	0	0	0
0	0	0	0	0	0	0	0	0	0
0	0	0	0	0	0	0	0	0	0
0	0	0	0	0	0	0	0	0	0
0	0	0	0	0	0	0	0	0	0
0	0	0	0	0	0	0	0	0	0
0	0	0	0	0	0	2	0	0	0
0	0	0	0	0	0	0	0	0	0
0	0	0	0	0	0	0	0	0	0
0	0	0	0	0	0	0	0	0	0
0	0	0	0	0	0	0	0	0	0
0	0	0	1	0	0	0	0	0	0
0	0	0	0	0	0	0	0	0	0
0	0	0	0	0	0	0	0	0	0
0	0	0	0	0	0	0	0	0	0
0	0	0	0	0	0	0	0	0	0
0	0	0	0	0	0	0	0	0	0
0	0	0	0	0	0	0	0	0	0
0	0	0	0	0	0	0	0	0	0
0	0	0	0	0	0	0	0	0	0
0	0	0	0	0	0	0	0	0	0
0	0	0	0	0	0	0	0	0	0
0	0	0	0	0	0	0	0	0	0
0	0	0	0	0	0	0	0	0	0
0	7	0	0	0	0	0	0	0	0
0	9	0	0	0	0	0	0	0	0
0	0	0	0	0	0	0	0	0	0
0	0	0	0	0	0	0	0	0	0
0	0	0	0	0	0	0	0	0	0
0	0	0	0	0	0	0	0	0	0
0	0	0	0	0	0	0	0	0	0
0	0	0	0	0	0	0	0	0	0
0	0	0	0	0	0	0	0	0	0
0	1	0	0	0	0	0	0	0	0
0	0	0	0	0	0	0	0	0	0
0	0	0	0	0	0	0	0	0	0
0	0	0	0	0	0	0	0	0	0
0	0	0	0	0	0	0	1	0	0
0	0	0	0	0	0	0	0	0	0
0	2	0	0	0	0	0	0	0	0
0	3	0	0	0	0	0	0	0	0
0	1	0	0	0	0	0	0	0	0
0	0	0	0	0	0	0	0	0	0
0	4	0	0	0	0	0	0	0	1
0	0	0	0	0	0	0	0	0	0
0	0	0	0	0	0	0	0	0	0
0	0	0	0	0	0	0	0	0	0
0	0	0	0	0	0	0	0	0	0
0	0	0	0	0	0	0	0	0	0
0	0	0	0	0	0	0	0	0	0
0	0	0	0	0	0	0	0	0	0

mucor_lit	geotri_lit	trichoderm_lit	paecilo_lit	autres_lit	csd_lit	levure_lit	asp_fumi_ta	sct_nid_ta
0	0	0	0	0	0	0	0	0
0	0	0	0	0	0	0	0	0
0	0	0	0	0	0	0	0	0
0	0	0	0	0	0	0	0	0
0	0	0	0	0	0	0	0	0
0	0	0	0	0	0	0	0	0
0	0	0	0	0	1	0	0	0
0	0	0	0	0	0	0	0	0
0	0	0	0	0	0	25	0	1
0	0	0	0	0	0	0	0	0
0	0	0	0	0	0	0	0	0
0	0	0	0	0	0	0	0	0
0	0	0	0	0	0	0	0	0
0	0	0	0	0	0	0	0	0
0	0	0	0	0	0	2	0	0
0	0	0	0	0	0	0	0	0
0	0	0	0	0	1	2	0	0
0	0	0	0	0	0	0	0	0
2	0	0	0	0	0	2	0	0
0	0	0	0	0	0	0	0	0
0	0	0	0	0	0	0	0	0
0	0	0	0	0	0	0	0	0
0	0	0	0	0	0	0	0	0
0	0	0	0	0	0	0	0	0
0	0	0	0	0	0	0	0	0
0	0	0	0	0	2	0	0	0
0	0	0	0	0	0	0	0	0
0	0	0	0	0	0	0	0	0
0	0	0	0	0	0	0	0	0
0	0	0	0	0	0	0	0	0
0	0	0	0	0	0	0	0	0
0	0	0	0	0	0	0	0	0
0	0	0	0	0	0	0	0	0
0	0	0	0	0	0	0	0	0
0	0	0	0	0	0	0	0	0
0	0	0	0	0	0	0	0	0
0	0	0	0	0	0	0	0	0
0	0	0	0	0	0	0	0	0
0	0	0	0	0	0	0	0	0
0	0	0	0	0	0	0	0	0
0	0	0	0	0	0	0	0	0
0	0	0	0	0	0	0	0	0
0	0	0	0	0	0	0	0	0
0	0	0	0	0	0	0	0	0
0	0	0	0	0	0	0	0	0
0	0	0	0	0	2	2	0	0
0	0	0	0	0	0	0	0	0
0	0	0	0	0	0	0	0	0
0	0	0	0	0	1	1	0	0
0	0	0	0	0	1	0	0	0
0	0	0	0	0	0	0	0	0
0	0	0	0	0	0	0	0	0
0	0	0	0	0	1	0	0	0
0	0	0	0	0	0	0	0	0
0	0	0	0	0	0	0	0	0

0	0	0	0	0	0	1	0	0
0	0	0	0	0	0	0	1	0
0	0	0	0	0	0	0	0	0
0	0	0	0	0	0	0	0	0
0	0	0	0	0	0	0	0	0
0	0	0	0	0	0	0	0	0
0	0	0	0	0	0	0	0	0
0	0	0	0	0	1	0	0	0
0	0	0	0	0	0	0	0	0
0	0	0	0	0	0	0	0	0
0	0	0	0	0	1	0	0	0
0	0	0	0	0	1	0	0	0
0	0	0	0	0	0	0	0	0
0	0	0	0	0	0	0	0	0
0	0	0	0	0	0	0	0	0
0	0	0	0	0	0	0	0	0
0	0	0	0	0	0	0	0	0
0	0	0	0	0	0	0	0	0
0	0	0	0	0	0	0	0	0
0	0	0	0	0	0	0	0	0
0	0	0	0	0	0	0	0	0
0	0	0	0	0	0	0	0	0
0	0	0	0	0	0	0	0	0
0	0	0	0	1	0	0	0	0
0	0	0	0	0	0	0	0	0
0	0	0	0	0	0	1	0	0
0	0	0	0	0	0	0	0	0
0	0	0	0	0	1	0	0	0
0	0	0	0	0	0	0	0	0
0	0	0	0	0	0	0	0	0
0	0	0	0	0	0	0	0	0
0	0	0	0	0	0	1	0	0
0	0	0	0	0	0	0	0	0
0	0	0	0	0	0	0	0	0
0	0	0	0	0	0	0	0	0
0	0	0	0	0	0	0	0	0

0	0	0	0	0	0	0	0	0
0	0	0	0	0	0	0	0	0
0	0	0	0	0	0	0	0	0
0	0	0	0	0	0	0	0	0
0	0	0	0	0	0	0	0	0
0	0	0	0	0	0	0	0	0
0	0	0	0	0	0	0	0	0
0	0	0	0	0	0	0	0	0
0	0	0	0	0	2	99	0	0
0	0	0	0	0	0	0	0	0
0	0	0	0	0	0	0	0	0
0	0	0	0	0	0	0	0	0
0	0	0	0	0	2	0	0	0
0	0	0	0	0	0	0	0	0
0	0	0	0	0	0	0	0	0
0	0	0	0	0	2	0	0	0
0	0	0	3	0	0	0	0	0
0	0	0	0	0	0	0	1	0
0	0	0	0	0	0	0	0	0

0	0	0	0	0	0	0	0	0
0	0	0	0	0	0	0	0	0
0	0	0	0	0	0	0	0	0
0	0	0	0	0	0	0	0	0
0	0	0	0	0	0	0	0	0
0	0	0	0	0	0	0	0	0
0	0	0	0	0	0	0	0	0
0	0	0	0	0	0	0	0	0
0	0	0	0	0	0	0	0	0
0	0	0	0	0	0	1	0	0
0	0	0	0	0	0	0	0	0
0	0	0	0	0	0	0	0	0
0	0	0	0	0	0	0	0	0
0	0	0	0	0	0	0	0	0
0	0	0	0	0	37	1	0	0
0	0	0	0	0	0	0	0	0
0	0	0	0	0	0	0	0	0
0	0	0	0	0	0	2	0	0
0	0	0	0	0	0	0	0	0
0	0	0	0	0	0	0	0	0
0	0	0	0	0	0	0	0	0
0	0	0	0	0	0	1	0	0
0	0	0	0	0	0	0	0	0
0	0	0	0	0	0	0	0	0
0	0	0	0	0	0	0	0	0
0	0	0	0	0	0	0	0	0
0	0	0	0	0	0	0	0	0
0	0	0	0	0	0	0	0	0
0	0	0	0	0	0	1	0	0
0	0	0	0	0	0	0	0	0
0	0	0	0	0	0	0	0	0
0	0	0	0	0	0	0	0	0
0	0	0	0	0	0	0	0	0
0	0	0	0	0	0	0	0	0
0	0	0	0	0	0	0	0	0

0	0	0	0	0	0	0	0	0
0	0	0	0	0	0	0	0	0
0	0	0	0	0	0	0	0	0
0	0	0	0	0	0	0	0	0
0	0	0	0	0	0	0	0	0
0	0	0	0	0	0	0	0	0
0	0	0	0	0	0	0	0	0
0	0	0	0	0	0	0	0	0
0	0	0	0	0	0	0	0	0
0	0	0	0	0	1	0	0	0
0	0	0	0	0	0	3	0	0
0	0	0	0	0	2	0	0	0
0	0	0	0	0	0	0	0	0
0	0	0	0	0	0	0	0	0

0	0	0	0	0	0	0	0	0
0	0	0	0	0	0	0	0	0
0	0	0	0	0	0	2	0	0
0	0	0	0	0	0	0	0	0
0	0	0	0	0	1	0	0	0

0	0	0	0	0	2	0	0	0
0	0	0	0	0	0	0	0	0
0	0	0	1	0	0	0	0	0
0	0	0	0	0	0	0	0	0
0	0	0	0	0	0	1	0	0
0	0	0	0	0	0	0	0	0
0	0	0	0	0	1	0	0	0
0	0	0	0	0	0	0	0	0
0	0	0	0	0	1	1	0	0
0	0	0	0	0	0	0	0	0
0	0	0	0	0	1	0	0	0
0	0	0	0	0	0	0	0	0
0	0	0	0	0	1	0	0	0
0	0	0	0	0	0	0	0	0

0	0	0	0	0	0	0	0	0
0	0	0	0	0	0	0	0	0
0	0	0	0	0	2	0	0	0
0	0	0	0	0	1	0	0	0
0	0	0	0	0	0	0	0	0
0	0	0	0	0	0	0	0	0
0	0	0	0	0	0	0	0	0
0	0	0	0	0	0	0	0	0
0	0	0	0	0	3	1	0	0

0	0	0	0	0	0	0	0	0
0	0	0	0	0	0	0	0	0
0	0	0	0	0	0	0	0	0
0	0	0	0	0	0	0	0	0
0	0	0	0	0	0	0	0	0
0	0	0	0	0	0	0	0	0
0	0	0	0	0	0	0	0	0
0	0	0	0	0	1	0	0	0
0	0	0	0	0	0	1	0	0
0	0	0	0	0	0	1	0	0
0	0	0	0	0	0	1	0	0
0	0	0	0	0	0	2	0	0
0	0	0	0	0	0	6	0	0
0	0	0	0	0	0	53	0	0
0	0	0	1	0	0	14	0	0
0	0	0	0	0	0	11	0	0
0	0	0	0	0	0	8	0	0
0	0	0	0	0	0	0	0	0
0	0	0	0	0	0	0	0	0
0	0	0	0	0	0	0	0	0
0	0	0	0	0	0	0	0	0
0	0	0	0	0	4	0	0	0
0	0	0	0	0	0	0	0	0
0	0	0	0	0	0	0	0	0
0	0	0	0	0	0	0	0	0
0	0	0	0	0	1	0	0	0
0	0	0	0	0	0	0	0	0
0	0	0	0	0	0	0	0	0
0	0	0	0	0	0	0	0	0

0	0	0	0	0	1	0	0	0
0	0	0	0	0	1	1	0	0
0	0	0	0	0	0	0	0	0
0	0	0	0	0	0	0	0	6
0	0	0	0	0	0	0	0	0
0	0	0	0	0	0	0	0	0
0	0	0	0	0	0	0	0	0
0	0	0	0	0	0	0	0	0
0	0	0	0	0	0	0	0	0
0	0	0	0	0	0	0	0	0
0	0	0	0	0	0	0	0	0
0	0	0	0	0	0	0	0	0
0	0	0	0	0	0	0	0	0
0	0	0	0	0	0	0	0	0
0	0	0	0	0	0	2	0	0
0	0	0	0	0	0	0	0	0
0	0	0	0	0	0	0	0	0
0	0	0	0	0	1	0	0	0
0	0	0	0	0	0	0	0	0
0	0	0	0	0	1	0	0	0
0	0	0	0	0	0	0	0	0
0	0	0	0	0	0	0	0	0
0	0	0	0	0	0	0	0	0
0	0	0	0	0	0	0	0	0
0	0	0	0	0	0	0	0	0

0	0	0	0	0	0	0	0	0
0	0	0	0	0	0	0	0	0
0	0	0	0	0	0	0	0	0
0	0	0	0	0	0	0	0	0
0	0	0	0	0	0	0	0	0
0	0	0	0	0	0	0	0	0
0	0	0	0	0	0	0	0	0
0	0	0	0	0	0	0	0	0
0	0	0	0	0	0	0	0	0
0	0	0	0	0	0	0	0	0
0	0	0	0	0	0	0	0	0
0	0	0	0	0	0	0	0	0
0	0	0	0	0	0	0	0	0
0	0	0	0	0	0	0	0	0
0	0	0	0	0	0	0	0	0
0	0	0	0	0	0	0	0	0
0	0	0	0	0	0	0	0	0
0	0	0	0	0	0	0	0	0
0	0	0	0	0	0	0	0	0
0	0	0	0	0	0	0	0	0
0	0	0	0	0	0	0	0	0
0	0	0	0	0	0	0	0	0
0	0	0	0	0	0	0	0	0
0	0	0	0	0	0	1	0	0

0	0	0	0	0	0	0	0	0
0	0	0	0	0	0	0	0	0
0	0	0	0	0	0	0	0	0
0	0	0	0	0	0	0	0	0
0	0	0	0	0	0	0	0	0

0	0	0	0	0	0	0	2	0
0	0	0	0	0	0	0	0	0
0	0	0	0	0	0	1	0	0
0	0	0	0	0	0	0	0	0
0	0	0	0	0	0	0	0	0
0	0	0	0	0	0	0	0	0
0	0	0	0	0	0	0	0	0
0	0	0	0	0	0	0	0	0
0	0	0	0	0	0	0	0	0
0	0	0	0	0	1	5	0	0
0	0	0	0	0	0	0	0	0
0	0	0	0	0	0	0	0	0
0	0	0	0	0	0	0	0	0
0	0	0	0	0	0	0	0	0
0	0	0	0	0	0	0	0	0
0	0	0	0	0	0	0	0	0
0	0	0	0	0	0	0	0	0
0	0	0	0	0	1	0	0	0
0	0	0	0	0	0	0	0	0
0	0	0	0	0	0	0	0	0
0	0	0	0	0	0	0	0	0
0	0	0	0	0	0	0	0	0
0	0	0	0	0	19	0	0	0
0	0	0	0	0	11	0	0	1
0	0	0	0	0	13	0	0	0
0	0	0	0	0	19	0	0	0
0	0	0	0	0	40	0	0	0
0	0	0	0	0	0	0	0	0
0	0	0	0	0	0	0	0	0
0	0	0	0	0	0	0	0	0
0	0	0	0	0	0	0	0	0
0	0	0	0	0	0	0	0	0

0	0	0	0	0	0	0	0	0
0	0	0	0	0	0	0	0	0
0	0	0	0	0	0	0	0	0
0	0	0	0	0	0	0	0	0
0	0	0	0	0	0	0	0	0
0	0	0	0	0	0	0	0	0
0	0	0	0	0	0	0	0	6
0	0	0	0	0	0	0	0	0
0	0	0	0	0	0	0	0	0
0	0	0	0	0	0	0	0	0
0	0	0	0	0	0	0	0	0
0	0	0	0	0	0	0	0	0
0	0	0	0	0	0	0	0	0
0	0	0	0	0	0	0	0	0
0	0	0	0	0	0	0	0	0
0	0	0	0	0	0	0	0	0
0	0	0	0	0	0	0	0	0
0	0	0	0	0	0	0	0	0
0	0	0	0	0	4	0	0	0
0	0	0	0	0	0	0	0	0

0	0	0	0	0	0	0	0	0
0	0	0	0	0	3	0	0	0
0	0	0	0	0	2	10	0	0
0	0	0	0	0	0	0	0	0
0	0	0	0	0	0	0	0	0
0	0	0	0	0	0	0	0	0
0	0	0	0	0	0	0	0	0
0	0	0	0	0	0	0	0	0
0	0	0	0	0	0	0	0	0
0	0	0	0	0	0	0	0	0
0	0	0	0	0	0	0	0	0
0	0	0	0	0	0	0	0	0
0	0	0	0	0	1	2	0	0
0	0	0	0	0	0	0	0	0
0	0	0	0	0	0	0	0	0
0	0	0	0	0	0	0	0	0
0	0	0	0	0	0	0	0	0
0	0	0	0	0	0	0	0	0
0	0	0	0	0	0	0	0	0
0	0	0	0	0	0	0	0	0
0	0	0	0	0	0	6	0	0
0	0	0	0	0	1	0	0	0
0	0	0	0	0	0	2	0	0
0	0	0	0	0	7	0	0	0
0	0	0	0	0	3	4	0	0
0	0	0	0	0	0	0	0	0
0	0	0	0	0	1	1	0	0
0	0	0	0	0	1	0	0	0

0	0	0	0	0	0	0	0	0
0	0	0	0	0	0	0	0	0
0	0	0	0	0	1	0	0	0
0	0	0	0	0	0	5	0	0
0	0	0	0	0	0	1	0	0
0	0	0	0	0	0	0	0	0
0	0	0	0	0	0	0	0	0
0	0	0	0	0	0	0	0	0
0	0	0	0	0	0	0	0	0
0	0	0	0	0	1	0	0	0
0	0	0	0	0	0	15	0	0
0	0	0	0	0	1	0	0	0
0	0	0	0	0	4	0	0	0
0	0	0	0	0	2	0	0	0
0	0	0	0	0	0	0	0	0
0	0	0	0	1	0	0	0	0
0	0	0	0	0	0	0	0	0
0	0	0	0	0	0	0	0	0
0	0	0	0	0	0	0	0	0
0	0	0	0	0	0	0	0	0
0	0	0	0	0	0	4	0	0
0	0	0	0	0	0	0	0	0
0	0	0	0	0	0	1	0	0
0	0	0	0	0	0	1000	0	0
0	0	0	0	0	0	0	0	0
0	0	0	0	0	0	1	0	0
0	0	0	0	0	1	0	0	0

0	0	0	0	0	0	1	0	0
0	0	0	0	0	0	5	0	0
0	0	0	0	0	1	0	0	0
0	0	0	0	0	0	0	0	1
0	0	0	0	0	0	0	0	0
0	0	0	0	0	0	0	0	0
0	0	0	0	0	0	0	0	0
0	0	0	0	0	1	2	0	0
0	0	0	0	0	0	0	0	0
0	0	0	0	0	0	0	0	0
0	0	0	0	0	0	0	0	0
0	0	0	0	0	0	0	0	0
0	0	0	0	0	0	0	0	0
0	0	0	0	0	0	0	0	0
0	0	0	0	0	0	0	0	0
0	0	0	0	0	0	0	0	0
0	0	0	0	0	0	0	0	0
0	0	0	0	0	0	0	0	0
0	0	0	0	0	0	0	0	0
0	0	0	0	0	0	0	0	0
0	0	0	0	0	0	0	0	0
0	0	0	0	0	0	0	0	0
0	0	0	0	0	0	0	0	0
0	0	0	0	0	0	0	0	0
0	0	0	0	0	0	0	0	0
0	0	0	0	0	0	0	0	0
0	0	0	0	0	0	0	0	0
0	0	0	0	0	1	0	0	0
0	0	0	0	0	0	0	0	0
0	0	0	0	0	0	0	0	0
0	0	0	0	0	0	10	0	0
0	0	0	0	0	0	0	0	0
0	0	0	0	0	0	0	0	0
0	0	0	0	0	0	0	0	0
0	0	0	0	0	0	1	0	0
0	0	0	0	0	0	0	0	0
0	0	0	0	0	0	0	0	0
0	0	0	0	0	0	0	0	0
0	0	0	0	0	0	0	0	0
0	0	0	0	0	0	0	0	0
0	0	0	0	0	2	0	0	0
0	0	0	0	0	0	0	0	0
0	0	0	0	0	0	0	0	0
0	0	0	0	0	0	1	0	0
0	0	0	0	0	6	0	0	0
0	0	0	0	0	0	0	0	0
0	0	0	0	0	0	0	0	0
0	0	0	0	0	0	0	0	0
0	0	0	0	0	0	0	0	0
0	0	0	0	0	0	0	0	0
0	0	0	0	0	0	1	0	0
0	0	0	0	0	0	1	0	0

asp_nid_ta	asp_ustus_ta	asp_niger_ta	asp_sp_ta	asp_terreus_ta	asp_flavus_ta	peni_ta	clado_ta
0	0	0	0	0	0	0	0
0	0	0	0	0	0	1	0
0	0	0	0	0	0	0	0
0	0	0	0	0	0	0	0
0	0	0	0	0	0	0	0
0	0	0	0	0	0	0	0
0	0	0	0	0	0	0	0
0	0	0	0	0	0	0	0
0	0	0	1	0	0	0	0
0	0	0	0	0	0	0	0
0	0	0	0	0	0	0	0
0	0	0	0	0	0	0	0
0	0	0	0	0	0	0	0
0	0	0	0	0	0	0	1
0	0	0	0	0	0	0	0
0	0	0	0	0	0	1000	0
0	0	0	0	0	0	0	0
0	0	0	0	0	0	0	0
0	0	0	0	0	0	0	0
0	0	0	0	0	0	1	0
0	0	0	0	0	0	0	0
0	0	0	0	0	0	0	0
0	0	0	0	0	0	0	0
0	0	0	0	0	0	0	0
0	0	0	0	0	0	0	0
0	0	0	0	0	0	0	0
0	0	0	0	0	0	0	0
0	0	0	0	0	0	0	0
0	0	0	0	0	0	0	0
0	0	0	0	0	0	0	0
0	0	0	0	0	0	0	0
0	0	0	0	0	0	0	0
0	0	0	0	0	0	0	0
0	0	0	0	0	0	0	0
0	0	0	0	0	0	0	0
0	0	0	0	0	0	0	0
0	0	0	0	0	0	0	0
0	0	0	0	0	0	15	0
0	0	0	0	0	0	0	0
0	0	0	0	0	0	0	0
0	0	0	0	0	0	0	0
0	0	0	0	0	0	0	0
0	0	0	0	0	0	0	0
0	0	0	0	0	0	0	0
0	0	0	0	0	0	1	0
0	0	0	0	0	0	0	0
0	0	0	0	0	0	0	0
0	0	0	0	0	0	0	0
0	0	0	0	0	0	0	0
0	0	0	0	0	0	0	0
0	0	0	0	0	0	0	0
0	0	0	0	0	0	0	0
0	0	0	0	0	0	0	0
0	0	0	0	0	0	3	0
0	0	0	0	0	0	0	0

0	0	0	0	0	0	0	0
0	0	0	0	0	0	0	0
0	0	0	0	0	0	0	0
0	0	0	0	0	0	0	0
0	0	0	0	0	0	0	0
0	0	0	0	0	0	0	0
0	0	0	0	0	0	0	0
0	0	0	0	0	0	0	0
0	0	0	0	0	0	0	0
0	0	0	0	0	0	0	0
0	0	0	0	0	0	0	0
0	0	0	0	0	0	0	0
0	0	0	0	0	0	0	0
0	0	0	0	0	0	0	0
0	0	0	0	0	0	0	0
0	0	0	0	0	0	0	0
0	0	0	0	0	0	0	0
0	0	0	0	0	0	0	0
0	0	0	0	0	0	0	0
0	0	0	0	0	0	0	0
0	0	0	0	0	0	0	0
0	0	0	0	0	0	0	0
0	0	0	0	0	0	0	0
0	0	0	0	0	0	0	0
0	0	0	0	0	0	0	0
0	0	0	0	0	0	1	0
0	0	0	0	0	0	0	0
0	0	0	0	0	0	0	0
0	0	0	0	0	0	0	0
0	0	0	0	0	0	0	0
0	0	0	0	0	0	0	0
0	0	0	0	0	0	0	0
0	0	0	0	0	0	0	0
0	0	0	0	0	0	0	0
0	0	0	0	0	0	0	0
0	0	0	0	0	0	0	0

0	0	0	0	0	0	0	0
0	0	0	0	0	0	0	0
0	0	0	0	0	0	1	0
0	0	3	0	0	0	2	6
0	0	0	0	0	0	0	0
0	0	0	0	0	0	1	0
0	0	0	0	0	0	0	0
0	0	0	0	0	0	0	0
0	0	0	0	0	0	0	0
0	0	0	0	0	0	1	0
0	0	0	0	0	0	18	0
0	0	0	0	0	0	0	0
0	0	1	0	0	0	0	0
0	0	0	0	0	0	0	0
0	0	0	0	0	0	1	0
0	0	0	2	0	0	0	0
0	0	0	0	0	0	0	0
0	0	0	0	0	0	0	0
0	0	0	0	0	0	0	0

0	0	0	0	0	0	1	0
0	0	0	0	0	0	0	0
0	0	0	0	0	0	0	0
0	0	0	0	0	0	0	0
0	0	0	0	0	0	0	0
0	0	0	0	0	0	3	0
0	0	0	0	0	0	0	0
0	0	0	0	0	0	0	0
0	0	0	0	0	0	0	0
0	0	0	0	0	0	0	0
0	0	0	0	0	0	0	0
0	0	0	0	0	0	0	0
0	0	0	0	0	0	0	0
0	0	0	0	0	0	0	0
0	0	0	0	0	0	0	0
0	0	0	0	0	0	0	0
0	0	0	0	0	0	0	0
0	0	0	0	0	0	0	0
0	0	0	0	0	0	0	0
0	0	0	0	0	0	0	0
0	0	0	0	0	0	0	0
0	0	0	0	0	0	0	0
0	0	0	0	0	0	0	0
0	0	0	0	0	0	0	0
0	0	0	0	0	0	0	0
0	0	0	0	0	0	0	0
0	0	0	0	0	0	0	0
0	0	0	0	0	0	0	0
0	0	0	0	0	0	0	0
0	0	0	0	0	0	0	0
0	0	0	0	0	0	0	0
0	0	0	0	0	0	0	0
0	0	0	0	0	0	0	0
0	0	0	0	0	0	0	0
0	0	0	0	0	0	0	0
0	0	0	0	0	0	0	0
0	0	0	0	0	0	0	0
0	0	0	0	0	0	0	0
0	0	0	0	0	0	0	0
0	0	0	0	0	0	0	0
0	0	0	0	0	0	0	0
0	0	0	0	0	0	0	0
0	0	0	0	0	0	0	0
0	0	0	0	0	0	0	0
0	0	0	0	0	0	0	0
0	0	0	0	0	0	0	0
0	0	0	0	0	0	0	0
0	0	0	0	0	0	0	0
0	0	0	0	0	0	0	0
0	0	0	0	0	0	0	0
0	0	0	0	0	0	0	0
0	0	0	0	0	0	0	0
0	0	0	0	0	0	0	0
0	0	0	0	0	0	0	0

0	0	0	0	0	0	0	0
0	0	0	0	0	0	0	0
0	0	0	0	0	0	0	0
0	0	0	0	0	0	0	0
0	0	0	0	0	0	0	0
0	0	0	0	0	0	0	0
0	0	0	0	0	0	0	0
0	0	0	0	0	0	0	0
0	0	0	0	0	0	0	0
0	0	0	0	0	0	0	0
0	0	0	0	0	0	0	0
0	0	0	0	0	0	0	0
0	0	0	0	0	0	0	0
0	0	0	0	0	0	0	0

0	0	0	0	0	0	0	0
0	0	0	0	0	0	0	0
0	0	0	0	0	0	10	1
0	0	0	0	0	0	0	0
0	0	0	0	0	0	0	0
0	0	0	1	0	0	0	0
0	0	0	0	0	0	0	0
0	0	0	1	0	0	0	0
0	0	0	0	0	0	0	0

0	0	0	0	0	0	0	0
0	0	0	0	0	0	0	0
0	0	0	0	0	0	0	0
0	0	0	0	0	0	0	0
0	0	0	0	0	0	0	0
0	0	0	0	0	0	0	0
0	0	0	0	0	0	0	0
0	0	0	0	0	0	0	0
0	0	0	0	0	0	0	0
0	0	0	0	0	0	0	0
0	0	0	0	0	0	0	0
0	0	0	0	0	0	0	0
0	0	0	0	0	0	1	0
0	0	0	0	0	0	0	0
0	0	0	0	0	0	0	0
0	0	0	0	0	0	0	0
0	0	0	0	0	0	0	0
0	0	0	0	0	0	0	0
0	0	0	0	0	0	0	0
0	0	0	0	0	0	0	0
0	0	0	0	0	0	0	0
0	0	0	0	0	0	0	0
0	0	0	0	0	0	0	0
0	0	0	0	0	0	0	0
0	0	0	0	0	0	0	0
0	0	0	0	0	0	0	0
0	0	0	0	0	0	0	0
0	0	0	0	0	0	0	0
0	0	0	0	0	0	0	0

0	0	0	0	0	0	0	0
0	0	0	0	0	0	0	0
0	0	0	0	0	0	0	0
0	0	0	0	0	0	0	0
0	0	0	0	0	0	0	0
0	0	0	0	0	0	0	0
0	0	0	0	0	0	0	0
0	0	0	0	0	0	0	0
0	0	0	0	0	0	0	0
0	0	0	0	0	0	0	0
0	0	0	0	0	0	0	0
0	0	0	0	0	0	0	0
0	0	0	0	0	0	0	0
0	0	0	0	0	0	0	0
0	0	0	0	0	0	0	0
0	0	0	0	0	0	0	0
0	0	0	0	0	0	0	0
0	0	0	0	0	0	0	0
0	0	0	0	0	0	0	0
0	0	0	0	0	0	0	0
0	0	0	0	0	0	0	0
0	0	0	0	0	0	0	0
0	0	0	0	0	0	0	0
0	0	0	0	0	0	0	0
0	0	0	0	0	0	0	0
0	0	0	0	0	0	0	0
0	0	0	0	0	0	0	0
0	0	0	0	0	0	0	0
0	0	0	0	0	0	0	0
0	0	0	0	0	0	0	0
0	0	0	0	0	0	0	0
0	0	0	0	0	0	0	0
0	0	0	0	0	0	0	0
0	0	0	0	0	0	0	0
0	0	0	0	0	0	0	0
0	0	0	0	0	0	0	0
0	0	0	0	0	0	0	0
0	0	0	0	0	0	0	0
0	0	0	0	0	0	0	0
0	0	0	0	0	0	0	0
0	0	0	0	0	0	0	0
0	0	0	0	0	0	0	0
0	0	0	0	0	0	0	0
0	0	0	0	0	0	0	0
0	0	0	0	0	0	0	0
0	0	0	0	0	0	0	0
0	0	0	0	0	0	1	0
0	0	0	0	0	0	0	0
0	0	0	0	0	0	0	0
0	0	0	0	0	0	0	0
0	0	0	0	0	0	0	0
0	0	0	0	0	0	0	0
0	0	0	0	0	0	0	0
0	0	0	0	0	0	0	0

0	0	0	0	0	5	0	0
0	0	0	0	0	0	0	0
0	0	0	0	0	0	0	0
0	0	0	0	0	0	0	0
0	0	0	0	0	0	0	0
0	0	0	0	0	0	0	0
0	0	0	0	0	0	0	0
0	0	0	0	0	0	0	0
0	0	0	0	0	0	0	0
0	0	0	0	0	0	0	0
0	0	0	0	0	0	0	0
0	0	0	0	0	0	0	0
0	0	0	0	0	0	0	0
0	0	0	0	0	0	0	0
0	0	0	0	0	0	0	0
0	0	0	0	0	0	0	0
0	0	0	0	0	0	0	0
0	0	0	0	0	0	1	0
0	0	0	0	0	0	0	0
0	0	0	0	0	0	0	0
0	0	0	0	0	0	0	0
0	0	0	0	0	0	0	0
0	0	0	0	0	0	0	0
0	0	0	0	0	0	0	0
0	0	0	0	0	0	0	0
0	0	0	0	0	0	0	0
0	0	0	0	0	0	0	0
0	0	0	0	0	0	0	0
0	0	0	0	0	0	0	0
0	0	0	0	0	0	0	0
0	0	0	0	0	0	0	0
0	0	0	0	0	0	0	0

0	0	0	0	0	0	0	0
0	0	0	0	0	0	0	0
0	0	0	0	0	0	0	0
0	0	0	0	0	0	0	0
0	0	0	0	0	0	0	0
0	0	0	0	0	0	0	0
0	0	0	0	0	0	1	0
0	0	0	0	0	0	0	0
0	0	0	0	0	0	0	0
0	0	0	0	0	0	0	0
0	0	0	0	0	0	0	0
0	0	0	0	0	0	0	0
0	0	0	0	0	0	0	0
0	0	0	2	0	0	1	0
0	0	0	0	0	0	0	0
0	0	0	0	0	0	0	0
0	0	0	0	0	0	0	0
0	0	0	0	0	0	0	0
0	0	0	0	0	0	0	0
0	0	0	0	0	0	0	0

0	0	0	10	0	0	13	0
0	0	0	0	0	0	0	0
0	0	0	0	0	0	0	0
0	0	0	0	0	0	0	0
0	0	0	0	0	0	0	0
0	0	0	0	0	0	0	0
0	0	0	0	0	0	0	0
0	0	0	0	0	0	0	0
0	0	0	0	0	0	0	0
0	0	0	0	0	0	0	0
0	0	0	0	0	0	0	0
0	0	0	0	0	0	0	0
0	0	0	0	0	0	0	0
0	0	0	0	0	0	0	0
0	0	0	0	0	0	0	0
0	0	0	0	0	0	0	0
0	0	0	0	0	0	0	0
0	0	0	0	0	0	0	0
0	0	0	0	0	0	0	0
0	0	0	0	0	0	0	0
0	0	0	0	0	0	0	0
0	0	0	0	0	0	0	0
0	0	0	0	0	0	0	0
0	0	0	0	0	0	0	0
0	0	0	0	0	0	0	0
0	0	0	1	0	0	0	0
0	0	0	0	0	0	0	0
0	0	0	0	0	0	0	0

0	0	0	0	0	0	0	0
0	0	0	0	0	0	0	0
0	0	0	0	0	0	0	0
0	0	0	0	0	0	0	0
0	0	0	0	0	0	0	0
0	0	0	0	0	0	0	0
0	0	0	0	0	0	0	0
0	0	0	0	0	0	0	0
0	0	0	0	0	0	0	0
0	0	0	0	0	0	0	0
0	0	0	0	0	0	0	0
0	0	0	0	0	0	0	0
0	0	0	0	0	0	0	0
0	0	0	0	0	0	0	0
0	0	0	0	0	0	0	0
0	0	0	0	0	0	0	0
0	0	0	0	0	0	0	0
0	0	0	0	0	0	0	0
0	0	0	0	0	0	0	0
0	0	0	0	0	0	0	0
0	0	0	0	0	0	0	0
0	0	0	0	0	0	0	0
0	0	0	0	0	0	0	0
0	0	0	0	0	0	0	0
0	0	0	0	0	0	0	0
0	0	0	0	0	0	0	0
0	0	0	0	0	0	0	0

0	0	0	0	0	0	0	0
0	0	0	0	0	0	0	0
0	0	0	0	0	0	0	0
0	0	0	0	0	0	7	0
0	0	0	0	0	0	0	0
0	0	0	0	0	0	0	0
0	0	0	0	0	0	0	0
0	0	0	0	0	0	0	0
0	0	0	0	0	0	0	0
0	0	0	0	0	0	0	0
0	0	0	0	0	0	0	0
0	0	0	0	0	0	0	1
0	0	0	0	0	0	0	0
0	0	0	0	0	0	0	0
0	0	0	0	0	0	0	0
0	0	0	0	0	0	0	0
0	0	0	0	0	0	0	0
0	0	0	0	0	0	0	0
0	0	0	0	0	0	0	0
0	0	0	0	0	0	0	0
0	0	0	0	0	0	0	0
0	0	0	0	0	0	0	0
0	0	0	0	0	0	0	0
0	0	0	0	0	0	5	0
0	0	0	0	0	0	0	0
0	0	0	0	0	0	135	0
0	0	0	0	0	0	129	0
0	0	0	0	0	0	0	0
0	0	0	0	0	0	0	0
0	0	0	0	0	0	0	0
0	0	0	0	0	0	1	1
0	0	0	0	0	0	0	0
0	0	0	0	0	0	0	0
0	0	0	0	0	0	0	0
0	0	0	0	0	0	0	0
0	0	0	0	0	0	0	0
0	0	0	0	0	0	0	0
0	0	0	0	0	0	1	0
0	0	0	0	0	0	1	0
0	0	0	0	0	0	0	0
0	0	0	0	0	0	0	0
0	0	0	0	0	0	0	0
0	0	0	0	0	0	1	0
0	0	0	0	0	0	0	0
0	0	0	0	0	0	0	0
0	0	0	0	0	0	0	0
0	0	0	0	0	0	0	0
0	0	0	0	0	0	0	0
0	0	0	0	0	0	0	0
0	0	0	0	0	0	0	0
0	0	0	0	0	0	0	0
0	0	0	0	0	0	0	0

alterna_ta	ulocla_ta	chaeto_ta	scopula_ta	acremo_ta	fusa_ta	rhizo_ta	mucor_ta	geotri_ta
0	0	0	0	0	0	0	0	0
0	0	0	0	0	0	0	0	0
0	0	0	0	0	0	0	0	0
0	0	0	0	0	0	0	0	0
0	0	0	0	0	0	0	0	0
0	0	0	0	0	0	0	0	0
0	0	0	0	0	0	0	0	0
0	0	0	0	0	0	0	0	0
0	0	0	0	0	0	0	0	0
0	0	0	0	0	0	0	0	0
0	0	0	0	0	0	0	0	0
0	0	0	0	0	0	0	0	0
0	0	0	0	0	0	0	0	0
0	0	0	0	0	0	0	0	0
0	0	0	0	0	0	0	0	0
0	0	0	0	0	0	0	0	0
0	0	0	0	0	0	0	0	0
0	0	0	0	0	0	0	0	0
0	0	0	0	0	0	0	0	0
0	0	0	0	0	0	0	0	0
0	0	0	0	0	0	0	0	0
0	0	0	0	0	0	0	0	0
0	0	0	0	0	0	0	0	0
0	0	0	0	0	0	0	0	0
0	0	0	0	0	0	0	0	0
0	0	0	0	0	0	0	0	0
0	0	0	0	0	0	0	0	0
0	0	0	0	0	0	0	0	0
0	0	0	0	0	0	0	0	0
0	0	0	0	0	0	0	0	0
0	0	0	0	0	0	0	0	0
0	0	0	0	0	0	0	0	0
0	0	0	0	0	0	0	0	0
0	0	0	0	0	0	0	0	0
0	0	0	0	0	0	0	0	0
0	0	0	0	0	0	0	0	0
0	0	0	0	0	0	0	0	0
0	0	0	0	0	0	0	0	0
0	0	0	0	0	0	0	0	0
0	0	0	0	0	0	0	0	0
0	0	0	0	0	0	0	0	0
0	0	0	0	0	0	0	0	0
0	0	0	0	0	0	0	0	0
0	0	0	0	0	0	0	0	0
0	0	0	0	0	0	0	0	0
0	0	0	0	0	0	0	0	0
0	0	0	0	0	0	0	0	0
0	0	0	0	0	0	0	0	0
0	0	0	0	0	0	0	0	0
0	0	0	0	0	0	0	0	0
0	0	0	0	0	0	0	0	0
0	0	0	0	0	0	0	0	0
0	0	0	0	0	0	0	0	0
0	0	0	0	0	0	0	0	0
0	0	0	0	0	0	0	0	0

0	0	0	0	0	0	0	0	0
0	0	0	0	0	0	0	0	0
0	0	0	0	0	0	0	0	0
0	0	0	0	0	0	0	0	0
0	0	0	0	0	0	0	0	0
0	0	0	0	0	0	0	0	0
0	0	0	0	0	0	0	0	0
0	0	0	0	0	0	0	0	0
0	0	0	0	0	0	0	0	0
0	0	0	0	0	0	0	0	0
0	0	0	0	0	0	0	0	0
0	0	0	0	0	0	0	0	0
0	0	0	0	0	0	0	0	0
0	0	0	0	0	0	0	0	0
0	0	0	0	0	0	0	0	0
0	0	0	0	0	0	0	0	0
0	0	0	0	0	0	0	0	0
0	0	0	0	0	0	0	0	0
0	0	0	0	0	0	0	0	0
0	0	0	0	0	0	0	0	0
0	0	0	0	0	0	0	0	0
0	0	0	0	0	0	0	0	0
0	0	0	0	0	0	0	0	0
0	0	0	0	0	0	0	0	0
0	0	0	0	0	0	0	0	0
0	0	0	0	0	0	0	0	0
0	0	0	0	0	0	0	0	0
0	0	0	0	0	0	0	0	0
0	0	0	0	0	0	0	0	0
0	0	0	0	0	0	0	0	0
0	0	0	0	0	0	0	0	0
0	0	0	0	0	0	0	0	0
0	0	0	0	0	0	0	0	0
0	0	0	0	0	0	0	0	0
0	0	0	0	0	0	0	0	0
0	0	0	0	0	0	0	0	0
0	0	0	0	0	0	0	0	0
0	0	0	0	0	0	0	0	0
0	0	0	0	0	0	0	0	0
5	0	0	0	0	0	0	0	0
0	0	0	0	0	0	0	0	0
0	0	0	0	0	0	0	0	0
0	0	0	0	0	0	0	0	0
0	0	0	0	0	0	0	0	0
0	0	0	0	0	0	0	0	0
0	0	0	0	0	0	0	0	0
0	0	0	0	0	0	0	0	0
0	0	0	0	0	0	0	0	0
0	0	0	0	0	0	0	0	0
0	0	0	0	0	0	0	0	0
0	0	0	0	0	0	0	0	0
0	0	0	0	0	0	0	0	0
0	0	0	0	0	0	0	0	0
0	0	1	0	0	0	0	0	0
0	0	0	0	0	0	0	0	0

0	0	0	0	0	0	0	0	0
0	0	0	0	0	0	0	0	0
0	0	0	0	0	0	0	0	0
0	0	0	0	0	0	0	0	0
0	0	1	0	0	0	0	0	0
0	0	0	0	0	0	0	0	0
0	0	0	0	0	0	0	0	0
0	0	0	0	0	0	0	0	0
0	0	0	0	0	0	0	0	0
0	0	0	0	0	0	0	0	0
0	0	0	0	0	0	0	0	0
0	0	0	0	0	0	0	0	0
0	0	0	0	0	0	0	0	0
0	0	0	0	0	0	0	0	0
0	0	0	0	0	0	0	0	0
0	0	0	0	0	0	0	0	0
0	0	0	0	0	0	0	0	0
0	0	0	0	0	0	0	0	0
0	0	0	0	0	0	0	0	0
0	0	0	0	0	0	0	0	0
0	0	0	0	0	0	0	0	0
0	0	0	0	0	0	0	0	0
0	0	0	0	0	0	0	0	0
0	0	0	0	0	0	0	0	0
0	0	0	0	0	0	0	0	0
0	0	0	0	0	0	0	0	0
0	0	0	0	0	0	0	0	0
0	0	0	0	0	0	0	0	0
0	0	0	0	0	0	0	0	0
0	0	0	0	0	0	0	0	0
0	0	0	0	0	0	0	0	0
0	0	0	0	0	0	0	0	0
0	0	0	0	0	0	0	0	0
0	0	0	0	0	0	0	0	0
0	0	0	0	0	0	0	0	0
0	0	0	0	0	0	0	0	0
0	0	0	0	0	0	0	0	0
0	0	0	0	0	0	0	0	0
0	0	0	0	0	0	0	0	0
0	0	0	0	0	0	0	0	0
0	0	0	0	0	0	0	0	0
0	0	0	0	0	0	0	0	0
0	0	0	0	0	0	0	0	0
0	0	0	0	0	0	0	0	0
0	0	0	0	0	0	0	0	0
0	0	0	0	0	0	0	0	0
0	0	0	0	0	0	0	0	0
0	0	0	0	0	0	0	0	0
0	0	0	0	0	0	0	0	0
0	0	0	0	0	0	0	0	0
0	0	0	0	0	0	0	0	0
0	0	0	0	0	0	0	0	0
0	0	0	0	0	0	0	0	0
0	0	0	0	0	0	0	0	0

2	0	0	0	0	0	0	0	0
0	0	0	0	0	0	0	0	0
0	0	0	0	0	0	0	0	0
0	0	0	0	0	0	0	0	0
0	0	0	0	0	0	0	0	0
0	0	0	0	0	0	0	0	0
0	0	0	0	0	0	0	0	0
0	0	0	0	0	0	0	0	0
0	0	0	0	0	0	0	0	0
0	0	0	0	0	0	0	0	0
0	0	0	0	0	0	0	0	0
0	0	0	0	0	0	0	0	0
0	0	0	0	0	0	0	0	0
0	0	0	0	0	0	0	0	0
0	0	0	0	0	0	0	0	0
0	0	0	0	0	0	0	0	0
0	0	0	0	0	0	0	0	0
0	0	0	0	0	0	0	0	0
0	0	0	0	0	0	0	0	0
0	0	0	0	0	0	0	0	0
0	0	0	0	0	0	0	0	0
0	0	0	0	0	0	0	0	0
0	0	0	0	0	0	0	0	0
0	0	0	0	0	0	0	0	0
0	0	0	0	0	0	0	0	0
0	0	0	0	0	0	0	0	0
0	0	0	0	0	0	0	0	0
0	0	0	0	0	0	0	0	0
0	0	0	0	0	0	0	0	0
0	0	0	0	0	0	0	0	0
0	0	0	0	0	0	0	0	0
0	0	0	0	0	0	0	0	0
0	0	0	0	0	0	0	0	0
0	0	0	0	0	0	0	0	0
0	0	0	0	0	0	0	0	0
0	0	0	0	0	0	0	0	0
0	0	0	0	0	0	0	0	0
0	0	0	0	0	0	0	0	0
0	0	0	0	0	0	0	0	0
0	0	0	0	0	0	0	0	0
0	0	0	0	0	0	0	0	0
0	0	0	0	0	0	0	0	0
0	0	0	0	0	0	0	0	0
0	0	0	0	0	0	0	0	0
0	0	0	0	0	0	0	0	0
0	0	0	1	0	0	0	0	0
0	0	0	0	0	0	0	0	0
0	0	0	0	0	0	0	0	0
0	0	0	0	0	0	0	0	0
0	0	0	0	0	0	0	0	0
0	0	0	0	0	0	0	0	0
0	0	0	0	0	0	0	0	0

0	0	0	0	0	0	0	0	0
0	0	0	0	0	0	0	0	0
0	0	0	0	0	0	0	0	0
0	0	0	0	0	0	0	0	0
0	0	0	0	0	0	0	0	0
0	0	0	0	0	0	0	0	0
0	0	0	0	0	0	0	0	0
0	0	0	0	0	0	0	0	0
0	0	0	0	0	0	0	0	0
0	0	0	0	0	0	0	0	0
0	0	0	0	0	0	0	0	0
0	0	0	0	0	0	0	0	0
0	0	0	0	0	0	0	0	0
0	0	0	0	0	0	0	0	0
0	0	0	0	0	0	0	0	0
0	0	0	0	0	0	0	0	0
0	0	0	0	0	0	0	0	0
0	0	0	0	0	0	0	0	0
0	0	0	0	0	0	0	0	0
0	0	0	0	0	0	0	0	0
0	0	0	0	0	0	0	0	0
0	0	0	0	0	0	0	0	0
0	0	0	0	0	0	0	0	0
0	0	0	0	0	0	0	0	0
0	0	0	0	0	0	0	0	0

0	0	0	0	0	0	0	0	0
0	0	0	0	0	0	0	0	0
0	0	0	0	0	0	0	0	0
0	0	0	0	0	0	0	0	0
0	0	0	0	0	0	0	0	0
0	0	0	0	0	0	0	0	0
0	0	0	0	0	0	0	0	0
0	0	0	0	0	0	0	0	0
0	0	0	0	0	0	0	0	0
0	0	0	0	0	0	0	0	0
0	0	0	0	0	0	0	0	0
0	0	0	0	0	0	0	0	0
0	0	0	0	0	0	0	0	0
0	0	0	0	0	0	0	0	0
0	0	0	0	0	0	0	0	0
0	0	0	0	0	0	0	0	0
0	0	0	0	0	0	0	0	0
0	0	0	0	0	0	0	0	0
0	0	0	0	0	0	0	0	0
0	0	0	0	0	0	0	0	0
0	0	0	0	0	0	0	0	0
0	0	0	0	0	0	0	0	0
0	0	0	0	0	0	0	0	0
0	0	0	0	0	0	0	0	0

0	0	0	0	0	0	0	0	0
0	0	0	0	0	0	0	0	0
0	0	0	0	0	0	0	1	0
0	0	0	0	0	0	0	0	0
0	0	0	0	0	0	0	0	0

0	0	0	0	0	0	0	0	0
0	0	0	0	0	0	0	0	0
0	0	0	0	0	0	0	0	0
0	0	0	0	0	0	0	0	0
0	0	0	0	0	0	0	0	0
0	0	0	0	0	0	0	0	0
0	0	0	0	0	0	0	0	0
0	0	0	0	0	0	0	0	0
0	0	0	0	0	0	0	0	0
0	0	0	0	0	0	0	0	0
0	0	0	0	0	0	0	0	0
0	0	0	0	0	0	0	0	0
0	0	0	0	0	0	0	0	0
0	0	0	0	0	0	0	0	0
0	0	0	0	0	0	0	0	0
0	0	0	0	0	0	0	0	0
0	0	0	0	0	0	0	0	0
0	0	0	0	0	0	0	0	0
0	0	0	0	0	0	0	0	0
0	0	0	0	0	0	0	0	0
0	0	0	0	0	0	0	0	0
0	0	0	0	0	0	0	0	0
0	0	0	0	0	0	0	0	0
0	0	0	0	0	0	0	0	0
0	0	0	0	0	0	0	0	0
0	0	0	0	0	0	0	0	0
0	0	0	0	0	0	0	0	0
0	0	0	0	0	0	0	0	0
0	0	0	0	0	0	0	0	0
0	0	0	0	0	0	0	0	0
0	0	0	0	0	0	0	0	0
0	0	0	0	0	0	0	0	0

0	0	0	0	0	0	0	0	0
0	0	0	0	0	0	0	0	0
0	0	0	0	0	0	0	0	0
0	0	0	0	0	0	0	0	0
0	0	0	0	0	0	0	0	0
0	0	0	0	0	0	0	0	0
0	0	0	0	0	0	0	0	0
0	0	0	0	0	0	0	0	0
0	0	0	0	0	0	0	0	0
0	0	0	0	0	0	0	0	0
0	0	0	0	0	0	0	0	0
0	0	0	0	0	0	0	0	0
0	0	0	0	0	0	0	0	0
0	0	0	0	0	0	0	0	0
0	0	0	0	0	0	0	0	0
0	0	0	0	0	0	0	0	0
0	0	0	0	0	0	0	0	0
0	0	0	0	0	0	0	0	0
0	0	0	0	0	0	0	0	0
0	0	0	0	0	0	0	0	0

0	0	0	0	0	0	0	0	0
0	0	0	0	0	0	0	0	0
0	0	0	0	0	0	0	0	0
0	0	0	0	0	0	0	0	0
0	0	0	0	0	0	0	0	0
0	0	0	0	0	0	0	0	0
0	0	0	0	0	0	0	0	0
0	0	0	0	0	0	0	0	0
0	0	0	0	0	0	0	0	0
0	0	0	0	0	0	0	0	0
0	0	0	0	0	0	0	0	0
0	0	0	0	0	0	0	0	0
0	0	0	0	0	0	0	0	0
0	0	0	0	0	0	0	0	0
0	0	0	0	0	0	0	0	0
0	0	0	0	0	0	0	0	0
0	0	0	0	0	0	0	0	0
0	0	0	0	0	0	0	0	0
0	0	0	0	0	0	0	0	0
0	0	0	0	0	0	0	0	0
0	0	0	0	0	0	0	0	0
0	0	0	0	0	0	0	0	0
0	0	0	0	0	0	0	0	0
0	0	0	0	0	0	0	0	0
0	0	0	0	0	0	0	0	0
0	0	0	0	0	1	0	0	0
0	0	0	0	0	0	0	0	0
0	0	0	0	0	0	0	0	0

0	0	0	0	0	0	0	0	0
0	0	0	0	0	0	0	0	0
0	0	0	0	0	0	0	0	0
0	0	0	0	0	0	0	0	0
0	0	0	0	0	0	0	0	0
0	0	0	0	0	0	0	0	0
0	0	0	0	0	0	0	0	0
0	0	0	0	0	0	0	0	0
0	0	0	0	0	0	0	0	0
0	0	0	0	0	0	0	0	0
0	0	0	0	0	0	0	0	0
0	0	0	0	0	0	0	0	0
0	0	0	0	0	0	0	0	0
0	0	0	0	0	0	0	0	0
0	0	0	0	0	0	0	0	0
0	0	0	0	0	0	0	0	0
0	0	0	0	0	0	0	0	0
0	0	0	0	0	0	0	0	0
0	0	0	0	0	0	0	0	0
0	0	0	0	0	0	0	0	0
0	0	0	0	0	0	0	0	0
0	0	0	0	0	0	0	0	0
0	0	0	0	0	0	0	0	0
0	0	0	0	0	0	0	0	0
0	0	0	0	0	0	0	0	0
0	0	0	0	0	0	0	0	0
0	0	0	0	0	0	0	0	0

0	0	0	0	0	0	0	0	0
0	0	0	0	0	0	0	0	0
0	0	0	0	0	0	0	0	0
0	0	0	0	0	0	0	0	0
0	0	0	0	0	0	0	0	0
0	0	0	0	0	0	0	0	0
0	0	0	0	0	0	0	0	0
0	0	0	0	0	0	0	0	0
0	0	0	0	0	0	0	0	0
0	0	0	0	0	0	0	0	0
0	0	0	0	0	0	0	0	0
0	0	0	0	0	0	0	0	0
0	0	0	0	0	0	0	0	0
0	0	0	0	0	0	0	0	0
0	0	0	0	0	0	0	0	0
0	0	0	0	0	0	0	0	0
0	0	0	0	0	0	0	0	0
0	0	0	0	0	0	0	0	0
0	0	0	0	0	0	0	0	0
0	0	0	0	0	0	0	0	0
0	0	0	0	0	0	0	0	0
0	0	0	0	0	0	0	0	0
0	0	0	0	0	0	0	0	0
1	0	0	0	0	0	0	0	0
0	0	0	0	0	0	0	0	0
0	0	0	0	0	0	0	0	0
0	0	0	0	0	0	0	0	0
0	0	0	0	0	0	0	0	0
0	0	0	0	0	0	0	0	0
0	0	0	0	0	0	0	0	0
0	0	0	0	0	0	0	0	0
0	0	0	0	0	0	0	0	0
0	0	0	0	0	0	0	0	0
0	0	0	0	0	0	0	0	0
0	0	0	0	0	0	0	0	0
0	0	0	0	0	0	0	0	0
0	0	0	0	0	0	0	0	0
0	0	0	0	0	0	0	0	0
0	0	0	0	0	0	0	0	0
0	0	0	0	0	0	0	0	0
0	0	0	0	0	0	0	0	0
0	0	0	0	0	0	0	0	0
0	0	0	0	0	0	0	0	0
0	0	0	0	0	0	0	0	0
0	0	0	0	0	0	0	0	0
0	0	0	0	0	0	0	0	0
0	0	0	0	0	0	0	0	0
0	0	0	0	0	0	0	0	0
0	0	0	0	0	0	0	0	0
0	0	0	0	0	0	0	0	0
0	0	0	0	0	0	0	0	0
0	0	0	0	0	0	0	0	0

trichoderm_ta	paecilo_ta	autres_ta	csd_ta	levure_ta	asp_fumi_ra	sct_nid_ra	asp_nid_ra
0	0	0	0	0	0	0	0
0	0	0	0	0	0	0	0
0	0	0	0	0	0	6	0
0	0	0	0	0	0	0	0
0	0	0	0	0	0	0	0
0	0	0	0	0	0	1	0
0	0	0	0	0	0	0	0
0	0	0	0	0	0	0	0
0	0	0	0	0	0	0	0
0	0	0	0	0	0	0	0
0	0	0	0	0	0	6	0
0	0	0	0	0	0	0	0
0	0	0	0	0	0	0	0
0	0	0	0	0	0	0	0
0	0	0	0	1	0	0	0
0	0	0	0	1000	0	0	0
0	0	0	0	0	0	10	0
0	0	0	0	0	0	0	1000
0	0	0	0	1	0	0	0
0	0	0	0	0	0	0	1000
0	0	0	0	0	0	0	0
0	0	0	0	0	0	1	0
0	0	0	0	6	0	9	0
0	0	0	0	0	0	2	0
0	0	0	1	3	0	1	0
0	0	0	0	1	0	1	0
0	0	0	0	0	0	0	0
0	0	0	0	0	0	0	0
0	0	0	0	0	0	0	0
0	0	0	0	0	0	1000	0
0	0	0	0	0	0	0	0
0	0	0	0	0	0	0	0
0	0	0	0	0	0	2	0
0	0	0	0	0	0	0	0
0	0	0	1	0	0	0	0
0	0	0	0	0	0	0	0
0	0	0	1	0	0	0	0
0	0	0	7	0	0	0	0
0	0	0	0	0	0	0	0
0	0	0	0	0	0	3	0
0	0	0	0	0	0	0	0
0	0	0	0	0	0	0	0
0	0	0	0	0	0	0	0
0	0	0	0	11	0	0	0
0	0	0	0	4	0	0	0
0	0	0	0	0	0	0	0
0	0	0	0	0	0	0	0
0	0	0	1	0	0	0	0
0	0	0	0	1	0	0	0
0	0	0	0	0	0	0	0
0	0	0	0	1	0	0	0
0	0	0	0	0	0	0	0
0	0	0	0	0	0	0	0
0	0	0	0	0	0	0	0
0	0	0	0	0	0	0	0

0	0	0	0	0	0	0	0
0	0	0	0	0	0	0	0
0	0	0	0	0	0	0	0
0	0	0	0	0	0	0	0
0	0	0	0	0	0	0	0
0	0	0	0	0	0	0	0
0	0	0	0	0	0	1	0
0	0	0	0	0	0	0	0
0	0	0	0	0	0	0	0
0	0	0	0	0	0	0	0
0	0	0	0	0	0	0	0
0	0	0	0	0	0	1	0
0	0	0	0	0	0	0	0
0	0	0	0	0	0	0	0
0	0	0	0	0	0	0	0
0	0	0	0	0	0	0	0
0	0	0	0	0	0	0	0
0	0	0	0	0	0	0	0
0	0	0	0	7	0	0	0
0	0	0	0	3	0	0	0
0	0	0	0	0	0	0	0
0	0	0	0	0	0	0	0
0	0	0	0	0	0	0	0
0	0	0	1	1	0	0	0
0	0	0	0	0	0	0	0
0	0	0	0	0	0	0	0
0	0	0	0	0	0	0	0
0	0	0	0	0	0	0	0
0	0	0	0	0	0	11	0
0	0	0	0	0	0	0	0
0	0	0	0	0	0	0	0
0	0	0	0	1	0	0	0
0	0	0	0	0	0	0	0
0	0	0	0	0	0	0	0
0	0	0	0	0	0	15	0
0	0	0	0	1	0	7	0
0	0	0	0	0	0	0	0
0	0	0	0	0	0	0	0
0	0	0	0	494	0	0	0
0	0	0	2	5	0	17	0
0	0	0	0	0	0	26	0
0	0	0	2	0	0	0	0
0	0	0	0	0	0	0	0
0	0	0	0	0	0	0	0
0	0	0	0	0	0	0	0
0	0	0	0	43	0	0	0
0	0	0	0	1000	0	0	0
0	0	0	0	0	0	0	0
0	0	0	0	0	0	0	0
0	0	0	0	0	0	0	0
0	0	0	0	0	0	0	0
0	0	0	0	0	0	0	0
0	0	0	0	0	0	0	0
0	0	0	0	23	0	22	0
0	0	0	0	0	0	0	0

0	0	0	0	0	0	2	0
0	0	0	0	0	0	0	0
0	0	0	0	0	0	0	0
0	0	0	0	0	0	0	0
0	0	0	0	0	0	0	0
0	0	0	0	1	0	0	0
0	0	0	0	0	0	0	0
0	0	0	0	0	0	0	0
0	0	0	0	0	0	0	0
0	0	0	0	2	0	0	0
0	0	0	0	0	0	0	0
0	0	0	0	1	0	0	0
0	0	0	0	0	0	0	0
0	0	0	0	0	0	0	0
0	0	0	4	0	0	0	0
0	0	0	0	208	0	0	0
0	108	0	1	25	0	0	0
0	0	0	0	0	0	1	0
0	0	0	0	0	0	2	0
0	0	0	0	0	0	0	0
0	0	0	0	4	0	0	0
0	0	0	0	0	0	0	0
0	0	0	0	0	0	0	0
0	0	0	0	0	0	0	0
0	0	0	0	92	0	0	0
0	0	0	0	0	0	0	0
0	0	0	0	0	0	0	0
0	0	0	0	0	0	0	0
0	0	0	0	0	0	0	0
0	0	0	0	0	0	0	0
0	0	0	0	0	0	0	0
0	0	0	0	0	0	0	0
0	0	0	0	0	0	0	0
0	0	0	0	0	0	0	0
0	0	0	0	0	0	1	0
0	0	0	0	0	0	1	0
0	0	0	0	0	0	0	0
0	0	0	0	0	0	0	0
0	0	0	0	0	0	0	0
0	0	0	0	0	0	0	0
0	0	0	1	0	0	0	0
0	0	0	0	0	0	0	0
0	0	0	0	0	0	0	0
0	0	0	0	0	0	1	0
0	0	0	0	0	0	0	0
0	0	0	0	0	0	0	0
0	0	0	0	0	0	0	0
0	0	0	0	0	0	0	0
0	0	0	0	0	0	0	0
0	0	0	0	0	0	0	0
0	0	0	0	0	0	0	0
0	0	0	0	1	0	0	0
0	0	0	2	0	1	0	0
0	0	0	1	0	0	1	0

0	0	0	1	0	0	1	0
0	0	0	0	53	0	0	0
0	0	0	1	0	0	4	0
0	0	0	0	0	0	0	0
0	0	0	0	0	0	0	0
0	0	0	0	0	0	0	0
0	0	0	1	0	0	0	0
0	0	0	1	0	0	0	0
0	0	0	1	9	0	0	0
0	0	0	0	0	0	0	0
0	0	0	0	0	0	0	0
0	0	0	0	0	0	0	0
0	0	0	0	7	0	0	0
0	0	0	0	0	0	1	0
0	0	0	0	0	0	0	0
0	0	0	0	0	0	0	0
0	0	0	2	259	0	0	0
0	0	0	0	0	0	0	0
0	0	0	0	0	0	0	0
0	0	0	0	0	0	0	0
0	0	0	0	0	0	0	0
0	0	0	0	0	0	10	0
0	0	0	0	0	0	26	0
0	0	0	0	0	0	0	0
0	0	0	0	0	0	0	0
0	0	0	0	0	0	0	0
0	0	0	0	0	0	0	0
0	0	0	0	0	0	3	0
0	0	0	0	0	0	0	0
0	0	0	0	0	0	0	0
0	0	0	0	2	0	0	0
0	0	0	0	0	0	0	0
0	0	0	1	0	0	0	0
0	0	0	0	0	0	0	0
0	0	0	0	24	0	1	0
0	0	0	0	0	0	0	0
0	0	0	0	14	0	0	0
0	0	0	0	2	0	0	0
0	0	0	0	0	0	0	0
0	0	0	0	3	0	2	0
0	0	0	0	0	0	0	0
0	0	0	0	0	0	0	0
0	0	0	0	0	0	0	0
0	0	0	0	0	0	0	0
0	0	0	2	1	0	0	0
0	0	0	0	0	0	2	0
0	0	0	0	0	0	0	0
0	0	0	0	0	0	0	0
0	0	0	0	0	0	0	0
0	0	0	0	0	0	0	0
0	0	0	0	0	0	0	0
0	0	0	0	0	0	0	0

0	0	0	0	0	0	0	0
0	0	0	0	0	0	3	0
0	0	0	0	0	0	0	0
0	0	0	0	0	0	0	0
0	0	0	0	0	0	0	0
0	0	0	0	0	0	0	0
0	0	0	0	0	0	1	0
0	0	0	1	0	0	1	0
0	0	0	0	0	0	0	0
0	0	0	0	0	0	1	0
0	0	0	0	0	0	0	0
0	0	0	0	0	0	0	0
0	0	0	0	0	0	0	0
0	0	2	0	0	0	0	0
0	0	0	0	0	0	0	0
0	0	0	0	1000	0	0	0
0	0	0	0	0	0	0	0
0	0	0	0	0	0	0	0
0	0	0	0	0	0	0	0
0	0	0	0	0	0	0	0
0	0	0	0	0	0	1	0
0	0	0	0	0	0	0	0
0	0	0	0	0	0	0	0
0	0	0	1	0	0	0	0
0	0	0	0	0	0	0	0

0	0	0	0	0	0	0	0
0	0	0	0	0	0	0	0
0	0	0	0	0	0	0	0
0	0	0	0	0	0	0	0
0	0	0	0	0	0	1	0
0	0	0	0	0	0	0	0
0	0	0	0	0	0	0	0
0	0	0	0	0	0	0	0
0	0	0	0	0	0	0	0
0	0	0	0	0	0	0	0
0	0	0	0	0	0	0	0
0	0	0	0	0	0	1	0
0	0	0	1	0	0	0	0
0	0	0	0	0	0	0	0
0	0	0	0	0	0	0	0
0	0	0	1	0	0	0	0
0	0	0	0	0	0	0	0
0	0	0	0	0	0	0	0
0	0	0	0	0	0	0	0
0	0	0	0	0	0	0	0
0	0	0	0	0	0	0	0
0	0	0	0	0	0	0	0
0	0	0	0	0	0	0	0
0	0	0	0	0	0	0	0

0	0	0	0	0	0	0	0
0	0	0	0	0	0	0	0
0	0	0	0	0	0	0	0
0	0	0	1	0	0	0	0
0	0	0	0	0	0	0	0

0	0	0	0	0	1	0	0
0	0	0	0	0	0	0	0
0	0	0	0	0	0	0	0
0	0	0	0	0	0	0	0
0	0	0	0	0	0	0	0
0	0	0	0	0	0	0	0
0	0	0	0	0	0	0	0
0	0	0	0	0	0	0	0
0	0	0	0	0	1	1	0
0	0	0	2	0	0	0	0
0	0	0	0	0	0	0	0
0	0	0	1	1	0	1	0
0	0	0	0	0	0	0	0
0	0	0	0	0	0	0	0
0	0	0	0	0	0	0	0
0	0	0	0	0	0	0	0
0	0	0	0	0	0	0	0
0	0	0	0	0	0	0	0
0	0	0	0	0	0	42	0
0	0	1	0	0	0	0	0
0	0	0	0	0	0	0	0
0	0	0	0	0	0	0	0
0	0	0	0	1	0	2	0
0	0	0	43	0	0	2	0
0	0	0	0	0	0	14	0
0	0	0	0	21	0	0	0
0	0	0	0	0	0	0	0
0	0	0	0	0	0	0	0
0	0	0	0	0	0	0	0
0	0	0	0	0	0	0	0
0	0	0	0	0	0	0	0
0	0	0	0	0	0	0	0

0	0	0	0	0	0	0	0
0	0	0	0	0	0	0	0
0	0	0	0	0	0	0	0
0	0	0	0	0	0	0	0
0	0	0	1	1	0	0	0
0	0	0	0	0	0	0	0
0	0	0	0	1	0	0	0
0	0	0	0	0	0	0	0
0	0	0	0	0	0	0	0
0	0	0	0	0	0	0	0
0	0	0	0	0	0	0	0
0	0	0	0	0	0	0	0
0	0	0	0	0	0	1	0
0	0	0	0	0	0	0	0
0	0	0	0	0	0	0	0
0	0	0	0	0	0	0	0
0	0	0	0	0	0	0	0
0	0	0	0	0	0	1	0
0	0	0	0	0	0	0	0
0	0	0	0	1	0	0	0

0	0	0	1	0	0	0	0
0	0	0	0	0	0	0	0
0	0	0	0	0	0	0	0
0	0	0	1	0	0	0	12
0	0	0	0	0	0	0	0
0	0	0	0	0	0	0	0
0	0	0	0	0	0	0	0
0	0	0	0	0	0	0	0
0	0	0	0	0	0	0	0
0	0	0	0	0	0	2	0
0	0	0	0	0	0	0	0
0	0	0	0	0	0	0	0
0	0	0	0	0	0	2	0
0	0	0	0	0	0	0	0
0	0	0	0	0	0	2	0
0	0	0	0	0	1	0	0
0	0	0	0	0	0	0	0
0	0	0	0	1000	0	0	0
0	0	0	0	0	0	0	0
0	0	0	0	0	0	0	0
0	0	0	0	0	0	1	0
0	0	0	0	0	0	0	0
0	0	0	0	0	0	0	0
0	0	0	0	0	0	1	0
0	0	0	0	0	0	0	0
0	0	0	1	2	0	0	0
0	0	0	0	1	0	0	0
0	0	0	0	1	0	1	0
0	0	0	0	0	0	0	0
0	0	0	0	0	0	1	0
0	0	0	0	0	0	0	0
0	0	0	0	0	0	0	0
0	0	0	0	1	0	0	0
0	0	0	0	0	0	1	0
0	0	0	0	0	0	3	0
0	0	0	0	0	0	1	0
0	0	0	0	0	0	1	0
0	0	0	0	72	0	2	0
0	0	0	0	0	0	0	0
0	0	0	0	0	0	0	0
0	0	0	0	0	0	0	0
0	0	0	0	0	0	15	0
0	0	0	0	0	0	17	0
0	0	0	0	0	0	0	0
0	0	0	0	0	0	0	0
0	0	0	0	0	0	0	0
0	0	0	0	0	0	0	0
0	0	0	0	0	0	0	0
0	0	0	0	0	0	0	0
0	0	0	0	0	0	0	0
0	0	0	0	0	0	0	0
0	0	0	0	1	0	0	0
0	0	0	0	0	0	0	0
0	0	0	0	0	0	0	0
0	0	0	0	0	0	0	0

0	0	0	1	0	0	1	0
0	0	0	0	0	0	0	0
0	0	0	0	0	0	10	0
0	0	0	1	0	0	2	0
0	0	0	0	0	0	0	0
0	0	0	0	0	0	54	0
0	0	0	0	0	1	1	0
0	0	0	0	0	0	1	0
0	0	0	0	0	0	0	0
0	0	1	0	6	0	0	0
0	0	0	0	0	0	0	0
0	0	0	0	0	0	0	0
0	0	0	0	0	0	0	0
0	0	0	0	0	0	0	0
0	0	0	0	0	0	1	0
0	0	0	0	0	0	0	0
0	0	0	0	0	0	1	0
0	0	0	0	0	0	0	0
0	0	0	0	0	0	0	0
0	0	0	0	0	0	0	0
0	0	0	0	0	0	0	0
0	0	0	0	0	0	0	0
0	0	0	0	0	0	1	0
0	0	0	0	0	0	0	0
0	0	0	0	0	0	0	0
0	0	0	0	0	0	0	0
0	0	0	0	0	0	0	0
0	0	0	0	0	0	0	0
0	0	0	0	0	0	0	0
0	0	0	0	0	0	0	0
0	0	0	0	0	0	0	0
0	0	0	0	0	0	0	0
0	0	0	0	0	0	0	0
0	0	0	0	0	0	0	0
0	0	0	0	0	0	1	0
0	0	0	0	0	0	2	0
0	0	0	0	0	0	0	0
0	0	0	0	0	1	0	0
0	0	0	0	0	0	0	0
0	0	0	0	0	0	0	0
0	0	0	0	0	0	0	0
0	0	0	0	0	2	0	0
0	0	0	0	0	0	0	0
0	0	0	0	1	0	1000	0
0	0	0	0	0	0	1	0
0	0	0	0	0	0	0	0
0	0	0	0	0	0	0	0
0	0	0	0	0	0	0	0
0	0	0	0	0	0	0	0
0	0	0	0	0	0	0	0
0	0	0	0	0	0	0	0
0	0	0	0	0	0	0	0

asp_ustus_ra	asp_niger_ra	asp_sp_ra	asp_terreus_ra	asp_flavus_ra	peni_ra	clado_ra	alterna_ra
0	0	0	0	0	0	0	0
0	0	0	0	0	0	0	0
0	0	0	0	0	0	0	0
0	0	0	0	0	0	0	2
0	0	0	0	0	3	0	0
0	0	0	0	0	0	0	0
0	0	0	0	0	3	0	1
0	0	0	0	0	0	0	0
0	0	0	0	0	0	0	0
1	0	0	0	0	1	0	0
0	0	0	0	1	2	1	0
0	0	2	0	0	0	0	0
0	0	3	0	0	1	1	1
0	0	1	0	0	0	1	2
73	0	0	0	0	39	0	0
0	0	4	0	0	0	2	0
1	0	0	0	0	14	0	1
1000	0	0	0	0	0	0	1
0	0	0	0	0	0	0	0
1000	0	0	0	0	0	0	0
0	0	0	0	0	2	0	0
15	0	0	0	0	22	0	0
10	0	0	0	0	31	0	0
0	0	0	0	0	1	0	0
2	0	0	0	0	40	0	0
53	0	0	0	4	31	0	0
0	0	0	0	0	1	0	0
0	0	0	0	0	0	1	0
0	0	0	0	1	1	0	0
0	0	1000	0	0	1000	0	0
8	0	2	0	0	26	0	0
0	0	1	0	0	1	0	0
0	0	0	0	0	0	0	0
0	0	0	0	0	0	0	0
0	0	0	0	0	0	0	0
0	0	0	0	0	15	0	0
12	0	1	0	0	15	0	0
1	0	0	0	0	2	0	0
0	0	0	0	0	5	1	0
0	0	0	0	0	6	1	0
0	0	1	0	0	0	0	0
0	0	0	0	0	0	0	1
0	0	0	0	0	0	0	0
0	0	0	0	0	0	0	0
0	0	0	0	0	1	0	3
0	0	0	0	0	0	0	0
0	0	0	0	0	3	1	1
0	0	0	0	0	0	0	0
0	0	1	0	0	0	0	1
0	0	0	0	0	0	1	0
0	0	0	0	0	1	0	0
0	0	0	0	0	0	0	1
0	0	0	0	0	0	0	0
0	0	0	0	0	1000	0	0
0	0	0	0	0	0	0	0

0	0	0	0	0	13	0	0
0	0	0	0	0	20	0	0
0	0	0	0	0	0	0	0
0	0	0	0	0	0	0	0
0	0	0	0	0	1	0	0
0	0	0	0	0	0	0	0
0	4	4	0	0	3	0	1
0	0	0	0	0	0	0	0
0	0	0	0	0	0	0	0
0	0	0	0	0	0	0	0
0	1	3	0	0	0	0	0
0	0	0	0	0	0	1	0
0	0	0	0	0	0	0	0
0	1	1	0	0	1	0	2
0	0	0	0	0	1	0	0
0	0	0	0	0	4	0	2
0	0	0	0	1	0	0	0
0	0	0	0	0	1	0	0
0	0	0	0	0	2	0	3
0	0	0	0	0	0	0	0
0	0	0	0	0	1	0	0
0	0	0	0	0	2	0	0
0	0	0	0	0	0	0	0
0	0	0	0	0	0	0	0
0	0	0	0	0	0	0	0
0	0	0	0	0	1	0	2
0	0	0	0	0	0	0	0
0	0	0	0	0	2	0	0
0	0	0	0	0	83	0	0
0	0	0	0	0	2	0	0
0	0	0	0	0	0	0	0
0	0	0	0	0	0	0	0
0	0	0	0	0	1	0	5
0	0	0	0	0	0	0	0
0	0	0	0	0	6	0	0
0	0	0	0	0	27	1	0
0	0	0	0	0	0	1	0
0	0	0	0	0	0	0	0
0	0	2	0	0	6	1	0
2	0	0	0	0	22	0	1
0	0	0	0	0	2	5	3
0	0	3	0	0	10	0	0
0	1	0	0	0	0	1	2
0	0	6	0	0	3	0	0
2	0	0	0	0	1	0	0
0	0	0	0	0	0	0	0
0	0	0	0	0	22	0	4
0	0	0	0	0	59	1	1
0	0	0	0	0	2	0	1
0	0	0	0	0	1	0	0
0	0	0	0	0	1000	0	1
0	0	3	0	0	1	0	0
0	0	5	0	0	0	0	0
0	0	0	0	0	1	1	0
0	0	24	0	0	10	0	0

0	0	0	0	0	5	1	0
0	0	0	0	0	19	3	0
0	0	0	0	0	2	1	0
0	0	0	0	0	0	0	0
0	0	0	0	0	1	0	0
0	0	0	0	0	1	0	0
0	0	0	0	0	1	0	0
0	0	0	0	0	1	0	0
0	0	0	0	0	9	0	0
0	0	0	0	0	0	0	0
0	0	0	0	0	17	0	0
0	0	0	0	0	14	1	1
0	0	0	0	0	9	1	0
0	0	0	0	0	0	0	0
1	0	1	0	0	19	0	4
0	0	0	0	0	0	0	0
0	0	0	0	0	0	0	0
0	0	0	0	0	0	0	0
0	0	0	0	0	0	0	0
0	0	2	0	0	2	0	1
0	0	4	0	0	2	0	2
0	0	0	0	0	0	0	0
0	0	0	0	0	0	0	1
0	0	0	0	0	0	1	1
0	0	0	0	0	1	0	0
0	0	0	0	0	1	0	0
0	0	0	0	0	3	0	0
0	0	0	0	0	1	0	0
0	0	0	0	0	0	3	0
0	0	0	0	0	0	0	0
0	0	0	0	0	0	0	0
0	0	0	0	0	0	0	1
0	0	0	0	0	0	1	0
0	0	0	0	0	0	0	0
0	0	0	0	0	2	0	2
0	0	0	0	0	0	0	1
0	0	0	0	0	45	0	0
0	0	0	0	0	0	0	1
0	0	1	0	0	0	0	0
0	0	1	0	0	0	0	0
0	0	0	0	0	0	0	0
2	0	2	0	0	2	0	0
0	0	0	0	0	2	0	1
0	0	0	0	0	0	2	0
0	0	0	0	1	1	0	0
0	0	0	0	0	0	0	0
5	0	8	0	0	3	0	0
0	0	1	0	0	23	0	0
0	0	2	0	0	0	0	0
0	0	0	0	0	9	1	0
0	0	0	0	0	0	0	0
0	0	0	0	0	0	0	0
0	0	0	0	0	0	0	0
0	0	0	0	0	0	0	0

0	0	0	0	1	0	0	1
0	0	0	0	0	0	0	1
0	0	0	0	0	0	0	1
3	0	0	0	0	1	0	1
0	0	4	0	0	2	0	1
0	0	0	0	0	0	0	0
0	0	0	0	0	0	0	1
0	0	1	0	0	0	2	6
3	0	2	0	0	4	1	4
0	0	0	0	0	0	0	0
0	0	0	0	0	0	0	2
0	0	0	0	0	0	0	0
0	0	0	0	0	0	0	0
0	0	0	0	0	0	0	0

0	0	0	0	0	2	0	4
0	0	0	0	0	29	0	1
0	0	0	0	0	0	0	1
0	0	0	0	0	2	0	15
0	1	0	0	0	0	0	0
0	0	2	0	0	0	0	3
0	33	0	0	0	6	0	0
0	13	0	0	0	4	0	0
0	0	0	0	0	1	0	2

1	0	0	0	0	0	0	0
0	0	0	0	0	0	0	0
1	0	0	0	0	6	0	2
1	0	0	0	0	7	0	0
0	0	0	0	0	0	0	1
0	0	0	0	0	0	0	0
0	0	0	0	0	1	1	1
1	0	1	0	0	3	0	1
1	0	0	0	0	3	0	3
0	1	0	0	0	9	2	1
0	0	0	0	0	0	2	0
0	0	0	0	0	7	10	0
4	0	0	0	0	5	0	0
0	0	0	0	0	0	0	1
0	0	0	0	0	2	0	0
0	0	0	0	0	0	0	0
0	0	0	0	0	11	0	0
0	0	0	0	0	1	0	0
0	1	0	0	0	10	0	2
0	0	0	0	0	1	1	1
0	0	0	0	0	27	3	0
0	0	0	0	0	3	0	0
1	0	3	0	0	0	0	0
0	0	0	0	0	0	0	0
0	0	0	0	1	0	0	0
0	0	0	0	0	0	0	0
0	0	1	0	0	0	1	0
0	0	0	0	0	2	0	0
0	0	0	0	0	0	0	0

0	0	0	0	0	0	0	0
0	0	0	0	0	0	0	0
0	0	0	0	0	59	1	0
0	0	0	0	0	5	0	0
2	0	0	0	0	15	2	2
0	0	0	0	0	18	0	2
0	0	0	0	0	45	1	1
0	0	0	0	0	2	1	0
0	0	1	0	0	13	0	0
0	0	1	0	0	0	0	0
0	0	0	0	0	1	0	0
7	0	0	0	1	10	0	0
0	0	0	0	0	0	0	0
0	0	0	0	0	0	0	0
0	0	0	0	0	0	0	0
0	0	0	0	0	0	0	0
0	0	0	0	0	0	0	0
0	0	0	0	0	0	0	0
0	0	0	0	0	0	0	0
0	0	0	0	0	0	0	0
0	0	1	0	0	0	0	0
0	0	0	0	0	0	0	0
0	0	0	0	0	0	0	0
0	0	0	0	0	1	0	0
0	0	0	0	0	0	0	0

0	0	1	0	0	0	0	0
0	0	0	0	0	8	0	0
0	0	0	0	0	5	0	0
0	0	1	0	0	17	0	0
0	0	0	0	0	0	0	0
0	0	0	0	0	1	0	1
0	0	0	0	0	2	0	0
1	0	1	0	0	0	0	1
0	0	0	0	0	0	0	0
0	0	0	0	0	0	0	0
0	0	0	0	0	35	0	0
0	0	0	0	0	1	0	0
0	0	0	0	0	0	2	2
0	0	0	0	0	0	0	0
0	0	0	0	0	39	0	0
0	0	0	0	0	72	0	0
0	0	0	0	0	0	0	0
0	0	0	0	0	0	0	0
0	0	0	0	0	2	1	0
1	0	0	0	0	0	0	0
0	0	0	0	0	1	0	0
0	0	0	0	0	2	0	0
0	0	32	0	0	2	0	0
0	0	0	0	0	1	0	0

0	0	0	0	0	0	0	0
0	0	0	0	0	0	0	1
0	0	0	0	0	1	0	0
0	0	0	0	0	12	1	0
0	0	0	0	0	0	1	1

0	0	0	0	1	1	0	0
0	0	0	0	0	0	0	0
0	0	0	0	0	0	0	0
0	0	0	0	0	0	0	0
0	0	0	0	0	0	0	0
0	0	0	0	0	0	1	0
0	0	0	0	0	0	0	0
0	0	0	0	0	0	0	0
0	0	0	0	0	1	0	2
0	0	0	0	0	0	0	0
0	0	0	0	10	60	0	0
1	0	0	0	0	2	0	4
0	0	0	0	0	6	0	0
0	0	0	0	0	0	0	0
0	0	0	0	0	1	3	1
0	0	0	0	0	0	3	1
1	2	0	0	0	0	0	5
0	0	1	0	0	1	1	1
0	0	0	0	0	0	2	4
0	0	0	0	0	1	0	0
0	0	0	0	0	0	0	0
0	0	0	0	0	0	0	0
0	0	0	0	0	0	0	0
0	0	0	0	0	0	0	0
0	0	0	0	0	0	0	1
0	0	0	0	0	0	0	0
0	0	0	0	0	0	0	0
0	0	0	0	0	0	0	0
0	0	0	0	0	0	0	0
0	0	0	0	0	0	0	0
0	0	0	0	0	0	0	0
0	0	0	0	0	0	0	0

0	0	0	0	0	0	0	0
0	0	0	0	0	4	2	1
0	0	0	0	0	3	0	0
0	0	0	0	0	0	0	0
0	0	0	0	0	2	0	0
0	0	0	0	0	3	0	0
0	0	0	0	0	0	0	0
0	0	1	0	0	0	0	1
0	0	0	0	0	0	0	0
0	0	0	0	0	2	0	1
0	0	0	0	0	8	0	1
0	0	0	0	0	0	0	0
0	0	0	0	0	1	1	0
0	0	2	0	0	3	0	0
0	0	0	0	1	2	0	0
0	0	0	0	0	1	0	0
0	0	0	0	0	2	0	3
0	0	0	0	0	0	0	0
0	0	0	0	0	3	0	0
0	0	0	0	0	0	0	0

0	0	17	0	0	34	0	0
0	0	9	0	0	6	0	0
0	0	8	0	0	6	0	0
1	0	0	0	0	25	0	0
19	0	17	0	0	7	0	1
0	0	0	0	0	0	0	0
1	0	0	0	0	0	0	0
0	0	0	0	0	0	0	0
0	0	1	0	0	2	2	0
3	0	3	0	0	8	2	2
4	2	0	0	0	29	0	0
0	2	0	0	0	3	1	0
7	0	0	0	0	25	0	1
0	1	1	0	0	16	1	0
0	1	4	0	2	9	1	1
3	0	1	0	2	1	0	0
0	0	0	0	0	7	0	1
0	0	0	0	0	1	0	0
0	0	0	0	0	1	1	0
0	0	0	0	0	0	0	1
0	0	0	0	0	0	0	0
0	0	0	0	0	5	0	0
0	0	0	0	0	0	0	0
0	0	0	0	0	0	0	0
0	0	0	0	0	9	1	1
0	0	0	0	0	0	0	0
0	0	0	0	0	0	0	0
0	0	0	0	0	0	1	0
0	0	0	0	0	0	0	0
0	0	0	0	0	1	0	0
0	0	0	0	0	0	0	0
0	0	0	0	0	2	1	0
0	0	0	0	1	1	0	0
0	0	0	0	0	1	0	1
0	0	0	0	0	0	0	1
0	0	0	0	0	1	0	0
0	0	0	0	0	0	0	0
0	0	0	0	0	8	3	18
0	0	4	0	0	1	0	10
0	0	0	0	0	1	1	0
0	0	0	0	0	0	0	2
0	0	0	0	0	2	2	4
1	1	0	0	0	8	3	1
0	0	0	0	0	0	0	0
0	0	0	0	0	4	3	0
0	0	1	0	0	0	0	0
0	0	0	0	0	3	1	0
0	0	0	0	0	1	0	0
0	0	2	0	0	8	0	1
0	0	0	0	0	0	0	0
0	0	0	0	0	83	0	1
0	0	0	0	0	3	0	0
0	0	0	0	0	0	0	0
0	0	1	0	0	1000	3	3
0	0	0	0	0	3	0	0

0	0	0	0	0	4	4	0
0	1	0	0	0	7	6	0
0	0	0	0	0	44	0	0
0	0	0	0	0	3	0	0
0	0	0	0	0	0	0	0
0	0	0	0	0	15	0	0
0	0	0	0	0	0	0	0
0	0	0	0	0	0	1	0
0	0	0	0	0	0	0	0
0	0	0	0	0	1	0	0
0	0	0	0	0	0	0	5
0	0	0	0	0	0	0	0
15	0	0	0	0	0	0	0
0	0	0	0	0	2	0	0
0	0	0	0	0	2	0	1
1	0	0	0	0	22	1	1
0	0	0	0	0	0	0	0
0	0	15	0	0	0	0	0
0	0	0	0	0	0	0	0
0	0	0	0	0	7	0	0
0	0	0	0	0	7	0	1
0	0	0	0	0	0	2	1
0	0	0	0	0	0	0	1
0	0	2	0	0	8	2	1
0	0	0	0	0	26	0	1
0	0	0	0	0	1000	0	0
0	0	0	0	0	1000	0	0
0	0	0	0	0	0	0	0
0	0	0	0	0	0	1	0
0	0	0	0	0	6	0	0
0	0	0	0	0	1	0	1
0	0	0	0	0	4	0	0
0	0	0	0	0	0	0	1
3	0	0	0	0	0	0	0
0	0	0	0	0	0	0	0
2	0	0	0	0	0	0	0
10	0	0	0	0	1	0	0
0	0	0	0	0	1	0	1
0	0	0	0	0	0	0	0
0	0	0	0	0	0	0	0
0	0	0	0	0	0	1	4
0	0	3	0	0	7	1	0
0	9	5	0	0	1000	0	1
0	5	0	0	0	1000	0	1
0	0	0	0	0	9	0	1
0	0	0	0	0	0	0	0
0	0	0	0	0	0	0	0
0	0	0	0	0	0	0	0
0	0	0	0	0	1	0	0
0	0	0	0	0	0	0	0
0	0	1	0	0	2	1	0
0	0	0	0	0	0	0	0

ulocla_ra	chaeto_ra	scopula_ra	acremo_ra	fusa_ra	rhizo_ra	mucor_ra	geotri_ra	trichoderm_ra
0	0	0	0	0	0	0	0	0
0	0	0	0	0	0	0	0	0
0	0	0	0	0	0	0	0	0
0	0	0	0	0	0	0	0	0
0	0	0	0	0	0	0	0	0
0	0	0	0	0	0	0	0	0
0	0	0	0	0	0	0	0	0
0	0	0	0	0	0	0	0	0
0	0	0	0	0	0	0	0	0
0	0	0	0	0	0	0	0	0
0	0	1	0	0	0	0	0	0
1	0	0	0	0	0	0	0	0
0	0	1	1	0	0	0	0	0
2	0	0	0	0	0	0	0	0
0	0	0	0	0	0	0	0	0
0	0	0	0	0	0	0	0	0
0	0	0	0	0	0	0	0	0
0	0	0	0	0	0	0	0	0
0	0	0	0	0	0	0	0	0
0	0	0	0	0	0	0	0	0
0	0	0	0	0	0	0	0	0
0	2	0	0	0	0	0	0	0
0	0	0	0	0	0	0	0	0
0	0	0	0	0	0	0	0	0
0	2	0	0	0	0	1	0	0
0	0	0	0	0	0	0	0	0
0	0	0	0	0	0	1	0	0
0	0	0	0	0	0	0	0	0
0	0	0	0	0	0	0	0	0
0	0	0	0	0	0	0	0	0
0	24	0	0	0	0	0	0	0
0	0	0	0	0	0	0	0	0
0	0	0	0	0	0	0	0	0
0	0	0	0	0	0	0	0	0
0	0	0	0	0	0	0	0	0
0	0	0	0	0	0	0	0	0
0	0	0	0	0	0	0	0	0
0	1	0	0	0	0	0	0	4
3	0	0	0	0	0	0	0	0
0	0	0	0	0	0	0	0	0
0	0	0	0	0	0	0	0	0
0	0	0	1	0	0	0	0	0
0	0	0	0	0	0	0	0	0
0	0	0	0	0	0	0	0	0
0	0	0	0	0	0	0	0	0
0	0	0	0	0	0	0	0	0
0	0	0	0	0	0	0	0	0
0	0	0	0	0	0	0	0	0
0	0	0	0	0	0	0	0	0
0	0	0	0	0	0	0	0	0
0	0	0	0	0	0	0	0	0
0	0	0	0	0	0	0	0	0
0	0	0	0	0	0	0	0	0
0	0	0	0	0	0	0	0	0
0	0	0	0	0	0	0	0	0

0	1	0	0	0	0	0	0	0
0	0	0	0	0	0	0	0	0
0	0	0	0	0	0	0	0	0
0	0	0	0	0	0	0	0	0
0	0	0	0	0	0	0	0	0
0	0	0	0	0	0	0	0	0
0	0	0	0	0	0	0	0	0
0	0	0	0	0	0	0	0	0
0	2	0	0	0	0	0	0	0
0	1	0	0	0	0	0	0	0
0	0	0	0	0	0	0	0	0
0	0	0	0	0	0	0	0	0
0	0	0	0	0	0	0	0	0
0	0	0	0	0	0	0	0	0
0	0	0	0	0	0	0	0	0
0	0	0	0	0	0	0	0	0
0	0	0	0	0	0	0	0	0
0	0	0	0	0	0	0	0	0
0	0	0	0	0	0	0	0	0
0	0	0	0	0	0	0	0	0
0	0	0	0	0	0	0	0	0
0	0	0	0	0	0	0	0	0
0	1	0	0	0	0	0	0	0
0	0	0	0	0	0	0	0	0
0	0	0	0	0	0	0	0	0
0	0	0	0	0	0	0	0	0
0	0	0	0	0	0	0	0	0
0	0	0	0	0	0	0	0	0
0	0	0	0	0	0	0	0	0
0	0	0	0	0	0	0	0	0
0	0	0	0	0	0	0	0	0
0	0	0	1	0	0	0	0	0
0	0	0	0	0	0	0	0	0
0	0	0	0	0	0	0	0	0
0	0	0	0	0	0	0	0	0
0	0	0	0	0	0	0	0	0

0	0	0	0	0	0	0	0	0
1	0	0	0	0	0	0	0	0
0	0	0	0	0	0	0	0	0
0	0	0	0	0	0	0	0	0
0	0	0	0	0	0	0	0	0
0	0	0	0	0	0	0	0	0
0	0	0	0	0	0	0	0	0
0	0	0	0	0	0	0	0	0
0	0	0	0	0	0	0	0	0
0	0	9	0	0	0	0	0	0
0	0	1	0	0	0	0	0	0
0	1	0	0	0	0	0	0	0
0	0	0	0	0	0	0	0	0
0	0	0	0	0	0	0	0	0
0	0	0	0	0	0	0	0	0
0	1	0	0	0	0	0	0	0
0	0	0	0	0	0	0	0	0
0	0	0	0	0	0	0	0	0
0	0	0	0	0	0	0	0	0

0	0	0	3	0	0	0	0	0
0	0	0	0	0	0	0	0	0
0	0	0	0	0	0	0	0	0
0	0	0	0	0	0	0	0	0
0	0	0	0	0	0	0	0	0
0	0	0	0	0	0	0	0	0
0	0	0	0	0	0	0	0	0
0	0	0	0	0	0	0	0	0
0	0	0	0	0	0	0	0	0
0	0	0	0	0	1000	0	0	0
0	0	0	0	0	0	0	0	0
0	0	0	0	0	0	0	0	0
0	0	0	0	0	0	1	0	0
0	0	0	0	0	0	0	0	0
0	0	0	0	1	0	0	0	1
0	0	0	0	0	0	0	0	0
0	0	0	0	0	0	0	0	0
0	0	0	0	0	0	0	0	0
0	0	0	0	0	0	0	0	0
0	0	0	0	0	0	0	0	0
0	0	0	0	0	0	0	0	0
0	0	0	0	0	0	0	0	0
0	0	0	0	0	0	0	0	0
0	0	0	0	0	0	0	0	0
0	0	0	0	0	0	0	0	0
0	0	0	0	0	0	0	0	0
0	0	0	0	0	0	0	0	0
0	0	0	0	0	0	0	0	0
1	0	0	0	0	0	0	0	0
0	0	0	0	0	0	0	0	0
0	0	0	0	0	0	0	0	0
0	0	0	0	0	0	0	0	0
0	0	0	0	0	0	0	0	0
0	0	0	0	0	0	0	0	0
0	0	0	0	0	0	0	0	0

0	0	0	0	0	0	0	0	1
0	0	0	0	0	0	0	0	0
0	0	0	0	0	0	0	0	0
0	0	0	0	0	0	0	0	0
0	0	0	0	0	0	0	0	0
0	0	0	0	0	0	0	0	0
0	0	0	0	0	0	0	0	0
0	0	0	0	0	0	0	0	0
0	0	0	0	0	0	0	0	1
0	0	1	0	0	0	0	0	0
0	0	0	0	0	0	0	0	0
0	0	0	0	0	0	0	0	0
1	0	0	0	0	1	1	0	0
0	0	0	0	0	0	0	0	0

0	0	0	0	0	0	0	0	0
0	0	0	0	0	0	0	0	0
0	0	0	0	0	0	0	0	0
0	1	0	0	0	0	0	0	0
0	0	0	0	0	0	0	0	0

0	0	0	0	0	0	0	0	0
0	0	0	0	0	0	0	0	0
0	0	0	0	0	0	0	0	0
0	0	0	0	0	0	0	0	0
0	0	0	1	0	0	0	0	0
0	0	0	0	0	0	0	0	0
0	0	0	0	0	0	0	0	0
0	0	0	0	0	0	0	0	0
0	0	0	0	0	0	0	0	0
0	0	0	0	0	0	0	0	0
0	1	0	0	0	0	0	0	0
0	0	0	0	0	0	0	0	0
0	0	0	0	0	0	0	0	0
0	0	0	0	3	0	0	0	0
0	0	0	0	0	0	0	0	0
1	0	0	0	0	0	0	0	0
0	0	0	0	0	0	0	0	0
0	0	0	0	0	0	0	0	0
0	0	0	0	0	1	0	0	0
0	0	0	0	0	0	0	0	0
0	0	0	0	0	0	0	0	0
0	0	0	0	0	0	0	0	0
0	0	0	0	0	0	0	0	0
0	0	0	0	0	0	0	0	0
0	0	0	0	0	0	0	0	0
0	0	0	0	0	0	0	0	0
0	0	0	0	0	0	0	0	0
0	0	0	0	0	0	0	0	0
0	0	0	0	0	0	0	0	0
0	0	0	0	0	0	0	0	0
0	0	0	0	0	0	0	0	0
0	0	0	0	0	0	0	0	0
0	0	0	0	0	0	0	0	0
0	0	0	1	0	0	0	0	0
0	0	0	2	0	0	0	0	0
0	0	0	0	0	0	0	0	0
0	0	0	0	0	0	0	0	0
0	0	0	0	0	0	0	0	0
0	0	0	0	0	0	0	0	0
1	0	0	0	0	0	0	0	0
0	0	0	0	0	0	8	0	0
0	0	0	0	0	0	5	0	0
0	0	0	0	0	1	0	0	0
0	0	0	0	0	0	0	0	0
0	0	0	0	0	0	0	0	0
0	0	0	0	0	0	0	0	0
0	0	0	0	0	0	0	0	0
0	0	0	0	0	0	0	0	0
0	0	0	0	0	0	1	0	0
0	0	0	0	0	0	0	0	0
0	0	0	0	0	0	0	0	0
0	0	2	0	0	0	0	0	0

0	0	0	0	0	0	0	0	0
3	0	0	0	0	0	0	0	0
0	0	0	0	0	0	0	0	0
0	0	0	0	0	0	0	0	0
0	0	0	0	0	0	0	0	0
0	0	0	0	0	0	0	0	0
0	0	0	0	0	0	0	0	0
0	0	0	0	0	0	0	0	0
0	1	0	0	0	0	0	0	0
0	0	0	0	0	0	0	0	0
0	0	0	0	0	0	0	0	0
0	0	0	0	0	0	0	0	0
0	0	0	0	0	0	0	0	0
0	0	0	0	0	0	0	0	0
0	0	0	0	0	0	5	0	0
0	0	0	0	0	0	0	0	0
0	0	0	0	0	0	0	0	0
0	0	0	0	0	0	0	0	0
0	0	0	0	0	0	0	0	0
0	0	1	0	0	0	0	0	0
0	0	0	0	0	0	0	0	0
0	0	0	0	0	0	0	0	0
0	0	0	0	0	0	0	0	0
0	0	0	0	0	0	0	0	0
0	0	0	0	0	0	0	0	0
0	0	0	0	0	0	0	0	0
0	0	0	0	0	0	0	0	0
0	0	0	0	0	0	0	0	0
1	0	0	0	0	0	1	0	0
0	0	0	0	0	0	0	0	0
0	0	0	0	0	0	0	0	0
0	0	0	0	0	0	0	0	0
0	0	0	0	0	0	0	0	0
0	0	0	0	0	0	0	0	0
0	0	0	0	1	0	0	0	0
0	0	0	0	0	0	0	0	0
0	2	0	0	0	0	0	0	0
0	0	0	0	0	0	0	0	0
0	0	0	0	0	0	0	0	0
0	0	0	0	0	0	0	0	0
0	0	1	0	0	0	0	0	0
0	0	0	0	0	0	0	1	0
0	0	0	0	0	0	0	0	0
0	0	0	0	0	0	0	0	0
0	0	0	0	0	0	0	0	0
0	0	0	0	0	0	0	0	0
0	0	0	0	0	0	0	0	0
0	0	0	0	0	0	0	0	0
0	0	0	0	0	0	0	0	0
0	0	0	0	0	0	0	0	0
0	0	0	0	0	0	0	0	0
0	0	0	0	0	0	0	0	0
0	0	0	0	0	0	0	0	0
0	0	0	0	0	0	0	0	0

0	0	0	0	0	0	0	0	0
0	0	0	0	0	0	0	0	0
0	0	0	0	0	0	0	0	0
0	0	0	0	0	0	0	0	0
0	0	0	0	0	0	0	0	0
0	0	0	0	0	0	0	0	0
0	0	0	0	0	0	0	0	0
0	0	0	0	0	0	0	0	0
0	0	0	0	0	0	0	0	0
0	0	0	0	0	0	0	0	0
0	0	0	0	0	1	0	0	0
0	0	0	0	0	0	0	0	0
0	0	0	0	0	0	0	0	0
0	0	0	0	0	0	0	0	0
0	0	0	0	0	0	0	0	0
0	0	0	0	0	0	0	0	0
0	0	0	2	0	0	0	0	0
0	0	0	0	0	0	0	0	2
0	0	0	0	0	0	0	0	0
0	0	0	0	0	0	0	0	0
0	0	0	0	0	0	0	0	0
0	0	0	0	0	0	0	0	0
0	0	0	0	0	0	0	0	0
0	0	0	0	0	0	0	0	0
0	0	0	0	0	0	0	0	0
0	0	0	0	0	0	0	0	0
0	0	0	0	0	0	0	0	0
0	0	0	0	0	0	0	0	0
0	0	0	0	0	0	0	0	0
0	0	0	0	0	0	0	0	0
0	0	0	0	0	0	0	0	0
0	0	0	0	0	0	0	0	0

0	0	0	0	0	0	0	0	0
0	0	0	0	0	1	1	0	0
0	0	0	0	0	0	0	0	0
0	0	0	0	0	0	0	0	0
0	0	0	0	0	0	0	0	0
0	0	0	0	0	0	0	0	0
0	0	0	0	0	0	0	0	0
0	0	0	0	0	0	0	0	0
0	0	0	0	0	0	0	0	0
0	0	0	0	0	0	0	0	0
0	0	0	0	0	0	0	0	0
0	0	0	0	0	0	0	0	0
0	0	0	0	0	0	0	0	0
0	0	0	0	0	0	0	0	1
0	0	0	0	0	0	0	0	0
0	0	0	0	0	0	0	0	0
0	0	0	0	0	0	0	0	0
0	0	0	0	0	0	0	0	0
0	0	0	0	0	0	0	0	0
0	0	0	0	0	0	0	0	0

0	0	0	0	0	0	0	0	0
0	62	0	0	0	0	0	0	0
0	37	0	0	0	0	0	0	0
0	16	0	0	0	0	0	0	0
0	0	0	0	0	0	0	1	0
0	0	0	0	0	0	0	0	0
0	0	0	0	0	0	0	0	0
0	0	0	0	0	0	0	0	0
0	0	0	0	0	0	0	0	0
0	0	0	0	0	0	0	0	0
1	0	0	0	0	0	0	0	0
1	0	0	0	0	0	0	0	0
0	0	0	0	0	0	0	0	0
0	0	0	0	0	0	0	0	0
0	0	0	0	0	0	0	0	0
0	0	0	0	0	0	0	0	0
0	0	0	0	0	0	0	0	0
0	0	0	0	0	0	0	0	0
0	0	0	0	0	0	0	0	0
0	0	0	0	0	0	0	0	0
0	0	0	0	0	0	0	0	0
0	0	0	0	0	0	0	0	0
0	0	0	0	0	0	0	0	0
0	0	0	0	0	0	0	0	0
0	0	0	0	0	0	0	0	0
0	0	0	0	0	0	0	0	0
0	0	0	0	0	0	0	0	0
0	0	0	0	0	0	0	0	0

0	0	0	0	0	0	0	0	0
0	0	0	0	0	0	0	0	0
0	0	0	0	0	0	0	0	0
0	0	0	0	0	0	0	0	0
0	0	0	0	0	0	0	0	0
0	0	0	1	0	0	0	0	0
1	0	0	0	0	0	0	0	1
0	0	0	0	0	0	0	0	0
0	0	0	0	0	0	0	0	0
0	0	0	0	0	0	0	0	0
0	0	0	0	0	0	0	1	0
0	0	0	0	0	0	0	0	0
0	0	0	0	0	0	4	0	0
0	0	0	0	0	0	0	0	1
0	0	0	0	0	0	0	0	0
0	0	0	0	0	0	1000	0	0
0	0	0	0	0	0	0	0	0
0	0	0	0	0	0	0	0	0
0	0	0	0	0	0	0	0	0
0	0	0	0	0	0	0	0	0
0	0	0	0	0	0	0	2	0
0	0	0	0	0	0	1000	0	0
0	0	0	0	0	0	0	0	0
0	0	0	0	0	0	0	0	0
0	0	0	0	0	0	0	0	0
0	0	0	0	0	0	0	0	0
0	0	0	0	0	0	0	4	0

0	0	0	0	0	0	0	0	0
0	0	0	0	0	0	0	0	0
1	0	0	0	2	0	0	0	0
0	0	0	0	0	0	0	0	0
0	0	0	0	0	0	0	0	0
2	0	0	0	0	0	0	0	0
0	0	1	0	0	0	0	0	0
0	0	0	0	0	0	0	0	0
0	0	0	0	0	0	0	0	0
0	0	0	0	0	0	0	0	0
0	0	0	0	0	0	0	0	0
0	0	0	0	0	0	0	0	0
0	0	0	0	0	0	0	0	0
0	0	0	0	0	0	0	0	0
0	0	0	0	0	0	0	0	0
0	0	0	0	0	0	0	0	0
0	3	0	0	0	0	0	0	0
0	0	0	0	0	0	0	0	0
0	0	0	0	0	0	0	0	0
0	0	0	0	0	0	0	0	0
0	0	0	0	0	0	0	0	0
0	0	0	0	0	0	0	0	0
0	0	0	0	0	0	0	0	0
0	0	0	0	0	0	0	0	0
1	0	0	0	0	0	0	0	0
0	0	0	0	0	0	0	0	0
0	0	0	0	0	0	0	0	0
0	0	0	0	0	0	0	0	0
0	0	0	0	0	0	0	0	0
0	0	0	0	0	0	0	0	0
0	0	0	0	0	0	0	0	0
0	0	0	0	0	0	0	0	0
0	0	1	0	0	0	0	0	0
0	0	0	0	0	0	0	0	0
0	0	0	0	0	0	0	0	0
0	0	0	0	1	0	0	0	0
1	0	0	0	0	0	0	0	0
0	0	0	0	0	0	0	0	0
0	0	0	0	0	0	0	0	0
0	0	0	0	0	0	1	0	0
0	0	0	0	0	0	0	0	0
0	0	0	0	0	0	0	0	0
0	0	0	0	0	1	0	0	0
0	0	0	0	0	0	0	0	0
0	0	0	0	0	0	0	0	0
0	0	0	0	0	0	0	0	0
0	0	0	0	0	0	0	0	0
0	0	1	0	0	0	0	0	0
0	0	0	0	0	0	0	0	0
0	0	1	0	0	0	0	0	0
0	0	0	0	0	0	0	0	0
0	0	0	0	0	1000	0	0	0

paecilo_ra	autres_ra	csd_ra	levure_ra	asp_fumi_sol	sct_nid_sol	asp_nid_sol	asp_ustus_sol
0	0	0	0	0	0	0	0
0	1	0	0	0	3	0	0
0	1	2	3	0	0	0	0
0	0	1	0	0	0	0	0
0	0	0	2	0	0	0	0
0	0	0	0	0	0	0	0
0	0	0	0	0	0	0	0
0	0	0	0	0	0	0	0
0	0	0	0	0	0	0	0
0	0	0	0	0	0	0	0
0	0	10	0	0	0	0	0
0	0	15	0	0	0	0	0
0	0	14	4	0	0	0	0
0	0	8	0	0	0	0	0
0	0	0	0	0	0	0	1
0	0	10	3	0	0	0	0
0	1	6	4	0	0	0	0
0	0	0	0	0	0	0	0
0	0	0	1	0	0	0	0
0	0	0	0	0	0	0	0
0	0	1	0	0	0	0	0
0	0	2	0	0	0	0	0
0	0	0	0	0	0	0	0
0	0	2	1	0	0	0	0
0	0	0	0	0	0	0	0
0	0	1	0	0	1	0	0
0	0	1	0	0	0	0	0
0	0	0	0	0	0	0	0
0	0	0	0	0	0	0	0
0	0	1000	0	0	1000	0	0
0	0	0	0	0	0	0	0
0	0	7	1	0	1	0	4
0	0	5	3	0	0	0	0
0	0	2	0	0	0	0	0
0	0	0	0	0	0	0	0
0	0	1	0	0	0	0	0
0	0	9	14	0	0	0	0
0	0	0	0	0	1	0	0
0	0	1	1	0	0	0	0
4	0	1	6	0	2	0	0
5	0	2	1	0	1	0	0
0	0	0	0	0	0	0	0
0	0	2	1000	0	0	0	0
0	0	1	0	0	0	0	0
0	0	4	2	0	0	0	0
0	0	4	1	0	1	0	0
0	1	5	1	0	0	0	0
0	0	0	0	0	0	0	0
0	0	2	2	0	54	0	0
0	0	2	3	0	0	0	0
0	2	1	1	0	0	0	0
0	0	4	0	0	0	0	0
0	1	5	0	0	0	0	0
0	0	0	0	0	2	0	1
0	0	3	0	0	0	0	0

0	0	2	0	0	0	0	0
0	0	2	1	0	0	0	0
0	0	0	0	0	0	0	0
0	0	0	0	0	0	0	0
0	0	0	0	0	0	0	0
0	0	4	0	0	2	0	0
0	1	24	5	0	0	0	0
0	0	0	0	0	0	0	0
0	1	5	0	0	0	0	0
0	0	0	0	0	0	0	0
0	0	0	0	0	0	0	0
1	0	4	1	0	0	0	0
0	0	0	0	0	0	0	0
0	0	34	0	0	0	0	0
0	0	0	0	0	0	0	0
0	0	0	0	0	0	0	0
0	0	0	1	0	0	0	0
0	0	0	0	0	0	0	0
0	0	0	0	0	0	0	0
0	0	3	2	0	0	0	0
0	0	0	2	0	0	0	0
0	0	0	1	0	0	0	0
0	0	0	0	0	0	0	0
0	0	0	0	0	0	0	0
0	0	0	0	0	0	0	0
0	0	2	0	0	0	0	0
0	0	0	0	0	0	0	0
0	0	10	2	0	0	0	0
0	0	1	0	0	0	0	0
0	0	0	0	0	0	0	0
0	0	2	19	0	0	0	0
0	0	0	3	0	0	0	0
0	0	4	7	0	0	0	0
0	0	5	2	0	0	0	0
0	0	6	4	0	0	0	0
0	2	17	3	0	0	0	0
0	0	3	0	0	0	0	0
0	0	0	0	0	0	0	0
0	0	1	0	0	0	0	0
0	0	3	1	0	0	0	0
0	0	18	0	0	0	0	0
0	0	1	0	0	0	0	0
0	0	4	2	0	0	0	0
0	0	1	6	0	0	0	0
0	5	0	1	0	0	0	0
0	0	0	0	0	0	0	0
0	0	0	0	0	0	0	0
0	0	1	0	0	0	0	0
0	0	4	0	0	0	0	0
0	0	1	0	0	0	0	0
0	1	1	0	0	0	0	0
0	0	4	0	0	7	0	0
0	0	14	0	0	0	0	0
1	0	21	0	0	0	0	0
0	0	1	3	0	0	0	0

0	0	12	7	0	0	0	0
0	0	0	1	0	0	0	0
0	0	1	0	0	0	0	0
0	0	1	0	0	0	0	0
3	0	1	1	0	0	0	0
0	0	2	2	0	0	0	0
0	0	0	0	0	0	0	0
0	1	0	3	0	0	0	0
0	0	0	2	0	0	0	0
0	0	4	9	0	0	0	0
0	0	0	1	0	0	0	0
0	2	1	2	0	0	0	0
0	0	3	0	0	0	0	0
0	0	0	0	0	1	0	0
0	0	9	0	0	0	0	0
0	0	0	0	0	0	0	0
1000	0	0	0	0	0	0	0
96	0	0	0	0	0	0	0
10	0	0	1	0	0	0	0
0	0	0	4	0	0	0	0
0	0	3	1	0	0	0	0
0	0	2	0	0	0	0	0
0	0	1	6	0	0	0	0
0	0	0	0	0	0	0	0
0	0	1	1	0	0	0	0
0	0	6	7	0	0	0	0
0	0	2	5	0	0	0	0
0	0	2	1	0	0	0	0
0	0	5	6	0	0	0	0
0	0	0	0	0	0	0	0
0	0	0	0	0	0	0	0
0	0	3	0	0	0	0	0
0	0	0	0	0	0	0	0
0	0	0	0	0	0	0	0
0	0	5	7	0	0	0	0
0	0	0	0	0	0	0	0
0	0	0	3	0	0	0	0
0	1	2	0	0	0	0	0
0	0	0	1	0	0	0	0
0	0	2	4	0	0	0	0
0	0	0	1	0	0	0	0
0	0	3	0	0	0	0	0
0	0	1	0	0	0	0	0
0	0	5	0	0	0	0	0
0	0	0	0	0	0	0	0
0	0	0	0	0	0	0	0
0	0	0	0	0	0	0	1
0	0	0	0	0	0	0	0
0	0	0	0	0	0	0	0
0	0	0	0	0	0	0	0
0	0	0	7	0	1	0	0
0	0	0	1	0	0	0	0
0	0	0	5	0	0	0	0
0	1	1	1	0	0	0	0

0	0	17	1	0	0	0	0
0	0	1	0	0	0	0	0
0	0	0	1	0	0	0	0
0	0	1	1	0	0	0	0
0	0	4	5	0	0	0	0
0	0	0	0	0	0	0	0
0	0	13	0	0	0	0	0
0	0	15	2	0	0	0	0
0	0	26	2	0	0	0	0
0	0	0	0	0	1	0	0
0	0	6	2	0	0	0	0
0	0	0	0	0	0	0	0
0	0	0	0	0	0	0	0
0	0	0	1	0	0	0	0
0	0	5	1	0	0	0	0
0	0	8	6	0	0	0	0
0	0	3	0	0	0	0	0
0	0	12	4	0	0	0	0
0	0	0	0	0	1	0	0
0	0	8	5	0	0	0	0
0	0	3	2	0	7	0	0
1	2	5	0	0	0	0	0
0	0	4	1	0	0	0	0
0	0	1	0	0	0	0	0
0	0	0	4	0	0	0	0
0	0	2	0	0	0	0	0
0	0	1	0	0	0	0	0
0	0	10	0	0	0	0	0
0	1	0	0	0	0	0	0
0	0	5	6	0	0	0	0
0	1	4	65	0	0	0	0
0	0	1	38	0	0	0	0
0	0	14	48	0	0	0	0
0	0	0	25	0	0	0	0
0	0	1	1000	0	1	0	0
0	0	0	108	0	0	0	0
0	0	0	54	0	0	0	0
0	0	0	1000	0	0	0	0
0	0	0	42	0	0	0	0
0	0	0	37	0	0	0	0
0	0	0	0	0	0	0	0
0	0	3	5	0	1	0	0
0	0	0	1	0	0	0	0
0	0	20	3	0	0	0	0
0	0	25	8	0	0	0	0
0	0	24	2	0	0	0	0
0	0	1	0	0	0	0	0
0	0	0	0	0	0	0	0
0	0	0	1	0	0	0	0
0	0	2	4	0	0	0	0
0	0	1	0	1	0	0	0
0	0	0	0	0	0	0	0

0	0	0	0	0	2	0	2
0	0	5	0	0	0	0	0
0	0	2	1	0	0	0	0
0	0	1	2	0	0	0	0
0	0	5	4	0	0	0	0
0	0	0	0	0	4	0	0
0	0	4	5	0	0	0	0
0	0	0	0	0	0	0	0
0	0	0	2	0	0	0	0
0	1	1	0	0	0	0	0
0	0	0	0	0	0	0	0
0	0	0	2	0	0	0	0
0	0	0	0	0	0	0	0
0	0	0	0	0	0	0	0
0	0	0	0	0	0	0	0
0	0	1	0	0	0	0	0
0	0	0	0	0	0	0	0
0	0	0	0	4	0	0	0
0	0	0	0	0	0	0	0
0	0	0	1	0	0	0	0
0	0	1	1	0	12	0	0
0	0	0	0	0	0	0	0
0	0	0	0	0	0	0	0
0	0	0	0	0	0	0	0
0	0	0	0	0	0	0	0
0	0	0	0	0	0	0	0
0	0	0	1	0	0	0	0
0	0	0	0	0	0	0	0
0	0	3	11	0	0	0	0
0	0	0	2	0	0	0	0
0	0	2	5	0	0	0	0
0	2	2	3	0	0	0	0
0	0	3	0	0	0	0	0
0	0	1	0	0	0	0	0
0	0	1	0	0	0	0	0
0	0	0	2	0	0	0	0
0	2	3	1	0	0	0	0
0	0	4	5	0	0	0	0
0	0	0	8	0	0	0	0
0	0	0	0	0	0	0	0
0	0	0	0	0	0	0	0
0	0	0	0	0	0	0	1
0	0	0	0	0	0	0	0
0	0	1	0	0	0	0	0
0	0	2	4	0	0	0	0
1	0	0	0	0	0	0	0
0	3	0	0	0	0	0	0
0	0	0	0	0	0	0	0
0	0	2	0	0	0	0	0
0	0	0	0	0	0	0	0
0	0	1	1	0	0	0	0
0	0	3	4	0	0	0	0
0	0	2	3	0	0	0	0
0	0	2	5	0	0	0	0

0	0	0	0	0	0	0	0
0	0	0	0	0	0	0	0
0	0	0	0	0	0	0	0
0	0	0	0	1	0	0	0
0	0	1	0	0	0	0	0
0	0	0	0	0	0	0	0
0	0	0	0	0	0	0	0
0	0	0	0	0	0	0	0
0	1	1	0	0	0	0	0
0	0	0	1	0	0	0	0
0	0	0	7	0	0	0	0
0	0	15	0	0	0	0	0
0	0	2	0	0	0	0	0
0	0	1	0	0	0	0	0
0	0	9	3	0	0	0	0
0	0	2	0	0	0	0	0
0	0	6	0	0	0	0	0
0	0	14	2	0	0	0	0
0	0	14	5	0	0	0	0
0	0	2	2	0	0	0	0
0	0	0	0	0	0	0	0
0	0	0	0	0	0	0	0
0	0	14	0	0	0	0	0
0	0	43	0	0	0	0	0
0	0	50	0	0	3	0	0
0	0	4	1	0	0	0	0
0	0	8	2	0	0	0	0
0	0	0	0	0	0	0	0
0	0	0	0	0	0	0	0
0	0	0	0	0	0	0	0
0	0	0	0	0	0	0	0
0	0	0	0	0	0	0	0

0	0	0	0	0	0	0	0
0	0	5	1	0	0	0	0
0	0	1	22	0	0	0	0
0	0	0	0	0	0	0	0
0	0	1	1	0	0	0	0
0	0	1	22	0	0	0	0
0	0	0	0	0	4	0	0
0	0	2	1	0	0	0	0
0	0	0	0	0	0	0	0
0	0	2	1	0	0	0	0
0	0	6	1	0	1	0	1
0	0	0	0	0	0	0	0
0	0	3	1	0	0	0	0
0	0	0	0	0	0	0	0
0	0	2	0	0	1	0	0
0	0	1	0	0	0	0	0
0	0	3	2	0	0	0	0
0	0	0	1	0	0	0	0
0	0	0	2	0	0	0	0
0	0	0	0	0	0	0	0

0	0	3	0	0	1	0	0
0	0	0	0	0	0	0	0
0	0	2	0	0	0	0	0
0	0	0	0	0	0	0	0
0	0	3	1	0	0	0	0
0	0	0	0	0	0	0	0
0	0	0	0	0	0	0	0
0	0	0	0	0	0	0	0
0	0	0	0	0	0	0	0
0	0	3	2	0	0	0	0
0	0	0	3	0	0	0	0
0	0	11	1	0	0	0	0
0	0	6	3	0	1	0	0
6	0	0	2	0	0	0	0
0	0	5	2	0	0	0	0
0	0	4	0	0	2	0	0
0	0	0	0	0	0	0	0
0	0	0	0	0	0	0	0
0	0	1	0	0	0	0	0
0	0	1	6	0	0	0	0
0	0	1	1	0	0	0	0
0	0	0	0	0	0	0	0
0	0	0	0	1	0	0	0
0	0	28	0	0	0	0	0
0	0	14	2	0	0	0	0
0	0	0	0	0	0	0	0
0	0	1	1	0	0	0	0
0	0	10	0	0	0	0	0
0	0	0	0	0	0	0	0
0	0	0	0	1	0	0	0
0	0	0	0	0	0	0	0
0	0	6	1	0	0	0	0
0	0	3	0	0	0	0	0
0	0	1	6	0	0	0	0
0	0	6	0	0	0	0	0
0	0	5	1	0	0	0	0
0	0	0	0	0	0	0	0
0	0	3	7	0	0	0	0
0	1	12	0	0	0	0	0
0	0	1	1	0	0	0	0
0	0	0	0	0	0	0	0
0	0	7	7	0	0	0	0
0	0	7	0	0	7	0	0
0	0	17	0	0	0	0	0
0	0	5	2	0	0	0	0
0	0	0	0	0	0	0	0
0	0	6	0	0	0	0	0
0	0	3	1	0	0	0	0
0	0	4	4	0	0	0	0
0	0	0	0	0	0	0	0
0	0	3	1	0	0	0	0
0	0	0	1	0	0	0	0
0	0	2	1	0	0	0	0
0	0	7	25	0	0	0	0
0	0	0	1	0	0	0	0

0	0	15	0	0	0	0	0
0	0	2	3	0	0	0	0

0	1	1	0	0	0	0	0
0	0	2	0	0	0	0	0
0	0	0	0	0	0	0	0
0	0	8	9	0	0	0	1
0	0	7	0	0	0	0	0
0	0	1	0	0	1	0	0
0	0	1	4	0	0	0	0
0	0	0	1	0	0	0	0
0	0	2	0	0	0	0	0
0	0	1	0	0	0	0	0
0	0	0	0	0	0	0	0
0	0	0	0	0	0	0	0
0	0	14	0	0	0	0	0
0	0	4	0	0	0	0	0
0	0	2	0	0	0	0	0
0	0	12	0	0	3	0	0
0	0	0	0	0	0	0	0
0	0	1	2	0	0	0	0
0	0	1	0	0	0	0	0
0	0	10	0	0	0	0	0
0	0	8	0	1	0	0	0
0	1	9	0	0	0	0	0
0	0	2	5	0	0	0	0
0	0	0	0	0	0	0	0
0	0	0	0	0	0	0	0
0	0	0	0	0	0	0	0
0	0	0	0	0	0	0	0
0	0	0	2	0	0	0	0
0	0	4	0	0	0	0	0
0	0	0	0	0	0	0	0
0	0	6	0	0	0	0	0
0	0	3	7	0	0	0	0
0	0	0	0	1	0	0	1
0	0	0	8	0	1	0	0
0	0	7	6	0	0	0	0
0	0	2	0	0	0	0	0
0	0	0	0	0	0	0	0
0	0	3	1	0	0	0	0
0	0	3	0	0	0	0	0
0	0	4	2	0	0	0	0
0	0	1	2	0	0	0	0
0	0	0	0	0	0	0	0
0	0	1	0	0	0	0	0

0	0	0	0	0	0	0	0
0	0	0	0	0	0	0	0
0	0	1	0	0	0	0	0
0	0	1	0	0	0	0	0
0	0	0	0	0	0	0	0
0	0	12	1	0	0	0	0
0	0	0	0	0	0	0	0

asp_niger_sol	asp_sp_sol	asp_terreus_sol	asp_flavus_sol	peni_sol	clado_sol	alterna_sol	ulocla_sol
0	0	0	0	0	0	0	0
0	0	0	0	0	0	0	0
0	0	0	0	0	0	0	0
0	0	0	0	1	0	0	0
0	0	0	0	2	0	0	0
0	0	0	0	2	2	0	0
0	0	0	0	0	0	0	0
0	0	0	0	0	0	0	0
0	0	0	0	0	0	0	0
0	0	0	0	0	0	0	0
0	0	0	0	0	0	0	0
0	0	0	0	0	0	0	0
0	0	0	0	2	0	1	0
1	0	0	0	0	0	4	0
1	11	0	0	6	0	0	0
0	4	0	0	0	1	0	0
0	0	0	0	5	0	0	0
0	7	0	0	3	0	0	0
0	0	0	0	0	1	0	0
0	0	0	0	6	0	0	0
0	0	0	0	5	0	0	0
0	0	0	0	0	0	0	0
0	0	0	0	1	0	0	0
0	0	0	0	0	1	0	0
0	0	0	0	0	0	0	0
0	0	0	0	1	0	0	0
0	0	0	0	0	0	0	0
0	0	0	0	0	0	0	0
0	0	0	0	0	0	0	0
0	0	0	0	1000	0	0	0
0	0	0	0	0	12	0	0
0	7	0	0	10	0	0	0
0	0	0	0	0	0	0	0
0	6	0	0	0	0	0	0
0	0	0	0	4	0	0	0
0	0	0	0	3	0	1	0
0	0	0	0	7	0	2	0
0	0	0	0	31	0	0	0
0	0	0	0	0	0	0	0
0	0	0	0	0	0	0	0
0	1	0	0	0	0	0	0
0	0	0	0	0	0	0	0
0	1	0	0	1	0	0	0
0	0	0	0	1	0	0	0
1	0	0	0	0	0	3	0
1	0	0	1	5	0	4	0
0	0	0	0	21	2	0	0
0	0	0	0	0	1	1	0
0	0	0	0	14	0	0	0
0	0	0	0	2	2	1	0
0	0	0	0	0	0	0	0
0	0	0	0	0	0	0	0
0	0	0	0	11	0	0	0
0	0	0	0	3	0	0	1
0	0	0	0	0	0	1	0

0	0	0	0	0	0	0	0
0	0	0	0	1	0	0	0
0	0	0	0	0	0	0	0
0	0	0	0	0	0	0	0
0	0	0	0	0	0	0	0
0	0	0	0	1	1	0	0
0	0	0	0	0	0	0	0
0	0	0	0	0	0	0	0
0	0	0	0	0	0	1	0
0	0	0	0	0	0	0	0
0	0	0	0	1	0	0	0
0	0	0	0	0	0	0	0
0	0	0	0	0	0	0	0
0	0	0	0	0	0	0	0
0	0	0	0	0	0	0	0
0	0	0	0	0	0	0	0
0	0	0	0	0	0	0	0
0	0	0	0	0	1	0	0
0	0	0	0	0	0	0	0
0	0	0	0	0	0	0	0
0	6	0	0	3	0	0	0
0	0	0	0	0	0	0	0
0	0	0	0	0	0	1	0
0	0	0	0	0	0	0	0
0	0	0	0	0	0	0	0
0	2	0	0	1	0	0	0
0	0	0	0	0	1	0	0
0	0	0	0	1	0	0	0
0	0	0	0	0	0	0	0
0	0	0	0	0	0	0	0
0	0	0	0	0	0	0	0
0	1	0	0	0	0	3	0
0	0	0	0	0	0	0	0
0	0	0	0	0	0	1	0
0	0	0	0	0	0	0	0
0	0	0	0	2	0	0	0

0	0	0	0	0	0	0	2
0	0	0	0	0	0	0	0
0	0	0	0	0	1	0	2
5	1	0	0	0	0	2	0
0	0	0	0	1	2	0	0
1	0	0	0	15	0	0	0
0	0	0	0	0	0	1	0
0	0	0	0	0	0	0	0
0	2	0	0	3	2	0	0
0	0	0	0	0	0	0	0
0	0	0	0	7	0	0	0
0	0	0	0	1	0	0	0
1	0	0	0	1	0	3	0
0	0	0	0	5	0	1	0
0	0	0	0	0	0	0	0
0	0	0	0	0	0	0	0
0	0	0	0	0	0	0	0
0	0	0	0	0	0	0	0
0	0	0	0	0	1	0	0

0	0	0	0	0	0	0	0
0	0	0	0	2	0	0	0
0	0	0	0	0	0	0	0
0	0	0	0	0	0	0	0
0	0	0	0	0	0	0	0
0	0	0	0	0	0	0	0
0	0	0	0	0	0	0	0
1	0	0	0	0	0	0	0
0	0	0	0	0	0	0	0
0	0	0	0	2	2	0	0
0	0	0	0	5	0	0	0
0	0	0	0	1	0	1	0
0	0	0	0	1	0	0	0
0	2	0	0	1	2	0	0
0	0	0	0	0	0	0	0
0	0	0	0	1	0	0	0
0	0	0	0	0	0	0	0
0	0	0	0	0	3	0	0
0	0	0	0	0	0	1	0
0	0	0	0	1	0	0	0
0	0	0	0	1	0	0	0
0	0	0	0	1	0	0	0
0	0	0	0	0	0	1	0
0	0	0	0	2	0	0	0
0	0	0	0	0	0	0	0
0	0	0	0	2	0	0	0
0	1	0	0	0	0	0	0
0	0	0	0	0	0	0	0
0	0	0	0	0	0	0	0
0	0	0	0	0	0	0	1
0	0	0	0	0	0	0	0
0	0	0	0	0	0	0	0
0	0	0	0	0	0	0	0
0	0	0	0	0	0	0	0
0	0	0	0	0	1	0	0
0	0	0	0	1	0	0	0
0	0	0	0	49	0	0	0
0	0	0	0	2	1	0	0
0	0	0	0	0	0	0	0
0	1	0	0	0	0	1	0
0	0	0	0	0	0	0	0
0	0	0	0	0	0	0	0
0	0	0	0	0	0	0	0
0	0	0	0	0	0	0	0
0	0	0	0	1	0	0	0
0	0	0	0	0	0	0	0
0	0	0	0	0	0	0	0
0	0	0	0	0	0	0	0
0	0	0	0	1	0	0	0
0	0	0	0	0	0	1	0
0	0	0	0	0	0	0	0
0	0	0	0	0	0	0	0
0	0	0	0	0	0	0	0
0	0	0	0	0	0	0	0

0	0	0	0	0	0	0	0
0	0	0	0	0	0	0	0
0	0	0	0	0	0	0	0
0	0	0	0	0	1	0	0
1	0	0	0	0	0	1	0
0	0	0	0	0	0	0	0
0	0	0	0	0	0	0	1
0	0	0	0	1	0	0	0
0	0	0	0	7	0	5	0
0	2	0	0	1	0	0	0
0	0	0	0	7	0	0	0
0	0	0	0	0	0	0	0
0	0	0	0	0	0	0	0
0	0	0	0	0	0	0	0

0	0	0	0	1	1	1	0
0	0	0	0	0	0	0	0
0	0	0	0	0	0	1	0
0	0	0	0	0	1	11	0
0	0	0	0	0	2	0	0
0	0	0	0	1	7	1	0
0	0	0	0	10	0	3	0
0	0	0	0	0	0	1	0
0	0	0	0	0	0	0	0

0	0	0	0	0	0	0	0
0	0	0	0	0	0	0	0
0	0	0	0	0	0	0	0
0	0	0	0	0	0	0	0
0	0	0	0	0	0	0	0
0	0	0	0	2	0	0	0
0	0	0	0	0	0	0	0
0	0	0	0	0	0	1	0
0	0	0	0	0	0	1	0
0	0	0	0	5	16	0	0
0	0	0	0	1	0	0	0
0	0	0	0	0	0	0	0
0	0	0	0	0	0	0	0
0	0	0	0	0	0	0	0
0	0	0	0	0	0	0	0
0	0	0	0	0	0	0	0
0	0	0	0	0	0	0	0
0	0	0	0	0	0	0	0
0	0	0	0	0	0	0	0
0	0	0	0	0	0	0	0
0	0	0	0	1	1	1	0
0	0	0	0	0	0	0	0
0	0	0	0	0	0	0	0
0	0	0	0	0	0	0	0
0	0	0	0	0	0	0	0
0	0	0	0	0	0	1	0
0	0	0	0	0	0	0	0
0	0	0	0	0	0	0	0
0	0	0	0	0	0	0	0

0	3	0	0	29	0	0	0
0	0	0	0	0	0	0	0
0	0	0	0	0	0	0	0
0	0	0	0	9	0	0	0
0	0	0	0	0	0	0	0
0	0	0	0	25	0	0	1
0	1	0	0	0	0	0	0
0	0	0	0	0	0	0	0
0	1	0	0	0	0	0	0
0	0	0	0	1	0	0	0
0	0	0	0	0	0	0	0
0	0	0	0	0	0	0	0
0	0	0	0	0	0	0	0
0	0	0	0	0	0	0	0
0	0	0	0	0	0	0	0
0	0	0	0	0	0	0	0
0	0	0	0	0	0	0	0
0	0	0	0	1	1	0	0
0	0	0	0	0	0	0	0
0	0	0	0	0	2	0	0
0	0	0	0	0	0	0	0
0	0	0	0	3	0	0	0
0	0	0	0	0	0	0	0
0	0	0	0	0	0	0	0
0	0	0	0	2	0	1	0

0	0	0	0	0	0	0	4
0	0	0	0	0	0	0	0
0	0	0	0	0	0	0	0
0	0	0	0	0	0	0	0
0	0	0	0	1	0	0	0
0	0	0	0	0	0	0	0
0	0	0	0	0	0	0	0
0	1	0	0	0	0	0	0
0	0	0	0	0	0	0	0
0	0	0	0	0	0	0	0
0	0	0	0	1	0	0	0
0	0	0	0	0	0	0	0
0	0	0	0	0	0	0	0
0	0	0	0	0	0	0	0
0	0	0	0	0	1	0	0
0	0	0	0	0	0	0	0
0	0	0	0	2	0	0	0
0	0	0	0	0	0	0	0
0	0	0	0	0	0	0	0
0	0	0	0	0	0	1	0
0	0	0	0	0	0	0	0
0	0	0	0	0	0	1	0
0	0	0	0	0	0	0	0
0	0	0	0	1	0	0	0

0	0	0	0	0	0	1	0
0	0	0	0	0	0	0	0
0	0	0	0	1	1	0	0
0	0	0	0	0	0	1	0
0	0	0	0	0	0	1	0

0	0	0	1	0	0	0	0
0	0	0	0	0	0	0	0
0	0	0	0	0	0	0	0
0	0	0	0	0	0	0	0
0	1	0	0	0	0	0	0
0	0	0	0	0	0	0	0
0	0	0	0	0	0	0	0
0	0	0	0	0	0	0	0
0	0	0	0	0	1	0	0
0	0	0	0	0	0	0	0
0	0	0	0	0	0	4	0
0	0	0	0	0	1	0	0
0	0	0	0	0	0	0	0
0	0	0	0	0	0	0	0
0	0	0	0	0	1	0	0
0	0	0	0	0	0	1	0
0	0	0	0	0	0	0	0
0	1	0	0	0	0	2	0
0	0	0	0	0	0	1	0
0	0	0	0	0	0	0	0
0	0	0	0	0	0	0	0
0	0	0	0	0	0	0	0
0	0	0	0	0	0	0	0
0	0	0	0	0	0	0	0
0	1	0	0	0	0	0	0
0	0	0	0	0	0	0	0
0	0	0	0	1	0	1	0
0	0	0	0	0	0	0	0
0	0	0	0	0	0	0	0
0	0	0	0	0	0	0	0
0	0	0	0	0	0	0	0
0	0	0	0	0	0	0	0

0	0	0	0	0	0	0	0
0	0	0	0	1	0	0	0
0	0	0	0	0	0	0	0
0	0	0	0	0	0	0	0
0	0	0	0	0	0	0	0
0	0	0	0	3	0	0	0
0	3	0	0	17	0	0	0
0	0	0	0	0	0	0	0
0	0	0	0	0	0	0	0
0	0	0	0	0	0	0	0
0	0	0	0	0	0	0	0
0	0	0	0	0	0	0	0
0	1	0	0	0	0	0	0
0	0	0	0	17	0	0	0
0	0	0	0	0	0	0	0
0	0	0	0	1	0	0	0
0	0	0	0	0	0	0	0
0	0	0	0	0	0	0	0
0	0	0	0	0	0	0	0
1	0	0	0	0	0	0	0

0	23	0	0	47	0	0	0
0	0	0	0	0	0	0	0
0	0	0	0	36	0	0	0
0	0	0	0	17	0	0	0
0	1	0	0	0	0	0	0
0	0	0	0	2	0	0	0
0	0	0	0	0	0	0	0
0	0	0	0	0	0	0	0
0	0	0	0	0	1	0	0
0	0	0	0	0	0	0	0
0	1	0	0	1	1	0	0
0	0	0	0	0	0	0	0
0	0	0	0	2	0	0	0
0	0	0	0	1	0	0	0
0	0	0	0	0	0	0	0
0	0	0	0	3	0	0	0
0	0	0	2	0	0	0	0
0	0	0	0	0	1	0	0
0	0	0	0	0	0	0	0
0	0	0	0	0	0	0	0
0	0	0	0	0	0	0	0
0	0	0	0	0	0	0	0
0	0	0	0	0	0	0	0
0	0	0	0	0	0	0	0
0	0	0	0	0	1	0	0
0	0	0	0	0	0	0	0
0	0	0	0	1	0	0	0
0	0	0	0	0	0	1	0
0	0	0	0	0	0	0	0
0	0	0	0	0	0	0	0
0	0	0	0	0	0	0	0
0	0	0	0	0	0	0	0
0	0	0	0	0	0	0	0
0	0	0	0	0	0	0	0
0	0	0	0	0	0	0	0
0	0	0	0	0	0	0	0
0	0	0	2	3	0	0	0
0	0	0	0	0	0	1	0
0	0	0	0	0	0	2	0
0	0	0	0	0	0	1	0
0	0	0	0	0	0	1	0
0	0	0	0	0	0	0	0
0	0	0	0	1	0	0	0
0	0	0	0	2	0	0	0
0	0	0	0	1	0	0	0
0	1	0	0	16	1	1	0
0	0	0	0	0	1	0	0
0	0	0	0	0	1	0	0
0	0	0	0	1	0	0	2
0	0	0	0	0	0	0	0
0	0	0	0	0	1	0	1
0	0	0	0	0	0	1	0
0	0	0	0	0	0	0	0
1	0	0	0	124	1	1	0
0	0	0	0	2	2	1	0

0	0	0	0	0	0	0	0
0	0	0	0	5	2	0	0
0	0	0	0	0	1	0	0
0	0	0	0	0	0	0	0
0	0	0	0	0	0	0	0
0	0	0	0	0	0	0	1
0	0	0	0	7	0	0	0
0	0	0	0	0	0	1	0
0	0	0	0	0	2	0	1
0	0	0	0	0	0	0	0
0	0	0	0	0	1	0	0
0	0	0	0	0	0	0	0
0	0	0	0	0	0	0	0
0	0	0	0	0	1	0	0
0	0	0	0	0	0	0	0
0	0	0	0	0	0	0	0
0	0	0	0	0	0	1	0
0	0	0	0	0	0	0	0
0	0	0	0	0	0	0	0
0	0	0	0	0	0	0	0
0	0	0	0	0	0	0	0
0	0	0	0	0	0	1	0
0	0	0	1	0	0	0	0
0	0	0	0	0	0	0	0
0	0	0	0	0	0	1	0
0	0	0	0	1000	0	0	0
0	0	0	0	1000	0	0	0
0	0	0	0	1	0	0	0
0	0	0	0	0	0	0	0
0	0	0	0	0	0	0	0
0	0	0	0	0	0	0	0
0	0	0	0	13	1	3	0
0	0	0	0	0	0	0	0
0	0	0	0	0	0	0	0
0	0	0	0	1	1	2	0
0	0	0	0	0	0	1	0
0	0	0	0	0	0	0	0
0	5	0	0	3	0	0	0
0	0	0	0	9	4	0	0
0	0	0	0	3	0	0	2
0	1	0	0	1	0	2	0
0	0	0	0	0	0	1	0
1	0	0	0	83	0	0	0
0	0	0	0	0	0	1	0
0	0	0	0	0	0	0	0
0	0	0	0	0	0	0	0
0	0	0	0	1	0	6	0
0	0	0	0	0	0	0	0
0	0	0	0	0	0	0	0
0	0	0	0	0	0	2	0
0	0	0	0	1	0	0	0
0	0	0	0	0	0	0	0

chaeto_sol	scopula_sol	acremo_sol	fusa_sol	rhizo_sol	mucor_sol	geotri_sol	trichoderm_sol
0	0	0	0	0	0	0	0
4	1	0	0	0	0	0	0
0	0	0	0	0	0	0	0
0	0	0	0	0	0	0	0
0	0	0	0	0	0	0	0
0	0	0	0	0	0	0	0
0	0	0	0	0	0	0	0
0	0	0	0	0	0	1	0
0	0	0	0	0	0	0	0
0	0	0	0	0	0	0	0
0	0	0	0	0	0	0	0
0	0	0	0	0	0	0	0
0	0	0	0	0	0	0	0
0	0	0	0	0	0	0	0
0	0	0	0	0	0	0	0
0	0	0	0	0	0	0	0
0	0	0	0	0	0	0	0
0	0	0	0	0	0	0	0
0	0	0	0	0	0	0	0
0	0	0	0	0	0	0	0
0	0	0	0	0	0	0	0
0	0	0	0	0	0	0	0
0	0	0	0	0	0	0	0
0	0	0	0	0	0	0	0
0	0	0	0	0	0	0	0
0	0	0	0	0	0	0	0
0	0	0	0	0	0	0	0
0	0	0	0	0	0	0	0
0	0	0	0	0	0	0	0
0	0	0	0	0	1000	0	0
0	0	0	0	0	0	0	0
2	0	0	0	0	0	0	0
1	0	0	0	0	0	0	0
0	0	0	0	0	0	0	0
0	0	0	0	0	0	0	1
0	0	0	0	0	0	0	0
0	0	0	0	0	0	0	0
0	0	0	0	0	0	0	0
0	0	0	0	0	0	0	0
0	0	0	0	0	0	0	0
0	0	0	0	0	0	0	0
0	0	0	0	0	0	0	0
0	0	0	0	1	0	0	0
0	0	0	0	0	0	0	0
0	0	0	0	0	0	0	0
0	0	0	0	1	0	0	0
0	0	0	0	0	0	0	0
0	0	0	0	0	0	0	0
1	0	0	0	1	0	1	0
0	0	0	0	0	0	0	0
0	0	0	0	0	0	0	0
0	0	0	0	0	0	0	0
0	0	0	0	0	0	0	0
0	0	0	0	0	0	0	0
0	0	0	0	0	0	0	0

0	0	0	0	0	0	0	0
0	0	0	0	0	0	0	0
0	0	0	0	0	0	0	0
0	0	0	0	0	0	0	0
0	0	0	0	0	0	0	0
0	0	0	0	0	0	0	0
0	0	0	0	0	0	0	0
0	0	0	0	0	0	0	0
0	0	0	0	0	0	0	0
0	0	0	0	0	0	0	0
0	0	0	0	0	0	0	0
0	0	0	0	0	0	0	0
0	0	0	0	0	0	0	0
0	0	0	0	0	0	0	0
0	0	0	0	0	0	0	0
0	0	0	0	0	0	0	1
0	0	0	0	0	0	0	1
0	0	0	0	0	0	0	0
0	0	0	0	0	0	0	0
0	0	0	0	0	0	0	0
0	0	0	1	0	0	0	0
0	0	0	0	0	0	0	0
0	0	0	0	0	0	0	0
0	0	0	0	0	0	0	0
0	0	0	0	0	0	0	0
0	0	0	0	0	0	0	0
0	0	0	0	0	0	0	0
0	0	0	0	0	0	0	0
0	0	0	0	0	0	0	0
0	0	0	0	0	0	0	0
0	0	0	0	0	0	0	0
0	0	0	0	0	0	0	0
0	0	0	0	0	0	0	0
3	0	0	0	0	0	0	0
1	0	0	0	0	0	0	0
0	0	0	0	0	0	0	0

0	0	0	0	0	0	0	0
0	1	0	0	0	0	0	0
0	0	0	0	0	0	0	0
0	0	0	0	0	0	0	0
0	0	0	0	0	0	0	0
0	0	0	0	1	0	0	1
0	0	1	0	0	0	0	0
0	0	0	0	0	0	0	0
0	0	0	0	0	0	0	0
0	0	0	0	0	0	0	0
0	0	0	0	0	0	0	0
0	0	0	0	0	0	0	0
0	0	0	0	0	0	0	0
0	0	0	0	0	0	0	0
0	0	0	0	0	0	0	0
6	0	0	0	0	0	0	0
0	0	0	0	0	0	0	0
0	0	0	0	0	0	0	0
0	0	0	0	0	0	0	0

0	0	0	0	0	0	0	0
0	0	0	0	0	0	0	0
0	0	0	0	0	0	0	0
0	0	0	0	0	0	0	0
0	0	0	0	0	0	0	0
0	0	0	0	1000	0	0	0
0	0	0	0	0	0	0	0
0	0	0	0	0	0	0	0
0	0	0	0	0	0	0	0
0	0	0	0	0	0	0	0
0	0	0	0	0	0	0	0
0	0	0	0	0	0	0	1
0	0	0	0	0	0	0	0
0	0	0	0	0	0	0	0
0	0	0	0	0	0	0	0
0	0	0	0	0	0	0	0
0	0	0	0	0	0	0	0
0	0	0	0	0	0	0	0
0	0	0	0	0	0	0	0
0	0	0	0	0	0	0	0
0	0	0	0	0	0	0	0
0	0	0	0	0	0	0	0
0	0	0	0	0	0	0	0
0	0	0	0	0	0	1	0
0	0	0	0	0	0	0	0
0	0	0	0	0	0	0	0
0	0	0	0	0	0	0	0
0	0	0	0	0	0	0	0
0	0	0	1000	0	0	0	0
0	0	0	0	0	0	0	0
0	0	0	0	0	0	0	0
0	0	0	0	0	0	0	0
0	0	0	0	0	0	0	0
0	0	0	0	0	0	0	0
0	0	0	0	0	0	0	0

0	0	0	0	0	0	0	0
0	0	0	0	0	0	0	0
0	0	0	0	0	0	0	0
0	0	0	0	0	0	0	0
1	0	1	0	0	0	0	0
0	0	0	0	0	0	0	0
0	0	0	0	0	0	0	0
0	0	0	0	0	0	0	0
0	0	0	0	0	0	0	0
0	0	0	0	0	0	0	0
0	0	0	0	0	0	0	0
0	0	0	0	0	0	0	0
0	0	1	0	0	0	0	0
0	0	0	0	0	0	0	0

0	0	0	0	0	0	0	0
0	0	0	0	0	0	0	0
0	0	0	0	0	0	0	0
0	0	0	0	0	0	0	0
0	0	0	0	0	0	0	0

0	0	0	0	0	0	0	0
0	0	0	0	0	0	0	0
0	0	0	0	0	0	0	0
0	0	0	0	0	0	0	0
0	0	0	0	0	0	0	0
0	0	0	0	0	1	0	0
0	0	0	0	0	0	0	0
0	0	0	0	0	0	0	0
0	0	0	0	0	1000	0	0
0	0	0	0	0	0	0	0
0	0	0	0	1	0	0	0
0	0	0	0	0	0	0	0
0	0	0	0	0	0	0	0
0	0	0	0	0	0	0	0

0	0	0	0	0	0	0	0
0	0	0	0	0	0	0	0
0	0	0	0	0	0	0	0
0	0	0	0	0	0	0	0
0	0	0	0	0	0	0	0
0	0	0	0	0	0	0	0
0	0	0	0	0	0	0	0
0	0	0	0	0	0	0	0
0	0	0	0	0	0	0	0

0	0	0	0	0	0	0	0
0	0	0	0	0	0	0	0
0	0	1	0	0	1	0	0
0	0	0	0	0	0	0	0
0	0	0	0	0	0	0	0
0	0	0	0	0	0	0	0
0	0	0	0	0	0	0	0
0	0	0	0	0	0	0	0
0	0	0	0	0	0	0	0
0	0	0	0	0	0	0	0
0	0	0	0	0	0	0	0
0	0	0	0	0	0	0	0
0	0	0	0	0	0	0	0
0	0	0	0	0	0	0	0
0	0	0	0	0	0	0	0
0	0	0	0	0	0	0	0
0	0	0	0	0	0	0	0
0	0	0	0	0	0	0	0
0	0	0	0	0	0	0	0
0	0	0	0	0	0	1	0
0	0	0	0	0	0	0	0
1	0	0	0	0	0	0	0
0	0	0	0	0	0	0	0
0	0	0	0	0	0	0	0
0	0	0	0	0	0	0	0
0	0	0	0	0	0	0	0
0	0	0	0	0	0	0	0
0	0	0	0	0	0	0	0
0	0	0	0	0	0	0	0

0	0	0	0	0	0	0	0
0	0	0	0	0	0	0	0
0	0	0	0	0	0	0	0
0	0	0	0	0	0	0	0
0	0	0	0	1000	0	0	0
2	0	0	0	0	0	0	0
0	0	0	0	0	0	0	0
0	0	0	0	0	0	0	0
0	0	0	0	0	0	0	0
0	0	0	0	0	0	0	0
0	0	0	0	0	0	0	0
0	0	0	0	0	0	0	0
0	0	0	0	0	0	0	0
0	0	0	0	0	0	0	0
0	0	0	0	0	0	0	0
0	0	0	0	0	0	0	0
0	0	0	0	0	0	0	0
0	0	0	0	0	0	0	0
0	0	0	0	0	0	0	0
0	0	0	0	0	0	0	0
0	0	0	0	0	0	0	0
0	0	0	0	0	0	0	0
0	0	0	0	0	0	0	0
0	0	0	0	0	0	0	0
0	0	0	0	0	0	0	0

0	0	0	0	0	0	0	0
0	0	0	0	0	0	0	0
0	0	0	0	0	0	0	0
0	0	0	0	0	0	0	0
0	0	0	0	0	0	0	0
0	0	0	0	0	0	0	0
0	0	0	0	0	0	0	0
0	0	0	0	0	0	0	0
0	0	0	0	0	0	0	0
0	0	0	0	0	0	0	0
0	0	0	0	0	0	0	0
2	0	1	0	0	0	0	0
0	0	0	0	0	0	0	0
0	0	0	0	0	0	0	0
0	0	1	0	0	0	0	0
0	0	0	0	0	0	0	0
0	0	0	0	0	0	0	0
0	0	0	0	0	0	0	0
0	0	0	0	0	0	0	0
0	0	0	0	0	0	0	0
0	0	3	0	0	0	0	0
0	0	0	0	0	0	0	0
0	0	0	0	0	0	0	0
0	0	0	0	0	0	0	0

0	0	0	0	0	0	0	0
0	0	0	0	0	0	0	0
0	0	0	0	0	0	0	0
0	0	0	0	0	0	0	0
0	0	0	0	0	0	0	0

0	0	0	0	0	0	0	0
0	0	0	0	0	0	0	0
0	0	0	0	0	0	0	0
0	0	0	0	0	0	0	0
0	0	0	0	0	0	0	0
0	0	0	0	0	0	0	0
0	0	0	0	0	0	0	0
0	0	0	0	0	0	0	0
0	0	0	0	0	0	0	0
0	0	0	0	0	0	0	0
0	0	0	0	0	0	0	0
0	0	0	0	0	0	0	0
0	0	0	0	0	0	0	0
0	0	0	0	0	0	0	0
0	0	0	0	0	0	0	0
0	0	0	0	0	0	0	0
0	0	0	0	0	0	0	0
0	0	0	0	0	0	0	0
0	0	0	0	0	0	0	0
0	0	0	0	0	0	0	0
0	0	0	0	0	0	0	0
0	0	0	0	0	0	0	0
0	0	0	0	0	0	0	0
0	0	0	0	0	0	0	0
0	0	0	0	0	0	0	0
0	0	0	0	0	0	0	0
0	1	0	0	0	0	0	0
0	0	0	0	0	0	0	0
0	0	0	0	0	0	0	0
0	0	0	0	0	0	0	0
0	0	0	0	0	0	0	0
0	0	0	0	0	0	0	0

0	0	0	0	0	0	0	0
0	0	1	0	0	0	0	0
0	0	0	0	0	0	0	0
0	0	0	0	0	0	1	0
0	0	0	0	0	0	0	0
0	0	0	1	0	0	0	0
0	0	0	0	0	1	0	0
0	0	0	0	0	0	0	0
0	0	0	0	0	0	0	0
0	0	0	0	0	0	0	0
0	0	0	0	0	0	0	0
0	0	0	0	0	0	0	0
0	0	0	0	0	0	0	0
0	0	0	0	0	0	0	0
0	0	0	0	0	0	0	0
0	0	0	0	0	0	0	0
0	0	0	0	0	0	0	0
0	0	0	0	0	0	0	0
0	0	0	0	0	0	0	0
0	0	0	0	0	0	0	0

3	0	0	0	0	0	0	0
4	0	0	0	0	0	0	0
34	0	0	0	0	0	0	0
0	0	0	0	0	0	0	0
0	0	0	0	0	0	0	0
0	0	0	0	0	0	0	0
0	0	0	0	0	0	0	0
0	0	0	0	0	0	0	0
0	0	0	0	0	0	0	0
0	0	0	0	0	0	0	0
0	0	0	0	0	0	0	0
0	0	0	0	0	0	0	0
0	0	0	0	0	0	0	0
0	0	0	0	0	0	0	0
0	0	1	0	0	0	0	0
0	1	0	0	0	0	0	0
0	0	0	0	0	0	0	0
0	0	0	0	0	0	0	0
0	0	0	0	0	0	0	0
0	0	0	0	0	0	0	0
0	0	0	0	0	0	0	0
0	0	0	0	0	0	0	0
0	0	0	0	0	0	0	0
0	0	0	0	0	0	0	0
0	0	0	0	0	0	0	0
0	0	0	0	0	0	0	0
0	0	0	0	0	0	0	0
0	0	0	0	0	0	0	0
0	0	0	0	0	0	0	0
0	0	0	0	0	0	0	0
0	0	0	0	0	0	0	0
0	0	1	0	0	0	0	0
0	0	0	0	0	0	0	0
0	0	0	0	0	0	0	0
0	0	0	0	0	0	0	0
0	0	0	0	0	0	0	0
0	1	0	0	0	0	0	0
0	0	0	0	0	0	0	0
0	0	0	0	0	0	0	0
0	0	0	0	0	0	0	0
0	0	0	0	0	0	0	0
0	0	0	0	1	0	0	0
0	0	0	0	1	0	0	0
0	0	0	0	0	0	0	0
0	0	0	0	0	0	0	0
0	0	0	0	0	0	0	0
0	0	0	0	0	0	0	0
0	0	0	0	0	0	0	0
0	0	0	0	0	0	1000	0
0	0	0	0	1	0	0	0
0	0	0	0	0	0	0	0
0	0	0	0	0	0	0	0
0	0	0	0	0	0	0	0
0	0	0	0	0	0	0	0
0	0	0	0	0	0	0	0

0	0	0	0	0	0	0	0
0	0	0	0	0	0	0	0
0	0	0	22	0	0	0	0
0	0	0	0	0	0	0	0
0	0	0	0	0	0	0	0
0	0	0	0	0	0	0	0
0	0	0	0	0	0	0	0
0	0	0	0	0	0	0	0
0	0	0	0	0	0	0	0
0	2	0	0	0	0	0	0
0	0	0	0	0	0	0	0
0	0	0	0	0	0	0	0
0	0	0	0	0	0	0	0
0	0	0	0	0	0	0	0
0	0	0	0	0	0	0	0
0	0	0	0	0	0	0	0
1	0	0	0	0	0	0	0
0	0	0	0	0	0	0	0
0	0	0	0	0	0	0	0
0	0	1	0	0	0	0	0
0	0	0	0	0	0	0	0
0	0	0	0	0	0	0	0
0	0	0	0	0	0	0	0
0	0	0	0	0	0	0	0
0	0	0	0	0	0	0	0
0	0	0	0	0	0	0	0
0	0	0	0	0	0	0	0
0	0	0	0	0	0	0	0
0	0	0	0	0	0	0	0
1	1	0	0	0	0	0	0
0	0	0	0	0	0	0	0
0	0	0	0	0	0	0	0
0	0	0	0	0	0	0	0
0	0	0	0	0	0	0	0
1	0	0	0	0	0	0	0
0	0	0	0	0	0	0	0
0	0	0	0	0	0	0	0
0	0	0	0	0	0	0	0
0	0	0	2	0	0	0	0
0	0	0	0	0	0	0	0
0	0	0	0	0	0	1	0
0	0	0	0	0	0	0	0
0	0	0	0	0	0	0	0
0	0	0	0	0	0	0	0
0	0	0	0	0	0	0	0
0	0	0	0	0	0	0	0
0	0	0	0	0	0	0	0
0	0	0	0	0	0	0	0
0	0	0	0	0	0	0	0
0	0	0	0	0	0	0	0
0	0	0	0	0	0	0	0
0	0	0	0	0	0	0	0

paecilo_sol	autres_sol	csd_sol	levure_sol	fenetre	asp_fumi_fetv	sct_nid_fetv	asp_nid_fetv
0	0	0	1	VRAI	0	0	0
0	0	1	0	VRAI	0	0	0
0	0	0	0	VRAI	0	0	0
0	2	15	8	FAUX	0	0	0
1	0	0	1	FAUX	0	0	0
0	0	0	4	FAUX	0	0	0
0	0	0	0	FAUX	0	0	0
0	0	0	0	FAUX	0	0	0
0	0	0	3	FAUX	0	0	0
0	0	0	1	FAUX	0	0	0
0	0	1	0	VRAI	0	0	0
0	0	1	0	VRAI	0	0	0
0	0	3	0	VRAI	0	0	0
0	0	39	4	FAUX	0	0	0
0	0	7	1	FAUX	0	0	0
0	0	3	5	FAUX	0	0	0
0	1	1	4	FAUX	0	0	0
1	0	2	0	FAUX	0	0	0
0	0	2	0	FAUX	0	0	0
0	0	0	0	FAUX	0	0	0
0	0	2	0	FAUX	0	0	0
0	0	0	2	FAUX	0	0	0
0	0	0	1	FAUX	0	3	0
0	0	2	2	FAUX	0	6	0
0	0	0	1	FAUX	0	7	0
0	0	1	21	FAUX	0	0	0
0	0	0	0	FAUX	0	0	0
0	0	0	1	FAUX	0	0	0
0	0	0	0	FAUX	0	0	0
0	0	0	0	VRAI	0	0	0
0	0	1	2	VRAI	0	0	0
0	0	4	0	VRAI	0	0	0
0	0	0	0	VRAI	0	0	0
0	0	2	2	VRAI	0	0	0
0	0	4	2	VRAI	0	0	0
0	0	2	5	FAUX	0	0	0
0	0	5	100	FAUX	0	0	0
0	0	2	1	FAUX	0	0	0
0	0	0	0	FAUX	0	0	0
0	0	5	0	FAUX	0	1	0
0	0	2	1	FAUX	0	0	0
0	0	3	9	VRAI	0	0	0
0	0	0	0	FAUX	0	0	0
0	0	1	1	FAUX	0	0	0
0	0	6	0	FAUX	0	0	0
0	0	6	0	FAUX	11	0	0
0	0	7	0	FAUX	0	0	0
0	0	0	1	FAUX	0	0	0
0	0	1	0	FAUX	0	0	0
0	0	1	4	FAUX	0	0	0
0	0	0	0	VRAI	0	0	0
0	0	0	0	VRAI	0	0	0
0	0	5	1	VRAI	0	0	0
0	0	1	0	VRAI	0	0	0
0	0	0	0	VRAI	0	0	0

0	0	0	1	VRAI	0	0	0
0	0	1	0	VRAI	0	0	0
0	0	0	0	VRAI	0	0	0
0	0	0	0	VRAI	0	0	0
0	0	0	0	VRAI	0	0	0
0	0	0	1	VRAI	3	2	0
0	0	0	2	FAUX	0	0	0
0	0	0	0	FAUX	1	0	0
0	0	0	0	FAUX	0	0	0
0	0	1	5	FAUX	0	0	0
0	0	1	0	FAUX	0	0	0
0	0	0	0	FAUX	0	0	0
0	0	1	0	FAUX	0	0	0
0	0	0	0	FAUX	0	0	0
0	0	2	3	FAUX	0	0	0
0	0	2	1	VRAI	0	0	0
0	0	3	1	VRAI	0	0	0
0	0	1	3	VRAI	0	0	0
0	0	0	18	VRAI	0	0	0
0	0	0	10	FAUX	0	0	0
0	0	2	1	FAUX	0	0	0
0	0	0	1	FAUX	0	0	0
0	0	1	1	FAUX	0	0	0
0	0	0	0	FAUX	0	0	0
0	0	0	1	FAUX	0	0	0
0	0	2	1	FAUX	0	0	0
0	0	1	0	FAUX	0	0	0
0	0	14	1	FAUX	0	0	0
0	0	0	0	FAUX	0	0	0
0	0	0	2	FAUX	0	0	0
0	0	0	2	FAUX	0	0	0
0	1	2	0	FAUX	0	0	0
0	0	0	0	VRAI	0	0	0
0	0	0	1	VRAI	0	0	0
0	0	0	1	VRAI	0	0	0
1	0	0	0	VRAI	0	3	0
0	0	1	1	VRAI	0	0	0
0	0	0	0	VRAI	0	0	0
0	0	1	2	FAUX	0	0	0
0	0	6	0	FAUX	0	0	0
0	0	4	1	FAUX	0	0	0
0	0	0	27	FAUX	0	0	0
0	0	1	6	FAUX	0	0	0
0	0	1	0	FAUX	0	0	0
0	0	2	0	FAUX	0	0	0
0	0	0	0	FAUX	0	0	0
0	0	2	0	FAUX	0	0	0
0	0	1	14	FAUX	0	0	0
0	0	1	1	FAUX	0	0	0
0	0	4	5	VRAI	0	0	0
0	0	0	1	VRAI	0	0	0
0	0	0	0	FAUX	0	0	0
5	0	1	0	FAUX	0	0	0
0	0	0	2	FAUX	0	0	0
0	0	0	0	VRAI	0	0	0

0	0	1	1	VRAI	0	0	0
0	0	2	0	VRAI	0	0	0
0	0	0	0	VRAI	0	0	0
0	0	0	0	VRAI	0	0	0
0	0	0	0	VRAI	0	0	0
0	0	0	0	VRAI	0	0	0
0	0	0	0	VRAI	0	0	0
0	0	0	36	VRAI	0	0	0
0	1	0	3	VRAI	0	1	0
0	0	4	2	VRAI	0	0	0
0	0	9	35	FAUX	0	0	0
0	0	1	1	VRAI	0	0	0
0	0	0	0	VRAI	0	0	0
0	0	2	1	VRAI	0	0	0
0	0	1	1	VRAI	0	0	0
0	0	1	5	VRAI	0	0	0
1000	0	0	0	FAUX	0	0	0
1	0	3	1	FAUX	0	0	0
0	0	0	5	FAUX	0	0	0
0	0	0	1	FAUX	0	0	0
0	0	0	1	FAUX	0	0	0
0	0	4	4	FAUX	0	0	0
0	0	5	4	FAUX	0	0	0
0	0	2	1	FAUX	0	0	0
0	0	1	3	FAUX	0	0	0
0	1	2	1	VRAI	0	0	0
0	0	1	2	VRAI	0	0	0
0	0	2	1	FAUX	0	0	0
0	0	2	0	FAUX	0	0	0
0	0	0	0	VRAI	0	0	0
0	0	1	0	VRAI	0	1	0
0	0	0	0	FAUX	0	0	0
0	0	0	1	FAUX	0	1	0
0	0	44	12	FAUX	0	1	0
0	0	6	5	FAUX	0	0	0
0	0	0	0	VRAI	0	0	0
0	0	0	0	VRAI	0	0	0
0	0	1	3	FAUX	0	0	0
0	1	2	0	FAUX	0	0	0
0	0	6	34	FAUX	0	0	0
0	0	1	0	FAUX	0	0	0
0	0	0	0	FAUX	0	0	0
0	0	0	1	FAUX	0	0	0
0	0	1	0	FAUX	0	0	0
0	0	0	4	VRAI	0	0	0
0	0	2	2	FAUX	0	6	0
0	0	0	0	FAUX	0	0	0
0	0	0	3	FAUX	0	0	0
0	0	0	1	FAUX	0	0	0
0	0	0	0	VRAI	0	1	0
0	0	0	2	VRAI	0	0	0
0	0	0	1	FAUX	0	0	0
0	0	0	0	FAUX	0	0	0
0	0	0	0	FAUX	0	0	0

0	0	2	0	FAUX	0	0	0
0	0	0	1	FAUX	0	0	0
0	0	1	1	FAUX	0	0	0
0	1	8	2	FAUX	0	0	0
0	1	1	22	FAUX	0	0	0
0	0	2	2	VRAI	0	0	0
0	0	7	1	FAUX	0	0	0
0	0	1	0	FAUX	0	0	0
0	0	1000	1000	FAUX	0	0	0
0	1	1	5	FAUX	0	0	0
0	0	2	3	FAUX	0	0	0
0	0	0	0	FAUX	0	0	0
0	0	0	0	FAUX	0	0	0
0	0	0	0	FAUX	0	0	0
0	0	4	5	VRAI	0	0	0
0	0	1	1	VRAI	0	0	0
0	0	4	5	FAUX	0	0	0
0	0	4	2	FAUX	0	0	0
0	0	6	0	FAUX	0	0	0
0	0	13	9	FAUX	0	0	0
0	0	8	2	FAUX	0	0	0
0	0	1	0	FAUX	0	0	0
0	0	2	0	FAUX	0	0	0
0	0	0	0	VRAI	0	0	0
0	0	1	0	VRAI	0	0	0
0	0	1	0	VRAI	0	0	0
0	0	0	1	VRAI	0	0	0
0	1	0	1	VRAI	0	0	0
0	0	0	2	VRAI	0	0	0
0	0	0	1000	VRAI	0	0	0
0	0	0	1	FAUX	0	0	0
0	0	2	3	FAUX	0	0	0
0	0	2	5	FAUX	0	0	0
0	0	3	0	FAUX	0	0	0
0	0	1	47	FAUX	0	0	0
0	0	0	1	FAUX	0	0	0
0	0	0	22	FAUX	0	0	0
0	0	1	37	FAUX	0	0	0
0	0	0	23	FAUX	0	0	0
0	0	1	45	FAUX	0	0	0
0	0	0	1	VRAI	0	0	0
0	0	0	1	VRAI	0	0	0
0	1	0	3	FAUX	0	0	0
0	0	2	0	FAUX	0	0	0
0	0	1	1	FAUX	0	0	0
0	0	0	0	VRAI	0	0	0
0	0	0	1	FAUX	0	0	0
0	0	0	0	FAUX	0	0	0
0	0	0	0	FAUX	0	0	0
0	0	0	0	FAUX	0	0	0
0	0	1	3	FAUX	0	0	0
0	0	0	0	FAUX	0	0	0

0	0	0	0	VRAI	0	0	0
0	0	2	0	VRAI	0	5	0
0	1	1	0	VRAI	0	0	0
0	0	0	8	FAUX	0	0	0
0	0	0	0	FAUX	0	0	0
0	0	2	3	VRAI	0	0	0
0	0	0	2	VRAI	0	0	0
0	0	0	1	VRAI	0	0	0
0	0	0	2	VRAI	0	0	0
0	0	0	3	VRAI	0	0	0
0	0	1	5	VRAI	0	0	0
0	0	1	0	FAUX	0	0	0
0	0	1	0	VRAI	0	0	0
0	0	0	1	VRAI	0	0	0
0	0	0	0	FAUX	0	0	0
0	0	0	0	FAUX	0	0	0
0	0	0	0	FAUX	0	2	0
0	0	5	1	FAUX	0	1	0
0	0	1	0	FAUX	0	0	0
2	0	0	2	FAUX	0	0	0
0	0	0	0	FAUX	0	0	0
0	0	0	0	VRAI	0	0	0
0	0	0	0	VRAI	0	0	0
0	0	0	0	FAUX	0	4	0
0	0	4	0	FAUX	0	0	0
0	0	0	1	VRAI	0	0	0
0	0	0	0	VRAI	0	0	0
0	0	0	0	VRAI	0	0	0
0	0	1	0	VRAI	0	0	0
0	0	0	0	VRAI	0	0	0
0	0	0	0	VRAI	0	0	0
0	8	0	0	VRAI	0	0	0
0	0	2	0	VRAI	0	0	0
0	0	3	13	VRAI	0	0	0
0	0	0	0	VRAI	0	0	0
0	0	0	0	VRAI	0	0	0
0	0	2	38	VRAI	0	0	0
0	0	3	13	VRAI	0	0	0
0	0	0	0	VRAI	0	0	0
0	0	0	1	VRAI	0	0	0
0	0	0	1	VRAI	0	0	0
0	0	1	32	VRAI	0	0	0
0	1	0	3	VRAI	0	0	0
0	1	0	0	VRAI	0	0	0
0	0	0	1	VRAI	0	0	0
0	0	0	0	VRAI	0	0	0
0	0	1	7	FAUX	0	0	0
0	0	0	0	FAUX	0	0	0
0	0	1	1	VRAI	0	0	0
0	0	1	2	VRAI	0	0	0
0	0	0	0	VRAI	0	0	0
0	0	0	0	VRAI	0	0	0
0	0	0	0	VRAI	0	0	0
0	0	8	4	VRAI	0	0	0

0	0	0	0	VRAI	0	0	0
0	0	0	0	VRAI	0	0	0
0	0	1	1	VRAI	0	0	0
0	0	0	0	VRAI	0	14	0
0	0	0	0	VRAI	1	0	0
0	0	0	0	VRAI	0	0	0
0	0	0	0	VRAI	0	0	0
0	0	0	0	VRAI	0	0	0
0	0	1	9	VRAI	0	0	0
0	0	0	0	FAUX	0	0	0
0	0	6	5	FAUX	0	0	0
0	0	0	1	FAUX	0	0	0
0	0	8	1	FAUX	0	0	0
0	0	1	70	FAUX	0	0	0
0	0	3	3	FAUX	0	0	0
0	0	0	2	FAUX	0	0	0
0	0	1	0	FAUX	0	0	0
0	1	8	5	FAUX	0	0	0
0	0	8	42	FAUX	0	0	0
0	0	0	0	VRAI	0	0	0
0	0	0	0	VRAI	0	0	0
0	0	0	0	VRAI	0	0	0
0	0	0	0	FAUX	0	7	0
0	0	12	0	FAUX	0	0	0
0	0	0	0	FAUX	0	30	0
0	0	0	1	FAUX	0	0	0
0	0	0	2	FAUX	0	3	0
0	0	0	0	VRAI	0	0	0
0	0	0	0	VRAI	0	0	0
0	0	0	0	VRAI	0	0	0
0	0	0	0	VRAI	0	0	0
0	0	0	0	FAUX	0	0	0

0	0	0	0	FAUX	0	0	0
0	1	1	0	VRAI	0	0	0
0	0	0	0	VRAI	0	0	0
0	0	1	1	VRAI	0	0	0
0	0	0	0	VRAI	0	0	0
0	0	1	0	FAUX	0	0	0
0	1	7	0	FAUX	0	0	0
0	0	1	2	VRAI	0	0	0
0	0	2	0	VRAI	0	0	0
0	0	1	2	VRAI	0	0	0
0	0	5	37	VRAI	0	0	0
0	0	0	1	VRAI	0	0	0
0	0	0	0	VRAI	0	0	0
0	0	1	0	VRAI	0	0	0
0	0	1	0	VRAI	2	0	0
0	0	0	2	VRAI	0	1	0
0	0	2	2	VRAI	0	0	0
0	0	4	3	VRAI	0	0	0
0	0	1	5	FAUX	0	2	0
0	0	1000	0	FAUX	0	0	0

0	0	4	0	FAUX	0	0	0
0	0	12	0	FAUX	0	0	0
0	0	0	41	FAUX	0	0	0
0	0	12	0	FAUX	0	0	0
0	0	0	4	VRAI	0	0	0
0	1000	0	0	VRAI	0	0	0
0	0	0	0	FAUX	0	0	0
0	0	0	0	FAUX	0	0	0
0	0	1	0	FAUX	0	0	0
0	0	0	1	FAUX	0	1	0
0	0	1	3	FAUX	0	0	0
0	0	0	0	FAUX	0	2	0
0	0	0	0	FAUX	0	0	0
1	0	0	0	FAUX	0	0	0
0	0	1	1	FAUX	0	0	0
1	0	5	3	FAUX	0	0	0
0	0	0	0	VRAI	0	0	0
0	0	0	0	VRAI	0	0	0
0	0	0	0	VRAI	0	0	0
0	0	0	0	VRAI	0	0	0
0	0	1	0	FAUX	0	1	0
0	0	1	0	FAUX	0	0	0
0	0	2	0	FAUX	0	3	0
0	0	0	0	FAUX	0	3	0
0	0	0	2	FAUX	0	4	0
0	0	2	0	FAUX	0	0	0
0	0	6	2	FAUX	0	4	0
0	0	3	2	FAUX	0	0	0
0	0	0	1	VRAI	0	0	0
0	0	0	0	VRAI	0	0	0
0	0	1	1	FAUX	2	0	0
0	0	0	2	FAUX	0	2	0
0	0	0	1	VRAI	1	0	0
0	0	0	0	VRAI	0	0	0
0	0	0	1	VRAI	0	0	0
0	0	1	0	VRAI	0	2	0
0	0	11	2	VRAI	0	0	0
0	0	0	2	FAUX	0	0	0
0	0	0	4	FAUX	0	0	0
0	0	0	0	VRAI	0	0	0
0	0	6	5	FAUX	0	0	0
0	0	0	3	FAUX	0	5	0
0	0	0	4	FAUX	0	5	0
0	0	0	1	FAUX	0	0	0
0	1	5	6	VRAI	0	0	0
0	0	5	6	VRAI	0	0	1
0	0	4	1	VRAI	0	0	0
0	0	3	19	VRAI	0	0	0
0	0	0	0	FAUX	0	0	0
0	0	10	0	FAUX	0	0	0
0	0	0	0	FAUX	0	0	0
0	0	1	45	FAUX	0	0	0
0	0	0	1	FAUX	0	0	0
0	0	0	0	FAUX	0	0	0
0	1	3	3	FAUX	0	0	0

0	0	0	0	FAUX	0	0	0
0	0	1	1	FAUX	0	0	0
0	0	1	0	VRAI	0	0	0
0	0	0	0	VRAI	0	3	0
0	0	0	2	VRAI	0	0	0
0	0	12	0	VRAI	0	0	0
0	0	5	1	VRAI	0	0	0
0	0	4	2	VRAI	0	0	0
1	0	1	6	VRAI	0	0	0
0	0	0	1	VRAI	0	0	0
0	0	0	1	VRAI	0	0	0
0	0	0	0	VRAI	0	0	0
0	0	0	73	VRAI	0	0	0
0	0	0	0	VRAI	0	0	0
0	0	0	0	VRAI	0	9	0
0	0	0	1	VRAI	0	0	0
0	0	0	0	VRAI	0	0	0
0	0	3	7	VRAI	0	4	0
0	0	0	0	VRAI	0	0	0
0	0	4	0	VRAI	0	0	0
0	0	0	0	VRAI	0	0	0
0	0	2	2	VRAI	0	0	0
0	0	2	0	VRAI	0	0	0
0	0	0	13	VRAI	0	0	0
0	1	0	1	VRAI	0	0	0
0	0	0	0	VRAI	0	0	0
0	0	1	0	VRAI	0	0	0
0	0	0	1	VRAI	0	0	0
0	0	2	0	VRAI	0	0	0
0	0	0	0	VRAI	0	0	0
0	0	2	5	VRAI	0	0	0
0	0	2	32	VRAI	0	0	0
0	0	1	0	VRAI	0	0	0
0	0	6	2	FAUX	0	0	0
2	0	1	8	FAUX	0	0	0
0	0	1	0	FAUX	0	2	0
0	0	0	0	FAUX	0	0	0
0	0	0	0	FAUX	0	0	0
0	0	0	5	FAUX	0	0	0
0	0	31	12	VRAI	0	0	0
0	0	1	1	FAUX	0	0	0
0	0	1	2	FAUX	0	0	0
0	0	2	2	FAUX	0	0	0
0	1	4	0	FAUX	0	0	0
0	0	0	0	FAUX	0	4	0
0	0	0	1	FAUX	0	0	0
0	1	11	2	FAUX	0	0	0
0	1	0	0	FAUX	0	0	0
0	0	1	0	VRAI	0	0	0
0	0	2	0	FAUX	0	0	0
0	0	2	58	FAUX	0	0	0
0	0	1	0	FAUX	0	0	0

asp_ustus_fetv	asp_niger_fetv	asp_sp_fetv	asp_terreus_fetv	asp_flavus_fetv	peni_fetv	clado_fetv
0	0	0	0	0	0	0
0	0	0	0	0	0	0
0	0	0	0	0	0	0
0	0	0	0	0	0	0
0	0	0	0	0	0	0
0	0	0	0	0	25	0
0	1	0	0	0	5	0
0	0	0	0	0	2	0
0	0	0	0	0	0	0
0	1	5	0	0	5	0
0	0	0	0	0	0	1
0	0	0	0	0	0	5
0	0	0	0	0	0	1
0	0	0	0	0	0	2
0	0	0	0	0	0	0
0	0	0	0	0	2	0
0	0	0	0	0	0	0
0	0	0	0	0	0	0
0	0	1	0	0	0	0
0	0	0	0	0	5	0
1	0	1	0	0	9	0
0	0	0	0	0	0	0
0	0	0	0	0	3	1
1	0	5	0	0	6	1
2	0	0	0	0	6	0
0	0	0	0	0	0	0
0	0	0	0	0	0	0
0	0	0	0	0	0	0
0	0	0	0	0	0	0
0	0	0	0	0	0	0
0	0	0	0	0	0	0
0	0	0	0	0	1	0
0	0	0	0	0	0	0
0	0	0	0	0	0	0
1	0	0	0	0	0	0
0	0	0	0	0	18	0
0	0	0	0	0	0	0
0	0	0	0	0	0	0
4	0	0	0	0	6	0
0	0	0	0	0	0	0
0	0	0	0	0	0	0
0	0	0	0	0	0	0
0	0	0	0	0	2	0
0	0	0	0	0	1	0
0	0	0	0	0	0	0
0	0	0	0	0	0	0
0	0	0	0	0	0	0
0	0	0	0	0	1	0
0	0	0	0	0	0	0
0	0	0	0	0	0	0
0	0	0	0	0	0	0
0	0	0	0	0	0	0
0	0	0	0	0	0	0
1	0	0	0	0	100	0
0	0	0	0	0	0	0

0	0	0	0	0	3	0
0	0	0	0	0	0	0
0	0	0	0	0	0	0
0	0	0	0	0	0	0
0	0	0	0	0	0	0
0	0	0	0	0	2	0
0	0	0	0	0	0	0
0	11	0	0	0	0	0
0	0	0	0	0	0	0
0	0	0	0	0	0	0
0	7	0	0	0	0	1
0	0	0	0	0	0	0
0	0	1	0	0	0	0
0	0	0	0	0	1	0
0	0	1	0	0	0	0
0	0	0	0	0	0	0
0	0	0	0	0	0	0
0	0	0	0	0	0	0
0	0	0	0	0	0	0
0	0	0	0	0	0	0
0	0	0	0	0	0	0
0	0	3	0	0	2	0
0	0	0	0	0	0	0
1	2	0	0	0	4	0
0	0	0	0	0	1	0
0	0	3	0	0	0	1
0	0	0	0	0	0	0
0	0	0	0	0	0	0
0	0	0	0	0	0	0
0	0	0	0	0	0	0
0	0	0	0	0	0	0
0	0	0	0	0	0	0
0	0	0	0	0	0	0
0	0	0	0	0	0	0
0	0	3	0	0	0	0
0	0	0	0	0	2	0
0	0	0	0	0	0	0
0	0	0	0	0	0	0
0	1	0	0	0	0	0
0	0	0	0	0	0	0
0	0	0	0	0	0	0
0	2	0	0	0	0	0
0	0	0	0	0	0	0
0	0	0	0	0	0	0
0	0	0	0	0	0	0
0	0	0	0	0	0	0
0	0	0	0	0	0	0
0	0	0	0	0	0	0
0	0	0	0	0	0	0
0	0	0	0	0	0	0
0	0	0	0	0	4	0
0	0	0	0	1	1	1
0	0	0	0	0	0	0
0	0	0	0	0	1	0
0	0	0	0	0	0	0

0	0	0	0	0	0	0
0	0	0	0	0	0	0
0	0	0	0	0	0	0
0	0	0	0	0	0	0
0	0	0	0	0	0	0
0	0	0	0	0	0	0
0	0	0	0	0	0	0
0	0	0	0	0	0	0
0	0	0	0	0	0	0
0	0	0	0	0	0	0
0	0	0	0	0	0	0
0	0	0	0	0	0	0
0	0	0	0	0	0	0
0	0	0	0	0	0	0
0	0	0	0	0	0	0
0	0	0	0	0	0	0
0	0	0	0	0	0	0
0	0	0	0	0	0	0
0	0	0	0	0	0	0
0	0	0	0	0	0	0
0	0	1	0	0	0	0
0	0	0	0	0	0	0
0	0	0	0	0	0	0
0	0	0	0	0	0	0
2	0	0	0	0	0	1
0	0	0	0	0	0	0
0	0	0	0	0	0	0
0	0	0	0	0	0	0
0	0	1	0	0	9	0
0	0	0	0	0	0	0
0	0	0	0	0	0	0
0	0	0	0	0	1	0
0	0	0	0	0	1	2
0	0	0	0	0	1	0
0	0	0	0	0	2	0
0	0	0	0	0	0	0
0	0	0	0	0	1	0
0	0	0	0	0	0	0
0	0	0	0	0	0	0
0	0	0	0	0	0	0
0	0	0	0	0	0	0
0	0	0	0	0	0	0
0	0	0	0	0	0	0
0	0	1	0	0	0	0
0	1	0	0	0	1	0
2	0	0	0	0	2	0
0	0	0	0	0	0	0
0	0	0	0	0	0	0
0	0	0	0	0	0	0
0	0	0	0	0	8	0
0	0	0	0	0	0	0
1	0	0	0	0	0	1
0	0	0	0	0	1	0
0	0	0	0	0	0	0

0	0	0	0	0	1	0
0	0	0	0	0	0	0
0	0	0	0	0	0	0
0	0	0	0	0	0	0
0	0	0	0	0	0	0
0	0	0	0	0	0	0
0	0	0	0	1	0	0
0	0	0	0	0	0	0
0	0	0	0	0	0	0
0	0	0	0	0	0	0
0	0	0	0	0	1	0
0	0	0	0	0	0	0
0	0	0	0	0	0	0
0	0	0	0	0	0	0
0	0	0	0	0	0	0
0	0	0	0	0	0	0
0	0	0	0	0	0	0
0	0	0	0	0	0	0
0	0	0	0	0	0	0
0	0	0	0	0	0	2
0	2	0	0	0	0	0
0	0	0	0	0	0	1
0	0	0	0	0	0	0
0	0	0	0	0	0	0
0	0	0	0	0	0	0
0	0	0	0	0	3	0
0	0	0	0	0	16	0
0	0	0	0	0	3	0
0	0	0	0	0	0	0
0	0	0	0	0	0	0
0	0	0	0	0	1	0
0	0	0	0	0	0	0
0	0	0	0	0	0	0
0	0	0	0	0	0	0
0	0	0	0	0	0	0
0	0	0	0	0	0	0
0	0	0	0	0	0	0
0	0	0	0	0	0	0
0	0	0	0	0	0	0
0	0	0	0	0	0	0
0	0	0	0	0	0	0
0	0	0	0	0	0	0
0	0	0	0	0	0	0
0	0	0	0	0	0	1
0	0	0	0	0	0	0
0	0	1	0	0	0	0
0	0	0	0	0	0	0
0	0	0	0	0	2	0
0	0	0	0	0	0	0
0	0	1	0	0	4	2
0	0	0	0	0	0	0
0	0	0	0	0	0	0

0	0	0	0	0	1	0
0	0	0	0	0	8	0
0	0	0	0	0	0	0
3	0	0	0	0	31	0
0	0	4	0	0	11	0
0	0	0	0	0	4	0
0	0	0	0	0	0	0
0	0	0	0	0	0	0
0	0	0	0	0	0	0
0	0	0	0	0	1	0
0	0	0	0	0	0	0
0	0	0	0	0	11	0
0	0	0	0	0	0	0
0	0	0	0	0	0	0
0	0	0	0	0	0	0
0	0	0	0	0	0	0
0	0	0	0	0	3	2
0	1	0	0	0	4	0
2	1	0	0	0	2	1
0	0	0	0	0	1	1
0	1	0	0	0	1	0
0	0	0	0	0	0	0
0	0	0	0	0	0	0
0	4	0	0	0	17	0
0	1	0	0	0	7	0
0	0	0	0	0	0	0
0	0	0	0	0	1	0
0	0	0	0	0	0	0
0	0	0	0	0	0	0
0	0	0	0	0	0	0
0	0	0	0	0	0	0
0	0	0	0	0	0	0
0	0	0	0	0	0	0
0	0	0	0	0	0	0
0	0	0	0	0	3	0
0	0	0	0	0	13	0
0	0	0	0	0	0	0
1	0	0	0	0	0	0
0	0	0	0	0	0	0
0	0	0	0	0	1	0
0	0	0	0	0	0	0
0	0	0	0	0	0	0
0	0	0	0	0	0	0
0	0	0	0	0	0	0
0	0	0	0	0	1	0
0	0	0	0	0	0	0
0	0	0	0	0	1	0
0	0	15	0	0	1	0
0	0	0	0	0	0	0
0	0	0	0	0	0	0
0	0	0	0	0	0	0
0	0	0	0	0	0	0
0	0	0	0	0	0	0
0	0	0	0	0	0	0

0	0	0	0	3	0	0
0	0	0	0	0	1	0
0	0	0	0	0	0	0
0	0	0	0	0	0	0
0	0	0	0	0	0	0
0	0	0	0	0	0	0
0	0	0	0	0	0	0
0	0	0	0	0	0	0
0	0	0	0	0	0	0
0	0	0	0	0	2	0
0	0	0	0	0	3	0
0	0	0	0	0	0	0
1	0	1	0	0	0	0
0	0	0	0	0	0	0
0	0	0	0	0	0	0
0	0	0	0	0	0	0
0	0	0	0	0	0	0
0	0	0	0	0	0	0
0	0	0	0	0	13	0
0	0	0	0	0	0	0
0	0	0	0	0	0	0
0	0	0	0	0	0	0
0	0	0	0	0	0	0
0	0	0	0	0	0	0
0	0	0	0	0	1	0
2	0	0	0	0	0	0
0	0	0	0	0	0	0
0	0	0	0	0	0	0
0	0	0	0	0	0	0
0	0	0	0	0	0	0
0	0	0	0	0	0	0
0	0	0	0	0	0	0

0	0	0	0	0	0	0
0	0	0	0	0	0	0
0	0	0	0	0	0	0
0	0	0	0	0	0	0
0	0	0	0	0	1	0
0	1	0	0	0	0	0
0	0	0	0	0	7	0
0	0	0	0	0	0	0
0	0	0	0	0	0	0
0	0	0	0	0	0	0
0	0	0	0	0	0	0
0	0	0	0	0	3	0
0	0	0	0	0	1	0
0	0	4	0	0	0	0
0	0	0	0	0	30	0
0	0	0	0	0	0	0
0	0	0	0	0	0	0
0	0	0	0	0	0	0
0	0	0	0	0	0	0
0	0	0	0	0	5	0

1	0	6	0	0	10	0
0	0	1	0	0	10	0
0	0	0	0	0	4	0
0	0	2	0	0	6	0
0	0	0	0	0	7	0
0	0	0	0	0	18	0
0	0	0	0	0	1	0
0	0	0	0	0	0	0
0	0	0	0	0	0	0
0	1	0	0	0	4	0
0	1	0	0	0	34	0
0	0	0	0	0	1	1
3	0	0	0	2	0	0
0	0	0	0	0	1	0
0	0	0	0	0	0	0
0	0	0	0	0	0	0
0	0	0	0	0	57	0
0	0	0	0	0	0	0
0	0	0	0	0	0	0
0	0	0	0	0	0	0
0	1	0	0	0	1	0
0	0	0	0	0	2	0
0	0	0	0	0	6	0
0	0	0	0	0	1	0
0	0	0	0	0	1	0
0	0	0	0	0	1	0
0	1	0	0	0	1	0
0	0	0	0	0	1	0

0	0	0	0	0	0	0
0	0	0	0	0	0	0
0	0	0	0	0	2	0
0	0	0	0	0	1	0
0	0	0	0	0	0	1
0	0	0	0	0	1	0
0	0	0	0	0	0	0
0	0	0	0	0	3	1
0	0	0	0	0	0	0
0	0	0	0	0	0	0
0	0	0	0	0	1	0
0	0	0	0	0	1	0
0	0	0	0	0	1	0
0	0	0	0	0	4	0
0	0	0	0	0	24	2
1	6	0	0	0	1000	0
0	0	0	0	0	1	2
0	0	0	0	0	17	0
0	0	0	0	0	0	0
0	0	0	0	0	0	1
0	0	0	0	0	0	0
0	0	0	0	0	1	1
0	0	0	0	0	0	0
0	0	0	0	0	0	0
0	0	0	0	0	0	0
0	0	0	0	0	31	2
0	0	0	0	0	0	0

0	0	0	0	0	0	0
0	0	0	0	0	0	0
0	0	3	0	0	1	1
0	0	3	0	0	0	0
0	0	1	0	0	3	0
0	0	0	0	0	0	0
0	0	0	0	0	0	0
0	0	0	0	0	0	0
0	0	0	0	0	0	0
0	0	0	0	0	0	0
0	0	1	0	0	0	0
0	0	0	0	0	0	0
0	0	23	0	0	0	0
0	0	0	0	0	0	1
0	0	0	0	0	0	0
0	0	0	0	0	0	0
0	0	0	0	0	0	0
0	0	0	0	0	0	0
0	0	0	0	0	0	0
0	0	0	0	0	0	0
0	0	0	0	0	0	0
0	0	0	0	0	0	0
0	0	12	0	0	0	0
0	0	0	0	0	1	1
0	0	0	0	0	9	0
0	0	0	0	0	1000	0
0	0	0	0	0	1000	0
0	0	0	0	0	1	0
0	0	0	0	0	0	0
0	0	0	0	0	0	0
0	0	0	0	0	0	0
0	0	0	0	0	0	0
0	0	0	0	0	1	0
0	0	0	0	0	0	0
0	0	0	0	0	0	0
0	0	0	0	0	0	1
2	0	0	0	0	0	0
0	0	0	0	0	0	0
0	0	0	0	0	0	0
0	0	0	0	0	1	0
0	0	0	0	0	2	0
0	0	0	0	0	0	1
0	0	0	0	0	10	1
0	0	0	0	0	0	0
0	0	0	0	0	1	0
0	0	0	0	0	0	0
0	0	0	0	0	0	0
0	0	0	0	0	0	0
1	0	0	0	0	3	0
0	0	0	0	0	0	0
0	0	0	0	0	1	2
0	0	0	0	0	0	0

alterna_fetv	ulocla_fetv	chaeto_fetv	scopula_fetv	acremo_fetv	fusa_fetv	rhizo_fetv	mucor_fetv
0	0	0	0	0	0	0	0
0	0	0	0	0	0	0	0
0	0	0	0	0	0	0	0
0	0	0	0	0	0	0	0
0	0	0	0	0	0	0	0
3	0	0	0	1	0	1000	0
0	0	0	0	1	0	0	0
2	0	0	0	0	0	0	0
0	0	0	0	0	0	0	0
0	0	0	0	0	0	0	0
0	0	0	0	0	0	0	0
0	0	0	0	0	0	0	0
0	0	0	0	0	0	0	0
1	0	0	0	0	0	0	0
0	0	0	0	0	0	0	0
1	0	0	0	0	0	0	0
0	0	0	0	0	0	0	0
0	0	0	0	0	0	0	0
2	0	0	0	0	0	0	0
0	0	0	0	0	0	1000	0
0	1	0	0	0	0	0	0
0	0	0	0	0	0	0	0
1	1	0	0	0	0	0	0
1	0	0	0	0	0	0	0
2	0	0	0	0	0	0	0
0	0	0	0	0	0	0	0
0	0	0	0	0	0	0	0
0	0	0	0	0	0	0	0
0	0	0	0	0	0	0	0
0	0	0	0	0	0	0	0
0	0	0	0	0	0	0	0
0	0	0	0	0	0	0	0
0	0	0	0	0	0	0	0
0	0	0	0	0	0	0	0
0	0	0	0	0	0	0	0
0	0	0	0	0	0	0	0
0	0	0	0	0	0	0	0
0	0	0	0	0	0	0	0
0	0	0	0	0	0	3	0
0	0	0	0	0	0	0	0
0	0	0	0	0	0	1000	0
0	0	0	0	0	0	0	0
1	0	0	0	0	0	0	0
1	0	0	0	0	0	0	0
0	0	0	0	0	0	0	0
0	0	0	0	0	0	0	0
0	0	0	0	0	0	0	0
0	0	0	0	0	0	0	0
0	0	0	0	0	0	0	0
0	0	0	0	0	0	0	0
0	0	0	0	0	0	0	0
1	0	0	0	0	0	0	0
0	0	0	0	0	0	0	0
0	0	0	0	0	0	0	4
1	0	0	0	0	0	0	0

0	0	0	0	0	0	0	0
0	0	0	0	0	0	0	0
0	0	0	0	0	0	0	0
0	0	0	0	0	0	0	0
0	0	0	0	0	0	0	0
0	0	0	0	0	0	0	0
0	0	0	0	0	0	0	0
2	0	0	0	0	0	0	0
0	0	1	0	0	0	0	0
0	0	2	0	0	0	0	0
1	0	0	0	0	0	0	0
0	0	1	0	0	0	0	0
1	0	0	0	0	0	0	0
0	0	0	0	0	0	0	0
0	0	1	0	0	0	0	0
0	0	0	0	0	0	0	0
0	0	0	0	0	0	0	0
0	0	0	0	0	0	0	0
0	0	0	0	0	0	0	0
0	0	0	0	0	0	0	0
0	0	0	0	0	0	0	0
2	0	0	0	0	0	0	0
0	0	0	0	0	0	0	0
0	1	0	0	0	0	0	1
3	0	1	0	0	0	0	0
1	0	0	0	0	0	0	0
0	0	0	0	0	0	0	0
0	0	0	0	0	0	0	0
0	0	0	0	0	0	0	0
0	0	0	0	0	0	0	0
0	0	0	0	0	0	0	0
0	0	0	0	0	0	0	0
1	0	0	0	0	0	0	0
0	0	0	0	0	0	0	0
0	0	0	0	0	0	0	0
0	0	0	0	0	0	0	0

0	0	0	7	0	0	0	0
0	0	0	0	0	0	0	0
0	0	0	0	0	0	0	0
0	0	0	0	0	0	0	0
0	0	0	0	0	0	0	0
0	0	1000	0	0	0	1	0
0	0	0	0	0	0	0	0
0	0	0	0	0	0	0	0
0	0	0	0	0	0	0	0
0	0	0	0	0	0	0	0
0	0	0	0	0	0	0	0
0	0	0	0	0	0	0	0
0	0	0	0	0	0	0	0
0	0	0	0	0	0	0	0
0	0	0	0	0	0	0	0
0	0	0	1	0	0	0	0
0	0	0	0	0	0	0	0
0	0	0	0	0	0	0	0
0	0	0	0	0	0	0	0

0	0	0	0	0	0	0	0
0	0	0	0	0	0	0	0
0	0	0	0	0	0	0	0
0	0	0	0	0	0	0	0
0	0	0	0	0	0	0	0
0	0	0	0	0	0	0	0
0	0	0	0	0	0	0	0
0	0	0	0	0	0	0	0
0	0	0	0	0	0	0	0
0	0	0	0	0	0	0	0
0	0	0	0	0	0	0	0
0	0	0	0	0	0	0	0
0	0	0	0	0	0	0	0
0	0	0	0	0	0	0	0
0	7	0	0	0	0	0	0
0	0	0	0	0	0	0	0
1	0	0	0	0	0	0	0
0	0	0	0	0	0	0	0
1	0	0	0	0	0	0	0
0	0	0	0	0	0	0	0
0	0	0	0	0	0	0	0
0	0	0	0	0	0	0	0
0	0	0	0	0	0	0	0
0	0	0	0	0	0	0	0
0	0	0	0	0	0	0	0
0	0	0	0	0	0	0	0
0	0	0	0	0	0	0	0
0	0	0	0	0	0	0	0
0	0	0	0	0	0	0	0
0	0	0	0	0	0	0	0
2	0	0	8	0	0	0	0
0	0	0	0	0	0	0	0
0	0	0	0	0	0	0	0
1	0	0	0	0	0	0	0
1	0	0	0	0	0	0	0
0	0	0	0	0	0	0	0
0	0	0	1	0	0	0	0
0	0	0	0	0	0	0	0
0	0	0	0	0	0	0	0
0	0	0	0	0	0	0	0
0	0	0	0	0	0	0	0
0	0	0	0	0	0	0	0
0	0	0	0	0	0	0	0
0	0	0	0	0	0	0	0
0	0	0	0	0	0	0	0
1	1	5	0	0	0	0	0
0	0	0	0	0	0	0	0
0	0	0	0	0	0	0	0
0	0	0	0	0	0	0	0
0	0	0	0	0	0	0	0
0	0	0	0	0	0	0	0
0	0	0	0	0	0	0	0
0	0	1	0	0	0	0	0
0	0	0	0	0	0	0	0

2	0	0	0	0	0	0	0
1	0	0	0	0	0	0	0
0	0	0	0	0	0	0	0
0	0	0	0	0	0	0	0
0	0	0	0	0	0	0	0
0	0	0	0	0	0	0	0
0	0	0	0	0	0	0	0
0	0	0	0	0	0	0	0
0	0	0	0	0	0	0	0
0	0	0	0	0	0	0	0
0	0	0	0	0	0	0	0
0	0	0	0	0	0	0	0
0	0	0	0	0	0	0	0
0	0	0	0	0	0	0	0

0	0	0	0	0	0	0	0
1	0	0	0	0	0	0	0
0	0	0	0	0	0	0	0
0	0	0	0	0	0	0	0
0	0	0	0	0	0	1	0
0	0	0	0	0	0	0	0
0	0	0	0	0	0	0	0
0	0	0	0	0	0	0	0
0	0	0	0	0	0	0	0

0	0	0	0	0	0	0	0
0	0	0	0	0	0	0	0
0	0	0	0	0	0	0	0
1	0	0	0	0	0	0	0
0	0	0	0	0	0	0	0
0	0	0	0	0	0	0	0
0	0	0	0	0	0	0	0
0	0	0	0	0	0	0	0
0	0	0	0	0	0	0	0
0	0	0	0	0	0	0	0
0	0	0	0	0	0	0	0
0	0	0	0	0	0	0	0
0	0	0	0	0	0	0	0
0	0	0	0	0	0	0	0
0	0	0	0	0	0	0	0
0	0	0	0	0	0	0	0
0	0	0	0	0	0	0	0
0	0	0	0	0	0	0	0
0	0	0	0	0	0	0	0
1	1	0	0	1	0	0	0
0	0	0	0	0	0	0	0
0	0	0	0	0	0	0	1
0	0	0	1	0	0	0	0
0	0	0	0	0	0	0	0
0	0	0	0	0	0	0	1
0	0	0	0	0	0	0	0
0	2	1	0	0	0	0	0
0	0	0	0	0	0	0	0
0	0	0	0	0	0	0	0

0	0	0	0	0	0	0	0
0	4	1000	0	0	0	0	0
0	0	0	0	0	0	0	0
2	1	0	0	0	0	0	0
3	0	0	0	0	0	0	0
0	0	0	0	0	0	0	0
0	0	0	0	0	0	0	0
0	0	0	0	0	0	0	0
0	0	0	0	0	0	0	0
0	0	0	0	0	0	0	0
0	0	0	0	0	0	0	0
1	0	0	0	0	0	0	0
0	0	0	0	0	0	0	0
0	0	0	0	0	0	0	0
0	0	0	0	0	0	0	0
0	0	0	0	0	0	0	0
1	0	0	0	0	0	0	0
4	0	0	0	0	0	0	1
4	0	0	0	0	0	0	0
1	0	0	0	0	0	0	0
1	0	0	0	0	0	0	0
0	0	0	0	0	0	0	0
0	0	0	0	0	0	0	0
0	0	0	0	0	0	0	0
0	0	0	0	0	0	0	0
0	0	0	0	0	0	0	0
0	0	0	0	0	0	0	0
0	0	0	0	0	0	0	0
0	0	0	0	0	0	0	0
0	0	0	0	0	0	0	0
0	0	0	0	0	0	0	0
0	0	0	0	0	0	0	0
0	0	0	0	0	0	0	0
0	0	0	0	0	0	0	0
0	0	0	0	0	0	0	0
0	0	0	0	0	0	0	0
0	0	0	0	0	0	0	0
1	0	0	0	0	0	0	0
0	0	0	0	0	0	0	0
0	0	0	0	0	0	0	0
0	0	0	0	0	0	0	0
0	0	0	0	0	0	0	0
0	0	0	0	0	0	0	0
2	0	0	0	0	0	0	0
0	0	0	0	0	0	0	0
0	0	0	0	0	0	0	0
2	0	0	0	0	0	0	0
0	0	0	0	0	0	0	0
0	0	0	0	0	0	0	0
0	0	0	0	0	0	0	0
0	0	0	0	0	0	0	0
0	0	0	0	0	0	0	0
0	0	0	0	0	0	0	0
0	0	0	0	0	0	0	0

0	0	0	0	0	0	0	0
0	0	0	0	0	0	0	0
0	0	0	0	0	0	0	0
0	0	0	0	1	0	0	0
0	0	0	0	0	0	0	1
0	0	0	0	0	0	0	0
0	0	0	0	0	0	0	0
0	0	0	0	0	0	0	0
0	0	0	0	0	0	0	0
0	1	0	0	0	0	0	0
0	0	0	0	0	0	0	0
0	0	0	0	0	0	0	0
0	0	0	0	0	0	0	0
0	0	0	0	0	0	0	0
0	0	0	0	0	0	0	0
0	0	0	0	0	0	0	0
0	0	0	0	0	0	0	0
0	0	0	0	0	0	0	0
0	0	0	0	0	0	0	0
0	0	0	0	0	0	0	0
0	0	0	0	0	0	0	0
0	0	0	0	0	0	0	0
0	0	0	0	0	0	0	0
0	0	0	0	0	0	0	0
0	0	0	0	0	0	0	0
0	0	0	0	0	0	0	0
0	0	0	0	0	0	0	0
0	0	0	0	0	0	0	0
0	0	0	0	0	0	0	0
0	0	0	0	0	0	0	0
0	0	0	0	0	0	0	0
0	0	0	0	0	0	0	0

0	0	0	0	0	0	0	0
0	0	0	0	0	0	0	0
0	0	0	0	0	0	0	0
0	0	0	0	0	0	0	0
0	0	0	0	0	0	0	0
1	0	0	0	0	0	0	0
0	0	0	0	0	0	0	0
0	0	0	0	0	0	0	0
0	0	0	0	0	0	0	0
0	0	0	0	0	0	0	0
0	0	0	0	0	0	0	0
0	0	0	0	0	0	0	0
1	0	0	0	0	0	0	0
0	0	0	0	0	0	0	0
1	0	0	0	0	0	0	0
0	0	0	0	0	0	0	0
1	0	0	0	0	0	0	0
0	0	0	0	0	0	0	0
0	0	0	0	0	0	0	0
0	0	0	0	0	0	0	0

0	0	0	0	0	0	0	0
1	0	0	0	0	0	0	0
0	0	0	0	0	0	0	0
0	0	7	0	0	0	0	0
0	0	0	0	0	0	0	0
0	0	0	0	0	0	0	0
0	0	0	0	0	0	0	0
0	0	0	0	0	0	0	0
0	0	0	0	0	0	0	0
0	0	0	1	0	0	0	0
0	1	0	0	0	0	0	0
0	1	0	0	0	0	0	0
0	1	0	0	0	0	0	0
0	0	0	0	0	0	0	0
0	0	0	0	0	0	0	0
0	0	0	0	0	0	0	0
1	0	0	0	0	0	0	0
0	0	0	0	0	0	0	0
0	0	0	0	0	0	0	0
0	0	0	0	0	0	0	0
0	0	0	0	0	0	0	0
3	0	0	0	0	0	0	0
0	0	0	0	0	0	0	0
0	0	0	0	0	0	0	0
0	0	0	0	0	0	0	0
0	0	0	0	0	0	0	0
0	0	0	0	0	0	0	0
0	0	0	0	0	0	0	0
0	0	0	0	0	0	0	0
0	0	0	0	0	0	0	0
0	0	0	0	0	0	0	0
0	0	0	0	0	0	0	0
0	0	0	0	0	0	0	0
0	0	0	0	0	0	0	0
0	0	0	0	0	0	0	0
0	0	0	0	0	0	0	0
0	0	0	0	0	0	0	0
0	0	82	0	0	0	0	0
3	0	0	0	0	0	0	0
0	0	0	0	0	0	0	0
0	0	0	0	0	0	0	10
0	0	0	0	0	0	0	0
0	0	1000	0	0	0	0	0
1	0	0	0	0	0	1	0
1	0	0	0	0	0	0	0
1	0	0	0	0	0	0	0
0	0	0	0	0	0	0	0
0	0	0	0	0	0	0	0
0	0	0	0	0	0	0	0
0	0	0	0	0	0	0	3
0	0	0	0	0	0	0	0
0	0	0	0	0	0	0	0
0	0	1	0	0	0	0	0
0	1	0	0	0	0	0	0
0	0	0	0	0	0	0	0

0	0	0	0	0	0	0	0
0	0	0	0	0	0	0	0
0	0	0	0	0	0	0	0
0	0	0	0	0	0	0	0
0	0	0	0	0	0	0	0
0	0	0	0	0	0	0	0
0	0	0	0	0	0	0	0
0	0	0	0	0	0	0	0
0	0	0	0	0	0	0	0
0	0	0	0	0	0	0	0
0	0	0	0	1	0	0	0
0	0	0	0	0	0	0	0
0	0	0	0	0	0	0	0
1	0	0	0	0	0	0	0
0	0	0	0	0	0	0	0
0	0	0	0	0	0	0	0
0	0	0	0	0	0	0	0
0	0	0	0	0	0	0	0
0	0	0	0	0	0	0	0
0	0	0	0	0	0	0	0
0	0	0	0	0	0	0	0
0	0	0	0	0	0	0	0
0	0	0	0	0	0	0	0
0	0	0	0	0	0	0	0
1	0	0	0	0	0	0	0
0	0	0	0	0	0	0	0
0	0	0	0	0	0	0	0
0	0	0	0	0	0	0	0
0	0	0	0	0	0	0	0
0	0	0	0	0	0	0	0
0	0	0	0	0	0	0	0
0	0	0	0	0	0	0	0
0	0	0	0	0	0	0	0
0	0	0	0	0	0	0	0
0	0	0	0	0	0	0	0
0	0	0	0	0	0	0	0
0	0	0	0	0	0	0	0
0	0	0	0	0	0	0	0
0	0	0	0	0	0	0	0
0	0	0	0	0	0	0	0
0	0	0	0	0	0	0	0
0	0	0	0	0	0	0	0
0	0	0	0	0	0	0	0
0	0	0	0	0	0	0	0
3	0	0	0	0	0	0	0
0	0	0	0	0	0	0	0
0	0	0	0	0	0	0	0
0	0	0	0	0	0	0	0
1	0	0	0	0	0	0	0
1	0	0	0	0	0	0	0
1	0	0	0	0	0	0	0
0	0	0	0	0	0	0	0

geotri_fetv	trichoderm_fetv	paecilo_fetv	autres_fetv	csd_fetv	levure_fetv
0	0	0	0	0	0
0	0	0	2	1	0
0	0	0	0	0	0
0	0	0	0	0	0
0	0	0	0	0	0
0	0	0	0	3	0
0	0	0	0	8	9
0	0	0	0	5	1
0	0	0	0	0	0
0	0	0	0	5	10
0	2	0	0	2	1
0	0	0	0	4	0
0	0	0	0	7	4
0	0	0	2	1	3
0	0	0	0	0	0
0	0	5	0	2	1
0	0	0	0	0	0
0	0	0	0	0	0
0	0	0	40	4	25
0	0	0	0	0	0
0	0	0	0	4	3
0	0	0	0	0	0
0	0	0	0	7	1
0	0	0	0	3	2
0	0	0	0	3	0
0	0	0	0	0	0
0	0	0	0	0	0
0	0	0	0	0	0
0	0	0	0	0	0
0	0	0	0	0	0
0	0	0	0	5	2
0	0	0	0	0	0
0	0	0	0	1	0
0	0	0	0	2	0
0	0	0	0	0	0
0	0	3	0	0	0
0	0	1	0	0	0
0	0	0	0	0	0
0	0	0	9	0	4
0	0	0	0	0	0
0	0	0	23	0	6
0	0	0	0	0	0
0	0	0	0	1000	1
0	0	0	0	29	0
0	0	0	0	0	0
0	0	0	0	0	0
0	0	0	0	0	0
0	0	0	0	1	0
0	0	0	0	0	0
0	0	0	0	0	0
0	0	0	0	0	1
0	0	0	0	0	0
0	0	0	0	3	0
0	0	0	0	1	0
0	0	0	0	0	0

0	0	0	0	0	0
0	0	0	0	0	0
0	0	0	0	0	0
0	0	0	0	0	0
0	0	0	0	0	0
0	0	0	0	9	4
0	0	0	2	11	3
0	0	0	0	15	1
0	0	0	0	1	0
0	0	0	0	1	0
0	0	0	0	1	1
0	0	0	0	1	0
0	0	2	1	0	1
0	0	0	0	0	0
0	0	0	0	1	3
0	0	0	0	0	1
0	0	0	0	1	0
0	0	0	0	0	0
0	0	0	0	0	0
0	0	0	0	0	0
0	0	0	0	0	0
0	0	0	0	7	2
0	0	0	0	0	0
0	0	0	0	6	14
0	0	0	0	1	2
0	0	0	0	3	0
0	0	0	0	0	0
0	0	0	0	0	0
0	0	0	0	0	0
0	0	0	0	0	0
0	0	0	0	0	0
0	0	0	0	0	0
0	0	0	0	0	0
0	0	0	0	0	0
0	0	0	0	3	0
0	0	0	0	4	2
0	0	0	0	1	0
0	0	0	0	0	0
0	0	0	0	1000	0
0	0	0	0	1000	0
0	0	0	0	3	2
0	0	0	0	0	0
0	0	0	0	9	0
0	0	0	0	0	0
0	0	0	0	0	0
0	0	0	0	0	0
0	0	0	0	1	0
0	0	0	0	0	0
0	0	0	0	0	0
0	0	0	0	0	0
0	0	0	0	1	0
0	0	0	0	0	0
0	0	0	0	0	0
0	0	0	0	0	8
0	0	0	0	0	0

0	0	0	0	0	1
0	0	0	0	0	0
0	0	0	0	0	0
0	0	0	0	0	0
0	0	0	0	0	0
0	0	0	0	0	0
0	0	0	0	0	2
0	0	0	0	0	0
0	0	0	0	1	0
0	0	0	0	0	3
0	0	0	0	0	0
0	0	0	0	0	0
0	0	0	0	0	0
0	0	0	0	0	0
0	0	0	0	10	0
0	0	0	0	1	0
0	0	1000	0	0	0
0	0	0	0	1	0
0	0	31	0	4	2
0	0	0	0	1	0
0	0	1	0	1	0
0	0	0	0	0	0
0	0	0	0	0	0
0	0	0	0	0	0
0	0	0	0	3	2
0	0	0	0	0	0
0	0	0	0	1	0
0	0	0	0	0	0
0	0	0	1	3	0
0	0	0	0	0	0
0	0	0	0	0	0
0	0	0	1	40	0
0	0	0	0	43	0
0	0	0	0	17	0
0	0	0	0	37	0
0	0	0	0	2	0
0	0	0	0	0	0
0	0	0	0	0	0
0	0	0	0	0	0
0	0	0	0	0	0
0	0	0	0	0	0
0	0	0	0	0	0
0	0	0	0	0	0
0	0	0	0	3	0
0	0	0	0	2	12
0	0	0	0	6	2
0	0	0	0	0	0
0	0	0	0	0	0
0	0	0	0	0	0
0	0	0	0	0	0
0	0	0	0	0	0
0	0	0	0	1	0
0	0	0	0	0	2
0	0	0	0	0	0

0	0	0	0	0	1
0	0	0	0	2	0
0	0	0	0	0	0
0	0	0	0	0	0
0	0	0	0	0	0
0	0	0	0	0	0
0	0	0	0	0	0
0	0	0	0	0	0
0	0	0	0	7	1
0	0	0	0	0	0
0	0	0	0	10	3
0	0	0	0	0	0
0	0	0	0	0	0
0	0	0	0	14	0
0	0	0	0	0	0
0	0	0	0	0	2
0	0	0	0	0	0
0	0	0	0	1	1
0	0	0	0	1000	0
0	0	0	0	0	0
0	0	0	0	0	0
0	0	0	0	1	0
0	0	0	0	0	1
0	0	2	0	1	1
0	0	0	0	0	0
0	0	0	0	1	0
0	0	0	0	3	0
0	0	0	0	3	0
0	0	0	0	0	0
0	0	0	0	0	0
0	0	0	0	3	8
0	0	0	0	0	0
0	0	0	0	0	0
0	0	0	0	0	0
0	0	0	0	0	0
0	0	0	0	0	0
0	0	0	0	0	0
0	0	0	0	0	4
0	0	0	0	1	2
0	0	0	0	0	0
0	0	0	0	0	0
0	0	0	0	0	1
0	0	0	0	7	8
0	0	0	6	0	0
0	0	0	0	3	0
0	0	0	0	1	2
0	0	0	0	0	0
0	0	0	0	1	0
0	0	0	0	0	0
0	0	0	0	2	5
0	0	0	0	0	0
0	0	0	0	0	0

0	0	0	0	10	0
0	1	0	0	0	0
0	0	0	0	0	0
0	0	0	0	5	7
0	0	0	0	7	4
0	0	0	0	0	0
0	0	0	0	0	0
0	0	0	0	0	0
0	0	0	0	0	0
0	0	0	0	0	1
0	0	0	0	0	0
0	0	0	0	0	5
0	0	0	0	0	0
0	0	0	0	0	0
0	0	0	0	0	1
0	0	0	0	0	0
0	0	0	0	3	72
0	0	0	0	5	37
0	0	0	0	6	62
0	0	0	0	1	10
0	0	0	0	0	13
0	0	0	0	1	1
0	0	0	0	1	0
0	0	1	0	7	7
0	0	0	0	17	2
0	0	0	0	0	0
0	0	0	1	1	6
0	0	0	0	1	0
0	0	0	0	0	0
0	0	0	0	0	2
0	0	0	0	1	2
0	0	0	0	0	0
0	0	0	0	1	0
0	0	0	0	0	0
0	0	0	0	3	1
0	0	0	0	4	0
0	1	0	0	0	0
0	0	0	0	4	7
0	0	0	0	0	2
0	0	0	0	0	0
0	0	0	0	0	0
0	0	0	0	0	0
0	0	0	0	0	0
0	0	0	0	0	1
0	0	0	0	0	0
0	0	0	0	0	0
0	0	0	0	4	5
0	0	0	0	1	0
0	0	0	0	0	0
0	0	0	0	0	0
0	0	0	0	0	0
0	0	0	0	0	0
0	0	0	0	1	0
0	0	0	0	1	0

0	0	0	0	0	0
0	0	0	0	0	0
0	0	0	0	0	0
0	0	0	0	0	1
0	0	0	0	2	0
0	0	0	0	0	0
0	0	0	0	0	0
0	0	0	0	1	0
0	0	0	0	0	0
0	0	0	0	21	12
0	0	0	0	53	14
0	0	0	0	34	10
0	0	0	0	36	5
0	0	0	0	9	2
0	0	0	0	6	2
0	0	0	0	20	0
0	0	0	0	7	0
0	0	0	0	8	0
0	0	0	0	3	0
0	0	0	0	0	0
0	0	0	0	0	0
0	0	0	0	0	0
0	0	0	0	17	0
0	0	0	0	50	0
0	0	0	0	52	0
0	0	0	0	70	0
0	0	0	0	60	0
0	0	0	0	0	0
0	0	0	0	0	0
0	0	0	0	0	0
0	0	0	0	0	0
0	0	0	0	0	0

0	0	0	0	0	0
0	0	0	0	0	0
0	0	0	0	0	0
0	0	0	0	0	0
0	0	0	0	0	0
0	0	0	0	4	2
0	0	0	0	1	0
0	0	0	0	1	0
0	0	0	0	0	0
0	0	0	0	0	0
0	0	0	0	0	0
0	0	0	1	2	0
0	0	0	0	1	1
0	0	0	0	1	1
0	0	0	0	4	1
0	0	0	0	0	0
0	0	0	0	6	2
0	0	0	0	2	1
0	0	0	0	0	0
0	0	0	0	1	0

0	0	0	0	2	1
0	0	0	0	19	0
0	0	0	0	13	0
0	0	0	0	0	0
0	0	0	0	0	0
0	0	0	0	0	0
0	0	0	0	0	0
0	0	0	0	0	0
0	0	0	0	0	0
0	0	0	0	7	5
0	0	0	0	0	3
0	0	0	0	2	2
0	0	0	0	10	1
0	0	0	0	0	0
0	0	0	0	0	0
0	0	0	0	0	0
0	0	0	0	0	0
0	0	0	0	0	1
0	0	0	0	0	0
0	0	0	0	0	0
0	0	0	0	1	0
0	0	0	0	4	4
0	0	2	0	13	0
0	0	0	0	9	0
0	0	0	0	7	0
0	0	0	0	3	0
0	0	0	0	27	0
0	0	0	0	4	2
0	0	0	0	0	0
0	0	0	0	0	0
0	0	0	0	9	0
0	0	0	0	5	0
0	0	0	0	0	0
0	0	0	0	1	1
0	0	0	0	0	1
0	0	0	0	0	1
0	0	0	0	0	0
0	0	0	0	0	0
0	1	0	0	1	0
0	0	0	0	2	0
0	0	0	0	0	0
0	0	0	0	44	0
0	0	0	0	2	0
0	0	1	1	0	0
0	0	0	0	0	0
0	0	0	0	1	2
0	0	0	0	0	0
0	0	0	0	0	0
0	0	0	0	1	0
0	0	0	0	5	0
0	0	0	0	0	0
0	0	0	0	0	0
0	0	0	0	0	0
0	0	0	0	9	4
0	0	0	0	0	0

0	0	0	0	0	0
0	0	0	0	0	0
0	0	0	0	11	2
0	0	0	1	0	2
0	0	0	0	1	3
0	0	0	0	1	3
0	0	0	0	0	0
0	0	0	0	0	0
0	0	0	0	0	2
0	0	0	0	0	0
0	0	0	0	1	1
0	0	0	0	1	2
0	0	0	0	0	1
0	0	0	0	0	0
0	0	0	0	6	0
0	0	0	0	0	0
0	0	0	0	2	0
0	0	0	0	0	0
0	0	0	0	0	0
0	0	0	0	0	0
0	0	0	0	1	1
0	0	0	0	3	0
0	0	0	0	6	0
0	0	0	0	6	4
0	0	0	0	4	5
0	0	0	0	0	0
0	0	0	0	0	0
0	0	0	0	0	0
0	0	0	0	0	0
0	0	0	0	0	0
0	0	0	0	0	0
0	0	0	0	0	0
0	0	0	0	1	0
0	0	0	0	0	0
0	0	0	0	0	0
0	0	1	0	0	2
0	0	0	0	5	0
0	0	0	0	0	0
0	0	0	0	0	0
0	0	0	0	2	0
0	0	0	0	0	0
0	0	0	1	0	0
0	0	2	0	1	2
0	0	0	0	0	0
0	0	0	0	9	3
0	0	0	0	0	0
0	0	0	0	0	0
0	0	0	0	0	0
0	0	0	0	15	13
0	0	0	0	0	0
0	0	0	0	3	3
0	0	0	0	0	0
