# Supplementary figures and images for: Relationship between Fungal Colonisation of the Respiratory Tract in Lung Transplant Recipients and Fungal Contamination of the Hospital Environment
Source: PLoS One. 2015 Dec 2;10(12):e0144044. doi: 10.1371/journal.pone.0144044 (PMC4667873; doi:10.1371/journal.pone.0144044)

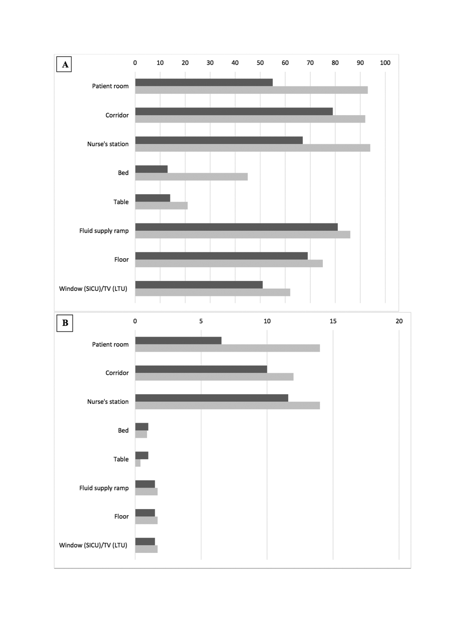

Supplement: S1 Fig — (A) for all molds and (B) for Aspergillus species in air (patient room, corridor and nurse’s station) or surface samples (bed, table, fluid supply ramp, floor, window and TV) collected in SICU (black bars) and LTU (grey bars). (TIF) [file pone.0144044.s002.tif]

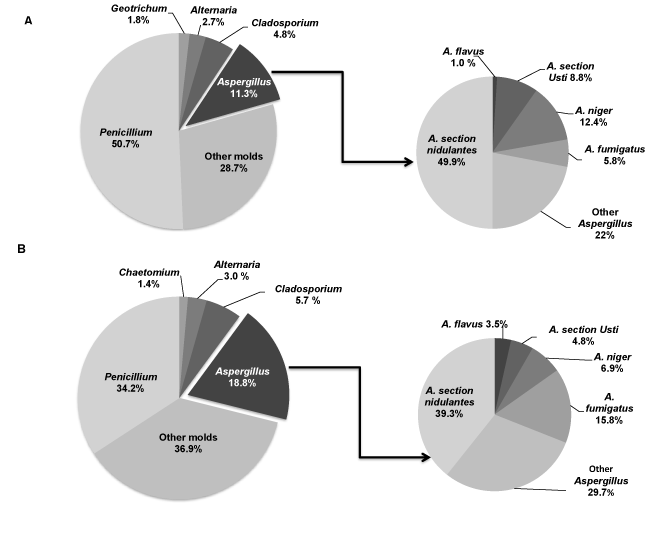

Supplement: S2 Fig — (A) in the SICU and (B) in the LTU. (TIF) [file pone.0144044.s003.tif]
